# Cell-type-specific prediction of 3D chromatin organization enables high-throughput in silico genetic screening

**DOI:** 10.1038/s41587-022-01612-8

**Published:** 2023-01-09

**Authors:** Jimin Tan, Nina Shenker-Tauris, Javier Rodriguez-Hernaez, Eric Wang, Theodore Sakellaropoulos, Francesco Boccalatte, Palaniraja Thandapani, Jane Skok, Iannis Aifantis, David Fenyö, Bo Xia, Aristotelis Tsirigos

**Affiliations:** 1 Institute for Systems Genetics, New York University Grossman School of Medicine, New York, NY, USA; 2 Department of Pathology, New York University Grossman School of Medicine, New York, NY, USA; 3 Applied Bioinformatics Laboratories, New York University Grossman School of Medicine, New York, NY, USA; 4 Perlmutter Cancer Center, NYU Langone Health, New York, NY, USA; 5 Department of Biochemistry and Molecular Pharmacology, New York University Grossman School of Medicine, New York, NY, USA; 6 Society of Fellows, Harvard University, Cambridge, MA, USA; 7 Gene Regulation Observatory, Broad Institute of MIT and Harvard, Cambridge, MA, USA; 8 Present address: The Jackson Laboratory for Genomics Medicine, Farmington, CT, USA; 9 Present address: Department of Women’s and Children’s Health, University of Padua, Padua, Italy

## Abstract

Investigating how chromatin organization determines cell-type-specific gene expression remains challenging. Experimental methods for measuring three-dimensional chromatin organization, such as Hi-C, are costly and have technical limitations, restricting their broad application particularly in high-throughput genetic perturbations. We present C.Origami, a multimodal deep neural network that performs de novo prediction of cell-type-specific chromatin organization using DNA sequence and two cell-type-specific genomic features–CTCF binding and chromatin accessibility. C.Origami enables in silico experiments to examine the impact of genetic changes on chromatin interactions. We further developed an in silico genetic screening approach to assess how individual DNA elements may contribute to chromatin organization and to identify putative cell-type-specific *trans*-acting regulators that collectively determine chromatin architecture. Applying this approach to leukemia cells and normal T cells, we demonstrate that cell-type-specific in silico genetic screening, enabled by C.Origami, can be used to systematically discover novel chromatin regulation circuits in both normal and disease-related biological systems.

In mammalian cells, interphase chromosomes are hierarchically organized into large compartments containing topologically associating domains (TADs) at the sub-megabase scale^1–3^. The genome organization is cell type-specific and largely determined by features in DNA sequence and *trans*-acting factors that regulate chromatin interactions^1–7^. Chromatin looping within TADs restricts enhancer–promoter interactions to regulate cell-type-specific gene expression^4,5,8,9^. While the general scaffold of chromatin organization is well described, revealing the mechanisms underlying cell-type-specific chromatin structure and the implications to gene expression remains challenging^10–14^. Chromatin conformation capture technologies, such as Hi-C, are typically time- and resource-consuming^1^, limiting their contribution to understanding how chromatin organization determines cell-type-specific gene expression.

Owing to its ability to model complex interactions, deep learning has emerged as a powerful approach for studying genomic features and reducing the need for experimental analyses of chromatin organization^15,16^. DNA sequence encodes motifs that act with chromatin binding proteins to define genome folding, and thus can be used to make approximate prediction of chromatin organization^17–20^. However, due to the lack of genomic features which govern cell-type-specific interactions, these approaches are unable to make accurate de novo predictions in different cell types^17–20^. Conversely, methods that rely only on chromatin profiles lacking motif features in DNA sequence often require multiple epigenomic data to improve predictive power^21–26^. These limitations render the current methods unsuitable for high-throughput in silico investigation of cell-type-specific mechanisms of chromatin organization.

We propose that an accurate de novo prediction of chromatin folding requires a multimodal neural network incorporating both DNA sequence and cell-type-specific genomic features. For practicality, it should also use a minimal set of inputs without compromising performance. Based on these principles, we developed C.Origami, a deep neural network that synergistically integrates DNA sequence features and two essential cell-type-specific genomic features: CTCF binding and chromatin accessibility (Supplementary Fig. 1). C.Origami achieved accurate de novo prediction of cell-type-specific chromatin organization in both normal and rearranged genomes.

The accuracy of C.Origami enables in silico genetic perturbation experiments that assess the impact of *cls*-elements on chromatin interactions, and, moreover, allows systematic identification of cell-type-specific mechanisms of genomic folding through in silico genetic screening (ISGS). Applying ISGS to T cell acute lymphoblastic leukemia (T-ALL) cells and normal T cells, we identified T-ALL-specific regulation of chromatin organization through *cis*-elements and cell-type-specific *trans*-regulators. Taken together, these results demonstrate that C.Origami can serve as a high-throughput in silico genetic perturbation platform for future studies of three-dimensional (3D) chromatin organization.

## Results

### C.Origami predicts cell-type-specific 3D chromatin organization

**Fig. 1 |:**
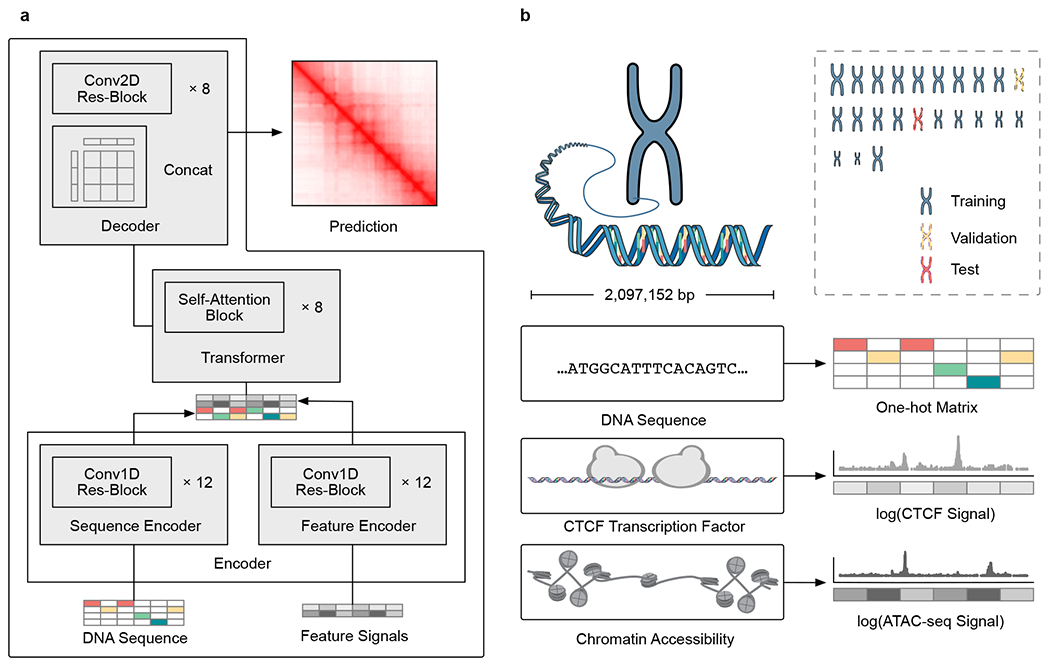
De novo, cell-type-specific prediction of 3D chromatin organization with C.Origami. **a**, A schematic of C.Origami architecture. **b**, C.Origami integrates DNA sequence, CTCF ChIP–seq and ATAC–seq signals as input features to predict Hi-C interaction matrix in 2-Mb windows. Concat, concatenation; Conv1D, one-dimensional convolution; Conv2D, two-dimensional convolution; res-block, residual network block.

To achieve accurate and cell-type-specific prediction of genomic features, we first developed Origami, a generic multimodal architecture to integrate both nucleotide-level DNA sequence feature and cell-type-specific genomic signal (Fig. 1a). Origami adopts an encoder–decoder design with two encoders, a transformer module and a task-specific decoder (Fig. 1a and Methods). The two encoders are one-dimensional (1D) convolutional neural networks that condense DNA sequence and genomic features. Condensed sequence and genomic feature representations are subsequently concatenated and processed by a transformer module, which enables long-range information exchange^27^. The decoder transforms the processed features to make task-specific predictions. In this study, we deployed a two-dimensional (2D) convolutional neural network with a large receptive field as a decoder and named this variant model Chromatin Origami (C.Origami) for predicting chromatin organization as captured by Hi-C contact matrices (Fig. 1a and Methods).

C.Origami predicts chromatin organization within a 2-megabase (2-Mb) window to cover typical TADs, and outputs a Hi-C contact matrix with a bin size of 8,192 base pairs (bp) (Fig. 1b and Methods)^11^. As potential inputs to the model, we considered genomic features that are cell-type-specific and widely available, and as few as possible without compromising model performance. CTCF binding is one of the most critical determinants of genome organization into TADs^2^. In addition, previous studies revealed that at accessible chromatin regions, interactions between enhancers and promoters contribute substantially to cell-type-specific chromatin organization^28–30^. Based on these insights, we considered CTCF chromatin immunoprecipitation followed by sequencing (ChIP–seq), assay for transposase-accessible chromatin using sequencing (ATAC–seq) and DNA sequence features as potential inputs that can informatively contribute to predicting cell-type-specific 3D chromatin organization (Fig. 1b).

**Extended Data Fig. 1 |:**
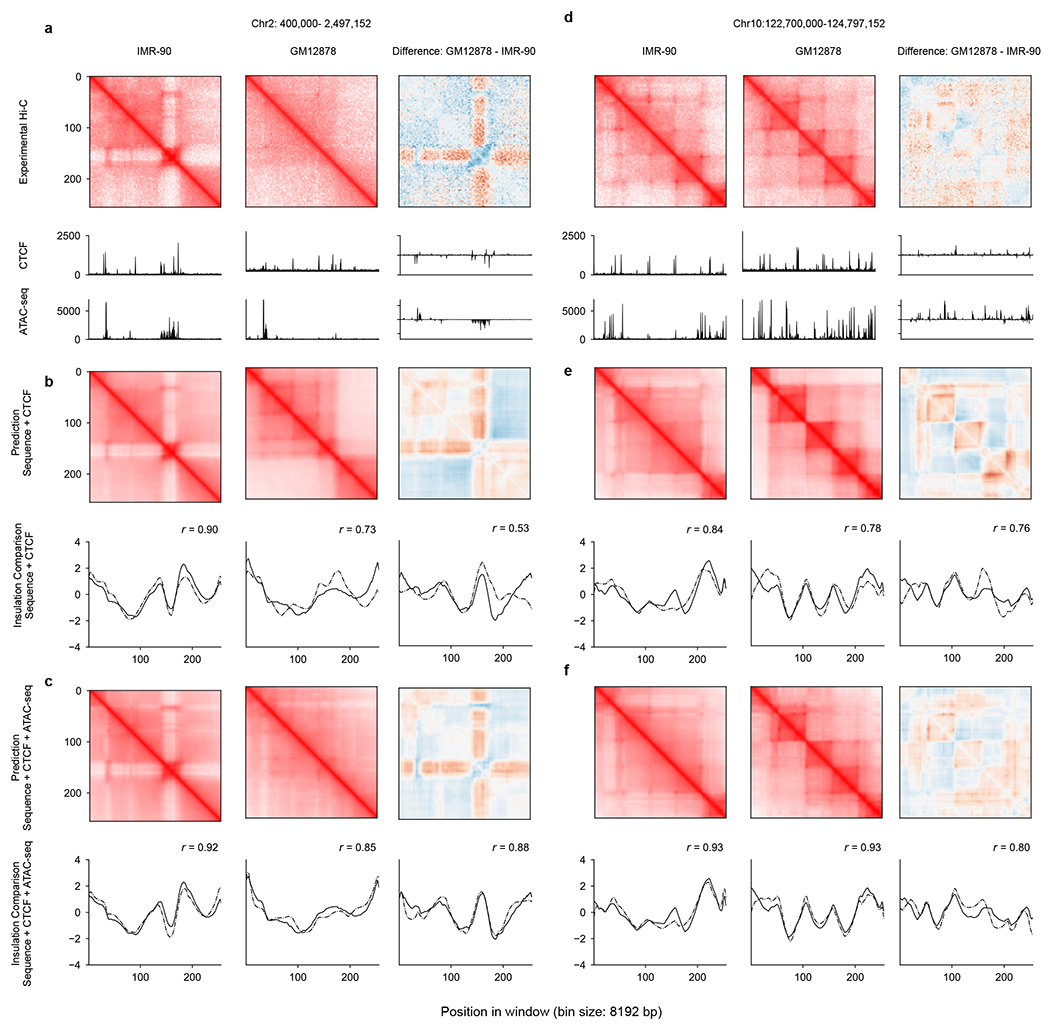
C.Origami trained with DNA sequence, CTCF binding and chromatin accessibility profiles performed optimally. **a**, Experimental Hi-C matrices and genomic profiles of IMR-90 and GM12878 cells at chr2: 400,000–2,497,152. The difference between the two cell lines were presented on the right. **b**–**c**, Cell-type-specific prediction of the chromatin organization at the same locus using C.Origami (**b**) or model trained with DNA sequence and CTCF binding (**c**). **d**–**f**, Same as **a**–**c** at a difference locus, chr10: 122,700,000–122,797,152.

**Fig. 2 |:**
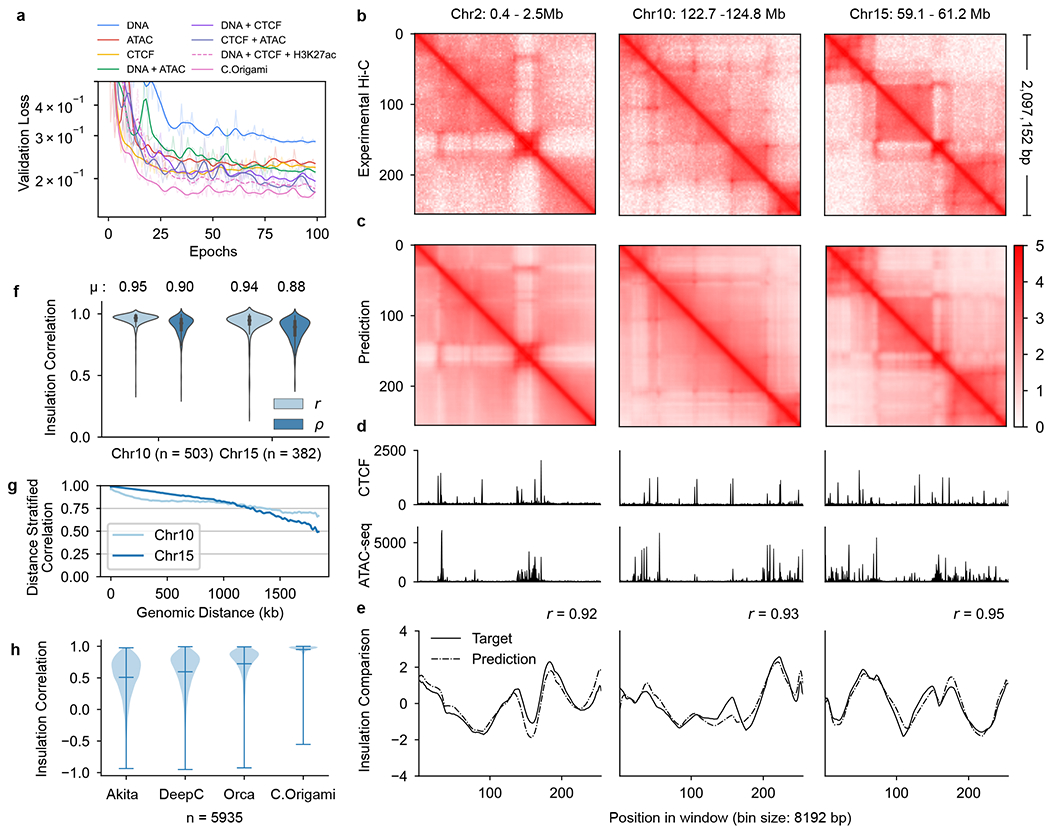
C.Origami accurately predicts 3D chromatin organization. **a**, Validation loss of models trained from different combinations of input features. Lower validation loss indicates better model performance. **b,c**, Experimental (**b**) and C.Origami-predicted (**c**) Hi-C matrices of IMR-90 on training (chr2), validation (chr10) and test (chr15) chromosomes. **d**, Input CTCF-binding and chromatin accessibility profiles. **e**, Insulation scores calculated from experimental (solid line) and C.Origami-predicted (dotted line) Hi-C matrices. Pearson correlation coefficients between prediction and target insulation scores are presented. **f**, Insulation score correlation between predicted and experimental Hi-C matrices across all windows in both validation and test chromosomes with Pearson (*r*) and Spearman (*ρ*) correlation coefficients. **g**, Chromosome-wide distance-stratified interaction correlation (Pearson) between prediction and experimental data. **h**, Comparison of model performance across Akita, DeepC, Orca and C.Origami using genome-wide insulation score correlation between prediction and experimental data from IMR-90 cells. Error bars in the violin plots indicate minimum, mean and maximum values. *μ*, average insulation correlation.

To examine model performance with different combinations of inputs, we trained the model using all possible combinations of the three potential input features using data from IMR-90 cells (Fig. 2a)^13^, and randomly split the chromosomes into training, validation (chromosome 10) and test sets (chromosome 15) (Fig. 1b). We found that C.Origami trained with DNA sequence, CTCF ChIP–seq and ATAC–seq achieved the best performance, accurately predicting contact matrices emphasizing both topological domains and chromatin loops (Fig. 2a–d and Methods). Removing or replacing any of the three input features during model training led to compromised performance (Fig. 2a, Extended Data Fig. 1 and Supplementary Fig. 2). Ablating any of the input features during model inference led to inferior prediction (Supplementary Fig. 3). Notably, adding DNA sequence to the genomic features during model training always led to substantially improved performance (Fig. 2a). Last, we trained the model using sparse input genomic features (ChIP–seq/ATAC–seq peaks) and found that it underperformed compared with dense features, indicating C.Origami’s capability of leveraging nuanced genomic features beyond peak positions and intensities (Supplementary Fig. 4).

### Genome-wide evaluation of C.Origami performance

**Extended Data Fig. 2 |:**
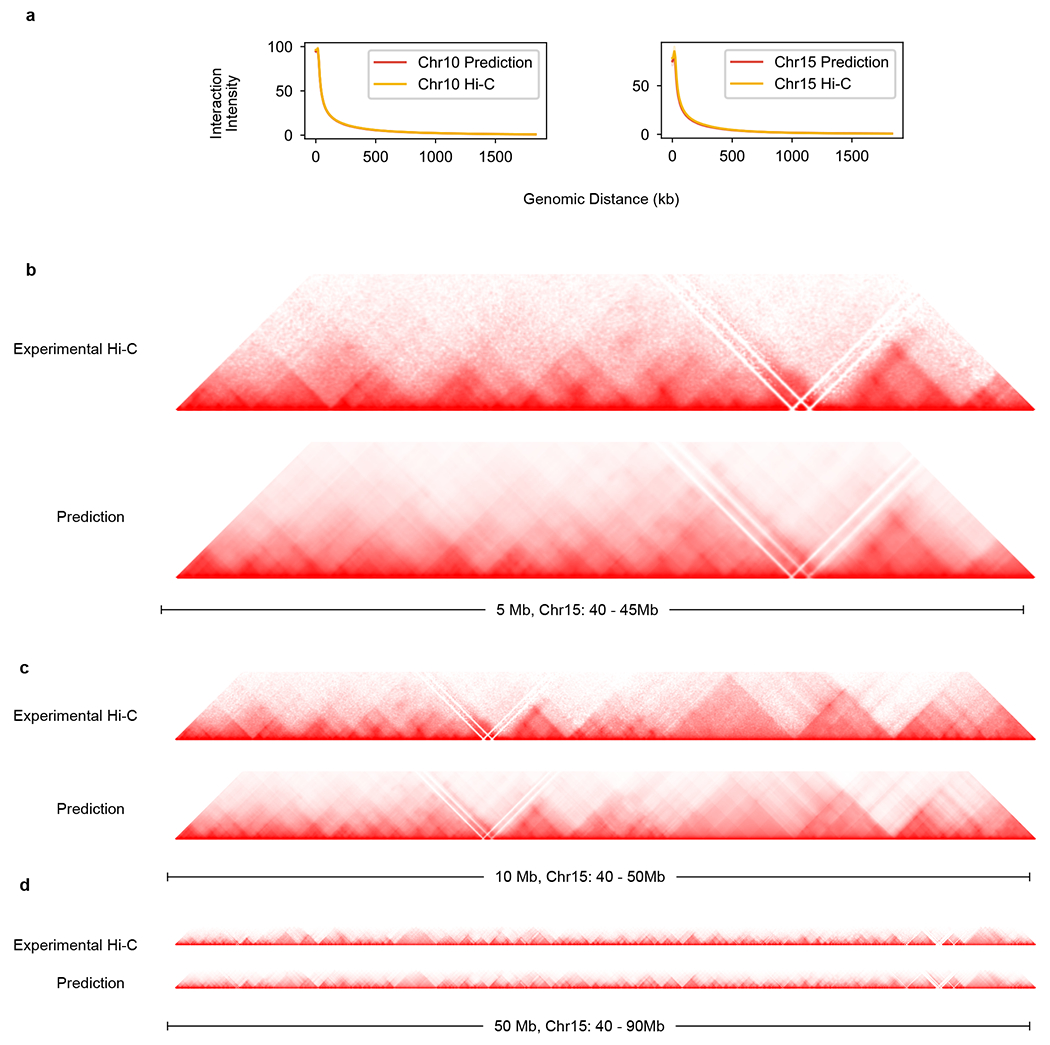
C.Origami-predicted 2 Mb Hi-C maps can be fused into larger interaction maps. **a**, Interaction intensity distribution of prediction and experimental Hi-C on validation (chromosome 10) and test chromosomes (chromosome 15). **b**–**d**, The predicted 2 Mb Hi-C maps were fused to 5 Mb (**b**), 10 Mb (**c**) and 50 Mb (**d**) on chromosome 15, all with the same starting site at 40 Mb.

To systematically assess C.Origami’s performance, we first calculated insulation scores to evaluate chromatin organization similarity between experimental and predicted Hi-C matrices (Methods and Fig. 2e). C.Origami achieved on average 0.95 and 0.94 insulation score correlation (Pearson) on validation and test chromosomes, respectively (Fig. 2f and Supplementary Fig. 5). C.Origami-predicted contact matrices also follow the same exponential decay pattern observed in experimental data (Extended Data Fig. 2a). In addition, we found that predicted contact matrices were stable across neighboring regions, enabling constructions of chromosome-wide predicted Hi-C matrices (Extended Data Fig. 2b–d and Methods). Based on the chromosome-wide predicted contact matrices, we calculated a distance-stratified correlation against experimental Hi-C (Methods). C.Origami achieved correlation above 0.8 within a 1-Mb region (Fig. 2g and Supplementary Fig. 6).

**Extended Data Fig. 3 |:**
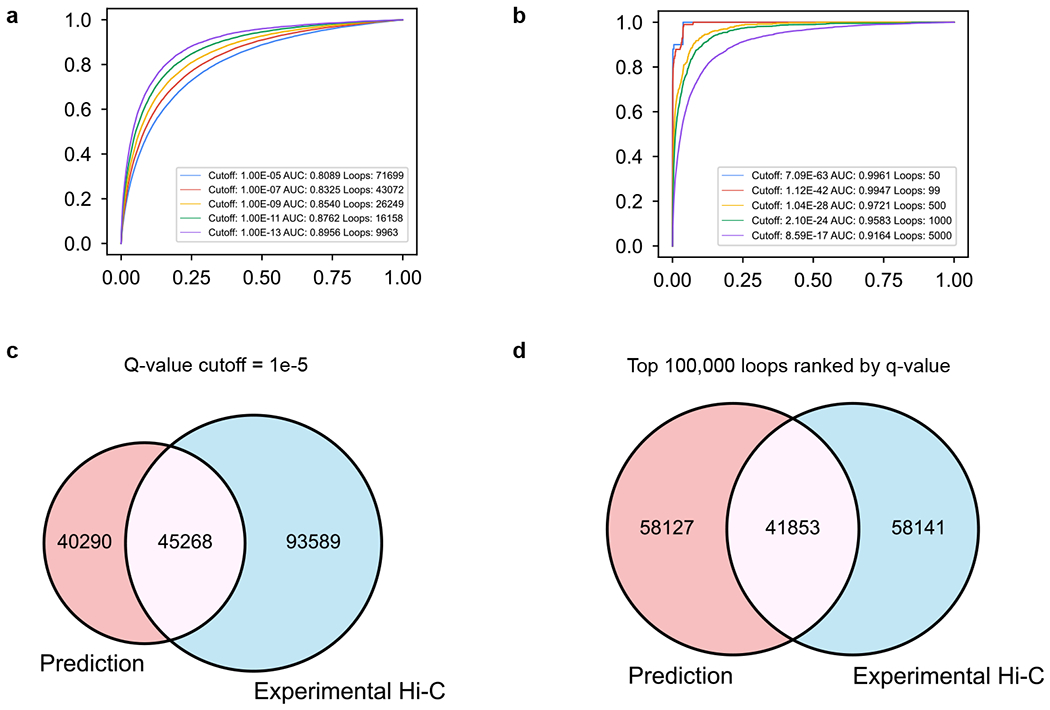
Evaluating C.Origami’s performance on detecting significant chromatin loops in IMR-90 cells. **a**, ROC curves of significant chromatin loops called in experimental Hi-C and prediction. Significant chromatin loop referring to global background were called at different *Q* value ranging from 1 × 10^−5^ to 1 × 10^−13^ from predicted Hi-C matrices. *Q* value of experimental Hi-C was ranked against predicted loops to calculate AUROC. Each curve represents an ROC curve comparing experimental Hi-C *Q* value to predicted loops with specific cutoffs. **b**, ROC curves of top 50 to top 5000 loops with corresponding *Q* value cutoffs. AUROC under each criterion is indicated in legends of **a** and **b**. **c**–**d**, Venn diagram of chromatin loop overlapping between experiment and prediction with *Q* value cutoff of at 1e-5 (**c**) or between the top 100,000 loops (**d**). All loop calling was carried out with global background as reference to increase sensitivity to all significant chromatin interactions.

Loop calling identifies point-to-point interactions from Hi-C matrix. To further evaluate C.Origami’s performance, we performed loop calling on both prediction and experimental Hi-C in IMR-90 cells (Methods). We found that C.Origami achieved high performance in loop detection, with an area under the receiver operating characteristic curve (AUROC) of 0.92 for the top 5,000 predicted loops (Extended Data Fig. 3). We categorized these loops into CTCF–CTCF loops, promoter-enhancer loops and promoter–promoter loops. We found that, in each category, C.Origami-predicted matrices can be applied to call chromatin loops comparably to the performance of experimental results (Supplementary Fig. 7).

Last, we compared C.Origami against three recent sequence-based approaches: Akita^18^, DeepC^19^ and Orca^20^. After preprocessing and standardizing the results from different methods (Methods and Supplementary Figs. 8–10), we used four metrics to evaluate the performance of each model: (1) insulation score correlation, (2) observed/expected Hi-C map correlation, (3) mean squared error (MSE) and (4) distance-stratified correlation (Methods). We found that C.Origami outperforms previous methods under all metrics (Fig. 2h and Supplementary Fig. 11).

### De novo prediction of cell-type-specific chromatin organization

**Extended Data Fig. 4 |:**
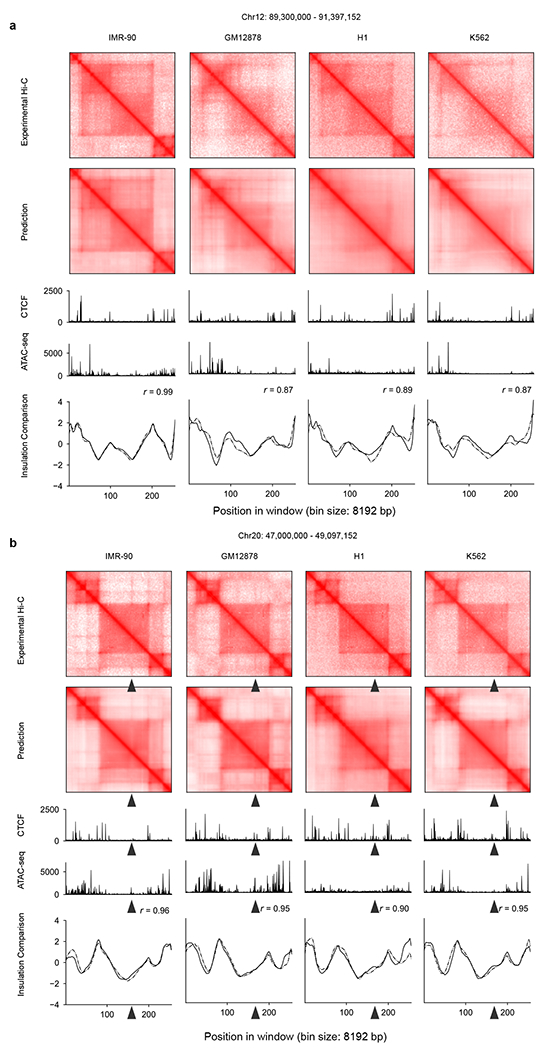
C.Origami predicts chromatin organizations across multiple cell types. Two representative loci were separately presented across IMR-90, GM12878, H1-hESCs and K562 in **a** and **b**. From top to bottom, each panel included experimental Hi-C matrix, predicted Hi-C matrix, CTCF and ATAC–seq signals, and insulation scores calculated from experimental and predicted Hi-C data.

**Fig. 3 |:**
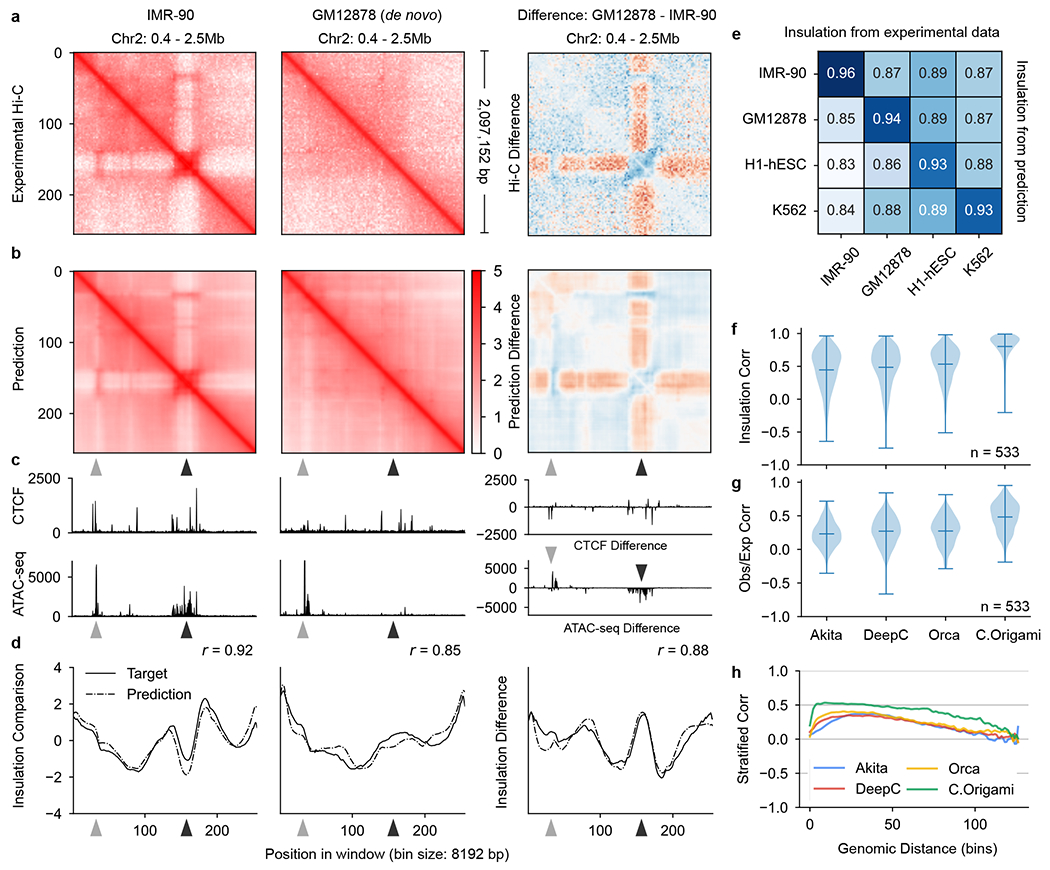
Cell-type-specific de novo prediction of chromatin structure. **a,b**, Experimental (**a**) and C.Origami-predicted (**b**) Hi-C matrices from IMR-90 (left) and GM12878 (middle), and their differences (right). Arrowheads highlight differential chromatin interactions between the two cell types. **c**, CTCF-binding and ATAC𡀓seq profiles. **d**, Insulation scores calculated from experimental Hi-C matrices (solid line) and C.Origami-predicted Hi-C matrices (dotted line). **e**, Pearson correlation between insulation scores calculated from predicted and experimental Hi-C matrices across cell types. **f**–**h**, Genome-wide evaluation of sequence-based models and C.Origami using de novo prediction results from GM12878 cells. Presented metrics include insulation score correlation (**f**), observed versus expected matrix correlation (**g**) and distance-stratified correlation (**h**). Error bars in violin plots of **f** and **g** indicate minimum, mean and maximum values within each group. Corr, correlation; obs/exp, observed/expected.

To assess C.Origami’s performance in a cell-type-specific de novo prediction task, we applied the model to a new cell type, GM12878, using its corresponding CTCF ChIP–seq and ATAC–seq profiles. GM12878 is a lymphoblastoid cell line with different chromatin organization from IMR-90 (ref. 13), represented by a locus on chromosome 2 (Fig. 3a). Comparing predictions in both IMR-90 and GM12878 cells at the same locus, we found that C.Origami transferred successfully to the new cell type and accurately predicted cell-type-specific chromatin interactions in GM12878 (Fig. 3a–c). Insulation scores calculated from predicted and experimental data in GM12878 are also highly correlated (Fig. 3d). We further expanded de novo prediction to two more cell lines, embryonic stem cells (H1-hESCs) and erythroleukemia K562 cells, and achieved the same accurate predictions, demonstrating the robustness of C.Origami and its practical potential for broader applications (Extended Data Fig. 4).

**Extended Data Fig. 5 |:**
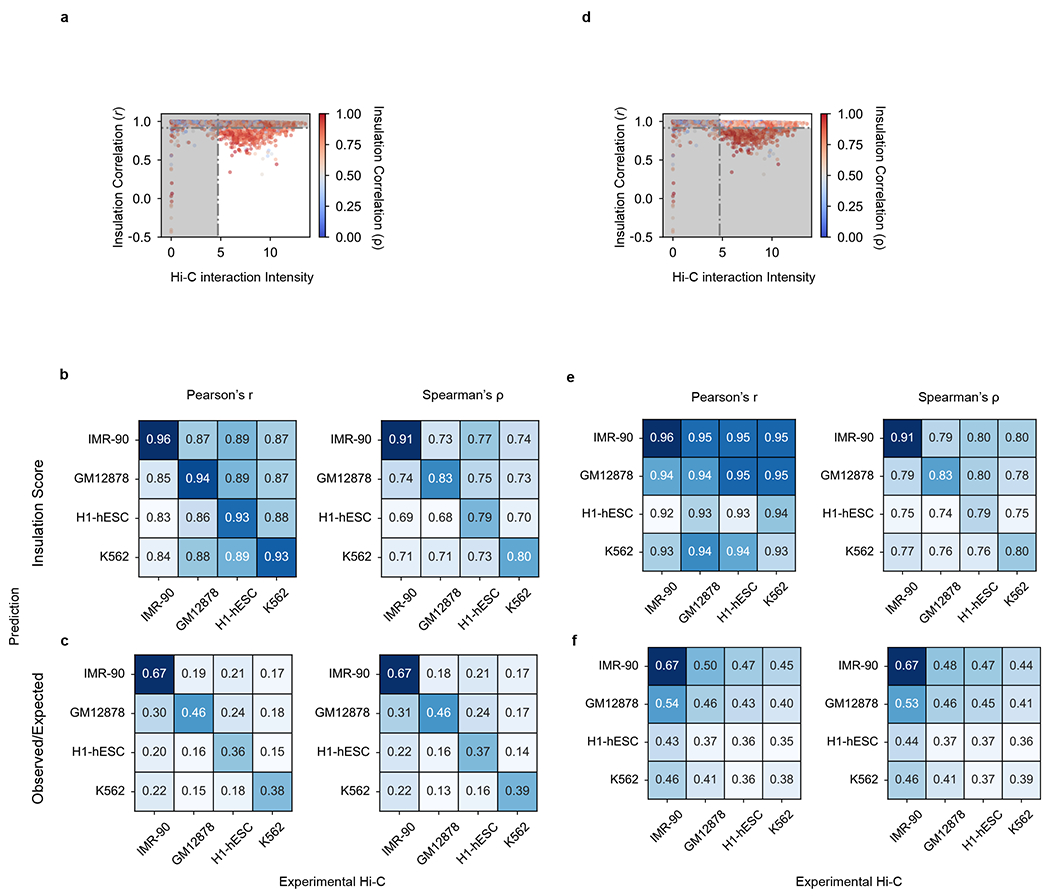
Genome-wide statistics on cell-type-specific prediction. **a**, The distribution of interaction intensity by insulation correlation (Pearson) between the experimental Hi-C matrices of IMR-90 and GM12878. Dotted lines denote the filtering criteria in selecting representative loci with cell-type specificity. Colormap indicates the corresponding Spearman correlation coefficient (*ρ*). **b**–**c**, Pearson’s *r* (left) and Spearman’s *ρ* (right) between prediction (row) and experimental data (column) for different cell types with insulation score (**b**) and observed/expected score (**c**) as metrics. Diagonal entries denote the metrics of prediction and Hi-C in the same cell type without filtering for cell-type-specific regions. The scores were calculated based on the differentially structured loci defined in Fig. 3. **d**, The distribution of interaction intensity selected for structurally conserved regions between two cell types, similar to **a**. **e**–**f**, Same as **b**–**c** but for the structurally conserved loci across different cell types.

To systematically evaluate the performance of C.Origami in de novo prediction, we carried out a genome-wide analysis. Since most TAD boundaries are conserved across cell types^12^, we first identified subsets of genomic loci with differential chromatin structures as testing regions, representing around 15% of the genome (Extended Data Fig. 5a and Methods). We performed this filtering process for each pair of cell types in the confusion matrix followed by evaluating model performance in these regions. In line with observations from the single-locus results (Fig. 3a–d), we found that predictions using input features from one cell type have the highest correlation coefficients with the experimental Hi-C data of the same cell type (Fig. 3e and Extended Data Fig. 5b,c, scores at the diagonal line). As a control, we performed a similar analysis using structurally conserved genomic regions and found universally high correlations across all cell types as expected (Extended Data Fig. 5d–f).

As an orthogonal validation, we performed loop calling on IMR-90 and GM12878 prediction and experimental Hi-C to evaluate C.Origami’s ability to detect cell-type-specific chromatin loops. We found that C.Origami can predict notable (log_2_ fold change (fc) > 1) IMR-90-specific and GM12878-specific loops with 0.88 and 0.87 AUROC, respectively (Supplementary Fig. 12). Calling cell-type-specific loops under different categories also achieved similar performance (Supplementary Fig. 13).

**Extended Data Fig. 6 |:**
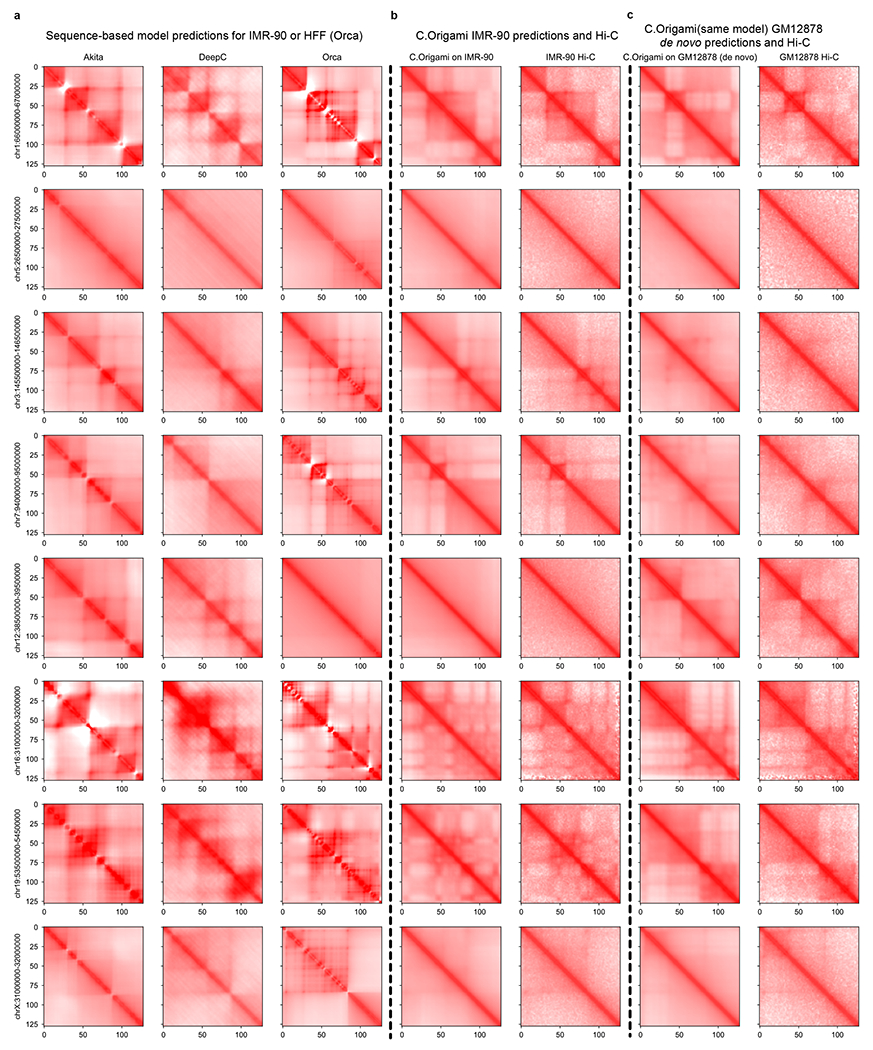
Randomly selected examples of cell-type-specific predictions from Akita, DeepC, Orca and C.Origami. **a**, Sequence-based model predictions, **b**, C.Origami prediction with IMR-90-specific genomic features (CTCF ChIP–seq and ATAC–seq) and IMR-90 experimental Hi-C. **c**, C.Origami de novo prediction with GM12878-specific genomic features and GM12878 experimental Hi-C. All presented results were aligned at randomly selected regions from different chromosomes. The full set of prediction results across all cell-type-specific chromatin regions between IMR-90 and GM12878 cells were included in the Supplementary material under Cell-type-specific predictions.

**Extended Data Fig. 7 |:**
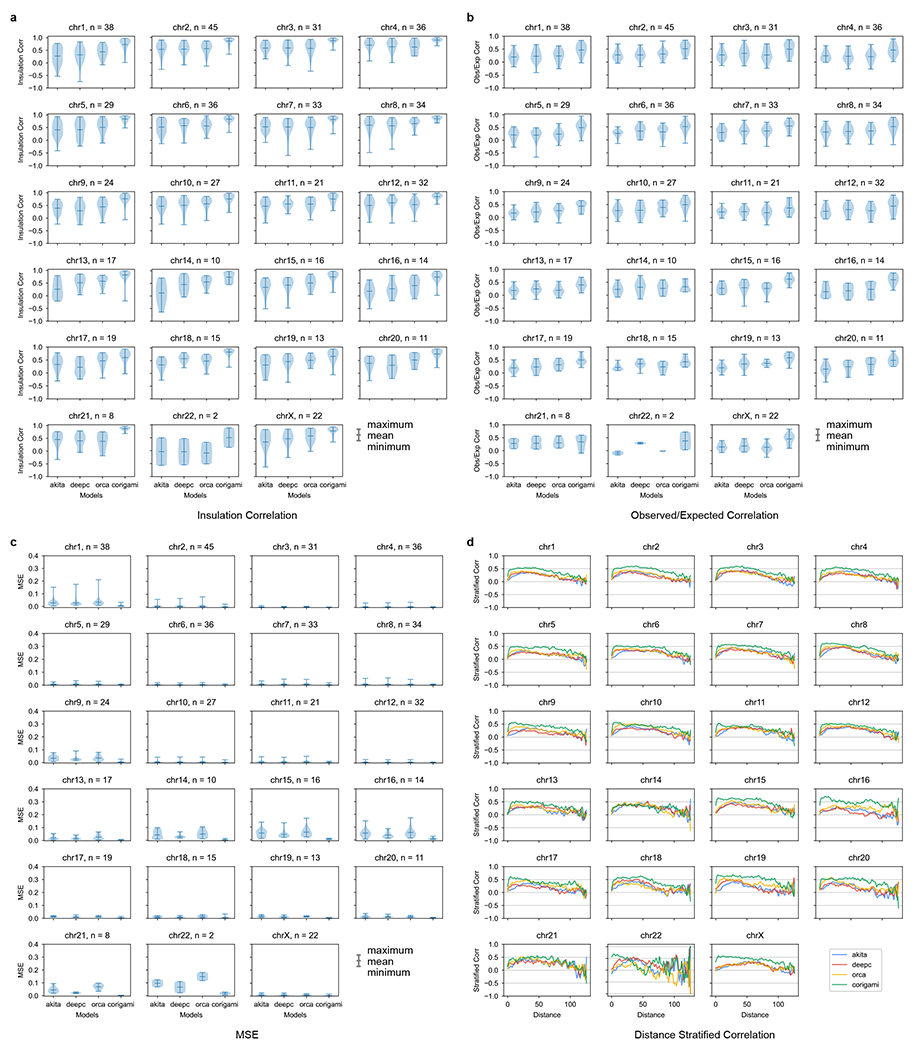
Genome-wide comparison of de novo prediction quality in GM12878. For de novo prediction results from each model (Akita, DeepC, Orca and C.Origami), we measured insulation score correlation (**a**), observed versus expected Hi-C matrices correlation (**b**), mean squared error (MSE) (**c**) and distance-stratified correlation (**d**). Prediction results at cell-type-specific regions between IMR-90 and GM12878 cells were selected for this analysis. Error bars in the violin plots indicate minimum, mean and maximum values within each group.

Since DNA sequence-based models are unable to generalize to unseen cell types, we expect C.Origami to have an advantage in cell-type-specific de novo prediction. This performance gap can be observed by comparing de novo predictions generated by sequence-based models and by C.Origami (Extended Data Fig. 6). Comparing genome-wide de novo predictions in regions with cell-type-specific chromatin organization (Methods), we found that C.Origami outperformed sequence-based models by a large margin under all metrics (Fig. 3f–h and Extended Data Fig. 7).

The mouse genome differs from human in its genomic components but the two share similar mechanisms in 3D chromatin organization^11,2 ,31^. We sought to test whether C.Origami could perform de novo prediction across species. We found that the model trained with data from human IMR-90 cells predicted mouse chromatin organization, indicating that C.Origami can transfer its learned genome organization principles to predictions across conserved species (Supplementary Fig. 14). Notwithstanding its good performance, the performance of C.Origami prediction in mouse can be further improved by training a model using mouse data to account for species-specific genomic features.

Last, we tested whether C.Origami is able to predict the chromatin organization changes upon removal of key *trans*-acting regulators, such as CTCF. A previous study found that acute degradation of CTCF protein led to the dissolving of TADs in mouse embryonic stem cells, and subsequent restoration of CTCF reestablished TAD structures^32^. We simulated such experiments by predicting chromatin organization in pre-depletion, CTCF-depleted and CTCF-restored conditions (Methods). We found that C.Origami accurately predicted the TAD loss and reformation upon CTCF depletion and restoration, respectively (Supplementary Fig. 15).

### C.Origami enables cell-type-specific in silico genetic experiments

**Fig. 4 |:**
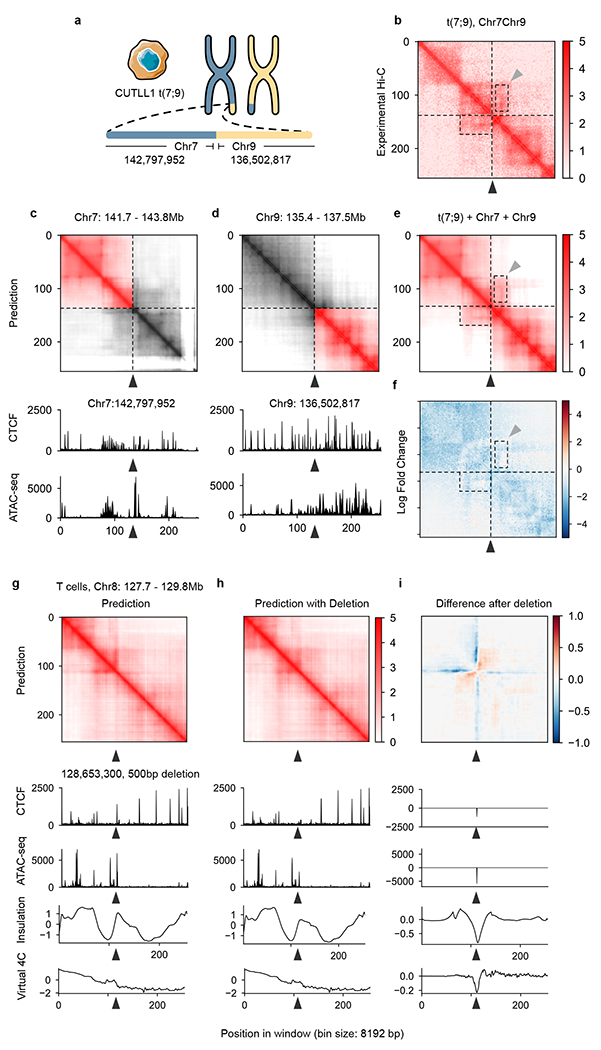
C.Origami enables prediction of 3D chromatin organization upon in silico genetic perturbations. **a**, Chromosomal translocation t(7;9) in CUTLL1 cells^35^. **b**, Experimental Hi-C data mapped to a custom reference chromosome with t(7;9) translocation^36^. **c**,**d**, C.Origami prediction of chromatin organization in intact chromosome 7 (**c**) and chromosome 9 (**d**), each centered at the translocation sites in CUTLL1 cells. **e**, C.Origami-predicted Hi-C matrix. The dotted boxes highlight the neo-TAD at the translocation locus. Black and gray arrowheads indicate the translocation site and a stripe in the neo-TAD, respectively. **f**, log-transformed fold change between experimental and predicted Hi-C matrix at t(7;9) translocation locus. **g**–**i**, A 500-bp deletion of a CTCF-binding site at *MYC* locus (arrowhead)^36^ led to chromatin looping changes in T cells. The 2-Mb window starts at the promoter region of *MYC*^36^. The presented results include C.Origami-predicted contact matrices with (**g**) or without (**h**) the deletion, and their difference (**i**). Virtual 4C signals calculated from predicted Hi-C matrices are shown at the bottom.

Chromosomal translocations and other structural variants generate recombinant DNA sequences, subsequently inducing chromatin interactions which may be critical in tumorigenesis and progression^33,34^. However, the allelic effect and high heterogeneity of structural variations make it challenging to study their custom chromatin organizations. As an example, CUTLL1, a T-ALL cell line, incorporated a heterozygous t(7;9) translocation^35^ (Fig. 4a). The translocation introduces a neo-TAD structure with a stripe which can be observed in experimental Hi-C data (Fig. 4b and Methods)^36^.

**Extended Data Fig. 8 |:**
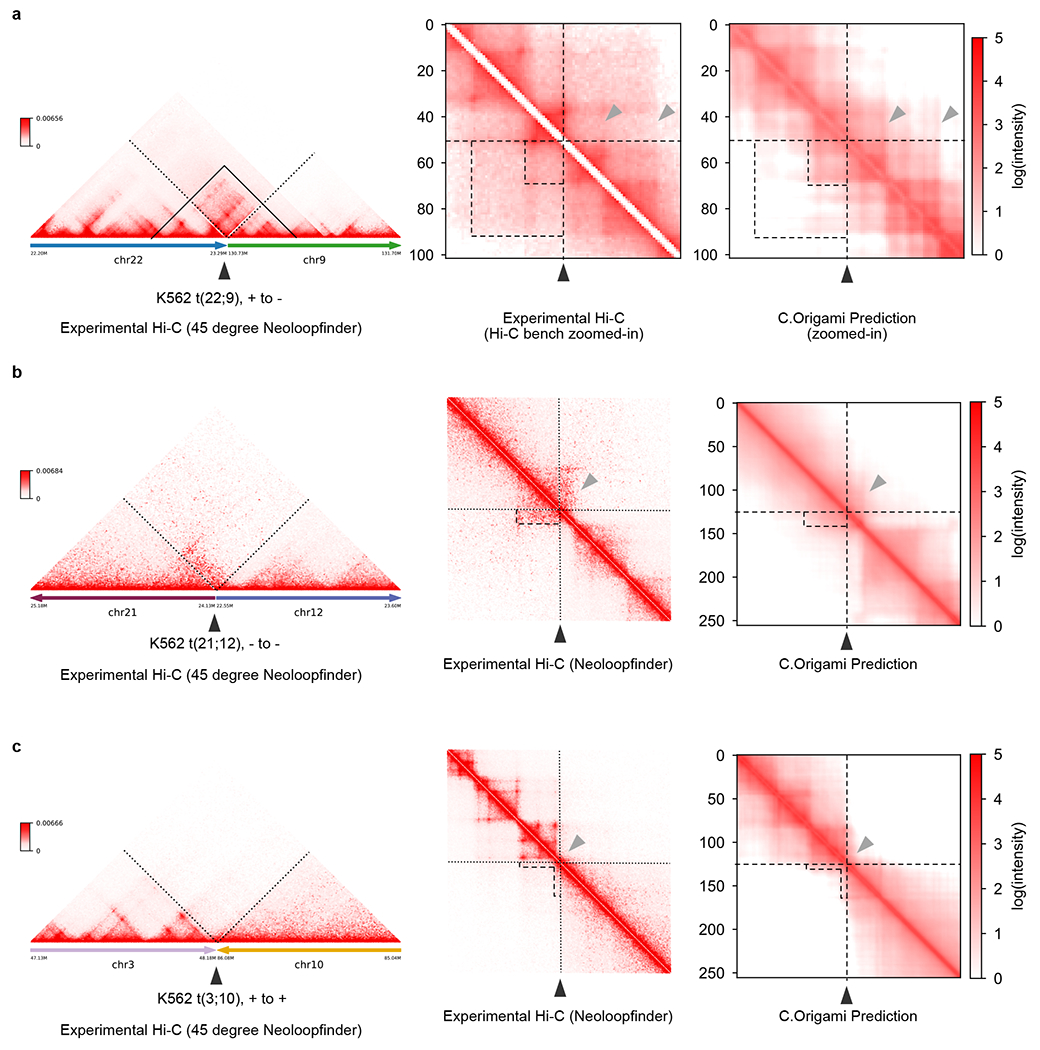
Predicting translocation-induced chromatin organizations and neo-TADs in K562 cells. **a**–**c**, Experimental and predicted Hi-C matrices at three translocation loci in K562 cells. In each case, chromatin organization structures were first reconstructed using HiC-bench^56^ and NeoLoopFinder^37^, followed by C.Origami prediction at the translocation loci using in silico fused genomic information. **a**, t(22;9) translocation, also known as the Philadelphia chromosome, that leads to a fused gene *BCR-ABL1*. **b**, t(21;12) translocation with a stripe interaction. **c**, t(3;10) translocation with a faint ‘L’-shape interaction as indicated by the dotted contour. Dotted boxes indicate neo-TAD forming at the translocation site. Black arrowhead indicates the translocation site.

To examine the performance of C.Origami in discovering new chromatin interactions in rearranged cancer genomes, we predicted Hi-C contact matrices at the translocation locus in CUTTL1 (Fig. 4c–e and Methods). We found that C.Origami prediction accurately captured the neo-TAD structure spanning t(7;9) translocation (Fig. 4e,f). Specifically, we found a stripe extending from translocated chromosome 9 to chromosome 7, indicating a regulation of the affected oncogene (*NOTCH1*) within the neo-TAD (Fig. 4b,e)^35,36^. We additionally performed the same in silico experiments around three experimentally verified translocation breakpoints in K562 cells and obtained similarly accurate results^37^, demonstrating C.Origami’s potential in cancer genomics studies (Extended Data Fig. 8).

Moreover, we expect that the high performance of C.Origami can enable cell-type-specific in silico genetic perturbation experiments as an efficient approach for studying chromatin interaction mechanisms. As an example, verifying the function of a specific CTCF-binding event in chromatin organization requires complicated experimental studies^38–41^. Using C.Origami, deletions of CTCF-binding and subsequent prediction of Hi-C contact matrix can be performed in silico within seconds. We found that in silico deletion at TAD boundaries with CTCF binding led to domain-merging events between the originally insulated adjacent TADs (Supplementary Fig. 16).

Our previous study showed that disrupting a CTCF-binding site near the *MYC* locus reduced chromatin looping in human naive CD4^+^ T cells, resulting in reduced chromatin insulation^36^. Applying C.Origami prediction to the *MYC* locus in T cells, we found a stripe in the predicted Hi-C matrix at the CTCF-binding site (Fig. 4g, arrowhead). A 500-bp in silico removal of the CTCF-binding region attenuated the stripe (Fig. 4h), and reduced its looping with *MYC* (Fig. 4i, virtual 4C), consistent with previous experimental data (Supplementary Fig. 7E in Kloetgen et al.)^36^. Similarly, the *DXZ4* locus is critical for determining chromosomal organization in X chromosome inactivation^42^. We found that deleting the *DXZ4* locus led to substantial loss of insulation at the flanking regions in female cell lines only (IMR-90, GM12878) and not in male cell lines (CUTLL1 and Jurkat, Supplementary Fig. 17), consistent with experimental knock-out results^42^.

### ISGS of putative *cis*-regulatory elements

**Extended Data Fig. 9 |:**
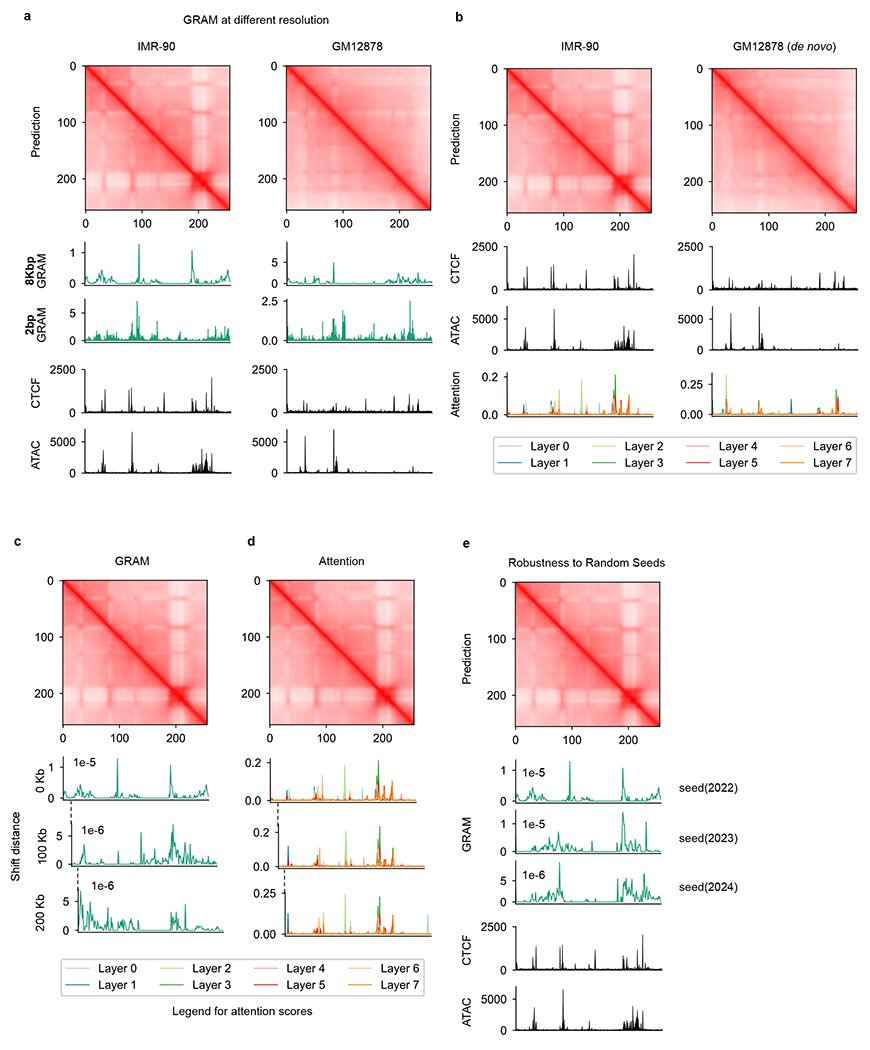
Comparing GRAM and attention scores. **a**, Comparison of GRAM scores at 2 bp and 8 kb resolution in IMR-90 (left) and GM12878 (right). **b**, Attention scores on IMR-90 and GM12878. Attention scores on different layers were colored according to legends. **c**–**d**, Comparison between GRAM (**c**) and attention scores (**d**) at three consecutive windows with 100Kb shifts. **e**, GRAM scores generated at different PyTorch random seeds.

**Fig. 5 |:**
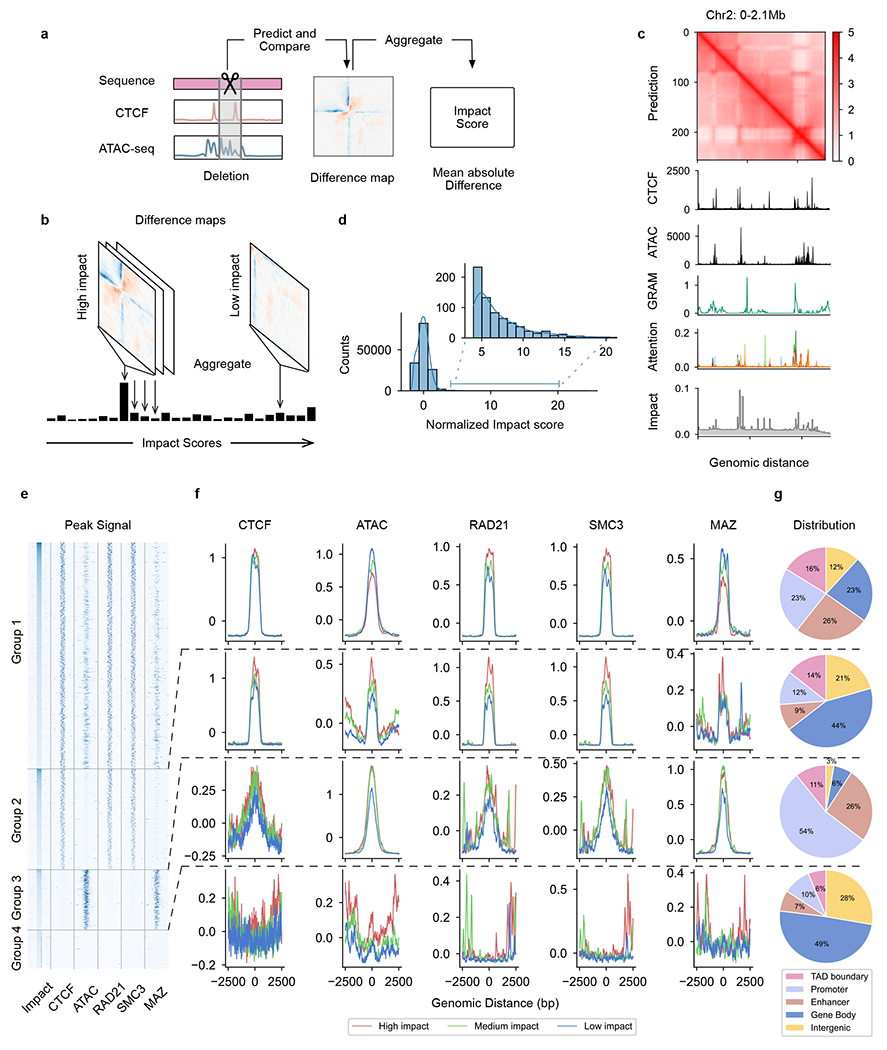
High-throughput ISGS identifies *cis*-regulatory elements determining chromatin organization. **a**, Schematic of impact score calculation from in silico deletion of *cis*-regulatory elements. **b**, Schematic of ISGS framework for identifying impactful *cis*-regulatory elements. **c**, Visualization of the three attribution methods: GRAM, attention score and impact score. **d**, Distribution of normalized impact scores in in silico deletion screening. **e**, Heatmap of ISGS-identified impactful *cis*-elements. Each row shows a 5-kb locus centered by an impactful 1-kb *cis*-element, ranked in descent by their impact scores. **f**, Relative enrichment (*Z*-score normalized) of ATAC–seq signal and multiple ChIP–seq signals at the four groups of impactful elements. According to the impact score values, *cis*-elements of each group were further grouped into high-, medium- and low-impact quantile groups when plotting the ChIP–seq/ATAC–seq signals. **g**, Characterization of in silico screening-identified *cis*-elements by their genomic annotations.

Identifying *cis*-regulatory elements required for chromatin organization is critical for 3D genome studies^43^. We propose using C.Origami to systematically and quantitatively assess how individual DNA elements contribute to the 3D chromatin organization (Fig. 5). Building on C.Origami’s model architecture, we first developed two fast approaches for identifying critical *cis*-elements: a gradient-based saliency method named Gradient-weighted Regional Activation Mapping (GRAM), and an attention-based score derived from the transformer module (Methods). Both metrics captured regions that determine genome structure, such as TAD boundaries (Fig. 5c). In particular, GRAM can be positioned at the bottom layer to obtain attribution maps at nucleotide-level resolution (Extended Data Fig. 9a). However, it is not stable to window shifts and random seed changes (Extended Data Fig. 9c–e). In contrast, the layer-specific attention score averaged across all channels of attention heads is more robust (Extended Data Fig. 9b,d). Visualization of all attention weights revealed that different attention heads attend to specific regions (Supplementary Fig. 18). While both approaches can quickly estimate the contribution of *cis*-elements, neither of them quantitatively assessed how much a specific DNA element influences local chromatin organization.

Inspired by the mechanism of reverse genetic screening, we developed an ISGS framework for identifying *cis*-regulatory elements required for chromatin organization. Different from qualitative GRAM and attention scores, ISGS quantifies the difference in C.Origami predictions upon systematic perturbations (deletions) of input elements (Methods). As an example, we first carried out ISGS in a 2-Mb window (chr2: 0–2.1 Mb) by sequentially perturbing 256 loci of ~8-kb lengths, followed by assessing Hi-C contact map changes through C.Origami prediction. We quantify the impact of a perturbation via a metric termed impact score, calculated as the mean absolute difference between predictions before and after perturbation (Fig. 5a,b and Methods). We found that perturbations at TAD boundaries with enriched CTCF ChIP–seq and ATAC–seq signals had higher impact on chromatin folding, consistent with the GRAM and attention scores (Fig. 5c).

To systematically identify the impactful *cis*-elements that are required for 3D chromatin organization, we conducted a genome-wide 1-kb-resolution ISGS (Fig. 5d). By examining the local impact scores, we isolated a set of impactful *cis*-elements representing ~1% of the screened genome (Methods). According to the presence or absence of CTCF-binding and ATAC–seq signals, these impactful *cis*-elements were classified into four groups (Fig. 5e). More than half of the impactful *cis*-elements are in open chromatin and cobound by CTCF (Group 1, Fig. 5e). Plotting CTCF-binding and ATAC–seq signals across *cis*-elements in three quantiles, we found that CTCF-bound *cis*-elements intensity stays overall the same across Group 1 and Group 2 quantiles, while the ATAC–seq signals are negatively correlated with the impact scores (Fig. 5f, top). Meanwhile, Group 1 and Group 2 elements are enriched with RAD21 and SMC3 binding signals, indicating chromatin organization through loop extrusion (Fig. 5f). Consistently, Group 1 and Group 2 elements enriched more at TAD boundaries and enhancer–promoter regions (Fig. 5g). Notably, we identified a substantial fraction of impactful *cis*-elements enriched in open chromatin, but not bound by CTCF (Group 3). Group 3 elements show a positive correlation between their impact scores and ATAC–seq signal intensity, and are highly enriched in promoter and enhancer regions, suggesting the presence of enhancer–promoter or promoter–promoter interactions (Fig. 5f)^30^. We also found a small set of elements that are not related to CTCF or ATAC–seq signals (Group 4, Fig. 5e–g), possibly indicating alternative mechanisms which shape local chromatin organization.

We sought to test whether additional factors could be enriched in the impactful elements for chromatin organization. Recently, Myc-associated zinc-finger protein (MAZ) has been shown to colocalize with CTCF, acting as a potential architectural protein to organize chromatin structure^44,45^. To test this observation, we performed a similar enrichment analysis of MAZ ChIP–seq profile across the four groups of impactful elements (Fig. 5f). We found that MAZ is enriched in open chromatin regions, regardless of CTCF binding (Group 1 and 3, Fig. 5e,f). This observation indicates that MAZ may organize chromatin interactions in active enhancer–promoter regions independently from CTCF.

### ISGS identifies T-ALL-specific chromatin organization

**Extended Data Fig. 10 |:**
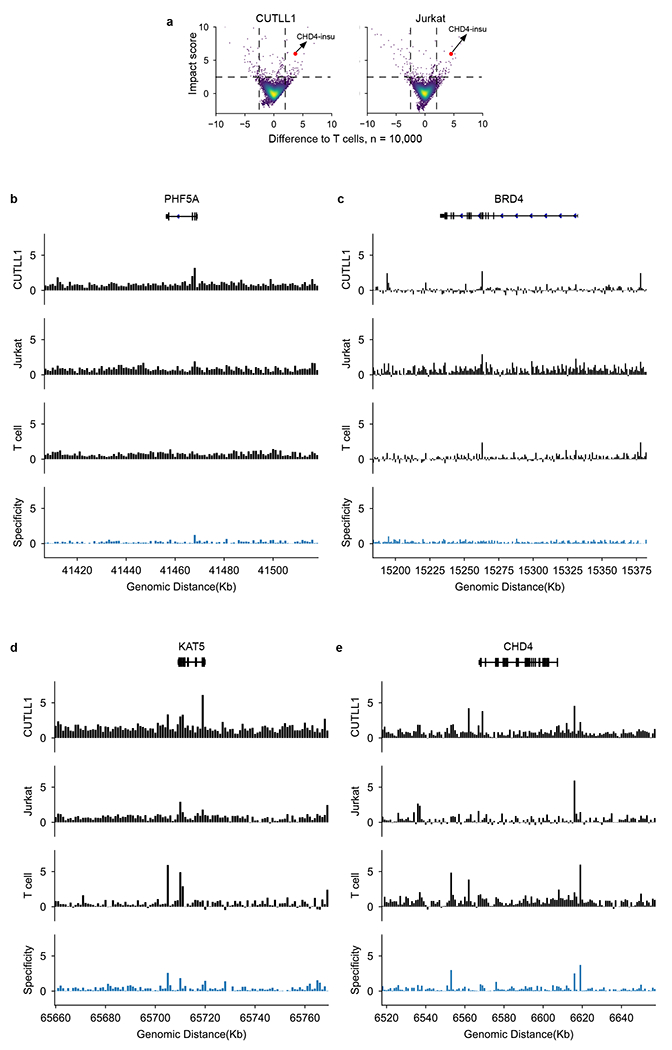
ISGS-identified impact scores at four chromatin remodeler genes in both T-ALL cells and T cells. **a**, Scatter plots showing impact scores of samples of screened regions (n = 10000). The impact score difference between target cell type (CUTLL1 and Jurkat) and T cell are shown on the x axis, and the higher impact scores between the corresponding cell type and the T cells are shown on y axis. **b**–**e**, Impact scores of the DNA elements in T-ALL cells and normal T cells were first calculated independently through ISGS and then visualized at the four chromatin remodeler genes (*PHF5A* (**b**), *BRD4* (**c**), *KAT5* (**d**) and *CHD4* (**e**), with 50Kb upstream and 50Kb downstream) which are required for Jurkat and CUTLL1 cell proliferation according to the CRISPR screening experiments. The specificity track (fourth track) was calculated as the difference between T cell impact score and T-ALL impact score (from CUTLL1 or Jurkat, whichever is smaller). *CHD4* has the highest specificity score between T-ALL cells and normal T cells.

**Fig. 6 |:**
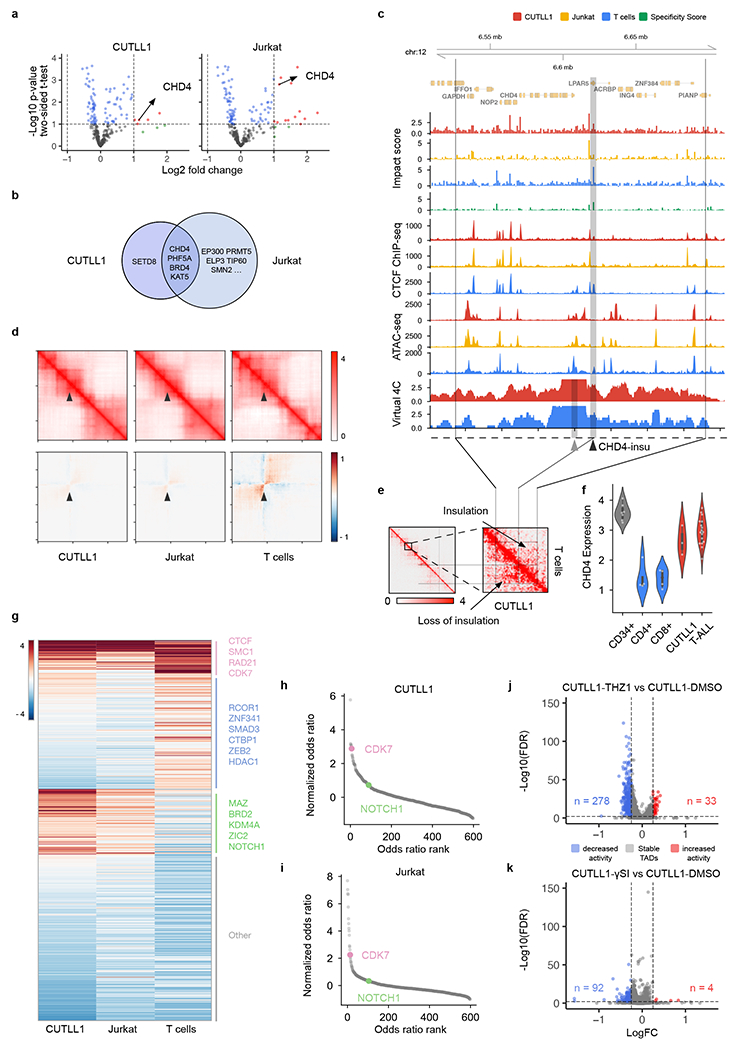
C.Origami-based ISGS reveals cell-type-specific *cis*-elements and *trans*-acting regulators of chromatin folding. **a**, Volcano plot of pooled CRISPR screening results on chromatin remodeling genes in CUTLL1 (left) and Jurkat (right) cell lines. The log_2_fc values indicate the normalized gRNA abundance in Day 4 versus Day 20 posttransfection, reflecting cell proliferation rate upon CRISPR targeting. **b**, Overlap between CRISPR screening-identified chromatin remodeling genes from CUTLL1 and Jurkat cells (**a**). **c**, Genomic tracks around the *CHD4* locus (170 kb). Presented tracks include impact scores, CTCF ChIP–seq and ATAC–seq profiles, and virtual 4C signal using *CHD4* promoter as viewing point (gray arrowhead). Black arrowhead indicates the T cell-specific *CHD4-insu* element. **d**, C.Origami prediction of interaction matrices at *CHD4* locus (top row) and the interaction changes (bottom row) upon in silico deletion of *CHD4-insu* across cell types. Black arrowhead points to the *CHD4-insu* element. **e**, Experimental Hi-C matrices of CUTLL1 and T cells at the *CHD4* locus. **f**, Violin plot of *CHD4* expression in RNA sequencing. **g**, A heatmap of normalized enrichment odds ratios of *trans*-acting regulators across cell types. Representative factors are listed next to the major categories. **h**,**i**, Elbow plots of ISGS-identified *trans*-acting regulators in CUTLL1 (**h**) and Jurkat (**i**) cells. CDK7 and NOTCH1 are highlighted in both plots.**j**,**k**, Volcano plots showing chromatin organization changes of individual TADs upon pharmacological inhibition of CDK7 (**j**) or NOTCH1 (**k**) in CUTLL1 cells. Each dot represents a TAD (*n* = 3,672).

We then envisioned that the ISGS framework could empower high-throughput discovery of disease-specific chromatin organization. To systematically identify T-ALL-specific *cis*-regulatory elements, we performed ISGS and calculated genome-wide impact scores in CUTLL1, Jurkat and normal naive T cells (Extended Data Fig. 10a). We hypothesized that the dysregulation of local *cis*-regulatory elements around chromatin remodeling factors can lead to their abnormal expression in cancer^46,47^. To connect the ISGS-identified impactful *cis*-elements with chromatin remodeling genes in T-ALL, we also performed a pooled CRISPR knock-out screen targeting chromatin remodeling factors in CUTLL1 and Jurkat cells. This screen identified a set of genes, including *CHD4*, *PHF5A*, *BRD4* and *KAT5*, as top hits relevant for T-ALL cell proliferation (Fig. 6a,b). By associating ISGS-identified impactful elements with these four genes (Extended Data Fig. 10b–e), we found that an insulator element upstream of *CHD4,* henceforth termed *CHD4-insu,* has a high impact score in T cells but low in T-ALL (Fig. 6c, black arrowhead).

CHD4 is the helicase component of NuRD complex, which functions to deacetylate H3K27ac (ref. 48). Perturbation of *CHD4* causes cell cycle arrest at G0 in childhood acute myeloid leukemia cells, indicating therapeutic potential^49^. We observed a loss of CTCF binding at the *CHD4-insu* element in T-ALL cells (Fig. 6c). Consistently, in silico deletion of *CHD4-insu* followed by C.Origami prediction in T cells led to a gain of chromatin interactions between the flanking regions compared with T-ALL cells (Fig. 6d).

To test the hypothesis that loss of CTCF binding at *CHD4-insu* leads to insulation loss in T-ALL, we compared the experimental Hi-C contact matrix and its derived virtual 4C signal in CUTLL1 and T cells. We found that, compared with T cells, CUTLL1 cells have stronger chromatin interactions between the flanking regions of *CHD4-insu,* indicating increased interactions between *CHD4* promoter and upstream *cis*-regulatory elements in T-ALL cells (Fig. 6d, virtual 4C tracks, and Fig. 6e). RNA sequencing experiments showed that *CHD4* expression is significantly up regulated in CUTLL1 cells and T-ALL patient samples (Fig. 6f). These results indicate that loss of insulation at *CHD4-insu* in T-ALL cells may have increased *CHD4* expression through establishing new chromatin interactions between *CHD4-insu* flanking regions, consequently promoting leukemia cell proliferation.

### Genome-wide ISGS uncovers *trans*-factors regulating chromatin folding

We next aimed to leverage C.Origami-enabled ISGS to identify cell-type-specific *trans*-acting regulators determining chromatin organization. To do so, we first conducted 1-kb-resolution ISGS to identify cell-type-specific impactful elements. High-impact elements were then aggregated and tested for enrichment in transcription factor binding profiles from ReMap database (Methods)^50^.

Applying this framework to the two T-ALL cell lines and normal T cells, we identified a compendium of cell-type-specific transcription factors contributing to genome organization (Fig. 6g and Supplementary Fig. 19). Notably, our analysis consistently identified known chromatin organization regulators, such as CTCF, RAD21 and SMC1/SMC3, as top candidates across cell types (Fig. 6g). In addition, we found differential sets of *trans*-acting regulators enriched in T cells and T-ALL cell lines, respectively. Several known factors critical for T cell function, such as RCOR1, SMAD3 and ZEB2, are enriched in the T cell-specific group of *trans*-acting factors (Fig. 6g). Consistently, CUTLL1 and Jurkat cells enriched similar groups of factors, represented by MAZ, BRD2 and NOTCH1 (Fig. 6g).

Previously, we found that both CDK7 and NOTCH1 regulate enhancer–promoter interactions in T-ALL cells^36^. Pharmacological inhibition of NOTCH1 (+γSI) leads to H3K27ac alterations in a subset of NOTCH1-associated chromatin interactions, while inhibiting CDK7 (+THZ1) leads to widespread H3K27ac changes^36^. To test the hypothesis that pharmacological inhibition of CDK7 leads to broader chromatin organization changes in T-ALL, we systematically assessed the relative contribution of *trans*-acting factors and found that CDK7 was ranked among the top factors in regulating chromatin organization, whereas the predicted contribution of NOTCH1 was ranked much lower (Fig. 6h,i). In addition, we found that pharmacological inhibition of CDK7 indeed leads to more TADs with chromatin organization changes than the effect from inhibiting NOTCH1 in CUTLL1 cells (Fig. 6j,k). Furthermore, ISGS-identified impactful elements are more enriched in the changed TADs compared with stable TADs upon CDK7 inhibition (Supplementary Fig. 20).

## Discussion

In this study, we developed a multimodal deep neural network architecture, C.Origami, that incorporates both DNA sequence and genomic features for de novo prediction of cell-type-specific genome organization (Fig. 1). We found that DNA sequence information together with CTCF-binding and ATAC–seq signals are sufficient for accurate de novo prediction, comparable to high-quality Hi-C experiments (Figs. 2 and 3). C.Origami was able to learn the general rules governing chromatin organization from one cell type and extrapolate prediction to unseen cell types, including those from different mammalian species. The high performance and minimal requirements on input data make C.Origami generally applicable for studies requiring de novo analysis of chromatin organization without performing chromatin conformation capture experiments (Fig. 4). Additionally, C.Origami can be useful in fields such as cancer genomics involving frequent genome rearrangements and synthetic regulatory genomics with de novo regulatory circuit construction^33,34,51,52^.

With accurate prediction of chromatin organization, our model enables in silico genetic perturbation as a tool to study how *cis*-elements determine 3D chromatin organization in a cell-type-specific manner. C.Origami can simulate the changes in chromatin organization upon in silico genetic perturbation within seconds, providing a highly efficient way to infer potentially causal relationships. Expanding the throughput of in silico genetic perturbations, the ISGS framework can be used for identifying critical DNA elements determining 3D chromatin organization (Fig. 5). While multiple previous methods, such as Expecto^53^, BPNet^54^ and Enformer^55^, have been developed to identify functional *cis*-regulatory elements, they do not identify elements related to cell-type-specific chromatin interactions.

Exploiting the power of ISGS, we identified cell-type-specific impactful *cis*-elements and *trans*-regulators between T-ALL cells and normal T cells. We found a loss-of-insulation event upstream of *CHD4* which might induce new chromatin interactions between the *CHD4* promoter and upstream regulatory elements, correlating with changes in gene expression levels in T-ALL cells (Fig. 6). The discovery of a T-ALL-specific *CHD4* gene expression regulation hints at a potential anti-leukemia target by perturbing the *CHD4-insu* element. Moreover, integrating ISGS results with transcription factor binding databases, we compiled the compendium of potential *trans*-acting regulators determining the chromatin organization in a cell-type-specific manner. As the numbers of publicly available CTCF ChIP–seq and ATAC–seq datasets expand into new cell types, we expect the model to be widely applicable in studies of cell-type-specific chromatin structure and *trans*-acting regulators. Application of in silico screening across normal and disease conditions may lead to the identification of novel targets for therapeutics.

By integrating DNA sequence and cell-type-specific genomic profiles, C.Origami can predict complex genomic features and enable in silico genetic screens. We expect that the underlying multimodal architecture, Origami, is generalizable for applications across a broader range of genomic features, such as epigenetic modifications and gene expression. We envision future genomics studies to shift towards using tools that leverage high-capacity machine learning models such as Origami to perform in silico experiments for discovering cell-type-specific genomic regulation mechanisms.

### Online content

Any methods, additional references, Nature Portfolio reporting summaries, source data, extended data, supplementary information, acknowledgements, peer review information; details of author contributions and competing interests; and statements of data and code availability are available at https://doi.org/10.1038/s41587-022-01612-8.

## Methods

### Hi-C data and processing

We used seven human and mouse Hi-C profiles in this study: IMR-90, GM12878, H1-hESC, K562, CUTLL1, T cell, Mouse Patski (Supplementary Table 1). All of the data are available on GEO (www.ncbi.nlm.nih.gov/geo) and/or the 4D Nucleome Data Portal (https://data.4dnucleome.org). To minimize bias in Hi-C data preprocessing, we obtained counts data in raw fastq format. The reads from human cell lines were aligned to GRCh38 human reference genome and mouse cell lines were aligned to mm10 mouse genome. The alignments were filtered at 10-kb resolution and iteratively corrected with HiC-bench^56^. To ensure the compatibility of the prediction result with downstream analytical tools, we only used a reversible natural log transform to process the Hi-C prediction targets. Prediction from C.Origami with exponential transformation can be directly used as Hi-C chromatin contact matrix data for any downstream analysis.

### CTCF ChIP–seq and ATAC–seq data

CTCF ChIP–seq and ATAC–seq data for most cell types are publicly available online from GEO (www.ncbi.nlm.nih.gov/geo) and the ENCODE data portal (www.encodeproject.org/). CUTLL1 ATAC–seq was sequenced according to a standard method^57^. ATAC–seq libraries were generated from 0.5 × 10^6^CUTLL1 cells. Libraries were sequenced on an Illumina NovaSeq using 100-bp paired-end reads. Details on accession number are listed in Supplementary Table 2. To maintain signal consistency across different cell lines, we aggregated fastq data from different replicates and subsampled them down to 40 million reads. The reads were processed by Seq-N-Slide to generate bigWig files (https://doi.org/10.5281/zenodo.6308846). The bigWig files were used as regular, dense inputs to our model. To prepare an alternative sparse input format, we used MACS2 to perform peak calling on the intermediate bam files to obtain sparse peaks for CTCF and ATAC–seq^58^. The sparse narrowPeak file was converted back to bigWig with ucscutils. We performed a log(*x* + 1) transformation on both dense and sparse bigWig files and used them as inputs to the model.

### DNA sequence

We used the reference genome sequence (hg38 and mm10) from the UCSC Genome Browser database. The original fasta file includes four types of nucleotides and ‘n’ for unknown type. We retained the ‘n’ category and encoded it as the unknown fifth ‘nucleotide’. After encoding, each nucleotide is a five-channel one-hot vector representing ‘ATCGN’, respectively. The same reference genome sequence was used for all cell types.

### Training data

The training data consist of DNA sequence, CTCF-binding signal, ATAC–seq signal and Hi-C matrix from the IMR-90 cell line. The input data to the model include DNA sequence, CTCF ChIP–seq signal and ATAC–seq signal at a 2,097,152-bp region. The output target is the Hi-C matrix at the corresponding region. The Hi-C matrix was originally called at 10-kb resolution and downscaled to 8,192 bp to match the model output resolution. To generate batches of training data, we defined 2-Mb sliding windows across the genome with 40-kb steps. Windows that have overlap with telomere or centromere regions were removed. We randomly split the genome into training, validation and test chromosomes. Chromosomes 10 and 15 were used as the validation set and the test set, respectively. The rest of the chromosomes were used as the training set.

### Model architecture

C.Origami is implemented with the PyTorch framework. The model consists of two 1D convolutional encoders, a transformer module and a task-specific 2D convolutional decoder. The sequence and genomic feature encoder has five and two input channels, respectively. To reduce memory consumption, encoders start with a 1D convolution header with stride 2. To reduce the input length from 2 Mb down to 256 bins, we deployed 12 convolution modules, each of which consists of a residual block and a scaling block. The residual block has two sets of convolution layers with kernel width 5 and the same padding. Batch normalization and ReLU nonlinearity follow each convolutional layer, and the start and end positions of the residual block are connected by a residual connection. The residual blocks do not alter dimension of inputs. The residual connections within the residual block help promote information propagation. The scaling block consists of a 1D convolutional layer with kernel size 5 and stride 2 followed by batch normalization and ReLU activation. The scaling block reduces input length by a factor of 2 and increases the number of hidden layers. We increase the hidden size according to this configuration: 32, 32, 32, 32, 64, 64, 128, 128, 128, 128, 256, 256. The output from the last scaling module has a length of 256 with 256 channels.

The transformer module is built with eight customized attention layers similar to a BERT model^59^. Specifically, we set the number of hidden layers to 256 and ReLU as the activation function, and used eight attention heads. We used relative key query as positional embedding and set the maximum length to be 256. After the transformer module, the model concatenates each position in the 256 bins to every other position to form a 256-by-256 interaction map. The concatenation function takes the 256-bin sequence from the feature extraction module and outputs a 256-by-256 grid where location (*i,j*) is a concatenation of the features at *i* and *j* positions. Since each bin has 256 channels, the concatenation produces a 512-channel 256-by-256 3D tensor.

The decoder consists of five dilated residual networks. We designed the dilation at the corresponding layer to be 2, 4, 8, 16, 32 so that the receptive field of each pixel at the last layer covers the input space, reinforcing interactions between different elements. At the end of the decoder, we use a Conv2D layer with 1 × 1 kernel to combine 256 channels down to one channel, and the output is a 256 × 256 matrix with one channel. The 256 × 256 output from the model was compared with the experimental Hi-C map (ground truth) via an MSE loss. The loss was back propagated through the whole network for gradient updates.

### Model training and prediction

To train the model, we used a training batch size of 8 and Adam optimizer with a learning rate of 0.002. A cosine learning rate scheduler with 200-epoch period was used for stabilizing training. We used three types of data augmentations. First, we selected the 2-Mb window with random shifts within 0.36-Mb range. Second, we reverse-complemented the sequence and flipped the target Hi-C matrix with 0.5 probability. Third, we added Gaussian noise to all input signals with zero mean and 0.1 standard deviation. The model achieved minimal validation loss when trained for 54 epochs. The model training time was 18 h on a GPU cluster with quad NVIDIA Tesla V100 GPUs, 320 GB of RAM and 10 CPU cores. Model inference with a mobile NVIDIA RTX 2060 GPU can be achieved in under 1 s, and 3 s on a mobile Intel i7-8750H CPU. To run prediction in IMR-90, the reference DNA sequence, CTCF ChIP–seq and ATAC–seq from IMR-90 in a 2-Mb region are taken as input. For de novo prediction in a target cell type, we replaced IMR-90 CTCF ChIP–seq and ATAC–seq with the corresponding CTCF and ATAC–seq from the specific target while keeping the same reference sequence.

### Insulation score

Insulation score is implemented as the ratio of maximum left and right region average intensity and the middle region intensity^56^. We also added a pseudocount calculated from chromosome-wide average intensity to prevent division by zero in unmappable regions. Given that all the regions contain *n* interactions, the insulation score can be formulated as follows:

$$\text{Insulation}=\frac{\max\left(\frac{1}{n}\sum_{n}\left(\text{Left Intensity}\right),\frac{1}{n}\sum_{n}\left(\text{Right Intensity}\right)\right)+\text{pseudocount}}{\frac{1}{n}\sum_{n}\left(\text{Center Intensity}\right)+\text{pseudocount}}$$

where pseudocount is set to the average intensity of one chromosome within 2 Mb.

### Loop calling

We used the Hi-C valid pairs with the FitHiC software^60,61^ to identify significant interactions. We used a resolution of 10 kb, and minimum and maximum distances of 30 kb and 1 Mb. For loop calling on predicted matrices, we converted the predicted matrix back to valid pairs by merging predictions to chromosomes and counting the discretized intensity value. FitHiC generated a list of significant interactions with corresponding false discovery rate (FDR)-corrected *Q* values using global background as reference. For loop analysis on IMR-90, we computed AUROC and overlap between loops called from experimental Hi-C and loops called from predicted Hi-C. To calculate AUROC, we used predicted loops as target. *Q* value cutoffs ranging from 1 × 10^−5^ to 1 × 10^−13^ are selected to filter significant loops called from the predicted Hi-C. Then, the *Q* values from loops called from experimental Hi-C were compared with significant loops called from prediction to calculate an AUROC. For overlap analysis, we chose a fixed 1 × 10^−5^ cutoff for loops called from predicted and experimental Hi-C and compared the overlap of significant loops. For loop analysis on specific types of interaction, we overlapped the two anchors of each loop and obtained the categories for each loop called. The loops were then filtered by different categories and the same AUROC and overlap analysis was performed on each category of loops.

For cell-type-specific loop analysis between IMR-90 and GM12878, we first used a more stringent cutoff of 1 × 10^−7^ as a threshold for significant loops. Then we further categorized specific loops into IMR-90 specific or GM12878 specific according to the log_2_fc of loop interaction counts. To calculate AUROC, we used log_2_fc in place of the *Q* value cutoff from previous analysis. We compared two log_2_fc values. The first log_2_fc is between predicted loops in cell type 1 and predicted loops in cell type 2 (for example, IMR-90 predicted loop/GM12878 predicted loop). The second log_2_fc is between experimental loops in cell type 1 and predicted loops in cell type 2 (for example, IMR-90 experimental loop/GM12878 predicted loop). Then the same AUROC and overlap analysis was performed for each of the two cell-type-specific groups. For loop analysis on a specific type of interaction in a cell-type-specific way, the same anchor overlap was performed with corresponding AUROC and overlap analysis.

### Chromosome-scale Hi-C contact matrix prediction

To bridge adjacent 2-Mb-window predictions into chromosome-wide Hi-C contact matrices, we ran the prediction in a sliding window with 262,144-bp step size, which is 1/8 of the 2-Mb prediction window. All predictions were in-painted to their corresponding location on the contact map with multiple overlaps. To correct for different levels of overlap, we counted the total times of overlap for every pixel and divided by the number of overlaps. The resulting chromosome-wide prediction can be directly used for downstream analysis such as TAD calling, loop calling and insulation score calculation.

### Distance-stratified intensity and correlation

Distance-stratified intensity and correlation calculations were based on fused chromosome prediction. Stratified intensity at distance *i* was calculated by aggregating the line that is parallel to the Hi-C diagonal with offset of *i.* Stratified correlation was calculated as Pearson’s *r* between the shifted diagonal line of prediction and ground truth.

### Performance comparison with previous methods

We compared the performance of C.Origami against three previously published methods: Akita^18^, DeepC^19^ and Orca^20^. We compared the performance using four metrics: insulation score correlation, observed versus expected Hi-C metrices correlation, MSE and distance-stratified correlation. We calculated the four metrics separately for the four models by comparing their prediction to the experimental Hi-C data. The comparison was carried out in two different cell types: (1) the training cell type, IMR-90 cells, which most models were trained on and (2) a new cell type, GM12878 cells, aiming to quantify the performance of de novo prediction of chromatin organization of the four models.

We generated a set of sliding windows that covers the whole genome and can be predicted by each model. Since Akita and DeepC are only able to predict interaction within a 1-Mb window, we restricted the test regions to 1-Mb blocks. To generate a genome-wide testing dataset, we selected all 1-Mb regions in a sliding window with 0.5-Mb overlap between neighboring regions. To ensure compatibility with all models’ prediction windows, the sliding window starts and ends 1.5 Mb after chromosome starting location and before ending location to create buffer regions for models requiring 2-Mb windows as inputs. In total, 5,935 regions were generated genome-wide. We used all four models to predict the interaction for the corresponding regions.

The most relevant versions of the previous models were selected for comparison. For Akita, the IMR-90 output channel was selected. For DeepC, we used their model trained with IMR-90 data. Orca was only trained on two cell types, human foreskin fibroblasts (HFFs) and H1-hESCs. We used the HFF model because HFF is also a fibroblast cell line similar to IMR-90. The comparison turned out to be valid because even though Orca was trained on HFF, it outperformed both Akita and DeepC on IMR-90 in many benchmarks. For C.Origami, we used the IMR-90-trained model.

It is necessary to perform scaling and normalization to each model’s outputs due to their varied prediction target customizations. Akita predicts a 1,048,576-bp region with 512 bins. We removed the extra 48,576 bp on the sides to make the prediction 1 Mb, followed by rescaling into 128 bins. Orca can predict interactions at multiple scales. Since C.Origami used a 2-Mb window as prediction target, we selected the 2-Mb window in Orca for consistency. The prediction was then cropped to 1 Mb and rescaled to 128-by-128. For C.Origami, the prediction is a 2,097,152-bp window. We cropped the prediction to leave the center 1-Mb regions and rescaled to 128 bins.

DeepC’s prediction target is different from other models, 45-degree rotations. DeepC also produces predicted Hi-C maps in different scales compared with other methods. Thus, we performed a series of transformations (Supplementary Fig. 11) including mirroring, rotating and cropping to make a comparable contact matrix to outputs produced by other models. We used a 1-Mb prediction window for DeepC and rescaled the output to 128-by-128.

The first step to make the models comparable is selecting a common ground truth Hi-C as the evaluation target. Since each model used a different ground truth with different transformations (for example, observed/expected, log, gaussian smoothing), they cannot be compared directly. We defined the evaluation target as logged Hi-C intensity with iterative correction and eigenvector decomposition (ICE): (log(ICE normalized counts +1)). Logged intensity has a few advantages over observed versus expected map. First, it allows for computing insulation scores. Second, it can be converted to observed versus expected while the reverse is not straightforward. It can also be converted to raw counts by taking the exponent. Third, it is used as the default Hi-C format for most downstream analysis pipelines such as loop calling and visualization.

The second step to make the models comparable is to normalize model outputs to the evaluation Hi-C target. Since each model used a different original prediction target, the intensities of prediction and evaluation target show a large discrepancy depending on the model. Specifically, DeepC results stood out with a unique pattern which might be a result of their custom stratified binning method (Supplementary Fig. 12). We also observed that the raw predicted matrix intensities were too different to compare (Supplementary Fig. 12).

We performed distance-stratified normalization to align all predictions to the target (Supplementary Fig. 13). We computed the mean and s.d. for each diagonal and then normalized the prediction to target experimental Hi-C. Formally, let $\hat{T}$ be the normalized matrix, *T* be the target ground truth matrix and *M* be the unnormalized matrix. Let *m_d,i_* be the corresponding element in *M*, and *μ* and *σ* denote the mean and s.d. at diagonal *d* in matrix *T* and *M*. Then, every *i*^th^ entry on *d*^th^ diagonal, *t*_*d,i*_ can be normalized as follows:

$$\forall t_{d,i}\in \hat{T},t_{d,i}=\frac{\sigma_{d}^{T}}{\sigma_{d}^{M}}\left(m_{d,i}-\mu_{d}^{M}\right)+\mu_{d}^{T}$$

The normalized predictions were compared with the target Hi-C using the four metrics. Each metric was calculated per chromosome for every tested model using their corresponding prediction and the experimental data as ground truth.

We also performed GM12878 de novo prediction comparison. For C.Origami, we used the same IMR-90-trained model but GM12878 CTCF ChIP–seq and ATAC–seq profiles as inputs to predict Hi-C. For sequence-only models, we used the same DNA sequence setup because they could not provide cell-type-specific de novo prediction. Though ideally input DNA sequence should be cell-type-specific, such a procedure is not realistic for general applications.

### De novo prediction evaluation

Regions with normal intensity (>10% intensity quantile) and low similarity (<20% insulation difference) between the experimental Hi-C matrices of the two analyzed cell types were selected as structurally different genomic regions. In total, ~15% of the entire genome (~450 Mb) was included for evaluating the performance of cell-type-specific Hi-C prediction in each pair of cell types. In comparison, structurally conserved genomic regions were characterized by normal intensity (>10% intensity quantile) and high similarity (>20% insulation difference). These regions were used for control analysis in parallel with the aforementioned evaluation in the structurally differential genomic regions.

### C.Origami prediction at the CUTLL1 t(7;9) translocation site

To generate experimental Hi-C data, we defined a custom chromosome in HiC-bench^56^. The custom genome in HiC-bench is defined at the matrix-filtered step where the pipeline assigns reads to chromosomes. For the CUTLL1 experiment, we defined a custom chromosome chr7chr9, with chr7:0-142800000 as the starting chromosome and chr9:136500000-138394717 as the ending chromosome. CUTLL1 t(7;9) translocation is heterozygous, leading to allele-specific complexity to its corresponding Hi-C matrix. Since only one allele is translocated, the experimental Hi-C data mapped to either the normal reference genome or the t(7;9) translocated reference genome would be a mixture of chromatin interactions from both translocated and normal chromosomes. To align with this hybrid effect of Hi-C contact map, we first separately predicted three sets of Hi-C maps: t(7;9) translocated chromosome, normal chromosome 7 and normal chromosome 9. The predicted Hi-C matrix at the t(7;9) locus is an average of the predicted Hi-C maps of t(7;9) translocation chromosome and a fused prediction map ranging from normal chr7 to the breakpoint chr7:142,797,952 and extending from chr9:136,502,817 to the rest of normal chr9. We did not count the interchromosomal interactions at these loci due to their much weaker intensity compared with the intrachromosomal interaction at the translocation site.

### Mouse prediction

For the mouse Patski cell-type prediction^42^, the CTCF ChIP–seq and ATAC–seq inputs were processed using the same pipeline with mm10 as the assembly number. The original C.Origami model trained with IMR-90 dense input features was used for prediction. For genome-wide evaluation of predicting mouse chromatin organization, we adopted the same procedure from the ’Performance comparison with previous methods’ section.

### CTCF depletion prediction in mouse embryonic stem cells (mESCs)

We preprocessed CTCF ChIP–seq and Hi-C on mESCs from Nora et al.^32^, following the same pipeline for ChIP–seq and Hi-C. In total, three sets of data, with conditions: untreated, auxin-induced CTCF depletion and wash-off, are processed. Since this study did not measure ATAC–seq, the C.Origami model was re-trained using only DNA sequence and CTCF ChIP–seq on the untreated condition. The re-trained model was then used for predicting chromatin organization in the CTCF depletion (auxin treatment) and restoration (auxin wash-off) conditions. Genome-wide performance benchmark followed the same procedure from the ‘Performance comparison with previous methods’ section.

### GRAM

The GRAM scoring system is a generalized version of Grad-CAM on 2D outputs^62^. Instead of taking a single output, GRAM operates on a region *r* in the output space *y* and runs backpropagation on all pixels within *r*. GRAM on region *r* in network layer *m* is defined as follows:

$${\text{GRAM}}_{m}\left(r\right)=\underset{k}{\sum^{}}\text{ReLU}\left(\alpha_{k}^{r}\right)\cdot \text{ReLU}\left(A_{k}^{m}\right)$$

where $\alpha_{k}^{r}$ is the activation weight for channel *k* and region *r*. Formally, $\alpha_{k}^{r}$ is defined as:

$$\alpha_{k}^{r}=\frac{1}{Z}\underset{i}{\sum }\underset{j}{\sum }\frac{\partial r}{\partial A_{k_{i,j}}^{m}}$$

where *Z* is the number of activations in the layer and the quotient is the gradient at position *i*,*j* in the activation layer *m* with respect to output *r*. $r.\alpha_{k}^{r}$ can be interpreted as the average gradient across width and height dimensions at the layer *m*. $A_{k}^{m}$ is the activation in channel *k* at layer *m*. In this study, we choose *r* to be the full output space. During forward propagations, activation (*A*^*m*^) at the target layer *m* is recorded. This activation map is a 3D tensor, or an image with *k* channels. Then, the *r* region of the output is selected for backpropagation and gradients are calculated for every layer. The gradients (used for calculating weights $\alpha_{k}^{r}$) at the target layer *m* are collected. The set of collected gradients is also an image-like 3D tens or with *k* channels. To obtain $\alpha_{k}^{r}$, we averaged the gradients across width and height dimensions, resulting in a *k*-dimensional array. The goal of GRAM is to visualize a gradient-weighted activation map that maximizes the output signal. To obtain this weighted activation, $\alpha_{k}^{r}$ is used as weights to average the *k* channels activation image (*A*^*m*^). The final averaged activation is defined as the GRAM output.

### Attention score

In the transformer module, we implemented the vanilla multi-head attention^27^:

$$\text{MultiHead}(Q,K,V)=\text{Concat}\left({\text{head}}_{1},\ldots ,{\text{head}}_{h}\right)W^{o}$$

where *Q, K,* *V* are query, key and values. *W*^o^ is the out projection of dimension (number of heads *h* times value dimension *d*_v_ by model dimension *d*_m_). In our implementation *d*_v_ and *d*_m_are set to 128. *head_i_* is a single attention head and is calculated by:

$${\text{head}}_{i}(Q,K,V)=\text{softmax}\left(\frac{\left(QW_{i}^{\text{Q}}\right){\left(KW_{i}^{\text{K}}\right)}^{T}}{\sqrt{d_{\text{k}}}}\right)VW_{i}^{V}$$

where *W*^Q^, *W*^K^, *W*^V^ are projection weights for query, key and value. *d*_k_ is the embedding dimension of key, also implemented as 128. During forward propagation, we extract attention weights for head *i* which is defined as the alignment between query and key:

$${\text{weights}}_{i}(Q,K)=\text{softmax}\left(\frac{\left(QW_{i}^{\text{Q}}\right){\left(KW_{i}^{\text{K}}\right)}^{T}}{\sqrt{d_{\text{k}}}}\right)$$

The attention score can be calculated by averaging attention weights across different heads:

$$\text{Attention score}(Q,K)=\frac{1}{N}\underset{i}{\sum }{\text{weights}}_{i}$$

where *N* = 8 because each layer has eight attention heads. Since the transformer module consists of eight attention layers, for each prediction, we obtained a set of eight attention scores. The attention score is visualized with the BertViz package^63^.

### Impact score

The impact score in the screening experiment is defined as the pixel-wise mean absolute difference between two predictions. Formally, given that we have a prediction *S*, a 2D contact matrix from the original input and S′ from the input perturbed at location *x*, and let *s_i,j_* be the individual pixel in *S* at position *i* and *j* and *n* be the width/heights of *S*, the impact score of location *x* is defined as:

$$\text{Impact score}(x)=\overset{n}{\underset{i}{\sum }}\overset{n}{\underset{j}{\sum }}\frac{\left|{s\prime }_{i,j}-s_{i,j}\right|}{n^{2}}$$

### In silico genetic screen

Typical ChIP–seq profiles have peak widths ranging from a few hundred base pairs to 1 kb. To capture fine-regulation elements, we performed genome-wide ISGS at 1-kb resolution. The screening starts from individual chromosomes with a window size of 2 Mb. Inside this window, a 1-kb perturbation region centered at the 2-Mb window was deleted followed by padding at the end and C.Origami prediction. For each window, the original input and perturbed input were predicted by C.Origami and collected. Once the output acquisition is completed for the window, the screening moves to a downstream overlapping window that has 1-kb offset from the current window. Since the in silico screening offset is equal to the length of perturbation size, this procedure produces a continuous impact score that covers all genomic regions with a resolution of 1 kb. It is worth noting that screening at 1-kb resolution could be computationally intensive. To reduce computational load, we randomly sampled 10 chromosomes (chr 5, 7, 8, 11, 12, 14, 15, 19, 20, 22) to represent the whole genome and performed 1-kb-resolution screening on the selected chromosomes.

To obtain the most impactful elements from the screening result, we designed a custom peak calling algorithm. We defined the peak score *p* of a locus as the difference between maximum and minimum signal within the range of three bins including the locus. We then selected the top 1% of the total screened regions as a cutoff for impactful elements based on the peak score.

To annotate the in silico genetic screen-identified impactful *cis*-elements, we compiled a set of genomic annotations including TAD boundary regions, enhancers, promoters, intragenic regions and intergenic regions. The boundary region was generated by calling TAD boundaries at 10-kb resolution with HiC-bench^56^, using its TopDom module and connecting adjacent TADs. To increase robustness of TAD boundary calling, we expanded the boundary width to 50 kb. The promoter region was defined as a 5.5-kb window, spanning 5 kb upstream and 500 bp downstream of gene transcription start site. Enhancers were defined by the H3K4me1 modification, which marks both active and inactive enhancers^64^. The H3K4me1 ChIP–seq peaks for IMR-90 were downloaded from ENOCDE with accession number ENCFF611UWF (https://www.encodeproject.org/files/ENCFF611UWF). To increase robustness, we expanded peaks to have at least 1-kb width.

### In silico genetic screen at 2-Mb windows

We conducted an in silico genetic screen at a fixed 2-Mb window without centering the deletion element. We systematically removed segments of 8,192 bp, or 1 bin, from model inputs. To scan through the entire 2-Mb region, we performed 256 deletion experiments at each bin and calculated the prediction difference map before and after deletion. To maintain input shape, we appended 8,192 bp of empty input features to the end.

### CRISPR screening for chromatin remodeling genes in T-ALL cell lines

Pooled CRISPR screenings across 313 chromatin remodeling genes in CUTLL1 and Jurkat cells were carried out in parallel with our previous studies for pooled screening of RNA binding protein in T-ALL cells^65^. Briefly, for each chromatin remodeling gene, we designed on average 6–8 single guide RNAs (sgRNAs), for a total of ~2,500 sgRNAs. The sgRNA sequences were synthesized by Twist Bioscience, and cloned into a lentivirus-based sgRNA vector tagged with GFP (Addgene plasmid no. 65656). Cas9-expressing T-ALL cell lines were transduced with sgRNA library virus at a low multiplicity of infection (MOI, ~0.3), followed by infection efficiency assessment through GFP percentage on Day 4 posttransduction. Remaining cells were placed back into culture until 20 day posttransduction.

Cell proliferation was measured by comparing the sgRNA frequencies between Day 4 and Day 20 cells. Genomic DNA was collected from Day 4 and Day 20 cells using Qiagen DNA Purification kit based on the manufacturer’s protocol. The gRNA frequencies in the genomic DNA were amplified and quantified following our previous procedure^65^. For pooled CRISPR screening analysis, samples of each time-point were normalized as sgRNA read count/total read count × 100,000. Subsequently, normalized reads were then used to calculate log_2_fc (as normalized read count Day 4/normalized read count Day 20) for each gRNA. The fold changes between Day 4 and Day 20 for each gene were averaged from all CRISPR gRNA targets. *P* values were calculated via a two-sided *t*-test comparing the fold changes of all gRNA targets of the same gene with fold change of 1.

### Virtual 4C

HiC-bench ‘virtual4C’ pipeline^56^ was used to compute the interactions of each selected viewpoint in a roll-window fashion. We summed the valid read pairs in a 5-kb area centered at 100-bp bins that covered the area of ±2.5 Mb from the viewpoint (50,000 bins per viewpoint). The interactions were normalized by the total number of valid pairs of the sample.

### *Trans*-acting regulator identification in T-ALL cell lines

To connect the differential patterns of *cis*-elements with *trans*-acting regulators, we compared the selected cell-type-specific impactful regions by a custom peak calling method, followed by a transcription factor enrichment test for identifying potential *trans*-acting regulators. We used the transcription factor database from ReMap2022 (ref. 50). To reduce low-quality signals from the ReMap database, we filtered out transcription factor profiles that had less than 7,000 hits, or profiles that only had a single experiment. Together, we collected 612 transcription factor binding profiles for downstream analysis. We used Fisher’s exact test to evaluate the overlap between impactful *cis*-elements from ISGS and each transcription factor from the database. The test was conducted using the LOLA (Locus Overlap Analysis) package^66^. For common transcription factors with hit counts larger than 20,000, we downsampled profiles to 20,000. We calculated the *Q* value with FDR correction based on the 612 transcription factor profiles tested and used odds ratio as the main metric to determine enrichment of each factor in impactful *cis*-elements.

To compare the contributing *trans*-acting regulator profiles between different cell types, we first normalized the odds ratio within each cell type. We performed *k*-means clustering of transcription factors based on their normalized odds ratios in CUTLL1, Jurkat and T cells. The *k*-means clustering was performed with standard Euclidean distance with six centroids. The clusters were further grouped and visualized using a heatmap.

### Intra-TAD activity analysis

Iteratively corrected matrices were re-normalized by dividing each bin value by the sum of all the values in the same distance bin in the same chromosome (distance normalization). All the TADs identified in the control sample were used as the reference TADs to compute the intra-TAD activity changes. The set of reference TADs between the two samples, S1 (control) and S2 (treatment), were denoted as set *T*. A paired two-sided *t*-test was performed on each single interaction bin within each reference TAD between the two samples. We also calculated the difference between the average scores of all interaction intensities within such TADs and the TAD interaction log fold change. Finally, a multiple testing correction is performed by calculating the FDR on the total number of TAD pairs tested. The TAD interaction change for each *t* in recalculated as follows:

$$\text{TAD change}(t)=\frac{\sum_{i}^{l_{t}}S_{2i}}{\left|I_{t}\right|}-\frac{\sum_{i}^{l_{t}}S_{1i}}{\left|I_{t}\right|}$$

We classified the reference TADs in terms of Loss, Gain or Stable intra-TAD changes by using the following thresholds: FDR < 0.01, absolute TAD interaction log fold change > 0.25 and absolute TAD interaction change > 0.1.

### Additional software

Additional software used included the following: software: HiC-bench, Seq-N-slide, MACS2 (v.2.1.1), FitHiC (v.2.0.7); Python packages: Pytorch (v.1.9.0), Numpy (v.1.22.3), pybigwig (v.0.3.18), scikit-image (v.0.19.3); R packages: EnhancedVolcano (v.3.16).

### Reporting summary

Further information on research design is available in the Nature Portfolio Reporting Summary linked to this article.

## Extended Data

## Supplementary Material

Supplementary Figures

Cell-type-specific predictions

## Data Availability

Most of the Hi-C,CTCF ChIP–seq and ATAC–seq datasets used in the study were public data from the ENCODE portal and/or NCBI GEO database, with the accession codes listed in the corresponding Methods section. The generated data (CUTLL1 ATAC–seq) are uploaded to GEO with accession number GSE216430.
