# Supplementary figures and images for: Cell-type-specific prediction of 3D chromatin organization enables high-throughput in silico genetic screening (part 2 of 2)
Source: Nat Biotechnol. 2023 Jan 9;41(8):1140–50. doi: 10.1038/s41587-022-01612-8 (PMC10329734; doi:10.1038/s41587-022-01612-8)

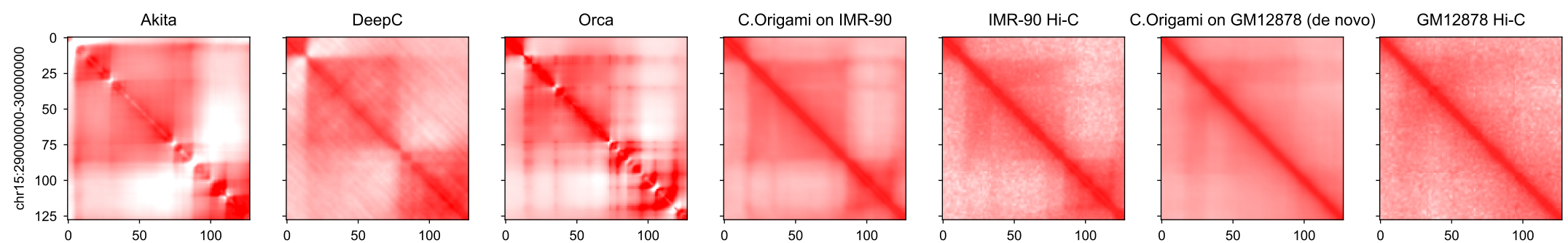

Supplement: Supplementary file 3 — Cell-type-specific predictions. [file 41587_2022_1612_MOESM3_ESM.zip › Cell type-specific predictions/chr15_29500000.pdf]

chr20:46500000-47500000

Akita

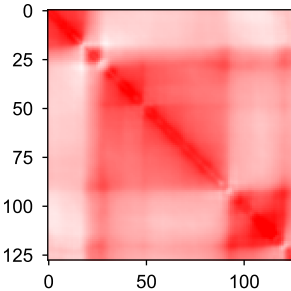

DeepC

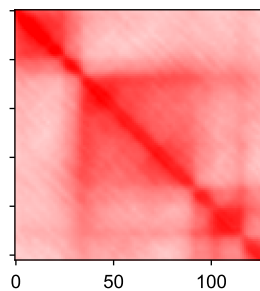

Orca

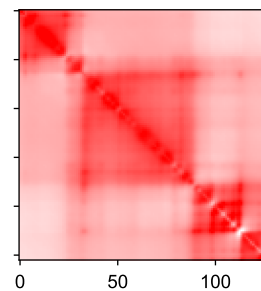

C.Origami on IMR-90

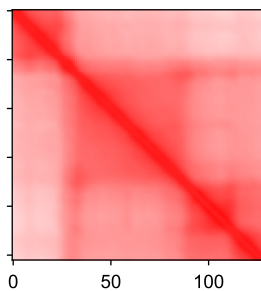

IMR-90 Hi-C

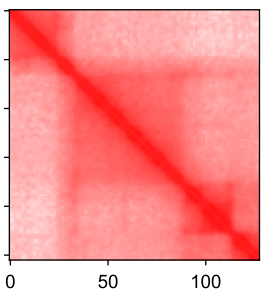

C.Origami on GM12878 (de novo)

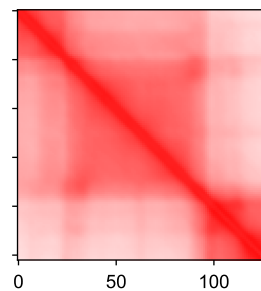

GM12878 Hi-C

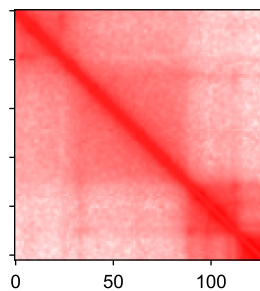

Supplement: Supplementary file 3 — Cell-type-specific predictions. [file 41587_2022_1612_MOESM3_ESM.zip › Cell type-specific predictions/chr20_47000000.pdf]

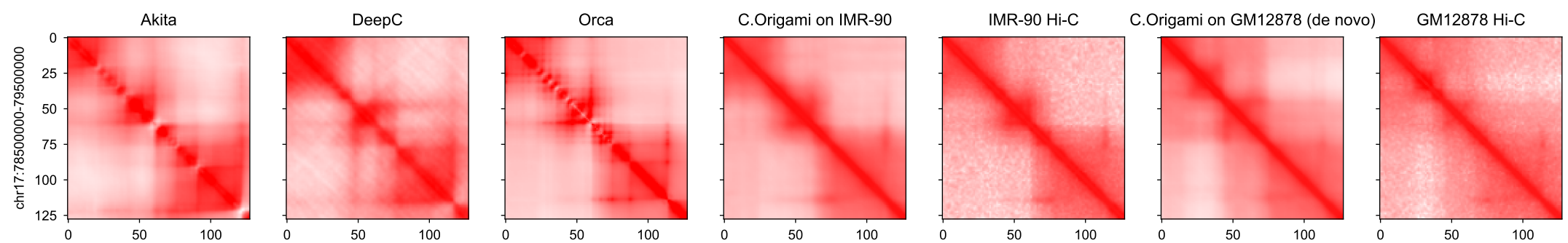

Supplement: Supplementary file 3 — Cell-type-specific predictions. [file 41587_2022_1612_MOESM3_ESM.zip › Cell type-specific predictions/chr17_79000000.pdf]

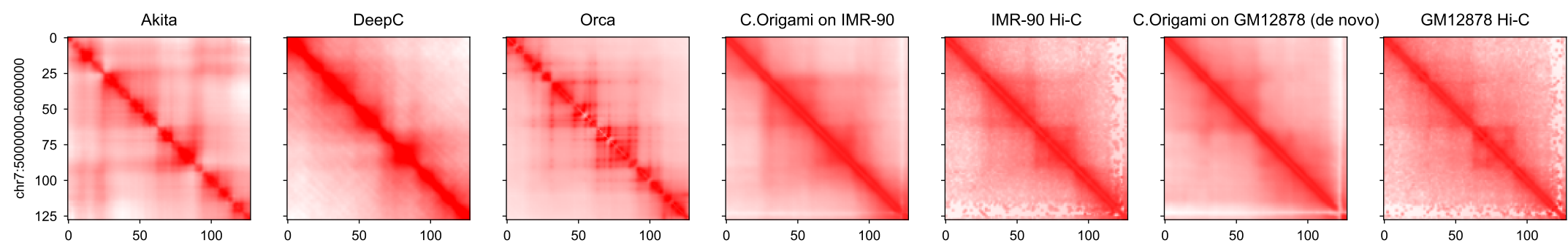

Supplement: Supplementary file 3 — Cell-type-specific predictions. [file 41587_2022_1612_MOESM3_ESM.zip › Cell type-specific predictions/chr7_5500000.pdf]

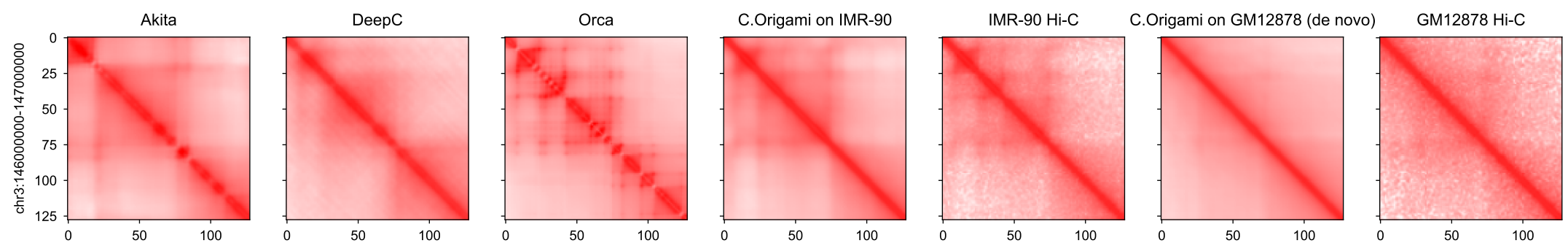

Supplement: Supplementary file 3 — Cell-type-specific predictions. [file 41587_2022_1612_MOESM3_ESM.zip › Cell type-specific predictions/chr3_146500000.pdf]

chr9:12500000-13500000

Akita

DeepC

Orca

C.Origami on IMR-90

IMR-90 Hi-C

C.Origami on GM12878 (de novo)

GM12878 Hi-C

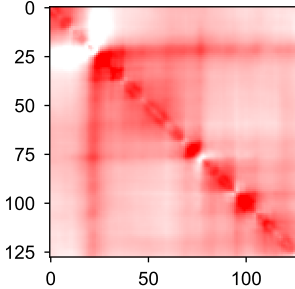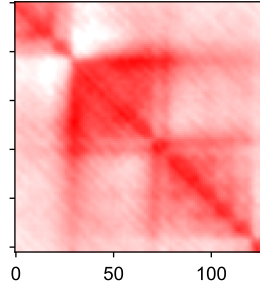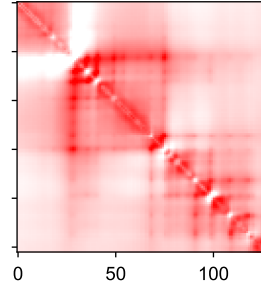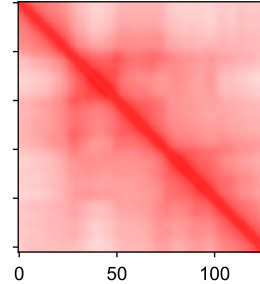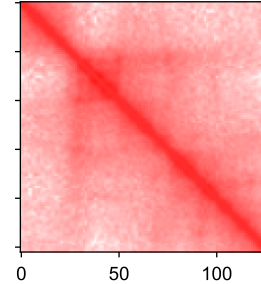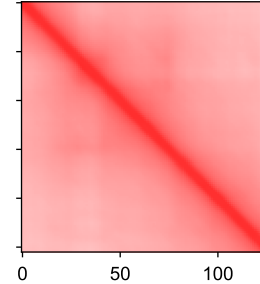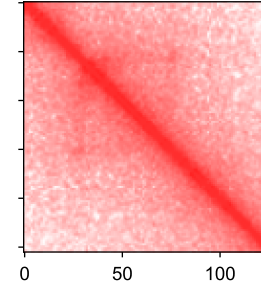

Supplement: Supplementary file 3 — Cell-type-specific predictions. [file 41587_2022_1612_MOESM3_ESM.zip › Cell type-specific predictions/chr9_13000000.pdf]

chrX:26000000-27000000

Akita

DeepC

Orca

C.Origami on IMR-90

IMR-90 Hi-C

C.Origami on GM12878 (de novo)

GM12878 Hi-C

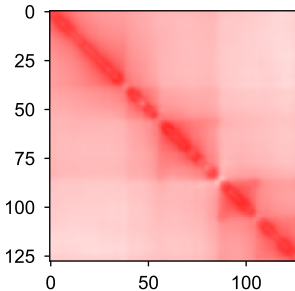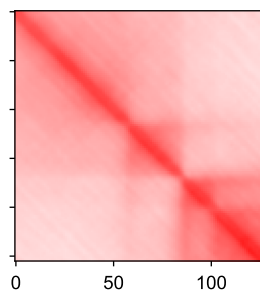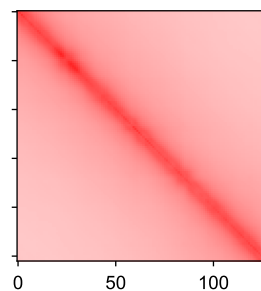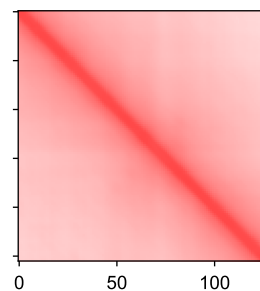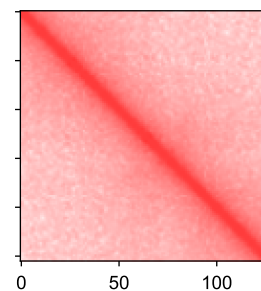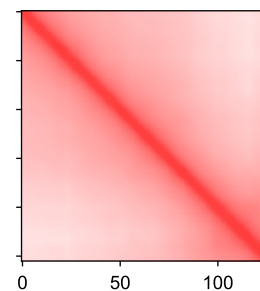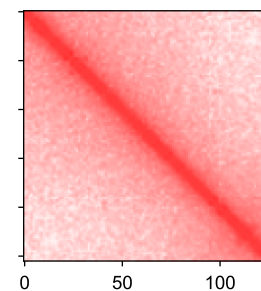

Supplement: Supplementary file 3 — Cell-type-specific predictions. [file 41587_2022_1612_MOESM3_ESM.zip › Cell type-specific predictions/chrX_26500000.pdf]

chr17:16500000-17500000

Akita

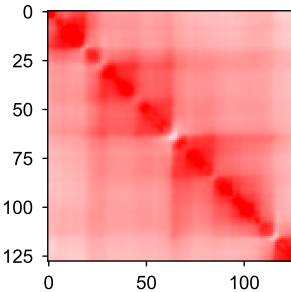

DeepC

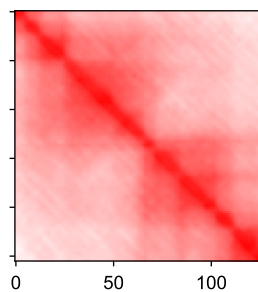

Orca

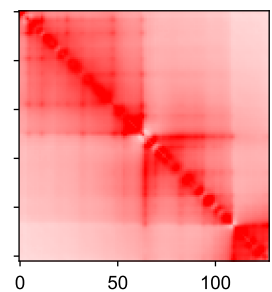

C.Origami on IMR-90

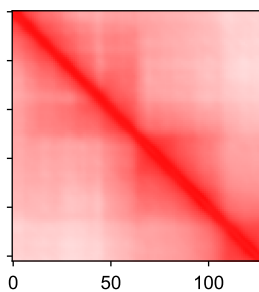

IMR-90 Hi-C

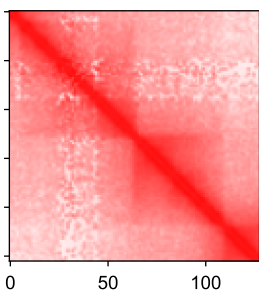

C.Origami on GM12878 (de novo)

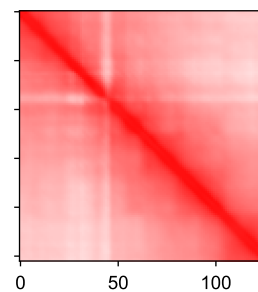

GM12878 Hi-C

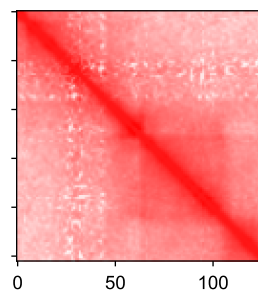

Supplement: Supplementary file 3 — Cell-type-specific predictions. [file 41587_2022_1612_MOESM3_ESM.zip › Cell type-specific predictions/chr17_17000000.pdf]

chr15:47000000-48000000

Akita

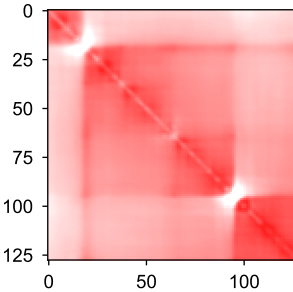

DeepC

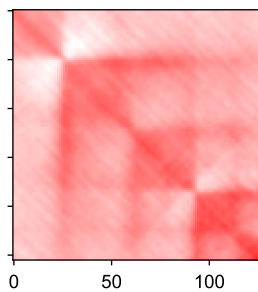

Orca

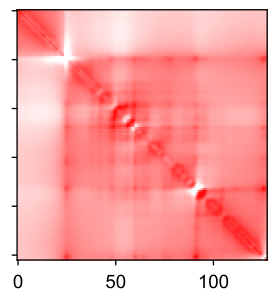

C.Origami on IMR-90

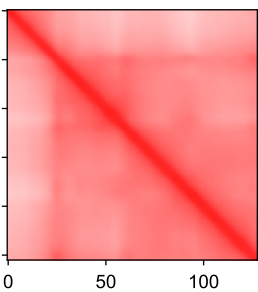

IMR-90 Hi-C

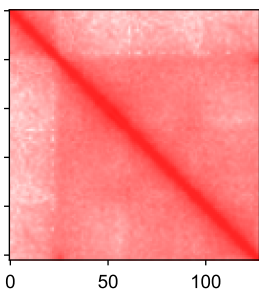

C.Origami on GM12878 (de novo)

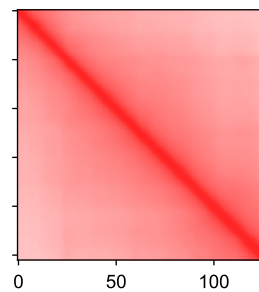

GM12878 Hi-C

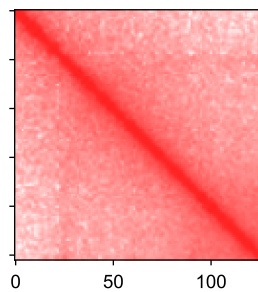

Supplement: Supplementary file 3 — Cell-type-specific predictions. [file 41587_2022_1612_MOESM3_ESM.zip › Cell type-specific predictions/chr15_47500000.pdf]

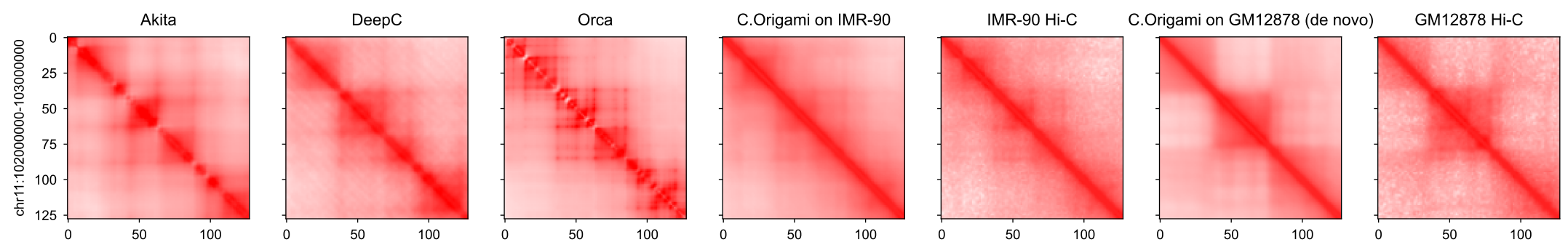

Supplement: Supplementary file 3 — Cell-type-specific predictions. [file 41587_2022_1612_MOESM3_ESM.zip › Cell type-specific predictions/chr11_102500000.pdf]

chr15:79500000-80500000

Akita

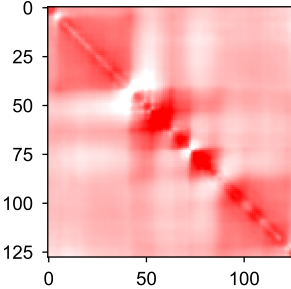

DeepC

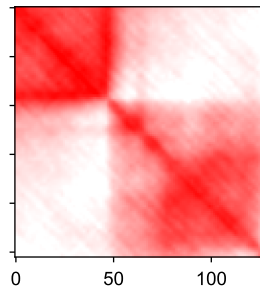

Orca

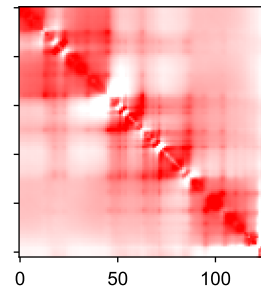

C.Origami on IMR-90

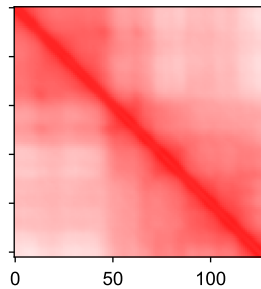

IMR-90 Hi-C

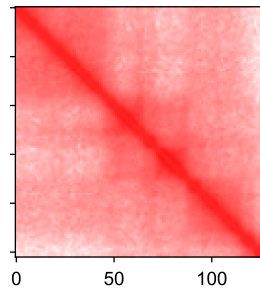

C.Origami on GM12878 (de novo)

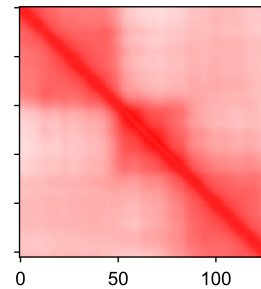

GM12878 Hi-C

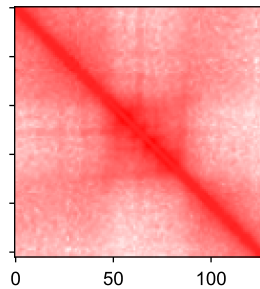

Supplement: Supplementary file 3 — Cell-type-specific predictions. [file 41587_2022_1612_MOESM3_ESM.zip › Cell type-specific predictions/chr15_80000000.pdf]

chr5:98500000-99500000

Akita

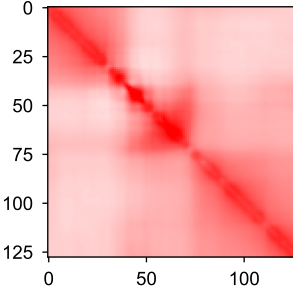

DeepC

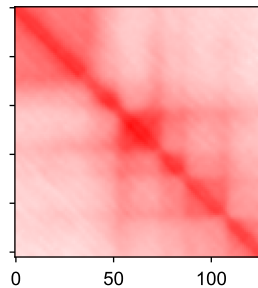

Orca

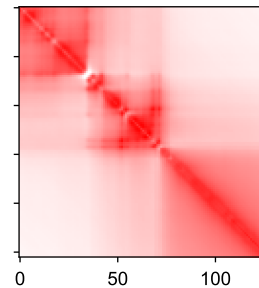

C.Origami on IMR-90

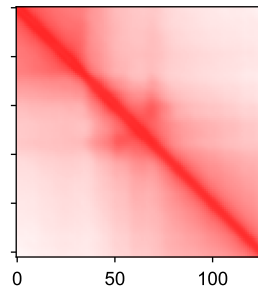

IMR-90 Hi-C

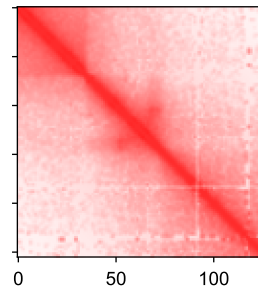

C.Origami on GM12878 (de novo)

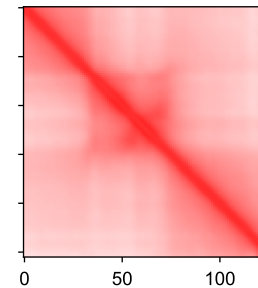

GM12878 Hi-C

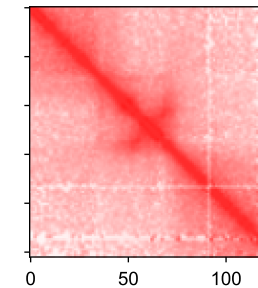

Supplement: Supplementary file 3 — Cell-type-specific predictions. [file 41587_2022_1612_MOESM3_ESM.zip › Cell type-specific predictions/chr5_99000000.pdf]

chr7:54500000-55500000

Akita

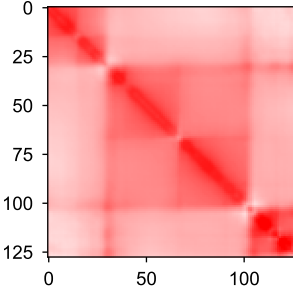

DeepC

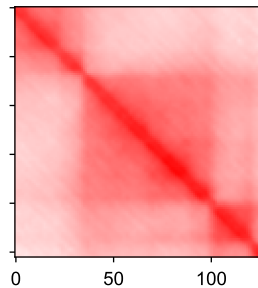

Orca

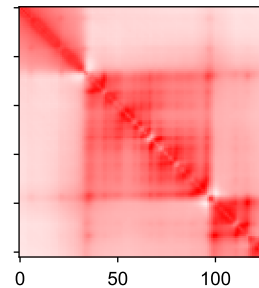

C.Origami on IMR-90

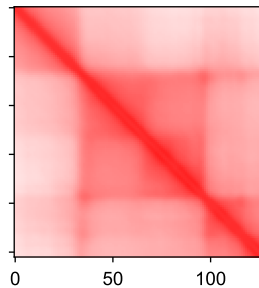

IMR-90 Hi-C

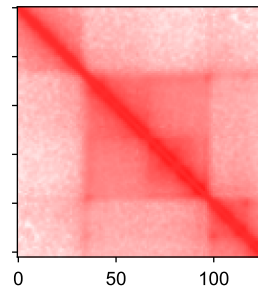

C.Origami on GM12878 (de novo)

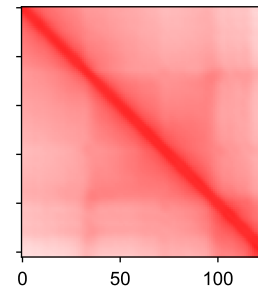

GM12878 Hi-C

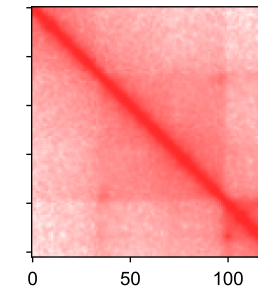

Supplement: Supplementary file 3 — Cell-type-specific predictions. [file 41587_2022_1612_MOESM3_ESM.zip › Cell type-specific predictions/chr7_55000000.pdf]

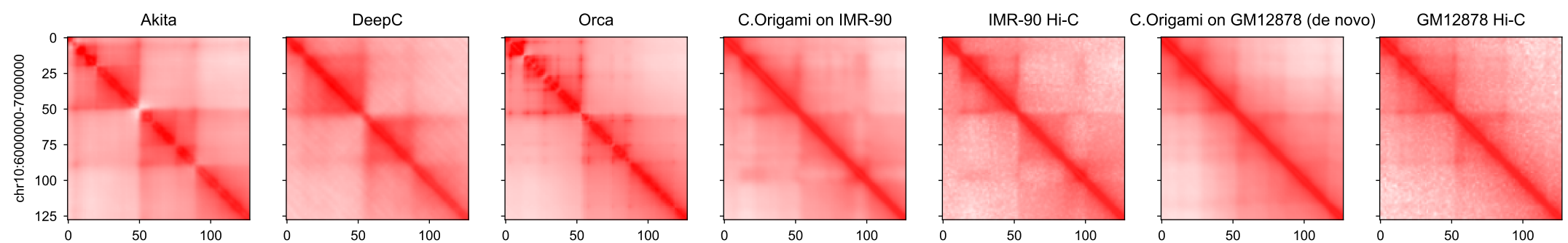

Supplement: Supplementary file 3 — Cell-type-specific predictions. [file 41587_2022_1612_MOESM3_ESM.zip › Cell type-specific predictions/chr10_6500000.pdf]

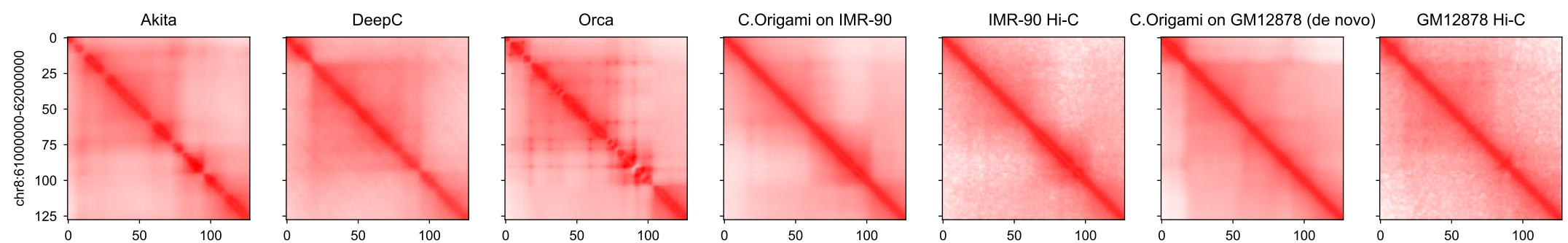

Supplement: Supplementary file 3 — Cell-type-specific predictions. [file 41587_2022_1612_MOESM3_ESM.zip › Cell type-specific predictions/chr8_61500000.pdf]

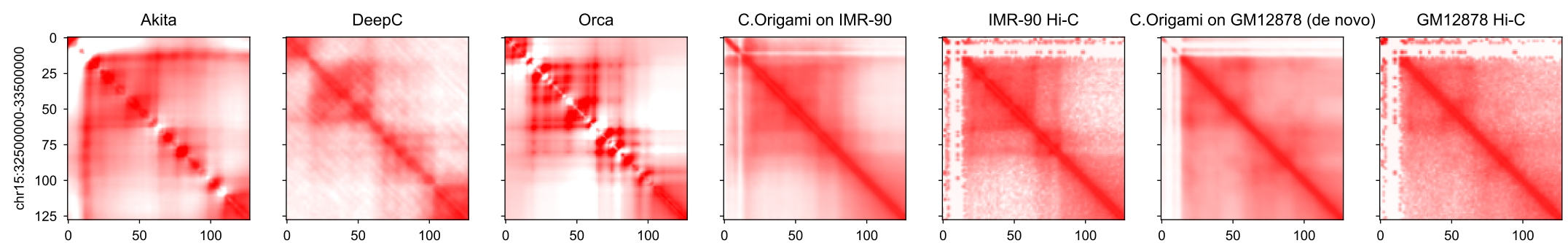

Supplement: Supplementary file 3 — Cell-type-specific predictions. [file 41587_2022_1612_MOESM3_ESM.zip › Cell type-specific predictions/chr15_33000000.pdf]

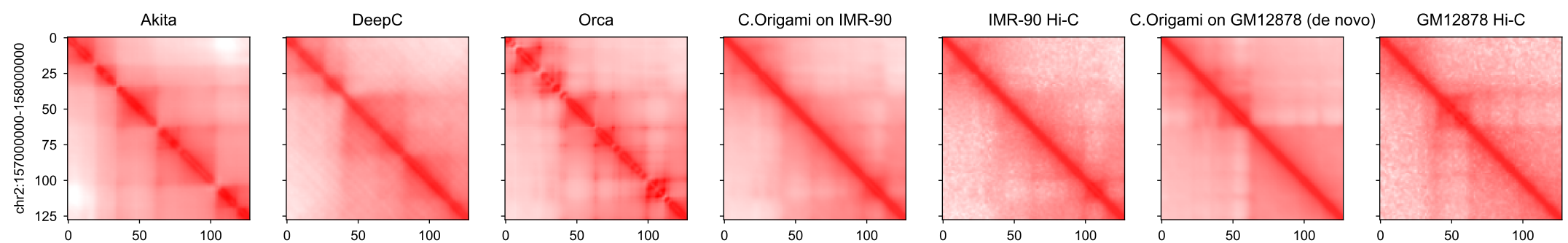

Supplement: Supplementary file 3 — Cell-type-specific predictions. [file 41587_2022_1612_MOESM3_ESM.zip › Cell type-specific predictions/chr2_157500000.pdf]

chr9:73000000-74000000

Akita

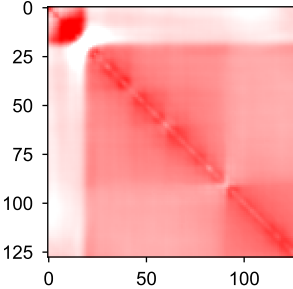

DeepC

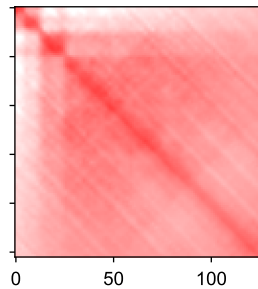

Orca

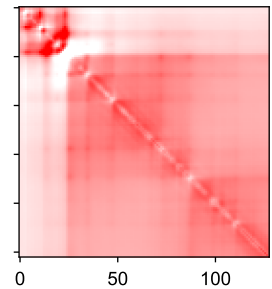

C.Origami on IMR-90

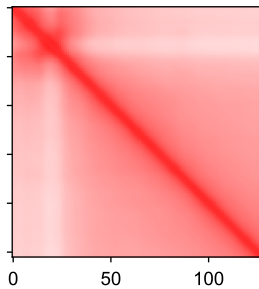

IMR-90 Hi-C

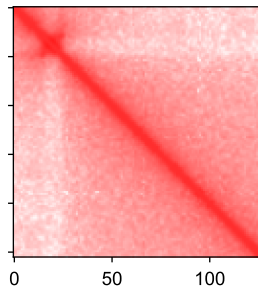

C.Origami on GM12878 (de novo)

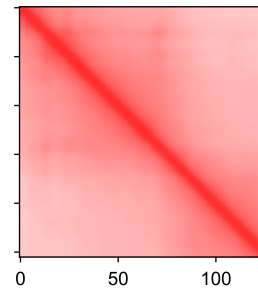

GM12878 Hi-C

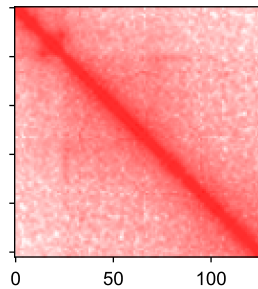

Supplement: Supplementary file 3 — Cell-type-specific predictions. [file 41587_2022_1612_MOESM3_ESM.zip › Cell type-specific predictions/chr9_73500000.pdf]

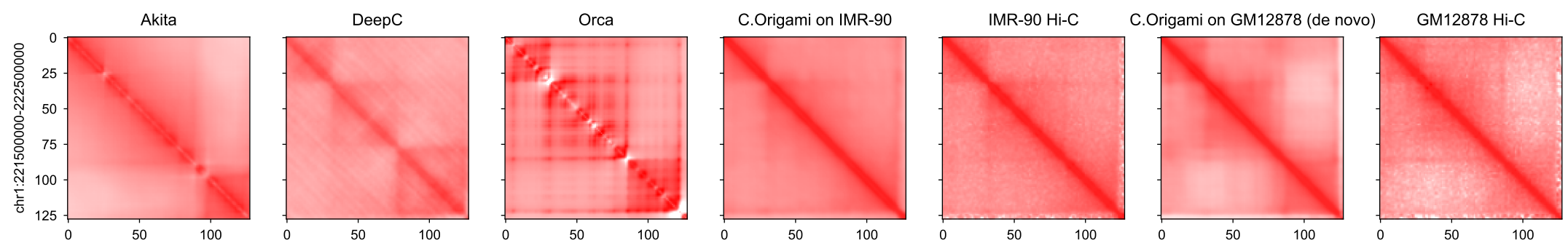

Supplement: Supplementary file 3 — Cell-type-specific predictions. [file 41587_2022_1612_MOESM3_ESM.zip › Cell type-specific predictions/chr1_222000000.pdf]

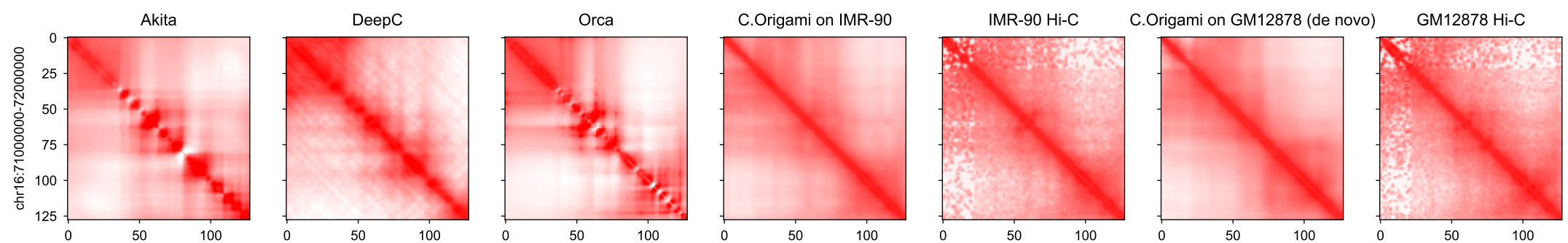

Supplement: Supplementary file 3 — Cell-type-specific predictions. [file 41587_2022_1612_MOESM3_ESM.zip › Cell type-specific predictions/chr16_71500000.pdf]

chr8:75000000-76000000

Akita

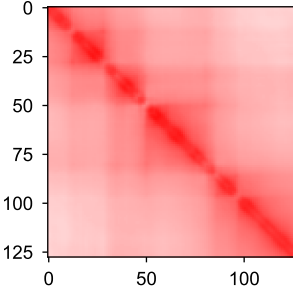

DeepC

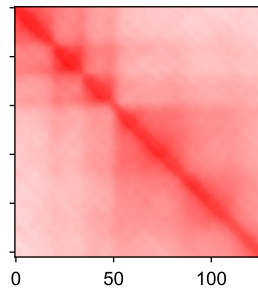

Orca

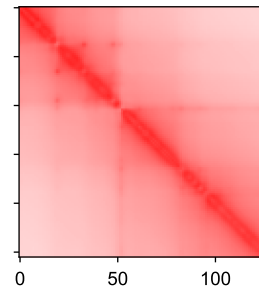

C.Origami on IMR-90

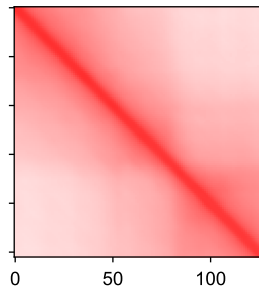

IMR-90 Hi-C

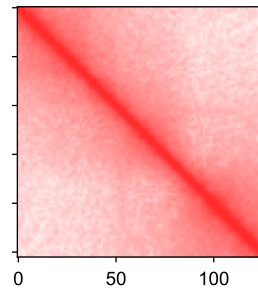

C.Origami on GM12878 (de novo)

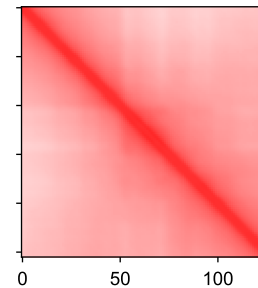

GM12878 Hi-C

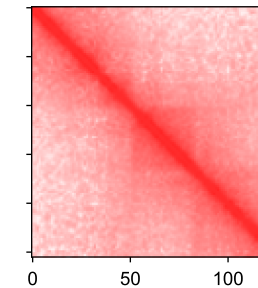

Supplement: Supplementary file 3 — Cell-type-specific predictions. [file 41587_2022_1612_MOESM3_ESM.zip › Cell type-specific predictions/chr8_75500000.pdf]

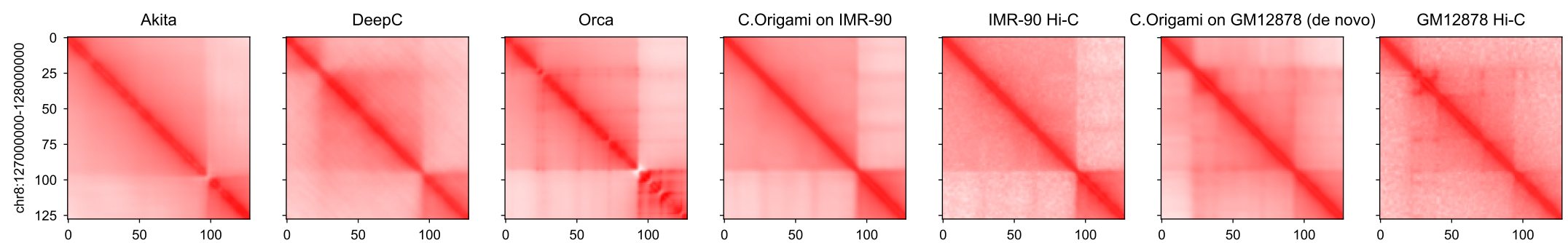

Supplement: Supplementary file 3 — Cell-type-specific predictions. [file 41587_2022_1612_MOESM3_ESM.zip › Cell type-specific predictions/chr8_127500000.pdf]

chr10:57500000-58500000

Akita

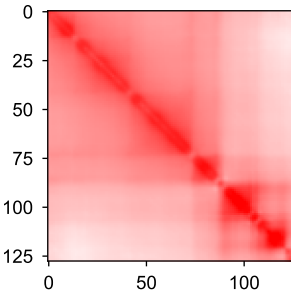

DeepC

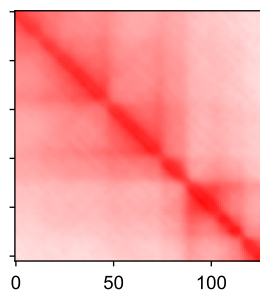

Orca

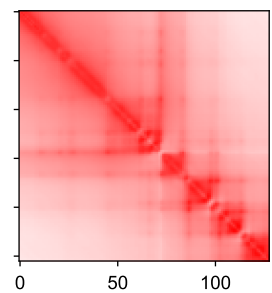

C.Origami on IMR-90

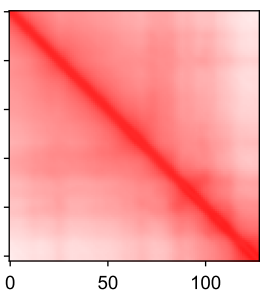

IMR-90 Hi-C

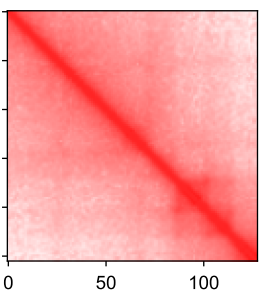

C.Origami on GM12878 (de novo)

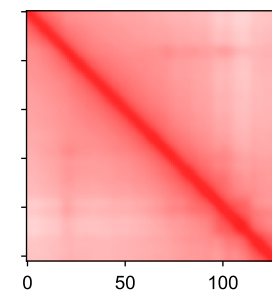

GM12878 Hi-C

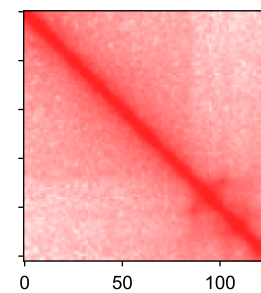

Supplement: Supplementary file 3 — Cell-type-specific predictions. [file 41587_2022_1612_MOESM3_ESM.zip › Cell type-specific predictions/chr10_58000000.pdf]

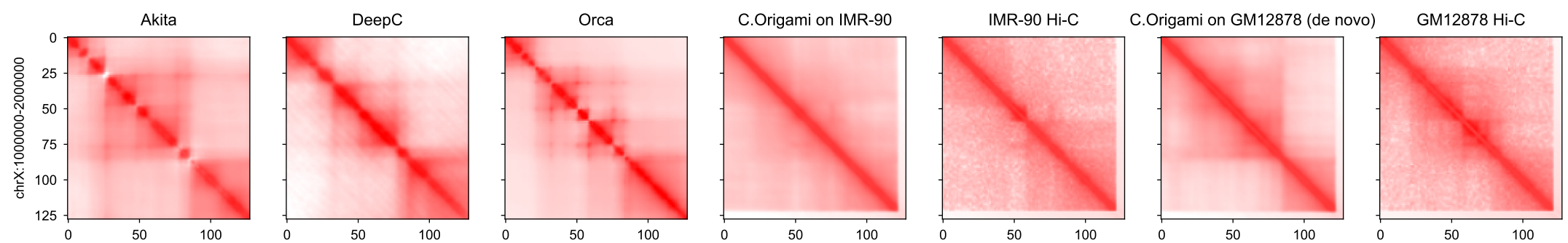

Supplement: Supplementary file 3 — Cell-type-specific predictions. [file 41587_2022_1612_MOESM3_ESM.zip › Cell type-specific predictions/chrX_1500000.pdf]

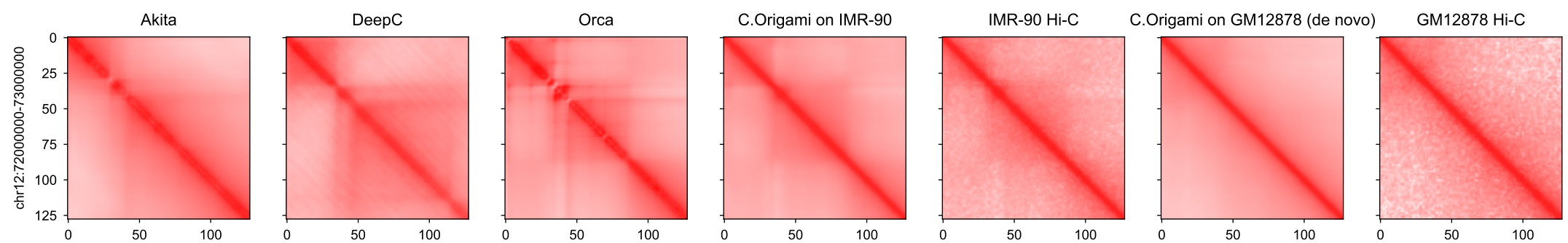

Supplement: Supplementary file 3 — Cell-type-specific predictions. [file 41587_2022_1612_MOESM3_ESM.zip › Cell type-specific predictions/chr12_72500000.pdf]

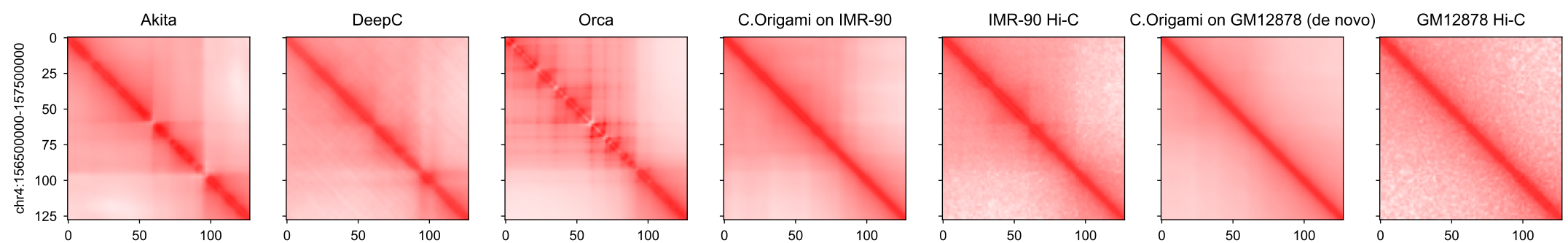

Supplement: Supplementary file 3 — Cell-type-specific predictions. [file 41587_2022_1612_MOESM3_ESM.zip › Cell type-specific predictions/chr4_157000000.pdf]

chr7:87500000-88500000

Akita

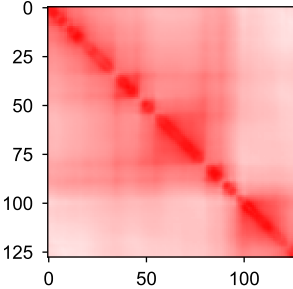

DeepC

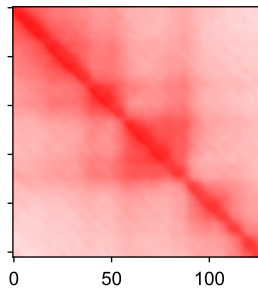

Orca

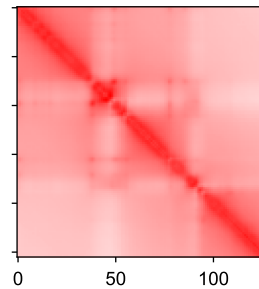

C.Origami on IMR-90

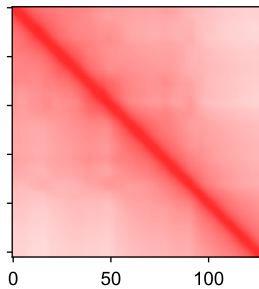

IMR-90 Hi-C

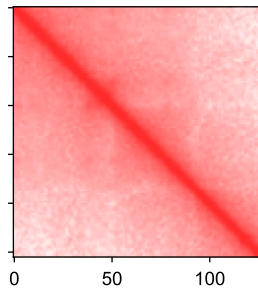

C.Origami on GM12878 (de novo)

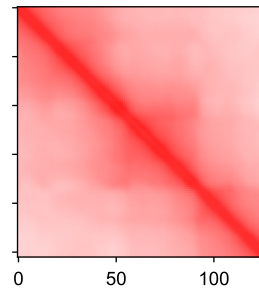

GM12878 Hi-C

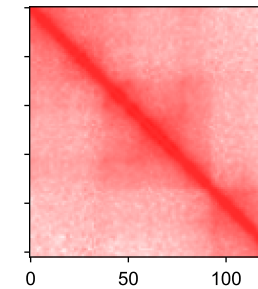

Supplement: Supplementary file 3 — Cell-type-specific predictions. [file 41587_2022_1612_MOESM3_ESM.zip › Cell type-specific predictions/chr7_88000000.pdf]

chr17:57500000-58500000

Akita

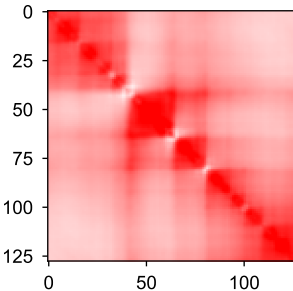

DeepC

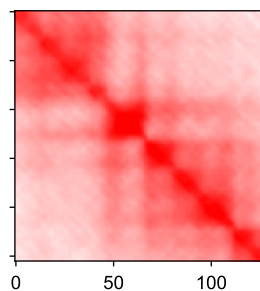

Orca

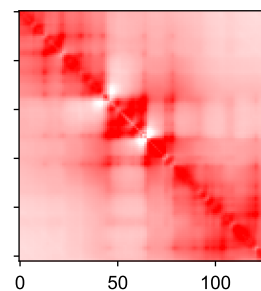

C.Origami on IMR-90

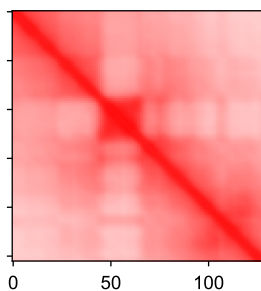

IMR-90 Hi-C

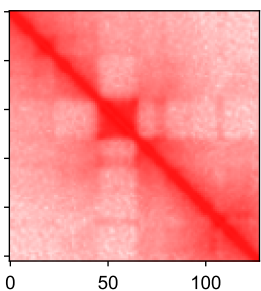

C.Origami on GM12878 (de novo)

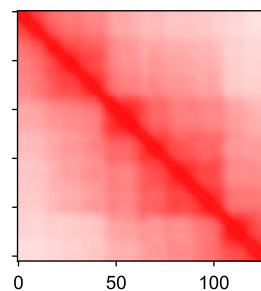

GM12878 Hi-C

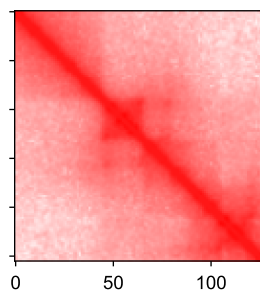

Supplement: Supplementary file 3 — Cell-type-specific predictions. [file 41587_2022_1612_MOESM3_ESM.zip › Cell type-specific predictions/chr17_58000000.pdf]

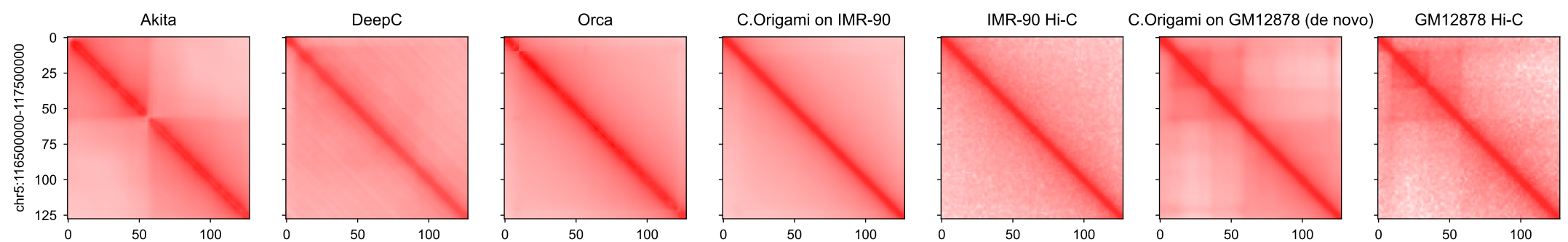

Supplement: Supplementary file 3 — Cell-type-specific predictions. [file 41587_2022_1612_MOESM3_ESM.zip › Cell type-specific predictions/chr5_117000000.pdf]

chr5:33500000-34500000

Akita

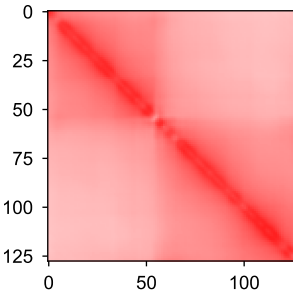

DeepC

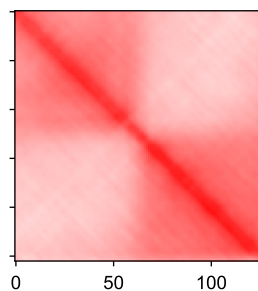

Orca

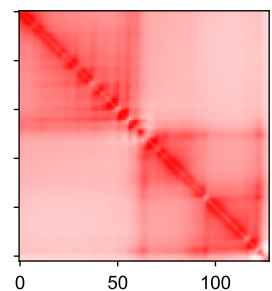

C.Origami on IMR-90

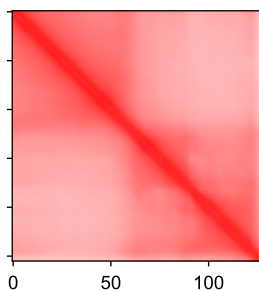

IMR-90 Hi-C

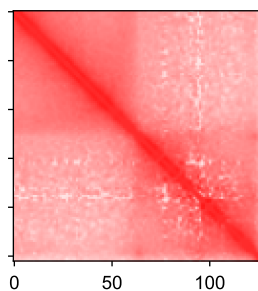

C.Origami on GM12878 (de novo)

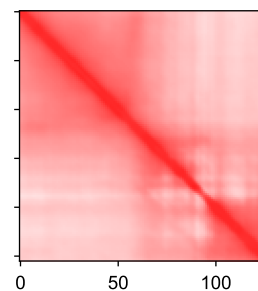

GM12878 Hi-C

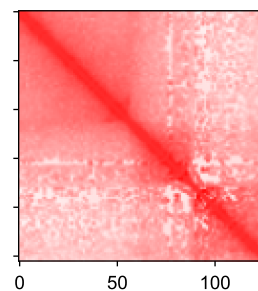

Supplement: Supplementary file 3 — Cell-type-specific predictions. [file 41587_2022_1612_MOESM3_ESM.zip › Cell type-specific predictions/chr5_34000000.pdf]

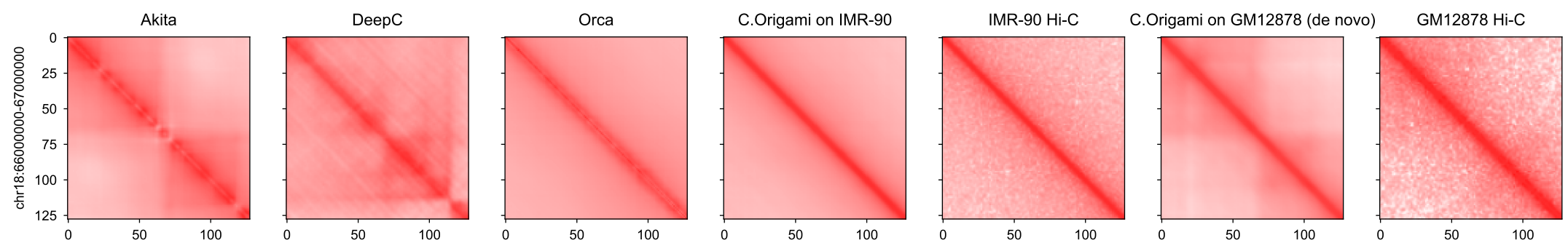

Supplement: Supplementary file 3 — Cell-type-specific predictions. [file 41587_2022_1612_MOESM3_ESM.zip › Cell type-specific predictions/chr18_66500000.pdf]

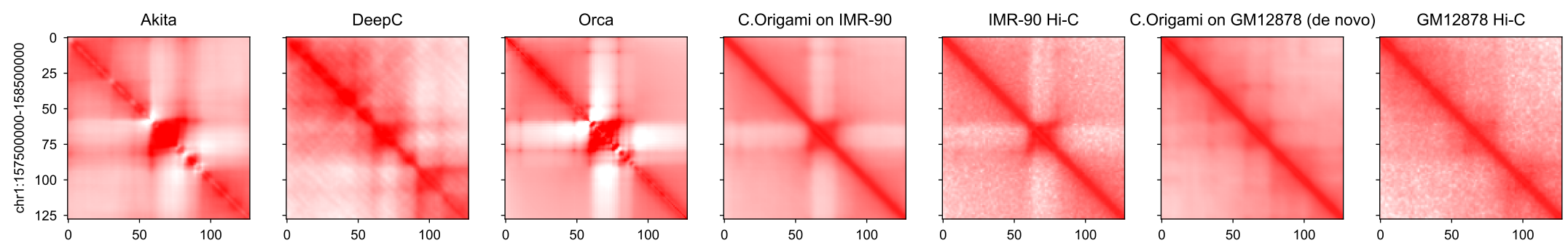

Supplement: Supplementary file 3 — Cell-type-specific predictions. [file 41587_2022_1612_MOESM3_ESM.zip › Cell type-specific predictions/chr1_158000000.pdf]

chr9:79000000-80000000

Akita

DeepC

Orca

C.Origami on IMR-90

IMR-90 Hi-C

C.Origami on GM12878 (de novo)

GM12878 Hi-C

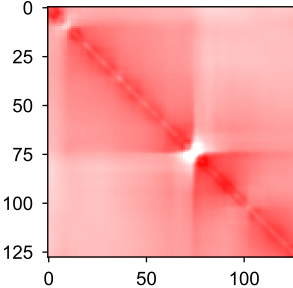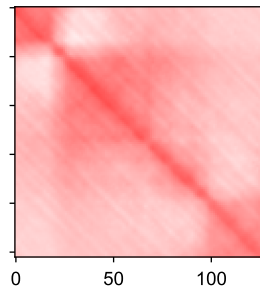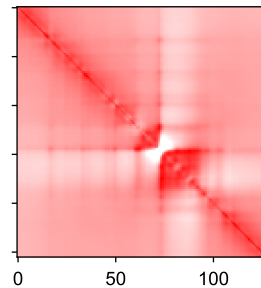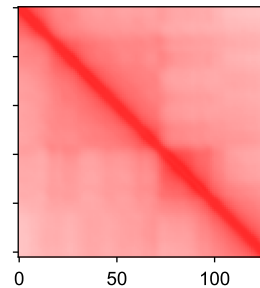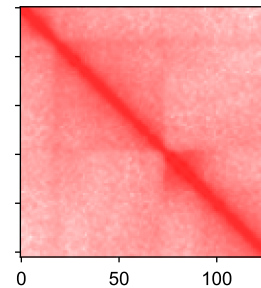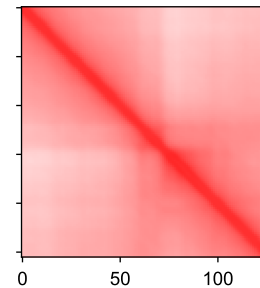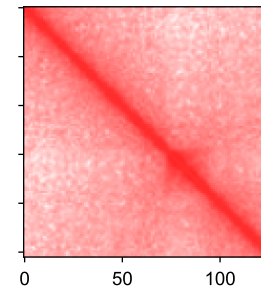

Supplement: Supplementary file 3 — Cell-type-specific predictions. [file 41587_2022_1612_MOESM3_ESM.zip › Cell type-specific predictions/chr9_79500000.pdf]

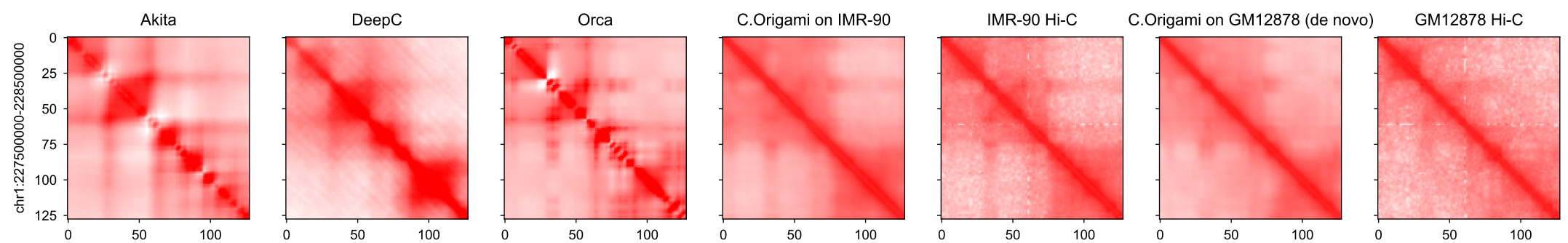

Supplement: Supplementary file 3 — Cell-type-specific predictions. [file 41587_2022_1612_MOESM3_ESM.zip › Cell type-specific predictions/chr1_228000000.pdf]

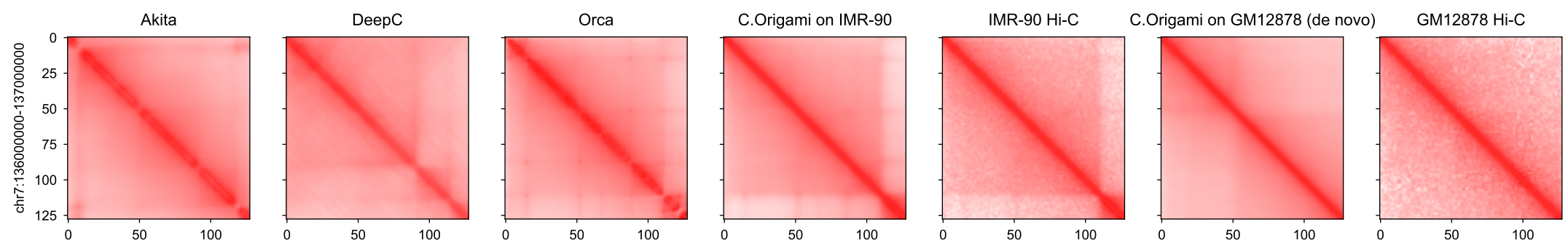

Supplement: Supplementary file 3 — Cell-type-specific predictions. [file 41587_2022_1612_MOESM3_ESM.zip › Cell type-specific predictions/chr7_136500000.pdf]

chrX:90500000-91500000

Akita

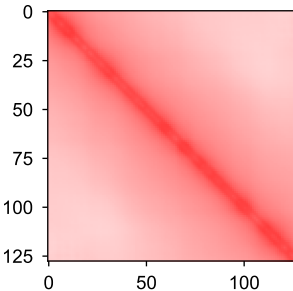

DeepC

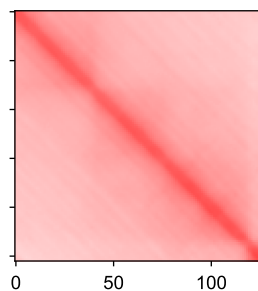

Orca

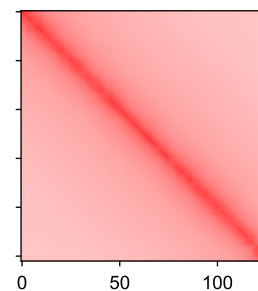

C.Origami on IMR-90

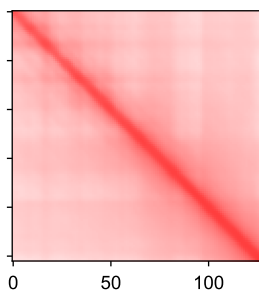

IMR-90 Hi-C

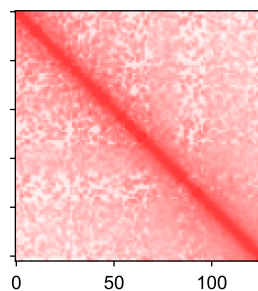

C.Origami on GM12878 (de novo)

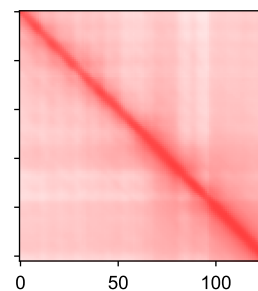

GM12878 Hi-C

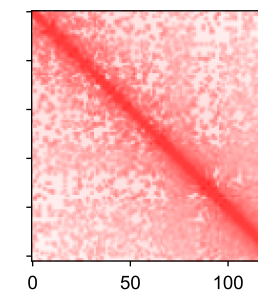

Supplement: Supplementary file 3 — Cell-type-specific predictions. [file 41587_2022_1612_MOESM3_ESM.zip › Cell type-specific predictions/chrX_91000000.pdf]

chrX:57500000-58500000

Akita

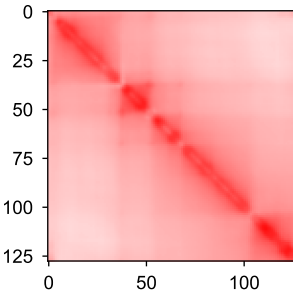

DeepC

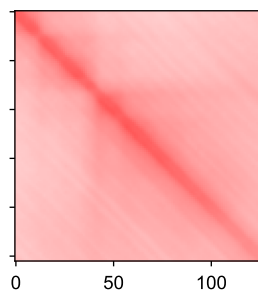

Orca

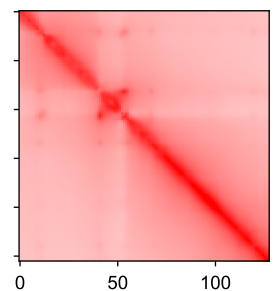

C.Origami on IMR-90

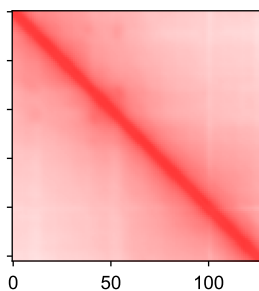

IMR-90 Hi-C

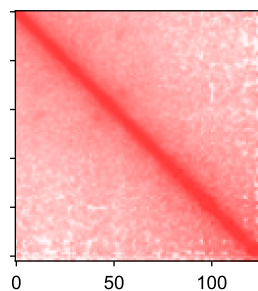

C.Origami on GM12878 (de novo)

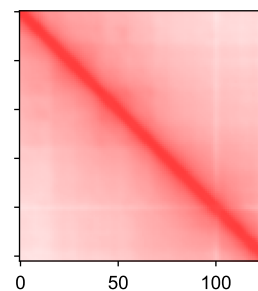

GM12878 Hi-C

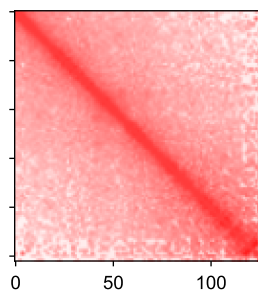

Supplement: Supplementary file 3 — Cell-type-specific predictions. [file 41587_2022_1612_MOESM3_ESM.zip › Cell type-specific predictions/chrX_58000000.pdf]

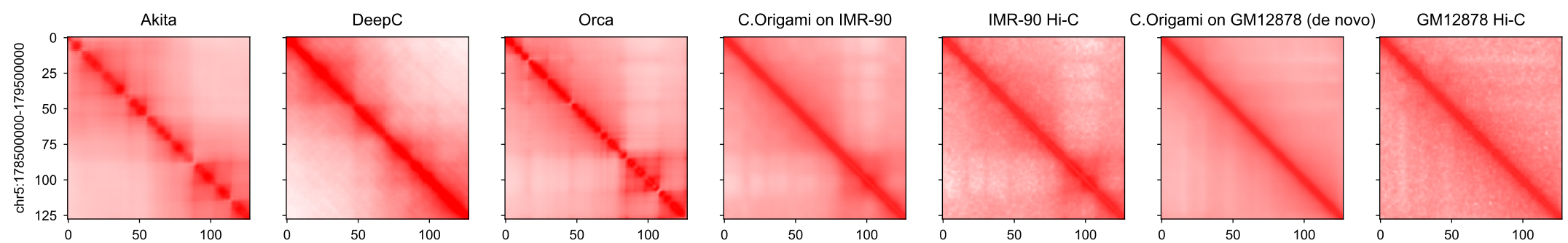

Supplement: Supplementary file 3 — Cell-type-specific predictions. [file 41587_2022_1612_MOESM3_ESM.zip › Cell type-specific predictions/chr5_179000000.pdf]

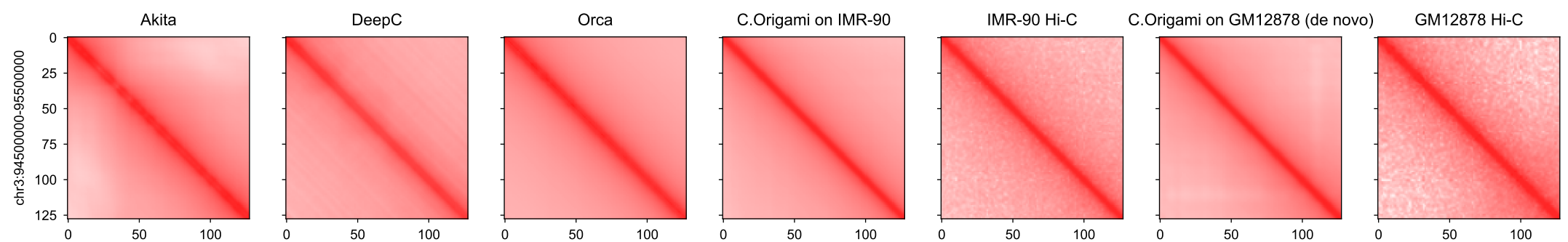

Supplement: Supplementary file 3 — Cell-type-specific predictions. [file 41587_2022_1612_MOESM3_ESM.zip › Cell type-specific predictions/chr3_95000000.pdf]

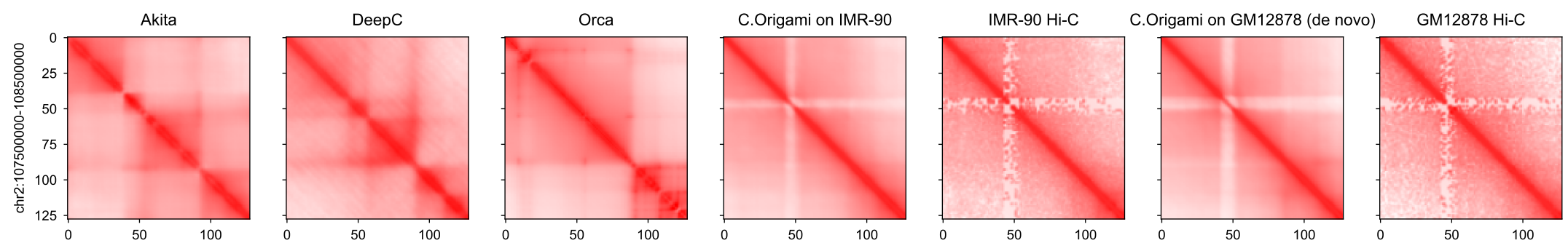

Supplement: Supplementary file 3 — Cell-type-specific predictions. [file 41587_2022_1612_MOESM3_ESM.zip › Cell type-specific predictions/chr2_108000000.pdf]

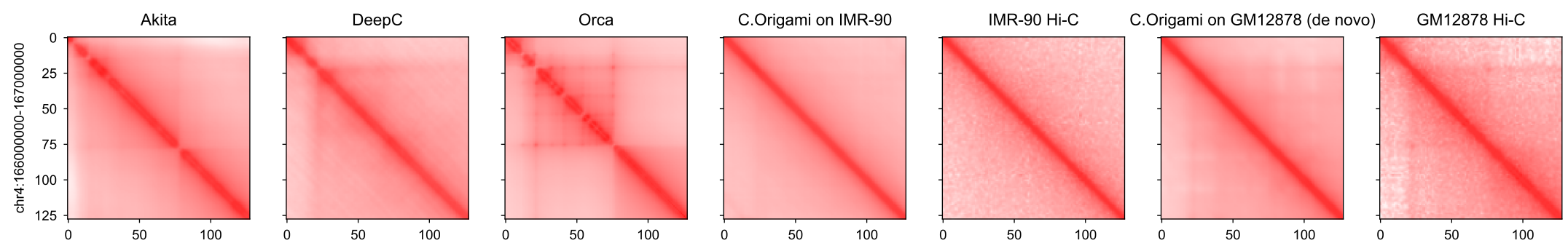

Supplement: Supplementary file 3 — Cell-type-specific predictions. [file 41587_2022_1612_MOESM3_ESM.zip › Cell type-specific predictions/chr4_166500000.pdf]

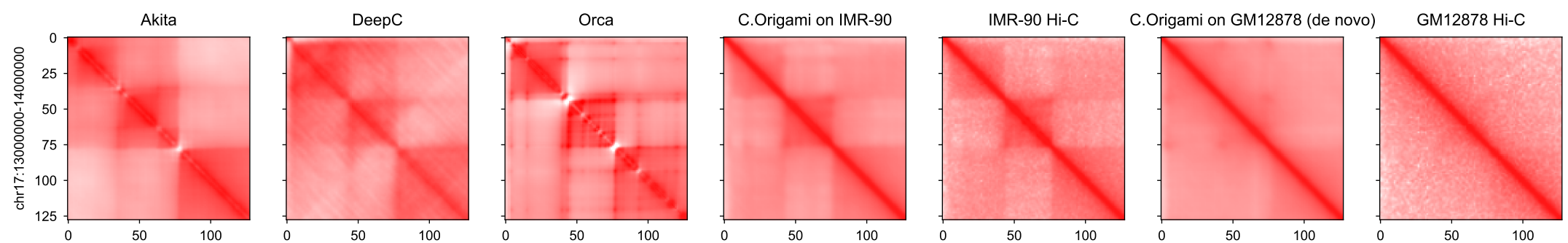

Supplement: Supplementary file 3 — Cell-type-specific predictions. [file 41587_2022_1612_MOESM3_ESM.zip › Cell type-specific predictions/chr17_13500000.pdf]

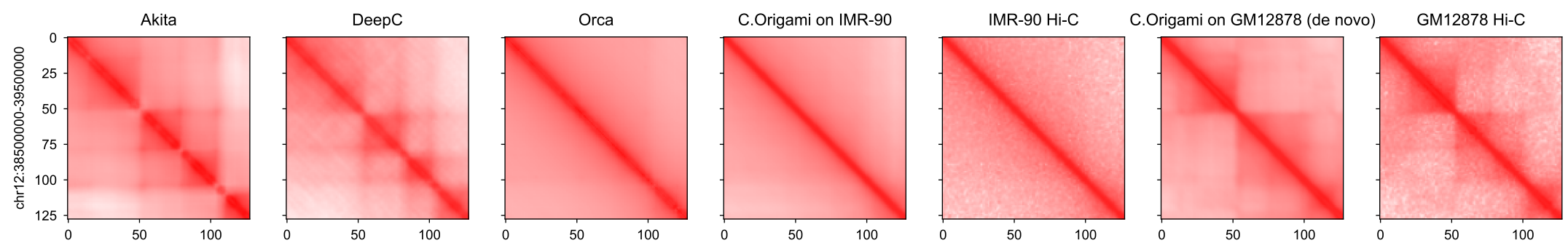

Supplement: Supplementary file 3 — Cell-type-specific predictions. [file 41587_2022_1612_MOESM3_ESM.zip › Cell type-specific predictions/chr12_39000000.pdf]

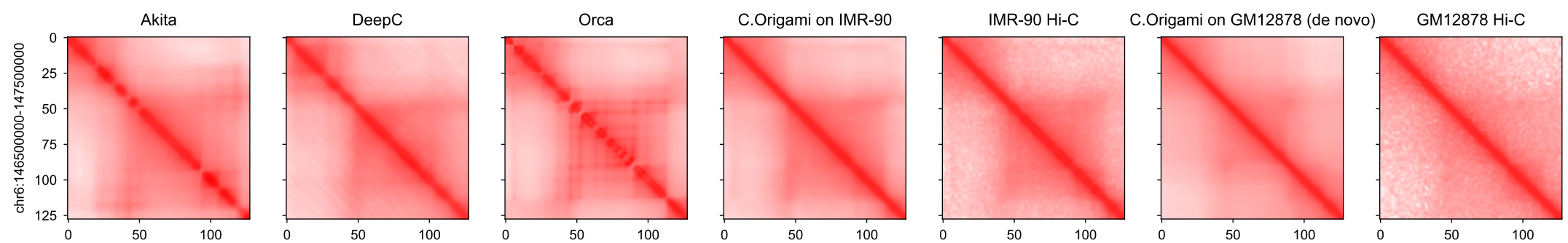

Supplement: Supplementary file 3 — Cell-type-specific predictions. [file 41587_2022_1612_MOESM3_ESM.zip › Cell type-specific predictions/chr6_147000000.pdf]

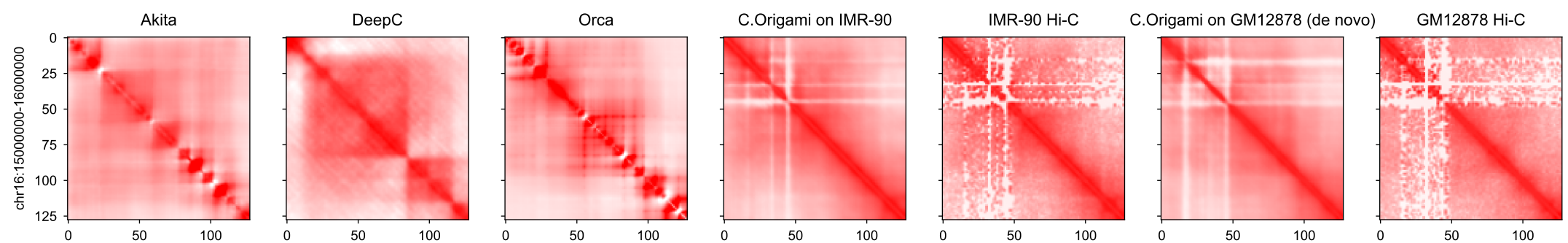

Supplement: Supplementary file 3 — Cell-type-specific predictions. [file 41587_2022_1612_MOESM3_ESM.zip › Cell type-specific predictions/chr16_15500000.pdf]

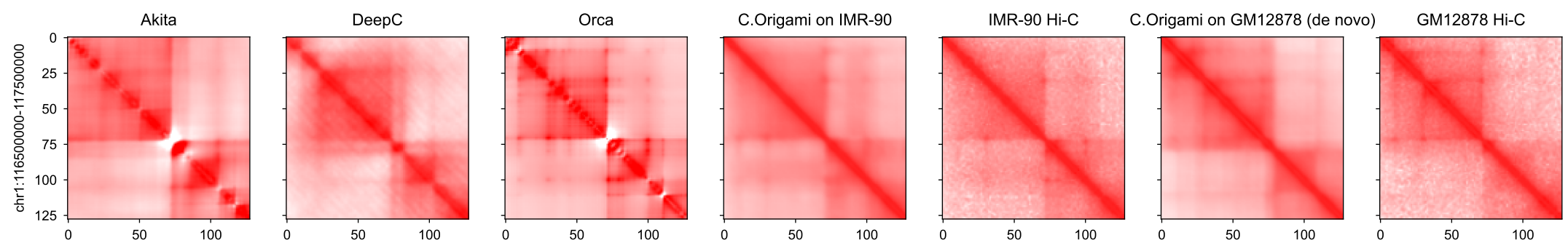

Supplement: Supplementary file 3 — Cell-type-specific predictions. [file 41587_2022_1612_MOESM3_ESM.zip › Cell type-specific predictions/chr1_117000000.pdf]

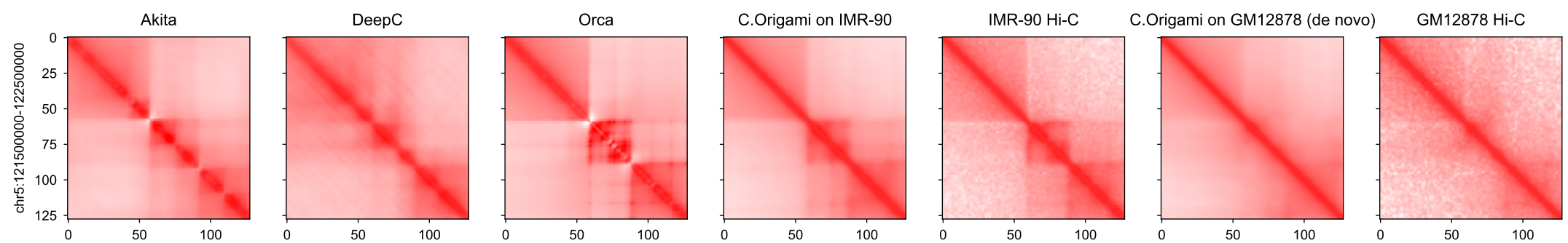

Supplement: Supplementary file 3 — Cell-type-specific predictions. [file 41587_2022_1612_MOESM3_ESM.zip › Cell type-specific predictions/chr5_122000000.pdf]

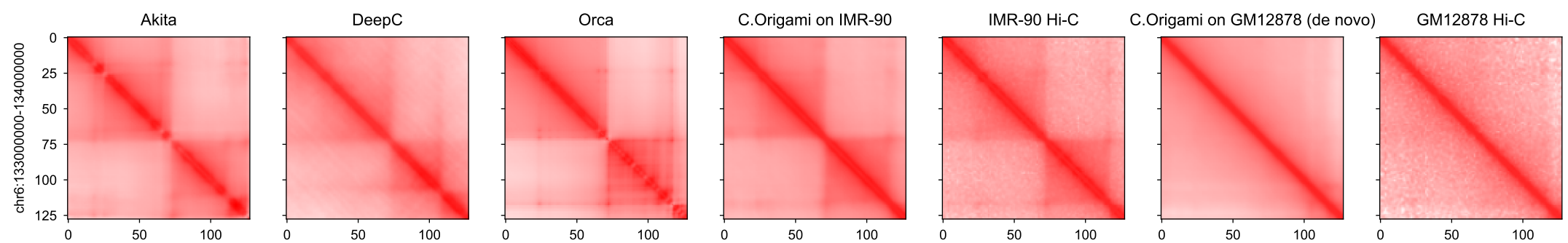

Supplement: Supplementary file 3 — Cell-type-specific predictions. [file 41587_2022_1612_MOESM3_ESM.zip › Cell type-specific predictions/chr6_133500000.pdf]

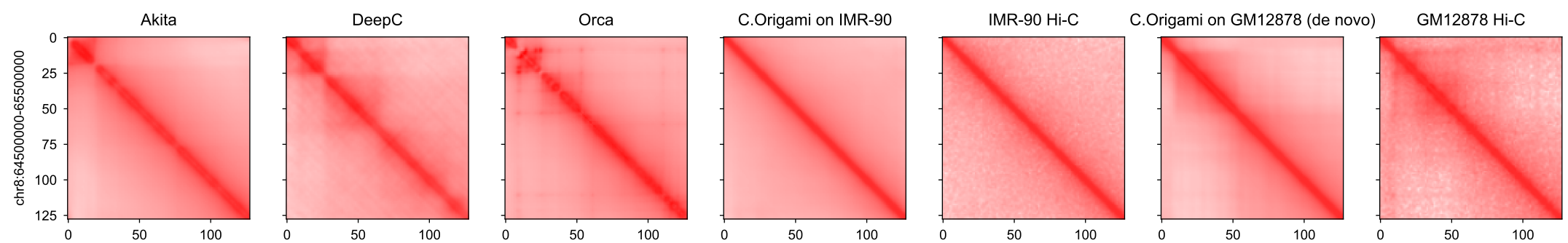

Supplement: Supplementary file 3 — Cell-type-specific predictions. [file 41587_2022_1612_MOESM3_ESM.zip › Cell type-specific predictions/chr8_65000000.pdf]

chr20:58500000-59500000

Akita

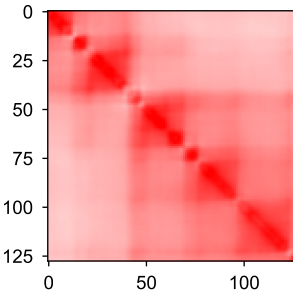

DeepC

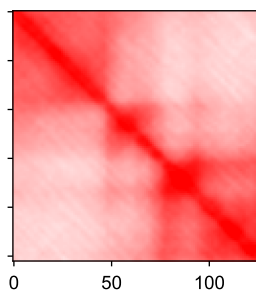

Orca

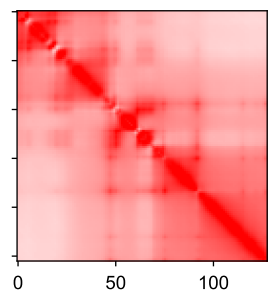

C.Origami on IMR-90

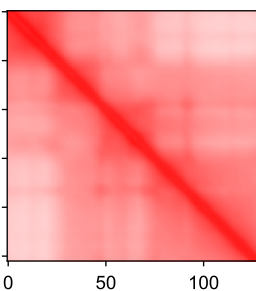

IMR-90 Hi-C

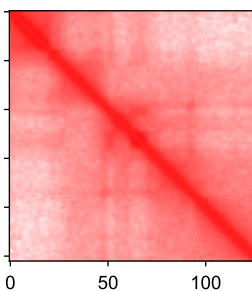

C.Origami on GM12878 (de novo)

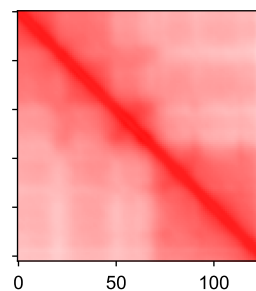

GM12878 Hi-C

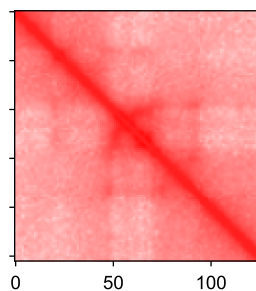

Supplement: Supplementary file 3 — Cell-type-specific predictions. [file 41587_2022_1612_MOESM3_ESM.zip › Cell type-specific predictions/chr20_59000000.pdf]

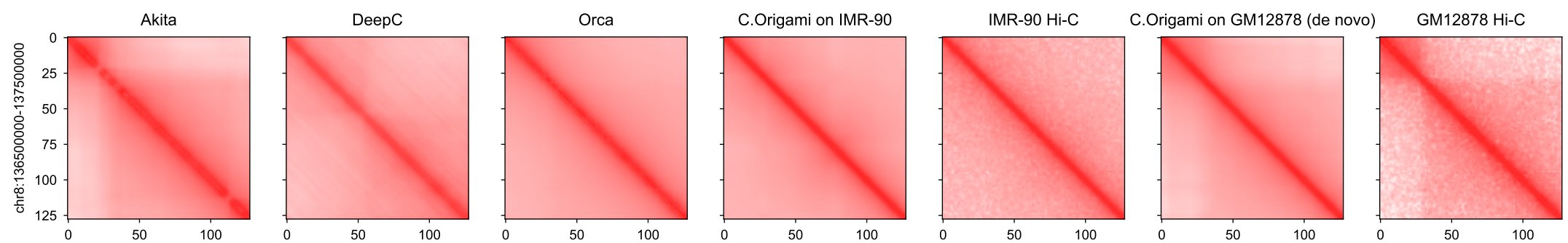

Supplement: Supplementary file 3 — Cell-type-specific predictions. [file 41587_2022_1612_MOESM3_ESM.zip › Cell type-specific predictions/chr8_137000000.pdf]

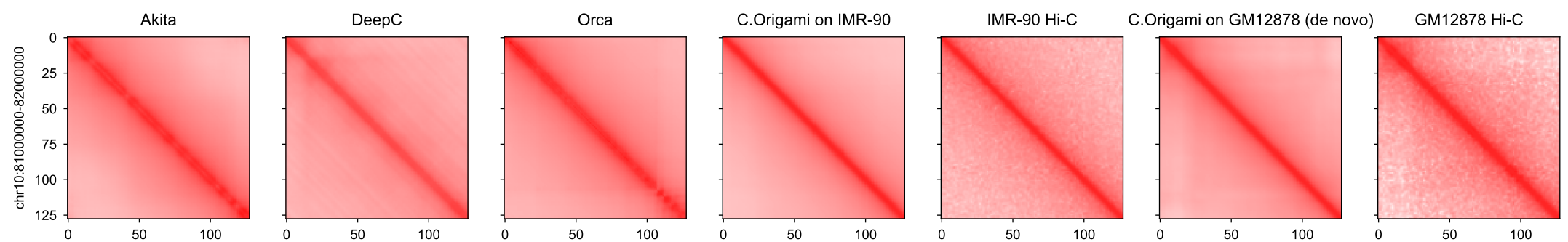

Supplement: Supplementary file 3 — Cell-type-specific predictions. [file 41587_2022_1612_MOESM3_ESM.zip › Cell type-specific predictions/chr10_81500000.pdf]

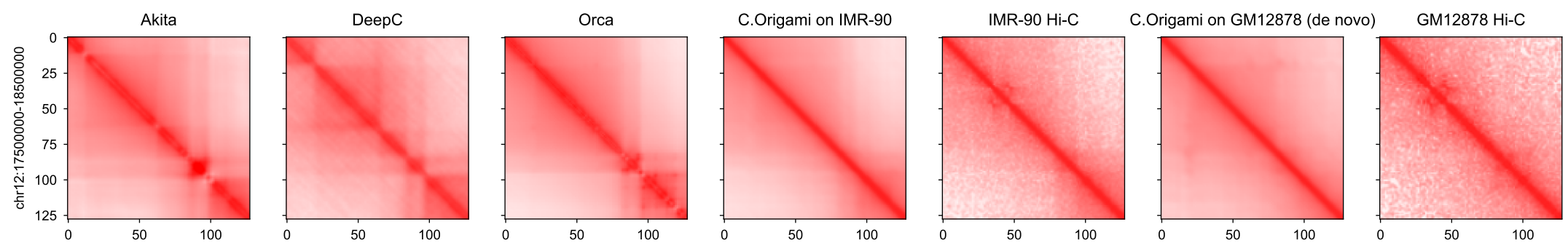

Supplement: Supplementary file 3 — Cell-type-specific predictions. [file 41587_2022_1612_MOESM3_ESM.zip › Cell type-specific predictions/chr12_18000000.pdf]

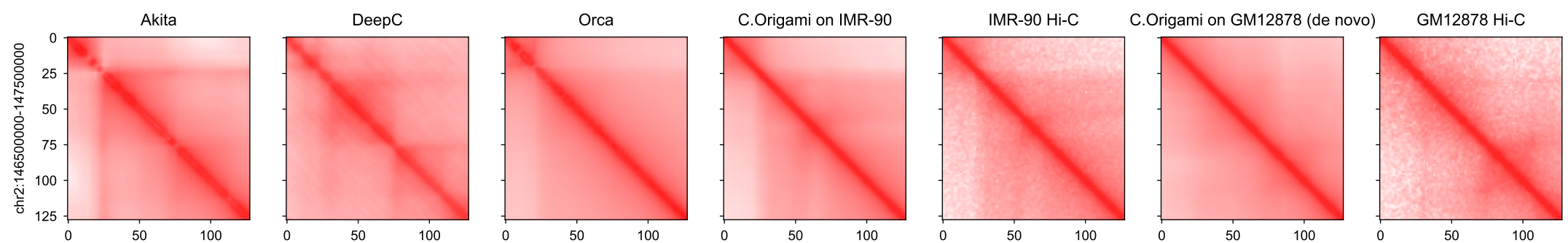

Supplement: Supplementary file 3 — Cell-type-specific predictions. [file 41587_2022_1612_MOESM3_ESM.zip › Cell type-specific predictions/chr2_147000000.pdf]

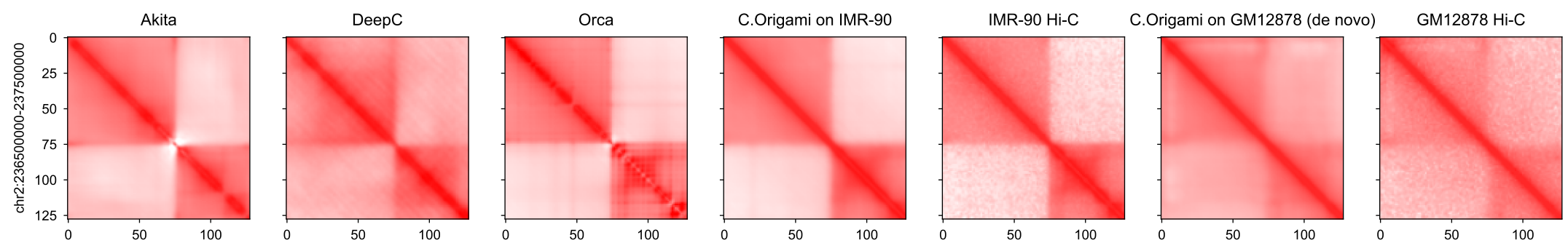

Supplement: Supplementary file 3 — Cell-type-specific predictions. [file 41587_2022_1612_MOESM3_ESM.zip › Cell type-specific predictions/chr2_237000000.pdf]

chr9:22000000-23000000

Akita

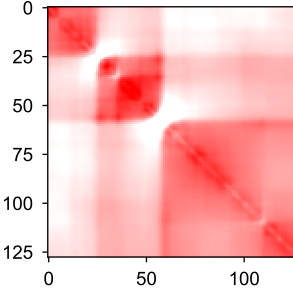

DeepC

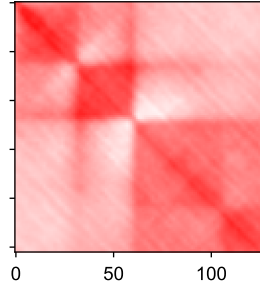

Orca

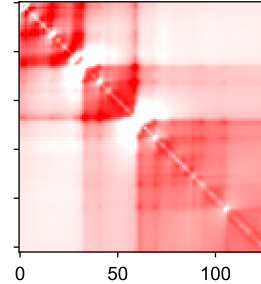

C.Origami on IMR-90

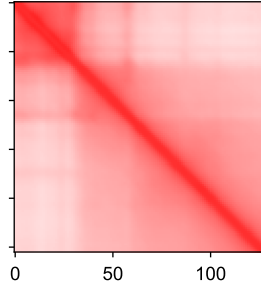

IMR-90 Hi-C

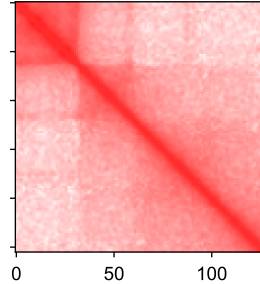

C.Origami on GM12878 (de novo)

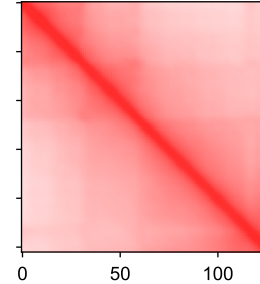

GM12878 Hi-C

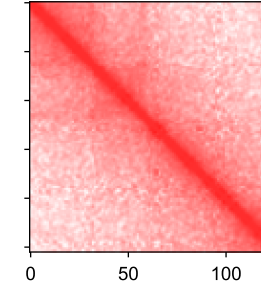

Supplement: Supplementary file 3 — Cell-type-specific predictions. [file 41587_2022_1612_MOESM3_ESM.zip › Cell type-specific predictions/chr9_22500000.pdf]

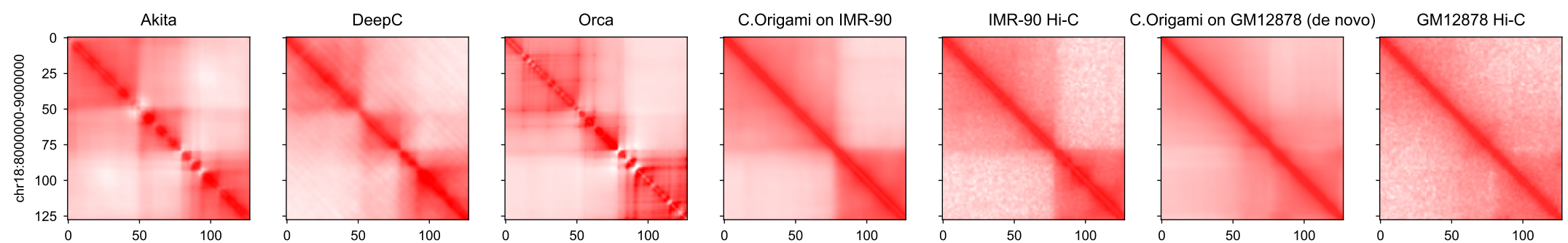

Supplement: Supplementary file 3 — Cell-type-specific predictions. [file 41587_2022_1612_MOESM3_ESM.zip › Cell type-specific predictions/chr18_8500000.pdf]

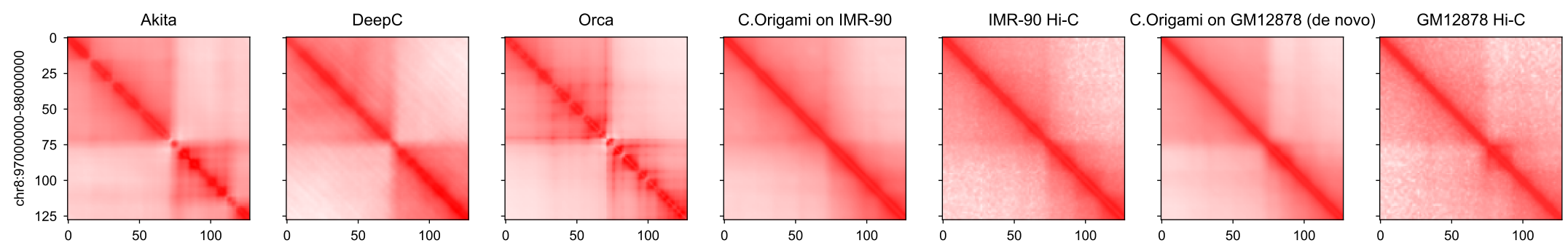

Supplement: Supplementary file 3 — Cell-type-specific predictions. [file 41587_2022_1612_MOESM3_ESM.zip › Cell type-specific predictions/chr8_97500000.pdf]

chr19:41000000-42000000

Akita

DeepC

Orca

C.Origami on IMR-90

IMR-90 Hi-C

C.Origami on GM12878 (de novo)

GM12878 Hi-C

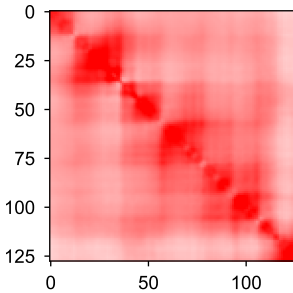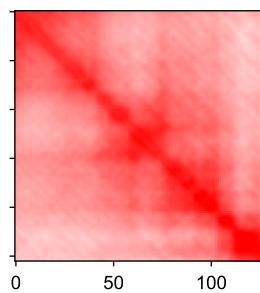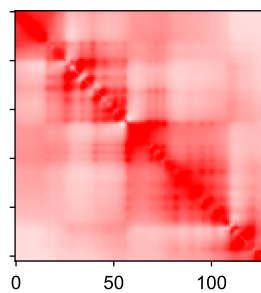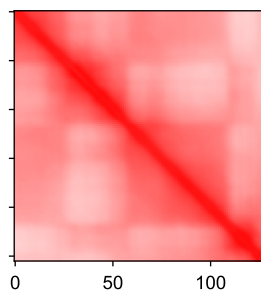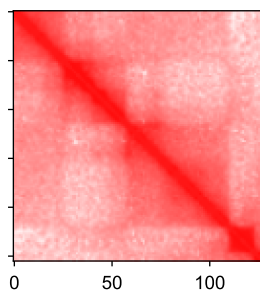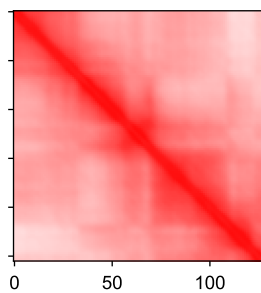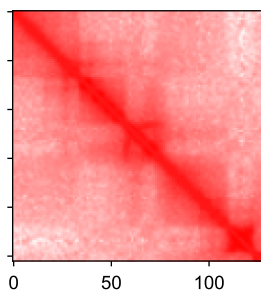

Supplement: Supplementary file 3 — Cell-type-specific predictions. [file 41587_2022_1612_MOESM3_ESM.zip › Cell type-specific predictions/chr19_41500000.pdf]

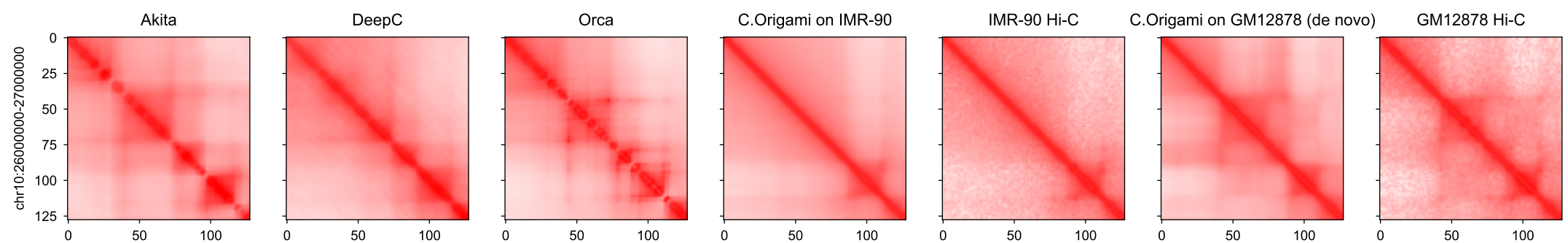

Supplement: Supplementary file 3 — Cell-type-specific predictions. [file 41587_2022_1612_MOESM3_ESM.zip › Cell type-specific predictions/chr10_26500000.pdf]

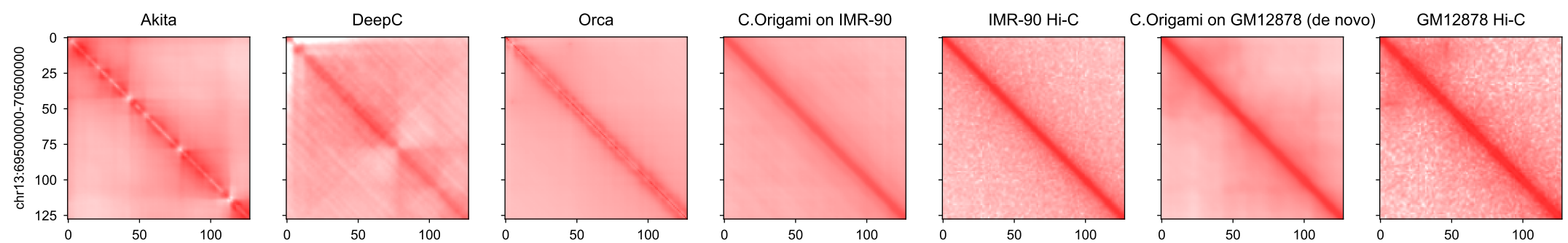

Supplement: Supplementary file 3 — Cell-type-specific predictions. [file 41587_2022_1612_MOESM3_ESM.zip › Cell type-specific predictions/chr13_70000000.pdf]

chr14:94500000-95500000

Akita

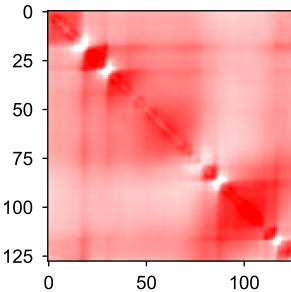

DeepC

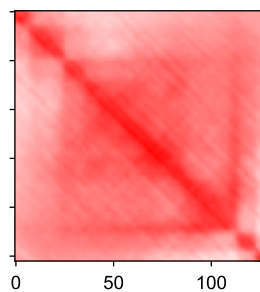

Orca

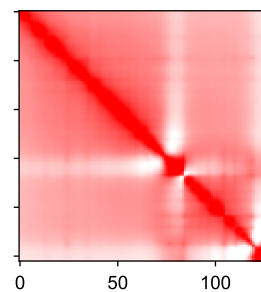

C.Origami on IMR-90

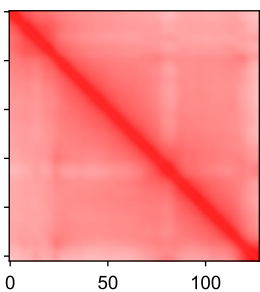

IMR-90 Hi-C

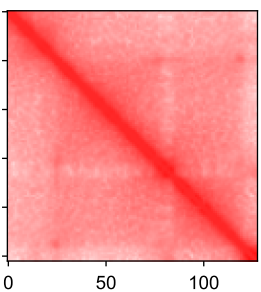

C.Origami on GM12878 (de novo)

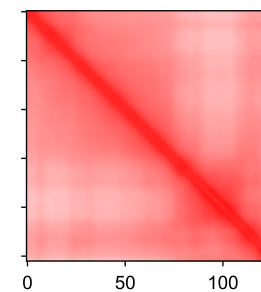

GM12878 Hi-C

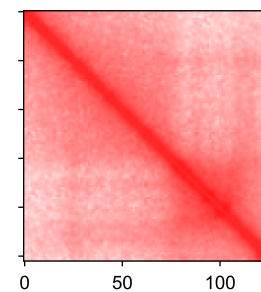

Supplement: Supplementary file 3 — Cell-type-specific predictions. [file 41587_2022_1612_MOESM3_ESM.zip › Cell type-specific predictions/chr14_95000000.pdf]

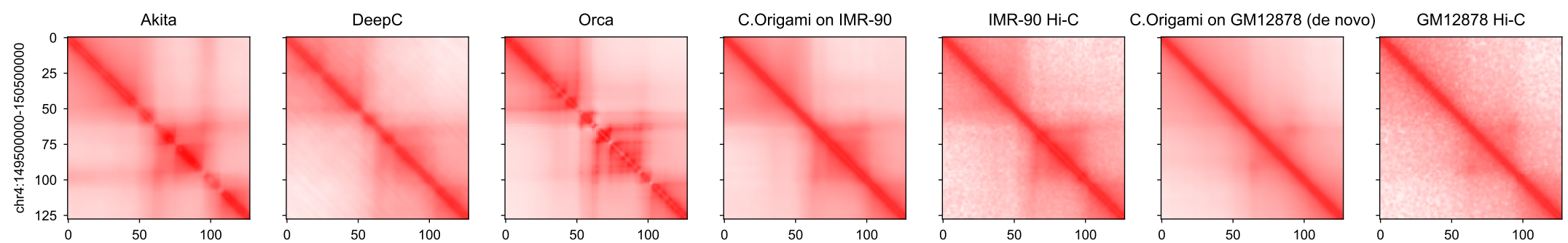

Supplement: Supplementary file 3 — Cell-type-specific predictions. [file 41587_2022_1612_MOESM3_ESM.zip › Cell type-specific predictions/chr4_150000000.pdf]

chr11:89500000-90500000

Akita

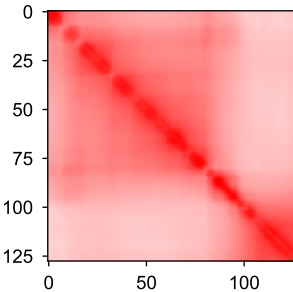

DeepC

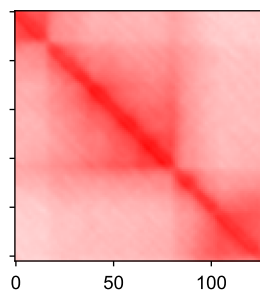

Orca

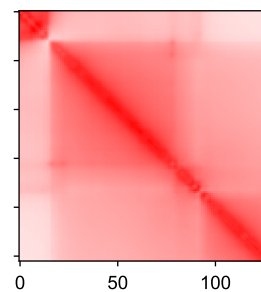

C.Origami on IMR-90

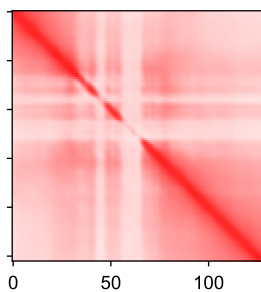

IMR-90 Hi-C

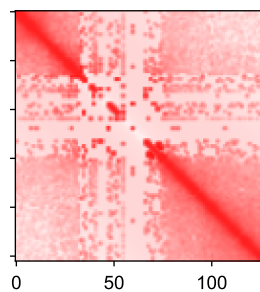

C.Origami on GM12878 (de novo)

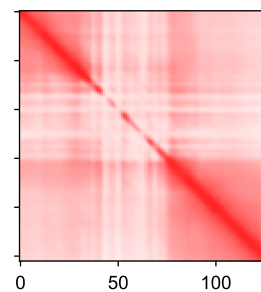

GM12878 Hi-C

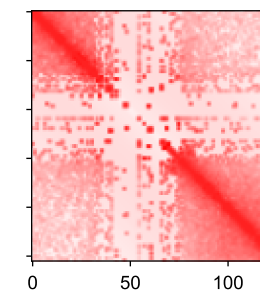

Supplement: Supplementary file 3 — Cell-type-specific predictions. [file 41587_2022_1612_MOESM3_ESM.zip › Cell type-specific predictions/chr11_90000000.pdf]

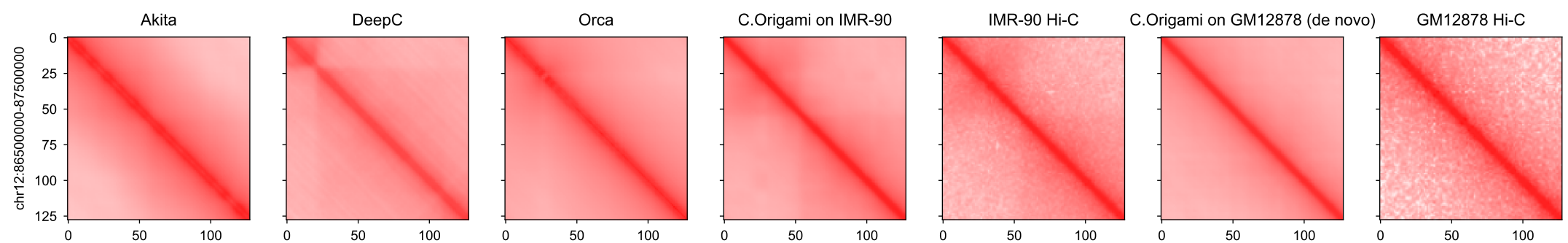

Supplement: Supplementary file 3 — Cell-type-specific predictions. [file 41587_2022_1612_MOESM3_ESM.zip › Cell type-specific predictions/chr12_87000000.pdf]

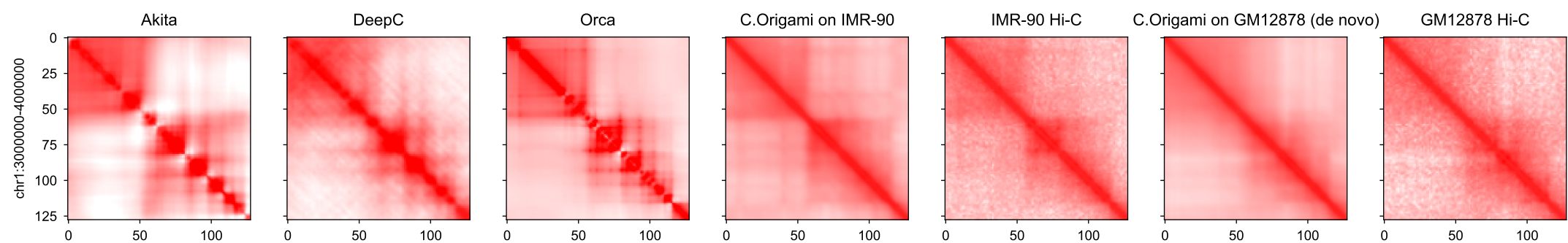

Supplement: Supplementary file 3 — Cell-type-specific predictions. [file 41587_2022_1612_MOESM3_ESM.zip › Cell type-specific predictions/chr1_3500000.pdf]

chr17:64000000-65000000

Akita

DeepC

Orca

C.Origami on IMR-90

IMR-90 Hi-C

C.Origami on GM12878 (de novo)

GM12878 Hi-C

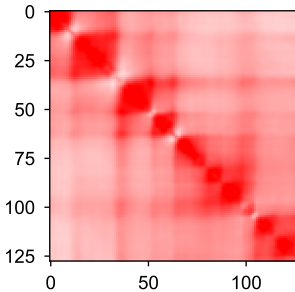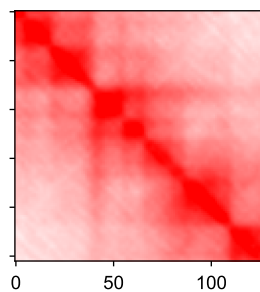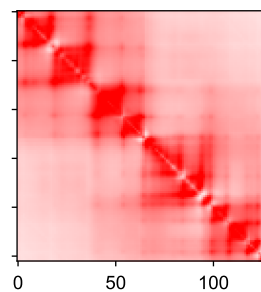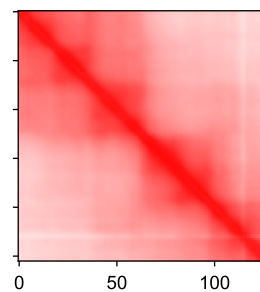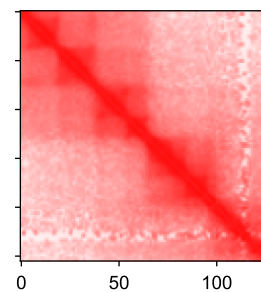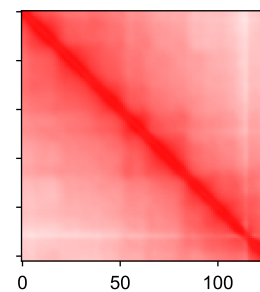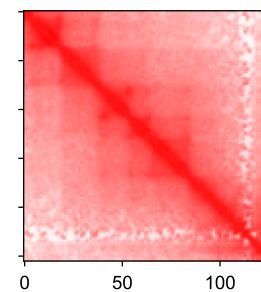

Supplement: Supplementary file 3 — Cell-type-specific predictions. [file 41587_2022_1612_MOESM3_ESM.zip › Cell type-specific predictions/chr17_64500000.pdf]

chrX:54500000-55500000

Akita

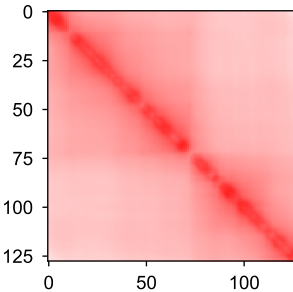

DeepC

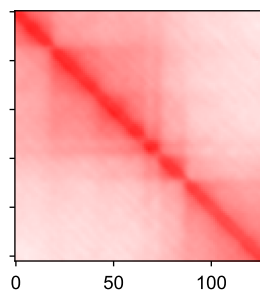

Orca

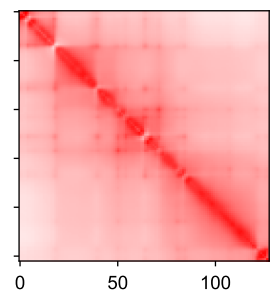

C.Origami on IMR-90

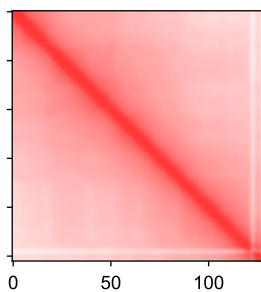

IMR-90 Hi-C

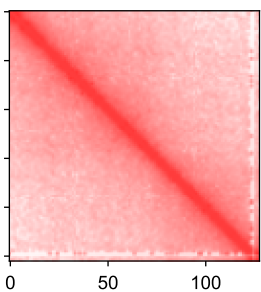

C.Origami on GM12878 (de novo)

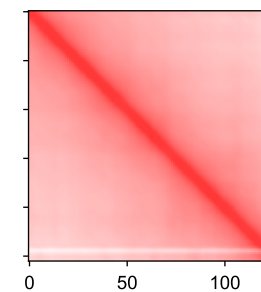

GM12878 Hi-C

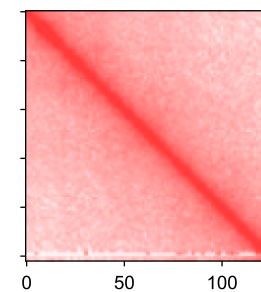

Supplement: Supplementary file 3 — Cell-type-specific predictions. [file 41587_2022_1612_MOESM3_ESM.zip › Cell type-specific predictions/chrX_55000000.pdf]

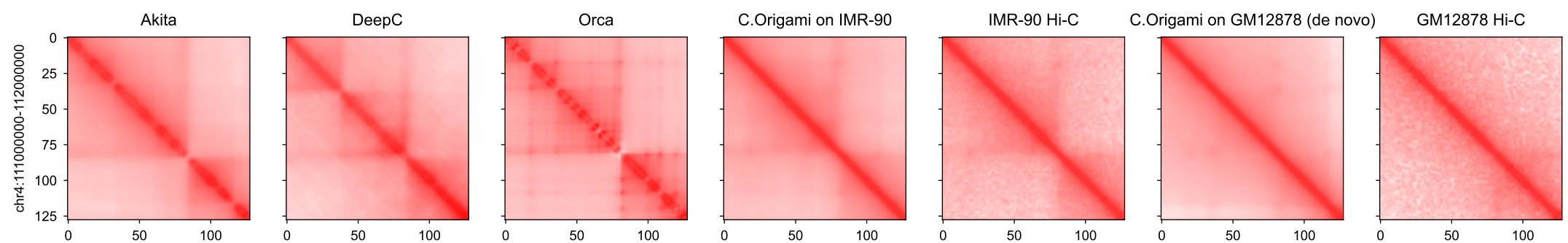

Supplement: Supplementary file 3 — Cell-type-specific predictions. [file 41587_2022_1612_MOESM3_ESM.zip › Cell type-specific predictions/chr4_111500000.pdf]

chr8:79500000-80500000

Akita

DeepC

Orca

C.Origami on IMR-90

IMR-90 Hi-C

C.Origami on GM12878 (de novo)

GM12878 Hi-C

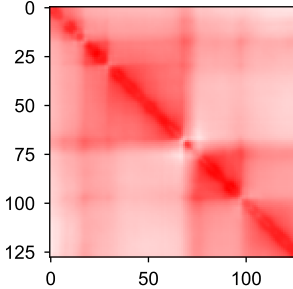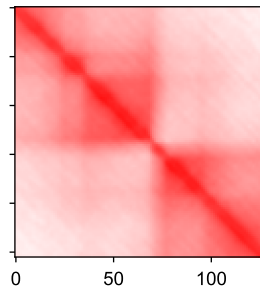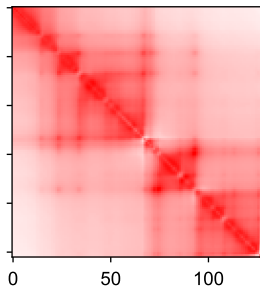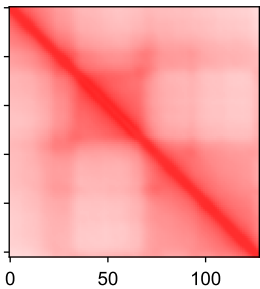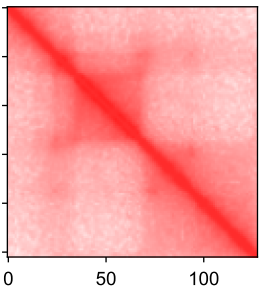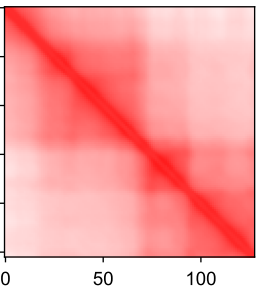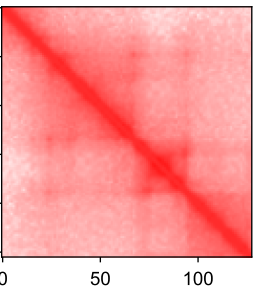

Supplement: Supplementary file 3 — Cell-type-specific predictions. [file 41587_2022_1612_MOESM3_ESM.zip › Cell type-specific predictions/chr8_80000000.pdf]

chr8:48500000-49500000

Akita

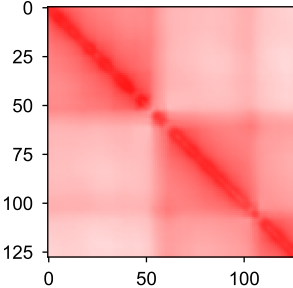

DeepC

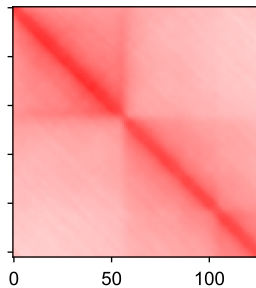

Orca

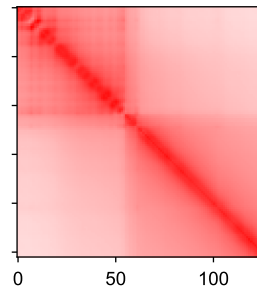

C.Origami on IMR-90

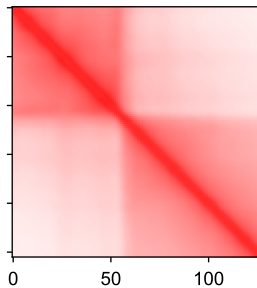

IMR-90 Hi-C

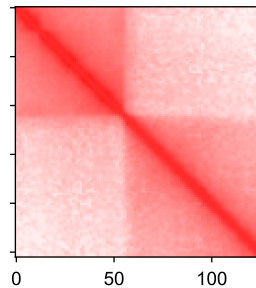

C.Origami on GM12878 (de novo)

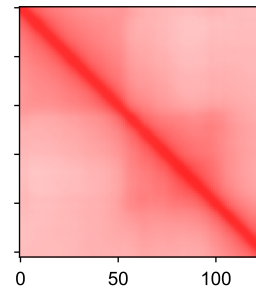

GM12878 Hi-C

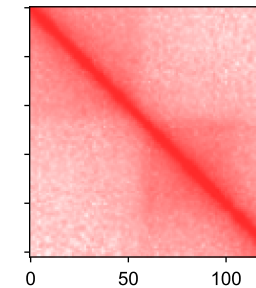

Supplement: Supplementary file 3 — Cell-type-specific predictions. [file 41587_2022_1612_MOESM3_ESM.zip › Cell type-specific predictions/chr8_49000000.pdf]

chr21:26000000-27000000

Akita

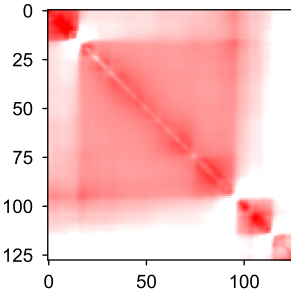

DeepC

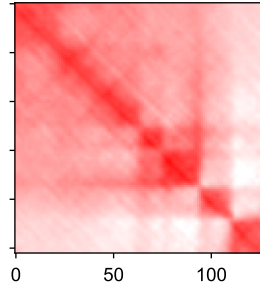

Orca

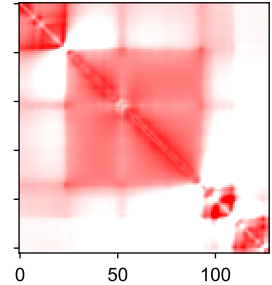

C.Origami on IMR-90

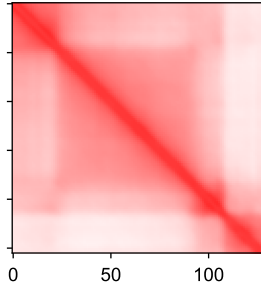

IMR-90 Hi-C

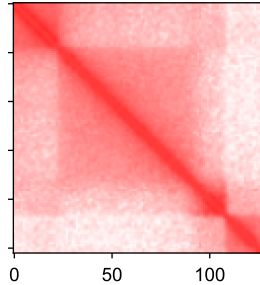

C.Origami on GM12878 (de novo)

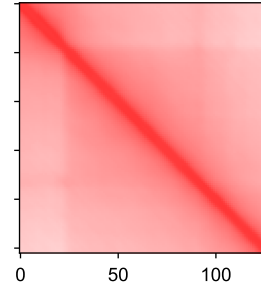

GM12878 Hi-C

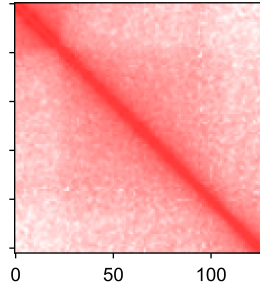

Supplement: Supplementary file 3 — Cell-type-specific predictions. [file 41587_2022_1612_MOESM3_ESM.zip › Cell type-specific predictions/chr21_26500000.pdf]

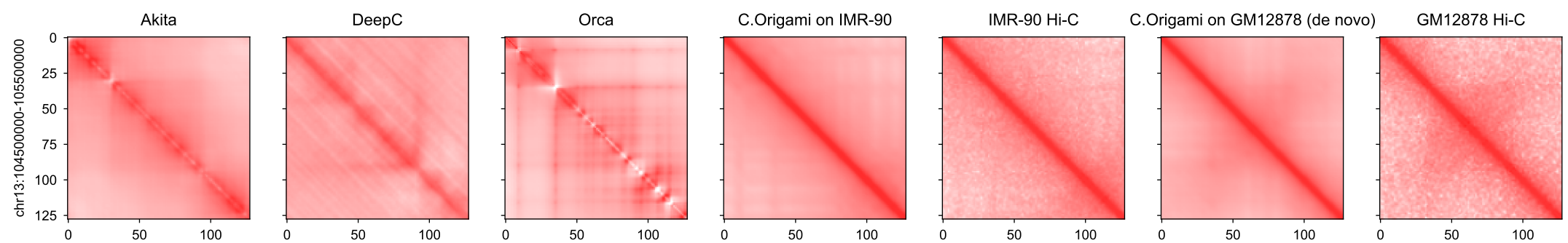

Supplement: Supplementary file 3 — Cell-type-specific predictions. [file 41587_2022_1612_MOESM3_ESM.zip › Cell type-specific predictions/chr13_105000000.pdf]

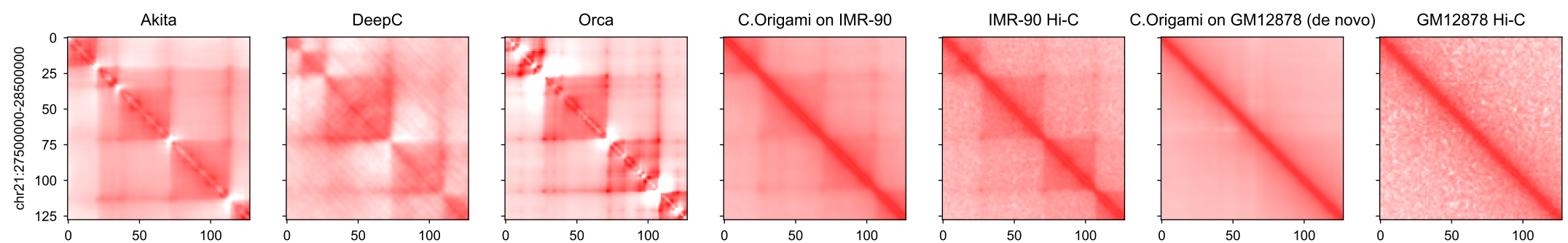

Supplement: Supplementary file 3 — Cell-type-specific predictions. [file 41587_2022_1612_MOESM3_ESM.zip › Cell type-specific predictions/chr21_28000000.pdf]

chr14:46000000-47000000

Akita

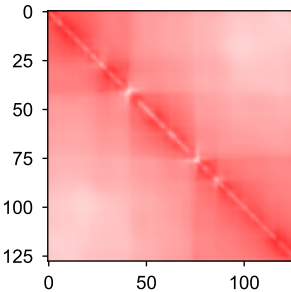

DeepC

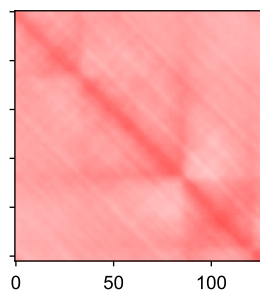

Orca

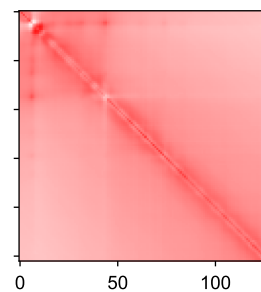

C.Origami on IMR-90

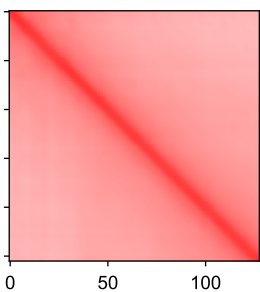

IMR-90 Hi-C

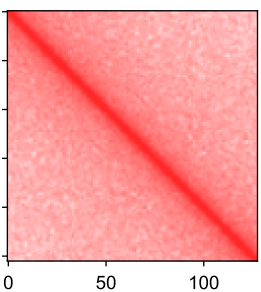

C.Origami on GM12878 (de novo)

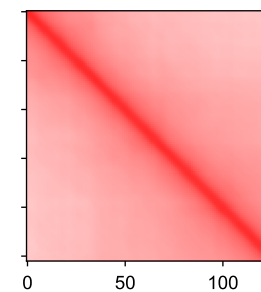

GM12878 Hi-C

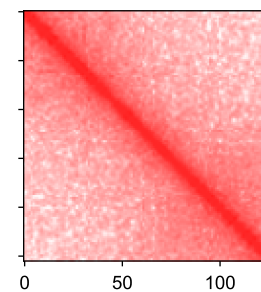

Supplement: Supplementary file 3 — Cell-type-specific predictions. [file 41587_2022_1612_MOESM3_ESM.zip › Cell type-specific predictions/chr14_46500000.pdf]

chr3:69500000-70500000

Akita

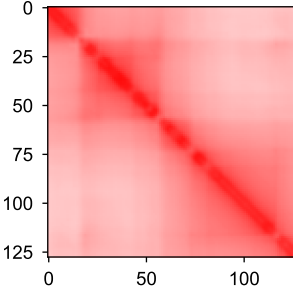

DeepC

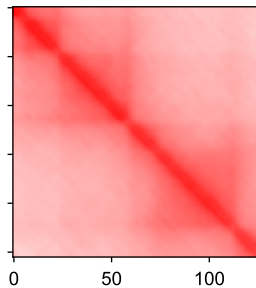

Orca

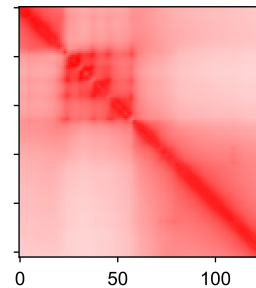

C.Origami on IMR-90

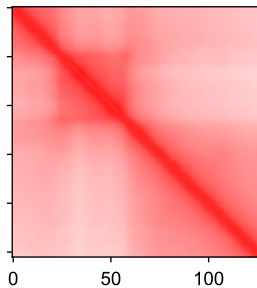

IMR-90 Hi-C

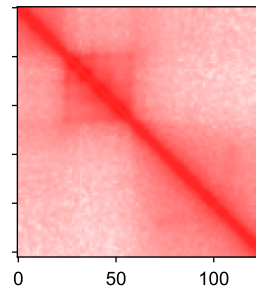

C.Origami on GM12878 (de novo)

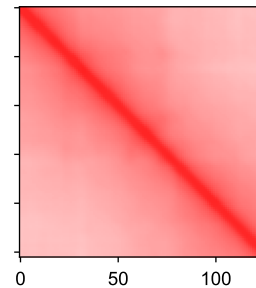

GM12878 Hi-C

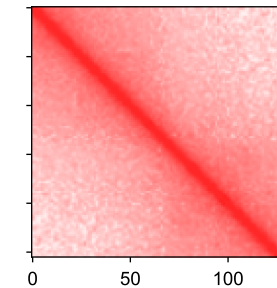

Supplement: Supplementary file 3 — Cell-type-specific predictions. [file 41587_2022_1612_MOESM3_ESM.zip › Cell type-specific predictions/chr3_70000000.pdf]

chr8:11500000-12500000

Akita

DeepC

Orca

C.Origami on IMR-90

IMR-90 Hi-C

C.Origami on GM12878 (de novo)

GM12878 Hi-C

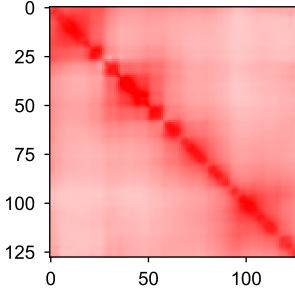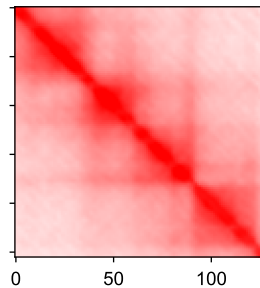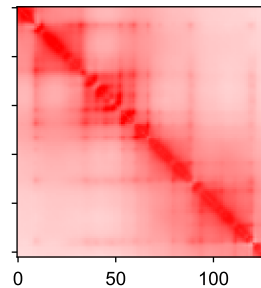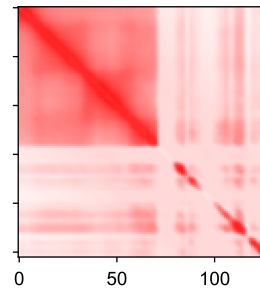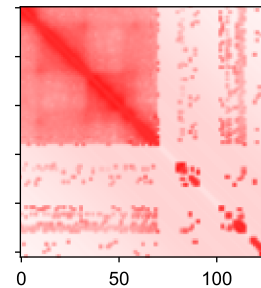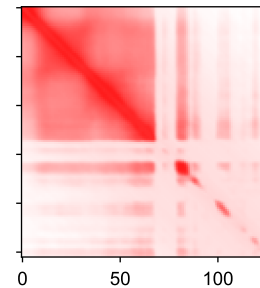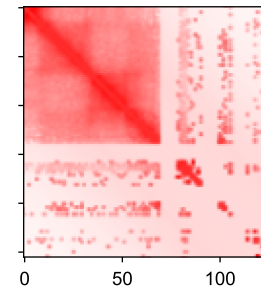

Supplement: Supplementary file 3 — Cell-type-specific predictions. [file 41587_2022_1612_MOESM3_ESM.zip › Cell type-specific predictions/chr8_12000000.pdf]

chr10:45000000-46000000

Akita

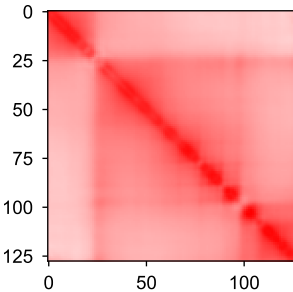

DeepC

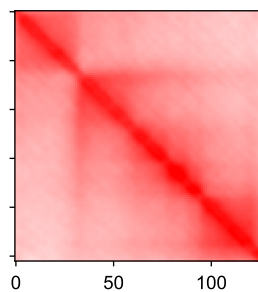

Orca

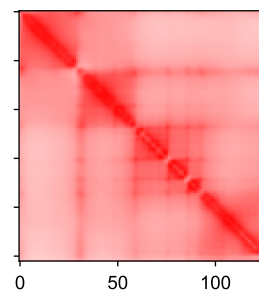

C.Origami on IMR-90

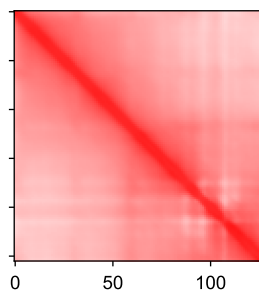

IMR-90 Hi-C

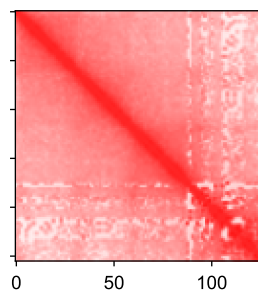

C.Origami on GM12878 (de novo)

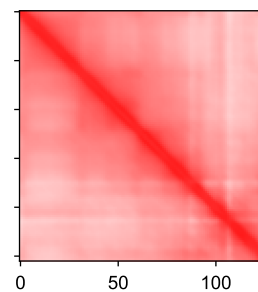

GM12878 Hi-C

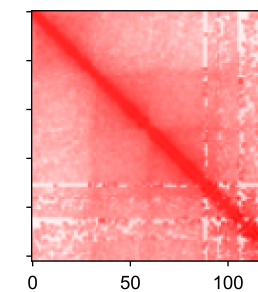

Supplement: Supplementary file 3 — Cell-type-specific predictions. [file 41587_2022_1612_MOESM3_ESM.zip › Cell type-specific predictions/chr10_45500000.pdf]

chr5:23000000-24000000

Akita

DeepC

Orca

C.Origami on IMR-90

IMR-90 Hi-C

C.Origami on GM12878 (de novo)

GM12878 Hi-C

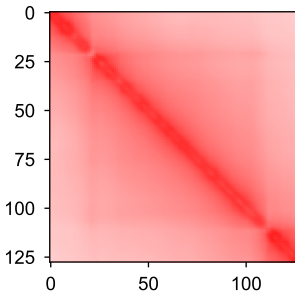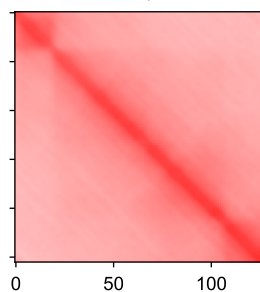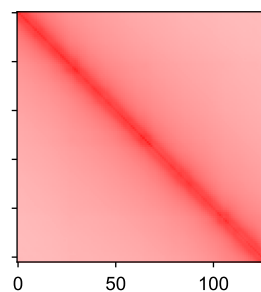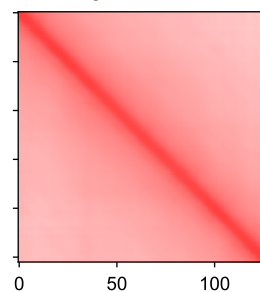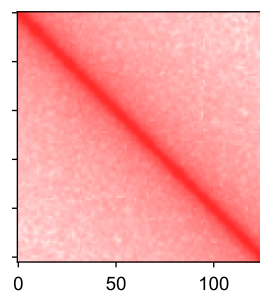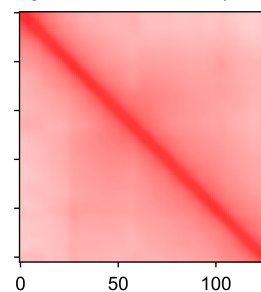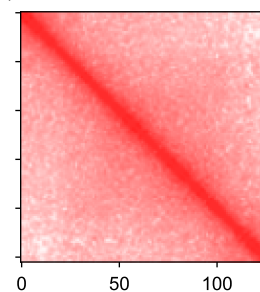

Supplement: Supplementary file 3 — Cell-type-specific predictions. [file 41587_2022_1612_MOESM3_ESM.zip › Cell type-specific predictions/chr5_23500000.pdf]

chr6:60500000-61500000

Akita

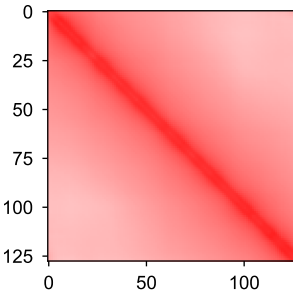

DeepC

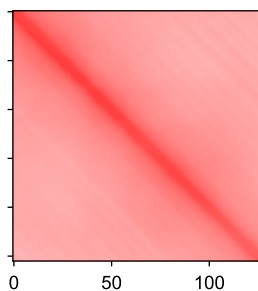

Orca

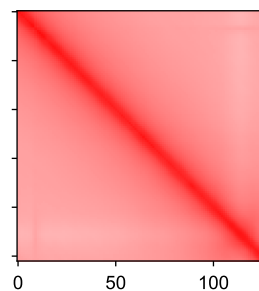

C.Origami on IMR-90

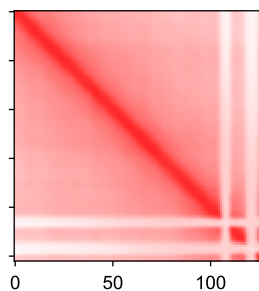

IMR-90 Hi-C

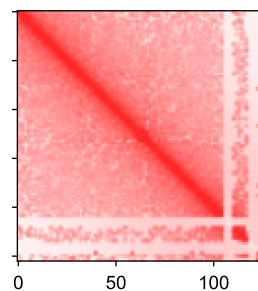

C.Origami on GM12878 (de novo)

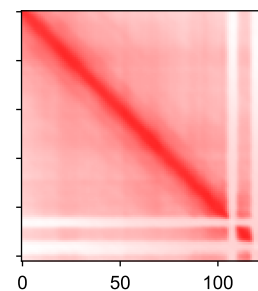

GM12878 Hi-C

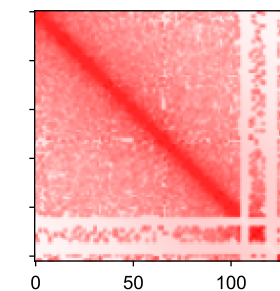

Supplement: Supplementary file 3 — Cell-type-specific predictions. [file 41587_2022_1612_MOESM3_ESM.zip › Cell type-specific predictions/chr6_61000000.pdf]

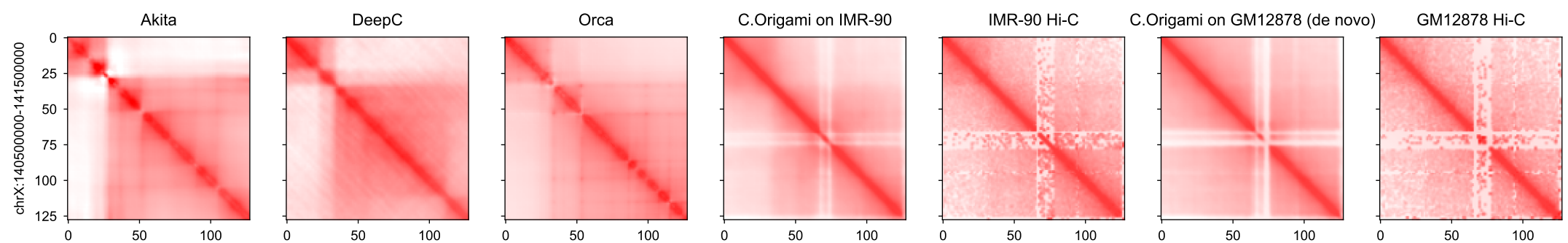

Supplement: Supplementary file 3 — Cell-type-specific predictions. [file 41587_2022_1612_MOESM3_ESM.zip › Cell type-specific predictions/chrX_141000000.pdf]

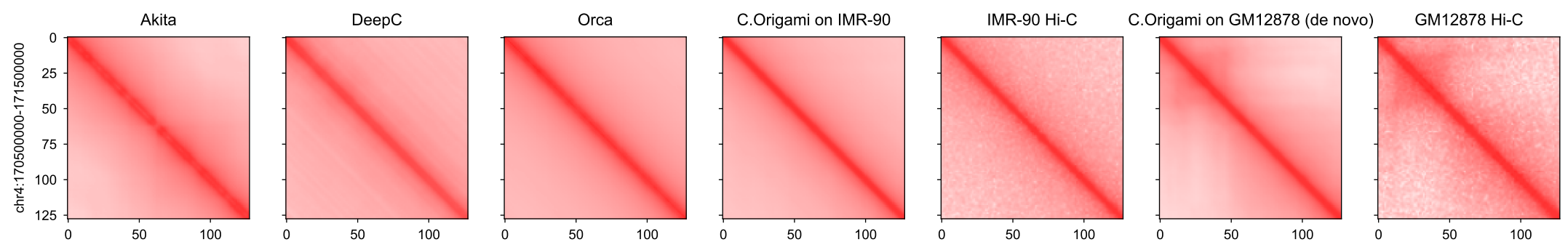

Supplement: Supplementary file 3 — Cell-type-specific predictions. [file 41587_2022_1612_MOESM3_ESM.zip › Cell type-specific predictions/chr4_171000000.pdf]

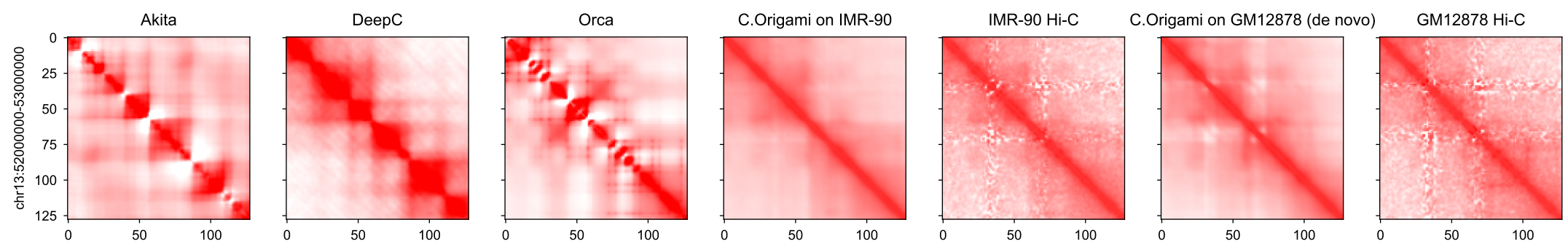

Supplement: Supplementary file 3 — Cell-type-specific predictions. [file 41587_2022_1612_MOESM3_ESM.zip › Cell type-specific predictions/chr13_52500000.pdf]

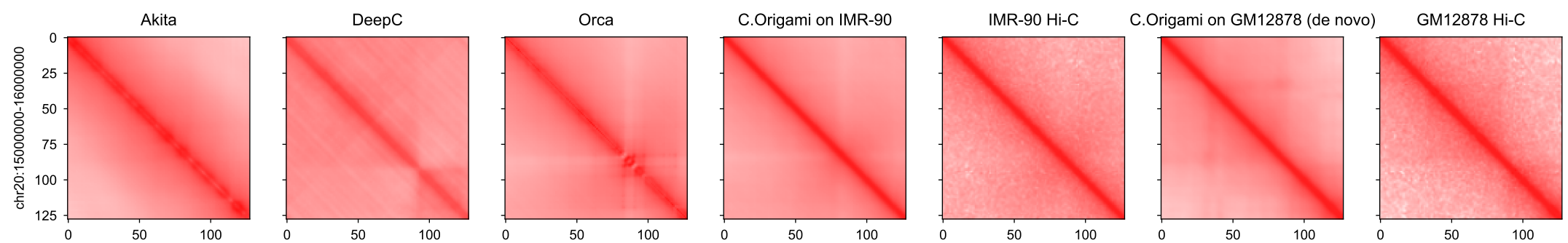

Supplement: Supplementary file 3 — Cell-type-specific predictions. [file 41587_2022_1612_MOESM3_ESM.zip › Cell type-specific predictions/chr20_15500000.pdf]

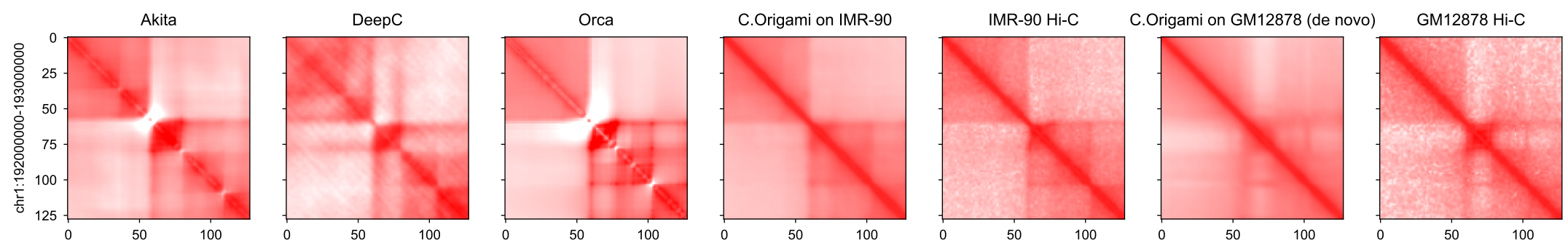

Supplement: Supplementary file 3 — Cell-type-specific predictions. [file 41587_2022_1612_MOESM3_ESM.zip › Cell type-specific predictions/chr1_192500000.pdf]

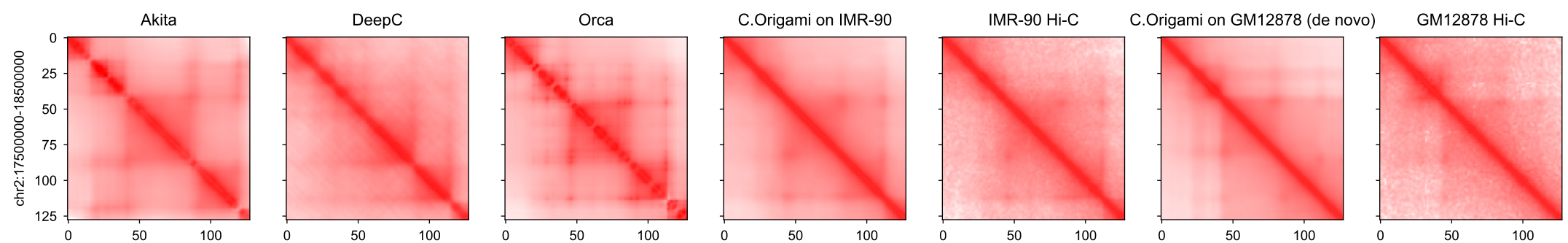

Supplement: Supplementary file 3 — Cell-type-specific predictions. [file 41587_2022_1612_MOESM3_ESM.zip › Cell type-specific predictions/chr2_18000000.pdf]

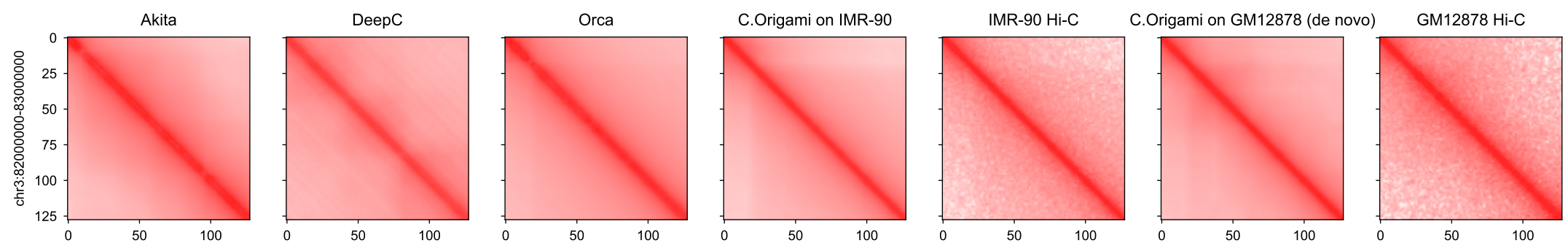

Supplement: Supplementary file 3 — Cell-type-specific predictions. [file 41587_2022_1612_MOESM3_ESM.zip › Cell type-specific predictions/chr3_82500000.pdf]

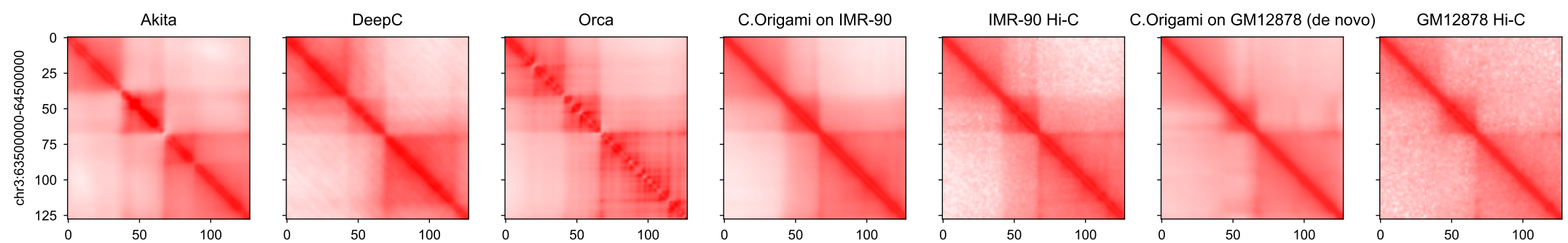

Supplement: Supplementary file 3 — Cell-type-specific predictions. [file 41587_2022_1612_MOESM3_ESM.zip › Cell type-specific predictions/chr3_64000000.pdf]

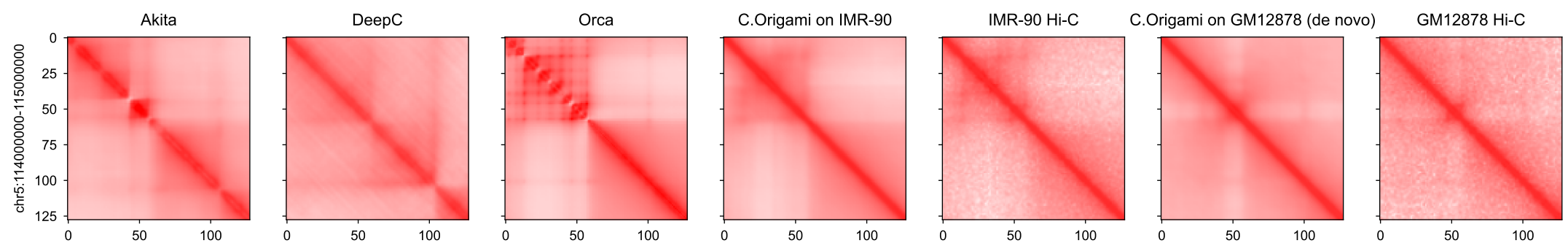

Supplement: Supplementary file 3 — Cell-type-specific predictions. [file 41587_2022_1612_MOESM3_ESM.zip › Cell type-specific predictions/chr5_114500000.pdf]

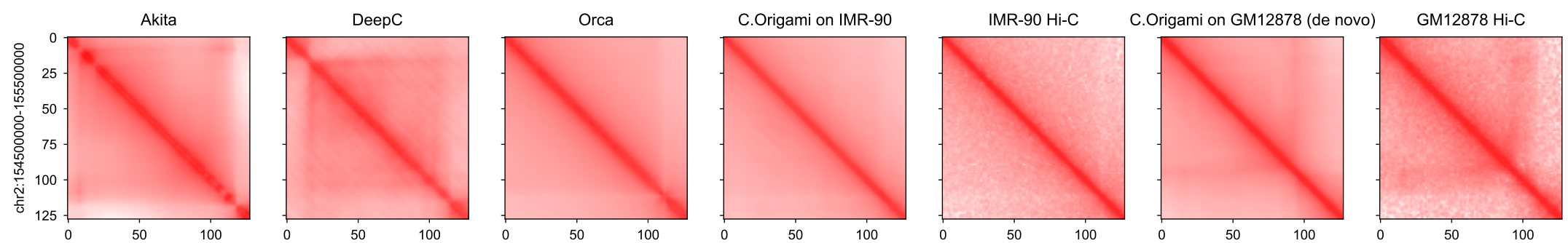

Supplement: Supplementary file 3 — Cell-type-specific predictions. [file 41587_2022_1612_MOESM3_ESM.zip › Cell type-specific predictions/chr2_155000000.pdf]

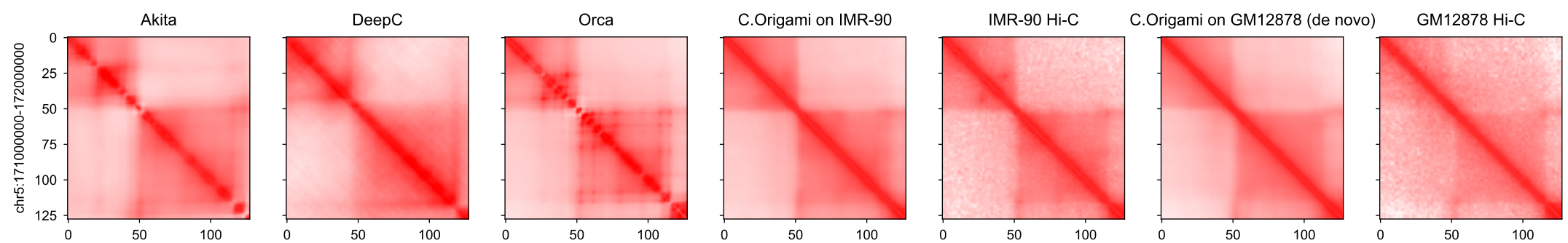

Supplement: Supplementary file 3 — Cell-type-specific predictions. [file 41587_2022_1612_MOESM3_ESM.zip › Cell type-specific predictions/chr5_171500000.pdf]

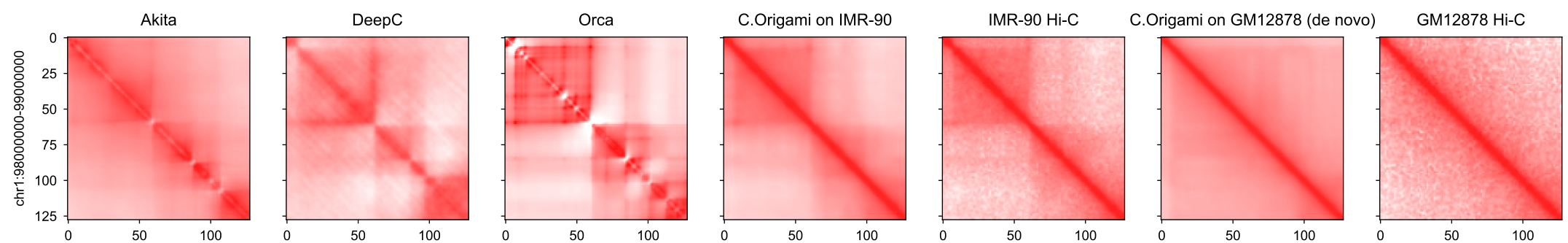

Supplement: Supplementary file 3 — Cell-type-specific predictions. [file 41587_2022_1612_MOESM3_ESM.zip › Cell type-specific predictions/chr1_98500000.pdf]

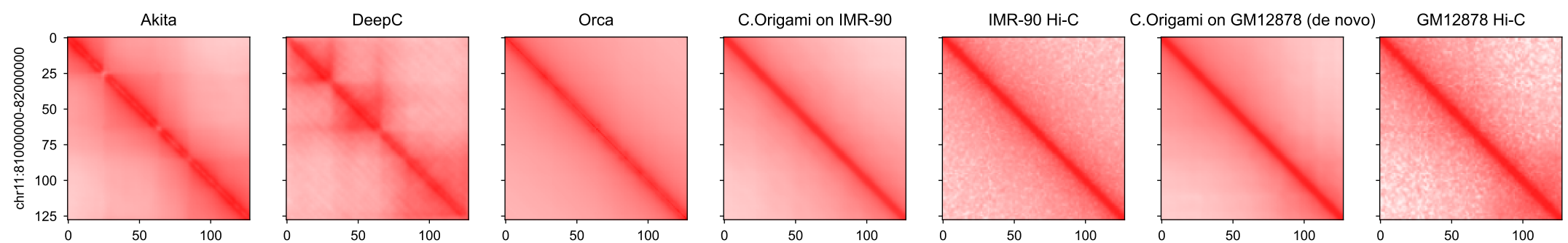

Supplement: Supplementary file 3 — Cell-type-specific predictions. [file 41587_2022_1612_MOESM3_ESM.zip › Cell type-specific predictions/chr11_81500000.pdf]

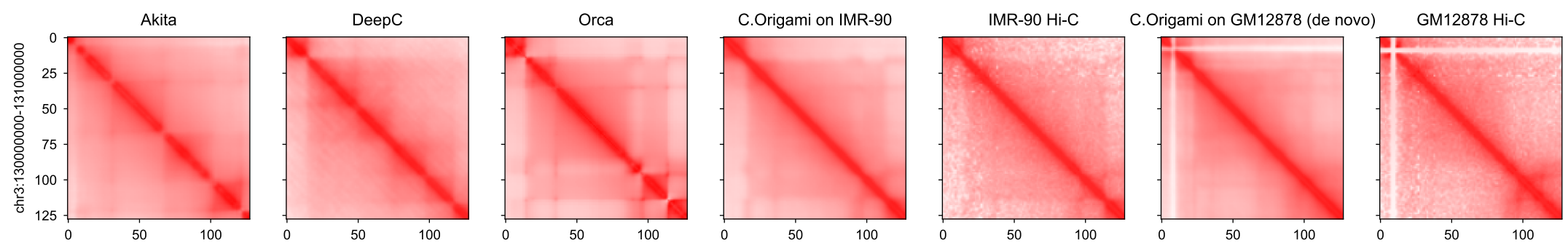

Supplement: Supplementary file 3 — Cell-type-specific predictions. [file 41587_2022_1612_MOESM3_ESM.zip › Cell type-specific predictions/chr3_130500000.pdf]

chr4:113500000-114500000

Akita

DeepC

Orca

C.Origami on IMR-90

IMR-90 Hi-C

C.Origami on GM12878 (de novo)

GM12878 Hi-C

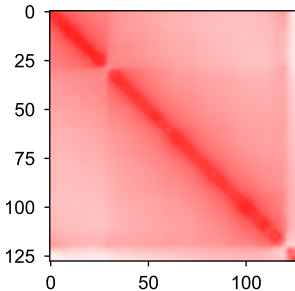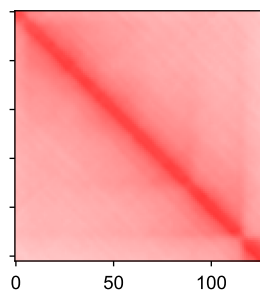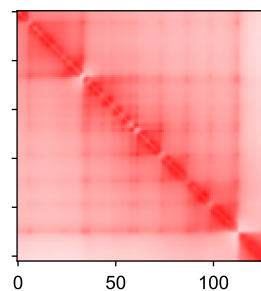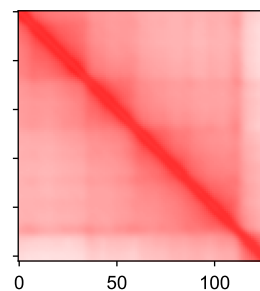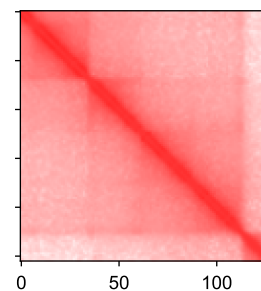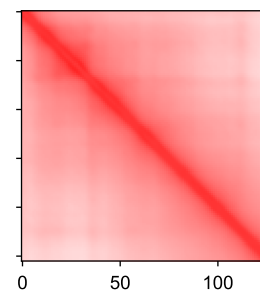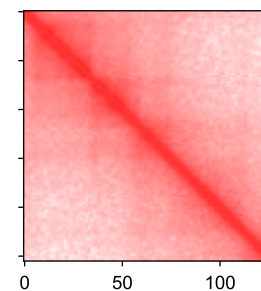

Supplement: Supplementary file 3 — Cell-type-specific predictions. [file 41587_2022_1612_MOESM3_ESM.zip › Cell type-specific predictions/chr4_114000000.pdf]

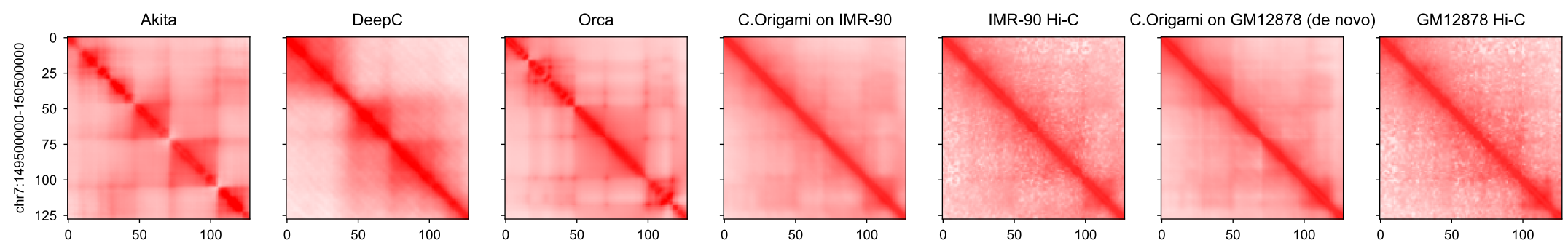

Supplement: Supplementary file 3 — Cell-type-specific predictions. [file 41587_2022_1612_MOESM3_ESM.zip › Cell type-specific predictions/chr7_150000000.pdf]

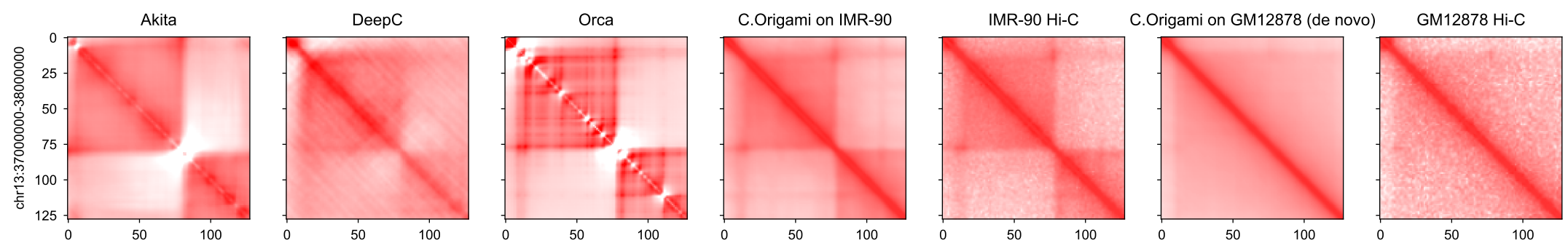

Supplement: Supplementary file 3 — Cell-type-specific predictions. [file 41587_2022_1612_MOESM3_ESM.zip › Cell type-specific predictions/chr13_37500000.pdf]

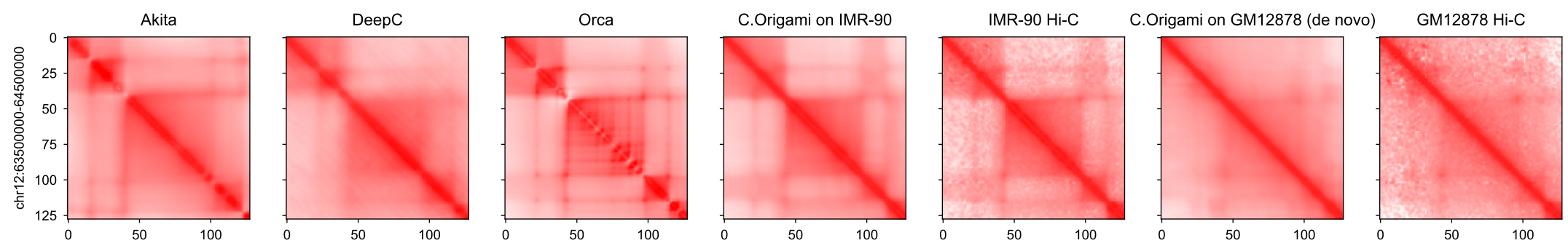

Supplement: Supplementary file 3 — Cell-type-specific predictions. [file 41587_2022_1612_MOESM3_ESM.zip › Cell type-specific predictions/chr12_64000000.pdf]

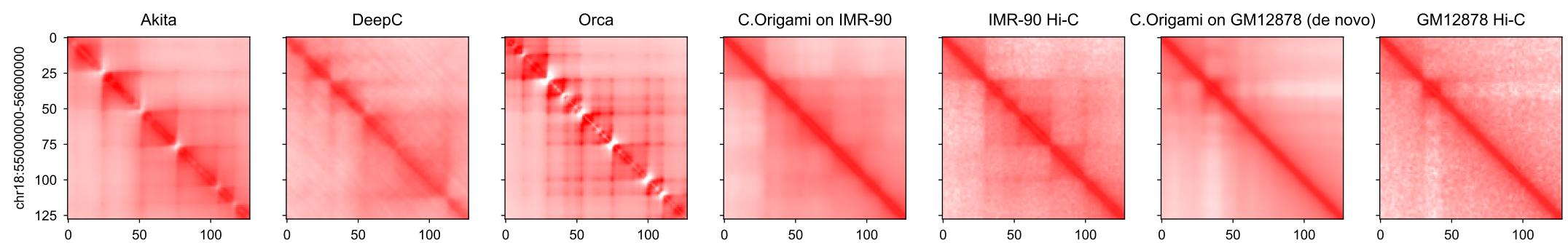

Supplement: Supplementary file 3 — Cell-type-specific predictions. [file 41587_2022_1612_MOESM3_ESM.zip › Cell type-specific predictions/chr18_55500000.pdf]

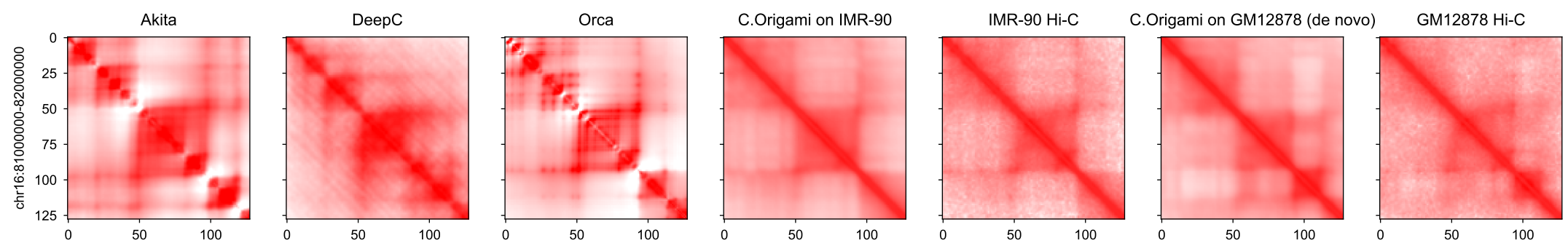

Supplement: Supplementary file 3 — Cell-type-specific predictions. [file 41587_2022_1612_MOESM3_ESM.zip › Cell type-specific predictions/chr16_81500000.pdf]
