# Supplementary figures and images for: Cell-type-specific prediction of 3D chromatin organization enables high-throughput in silico genetic screening (part 1 of 2)
Source: Nat Biotechnol. 2023 Jan 9;41(8):1140–50. doi: 10.1038/s41587-022-01612-8 (PMC10329734; doi:10.1038/s41587-022-01612-8)

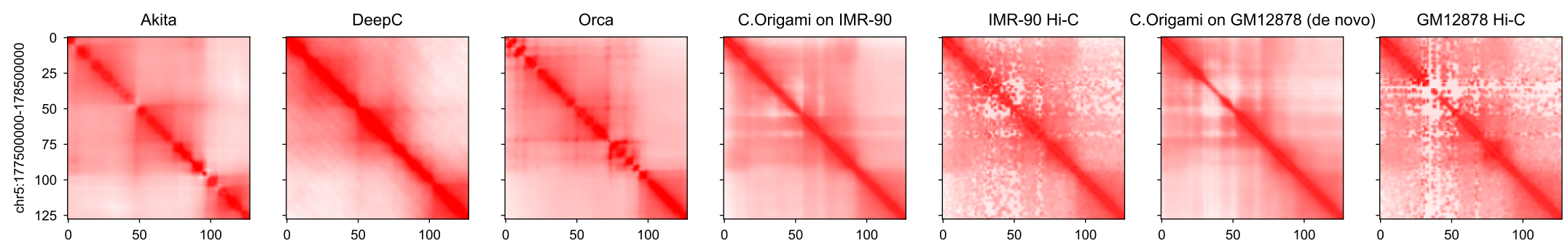

Supplement: Supplementary file 3 — Cell-type-specific predictions. [file 41587_2022_1612_MOESM3_ESM.zip › Cell type-specific predictions/chr5_178000000.pdf]

chr18:25500000-26500000

Akita

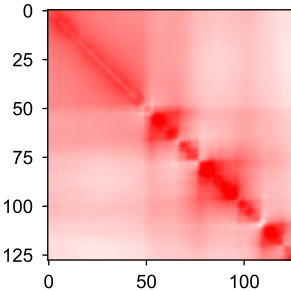

DeepC

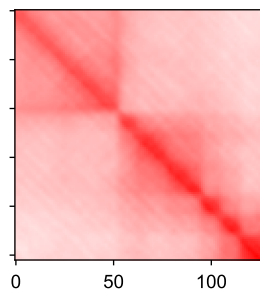

Orca

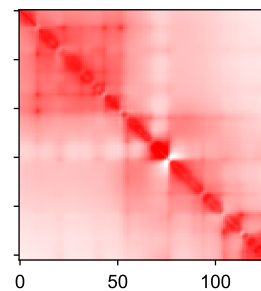

C.Origami on IMR-90

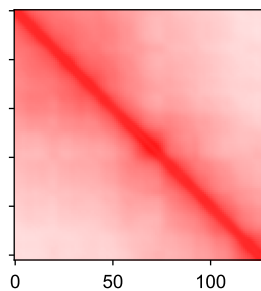

IMR-90 Hi-C

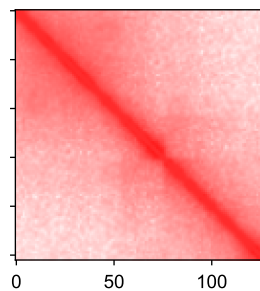

C.Origami on GM12878 (de novo)

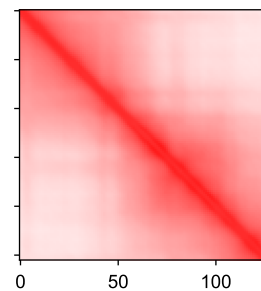

GM12878 Hi-C

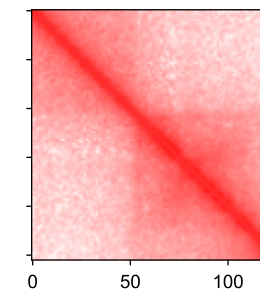

Supplement: Supplementary file 3 — Cell-type-specific predictions. [file 41587_2022_1612_MOESM3_ESM.zip › Cell type-specific predictions/chr18_26000000.pdf]

chrX:89500000-90500000

Akita

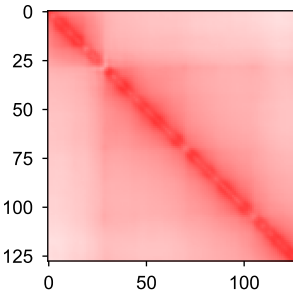

DeepC

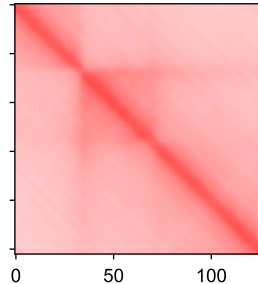

Orca

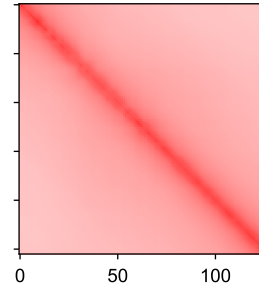

C.Origami on IMR-90

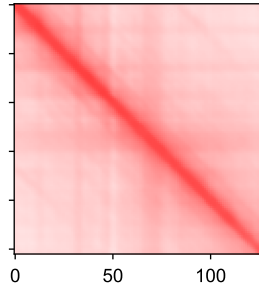

IMR-90 Hi-C

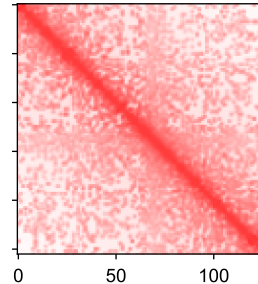

C.Origami on GM12878 (de novo)

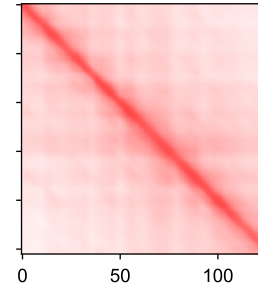

GM12878 Hi-C

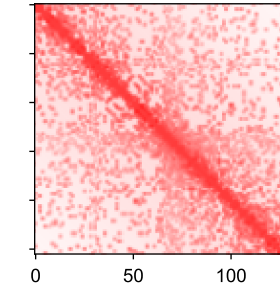

Supplement: Supplementary file 3 — Cell-type-specific predictions. [file 41587_2022_1612_MOESM3_ESM.zip › Cell type-specific predictions/chrX_90000000.pdf]

chr6:77000000-78000000

Akita

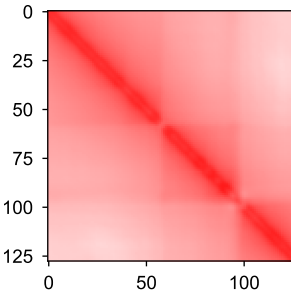

DeepC

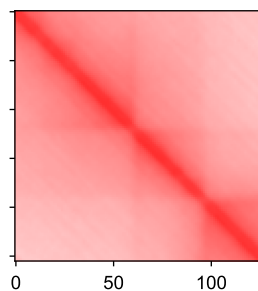

Orca

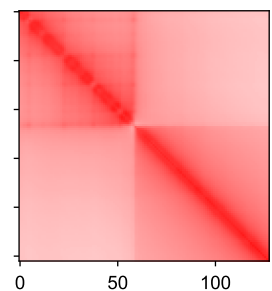

C.Origami on IMR-90

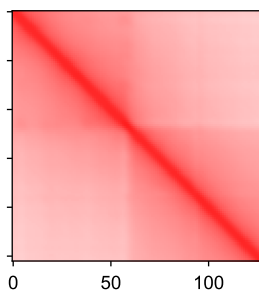

IMR-90 Hi-C

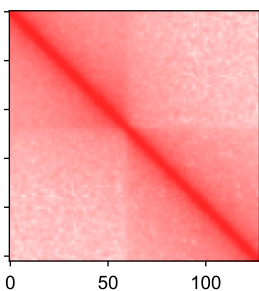

C.Origami on GM12878 (de novo)

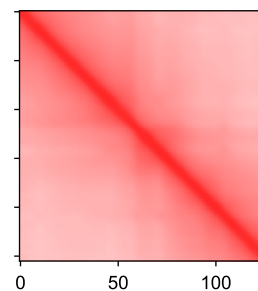

GM12878 Hi-C

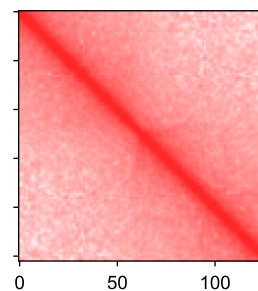

Supplement: Supplementary file 3 — Cell-type-specific predictions. [file 41587_2022_1612_MOESM3_ESM.zip › Cell type-specific predictions/chr6_77500000.pdf]

chr1:77000000-78000000

Akita

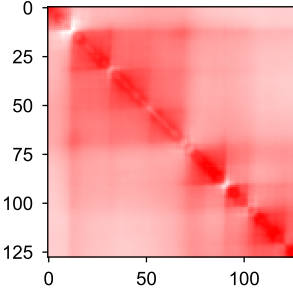

DeepC

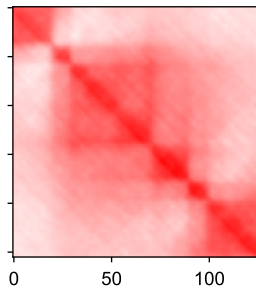

Orca

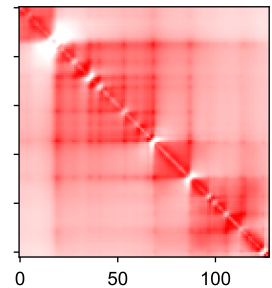

C.Origami on IMR-90

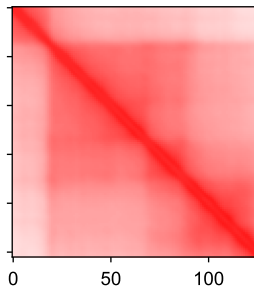

IMR-90 Hi-C

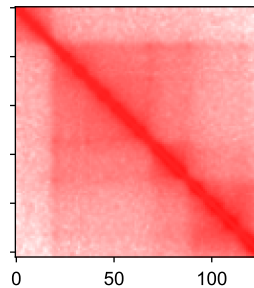

C.Origami on GM12878 (de novo)

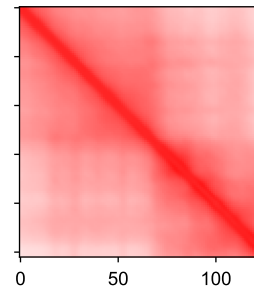

GM12878 Hi-C

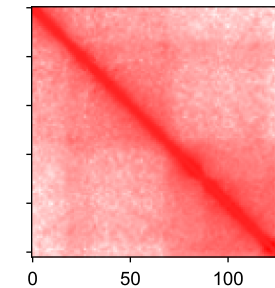

Supplement: Supplementary file 3 — Cell-type-specific predictions. [file 41587_2022_1612_MOESM3_ESM.zip › Cell type-specific predictions/chr1_77500000.pdf]

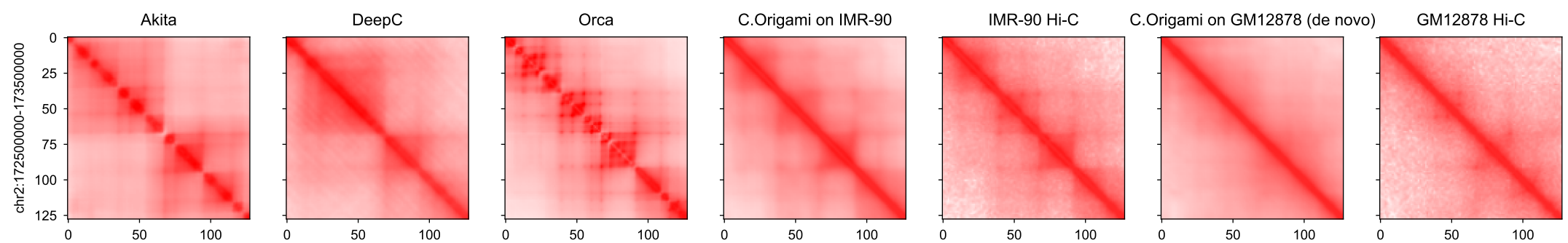

Supplement: Supplementary file 3 — Cell-type-specific predictions. [file 41587_2022_1612_MOESM3_ESM.zip › Cell type-specific predictions/chr2_173000000.pdf]

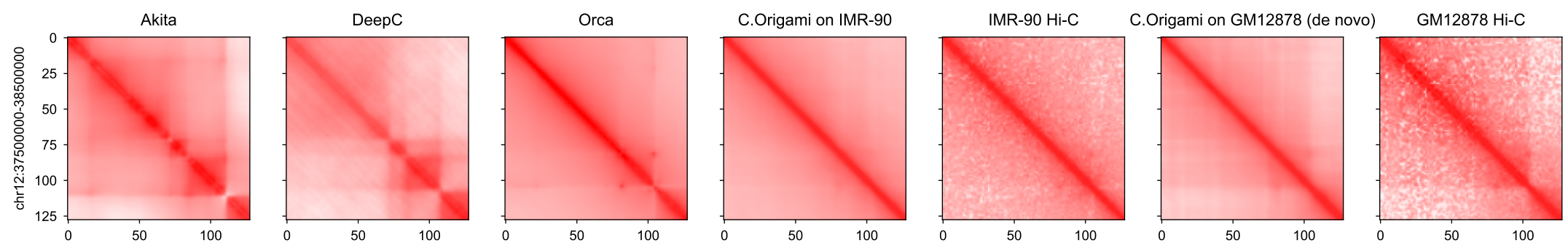

Supplement: Supplementary file 3 — Cell-type-specific predictions. [file 41587_2022_1612_MOESM3_ESM.zip › Cell type-specific predictions/chr12_38000000.pdf]

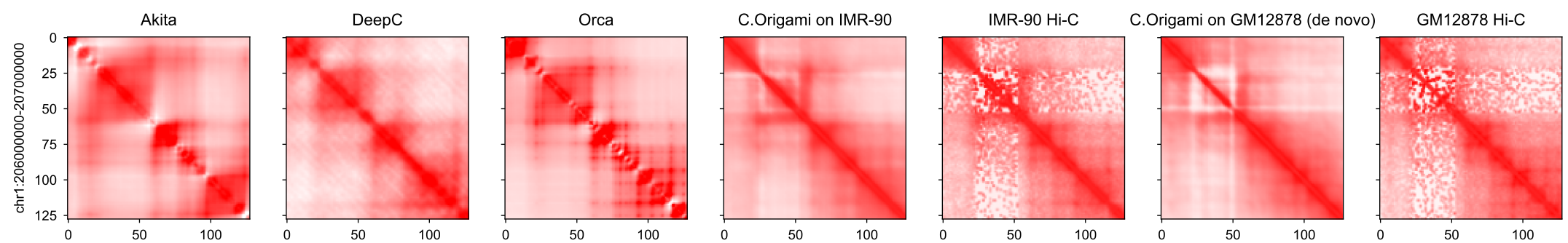

Supplement: Supplementary file 3 — Cell-type-specific predictions. [file 41587_2022_1612_MOESM3_ESM.zip › Cell type-specific predictions/chr1_206500000.pdf]

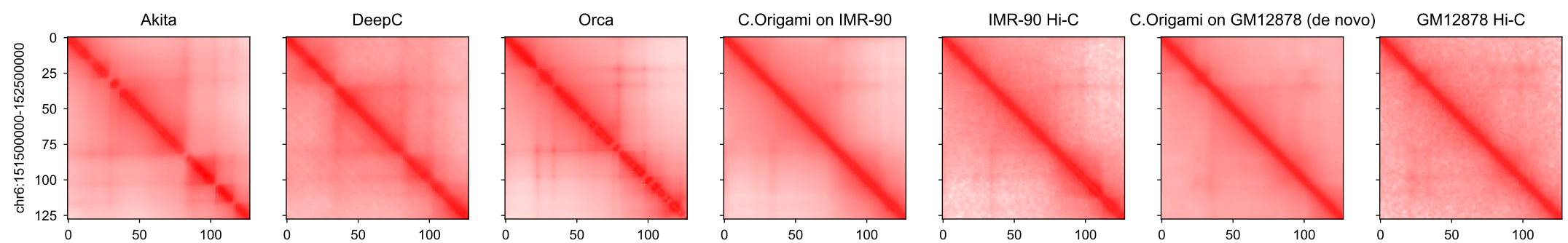

Supplement: Supplementary file 3 — Cell-type-specific predictions. [file 41587_2022_1612_MOESM3_ESM.zip › Cell type-specific predictions/chr6_152000000.pdf]

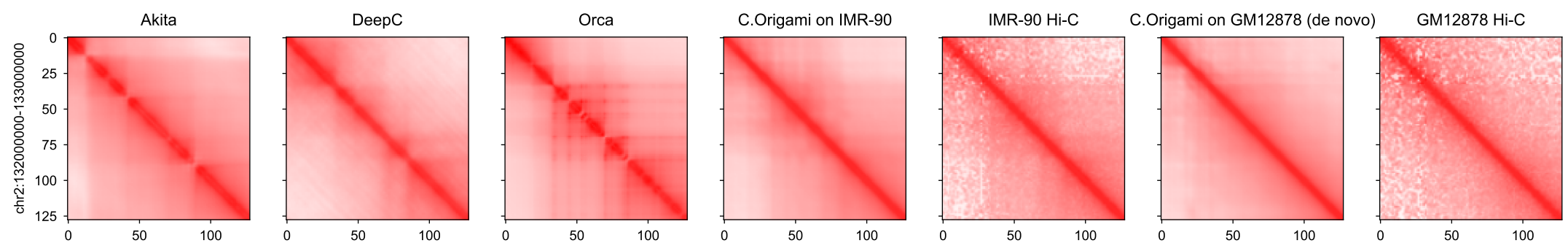

Supplement: Supplementary file 3 — Cell-type-specific predictions. [file 41587_2022_1612_MOESM3_ESM.zip › Cell type-specific predictions/chr2_132500000.pdf]

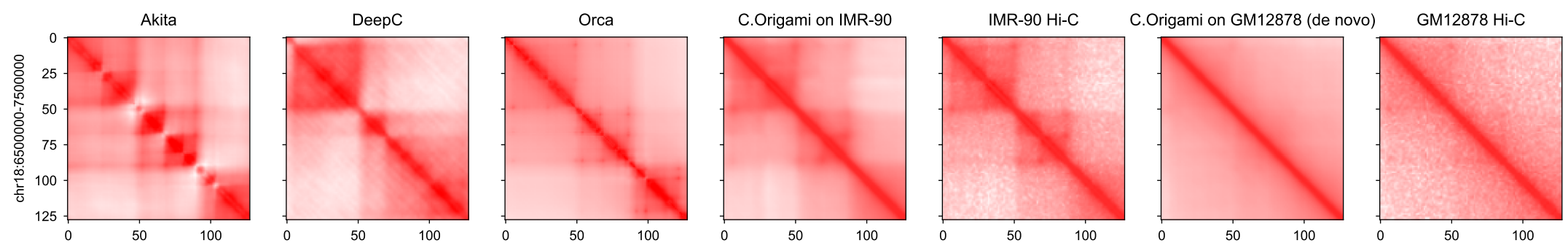

Supplement: Supplementary file 3 — Cell-type-specific predictions. [file 41587_2022_1612_MOESM3_ESM.zip › Cell type-specific predictions/chr18_7000000.pdf]

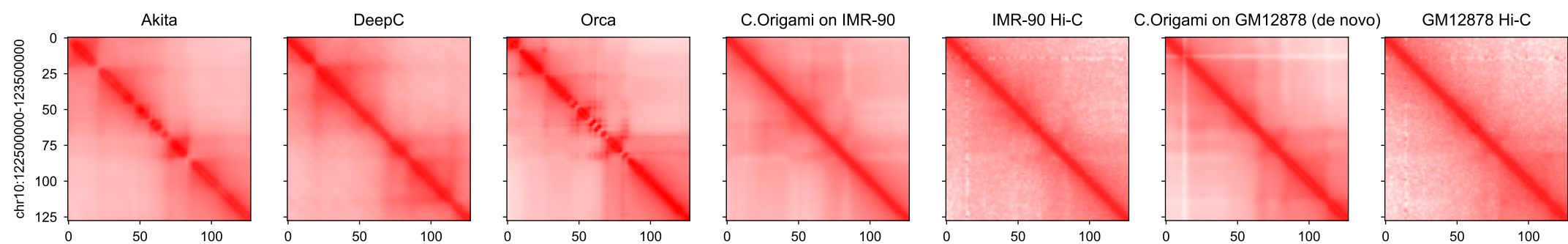

Supplement: Supplementary file 3 — Cell-type-specific predictions. [file 41587_2022_1612_MOESM3_ESM.zip › Cell type-specific predictions/chr10_123000000.pdf]

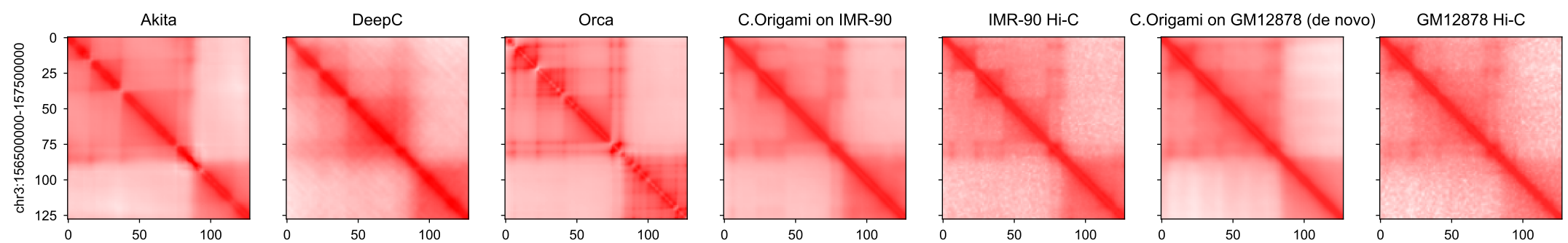

Supplement: Supplementary file 3 — Cell-type-specific predictions. [file 41587_2022_1612_MOESM3_ESM.zip › Cell type-specific predictions/chr3_157000000.pdf]

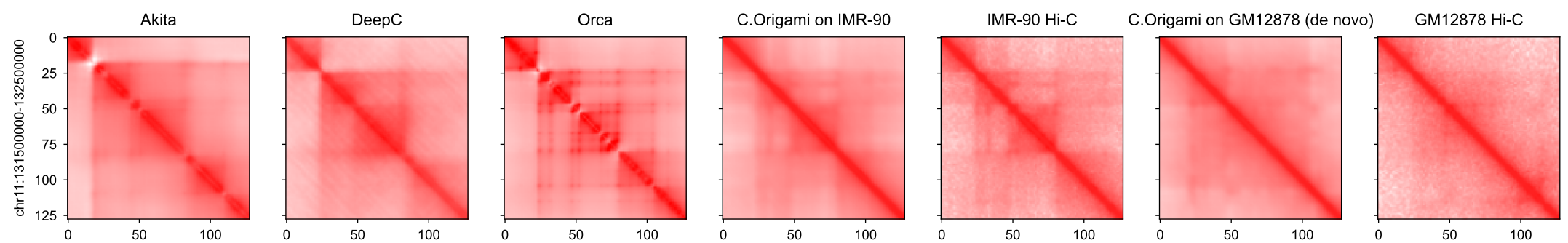

Supplement: Supplementary file 3 — Cell-type-specific predictions. [file 41587_2022_1612_MOESM3_ESM.zip › Cell type-specific predictions/chr11_132000000.pdf]

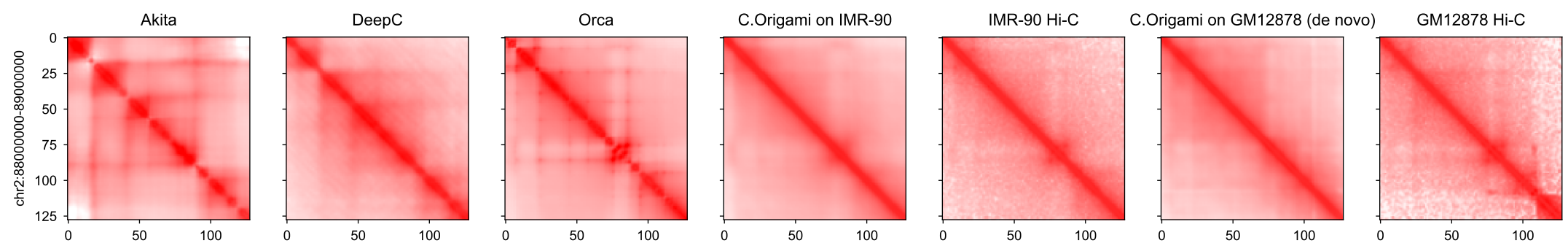

Supplement: Supplementary file 3 — Cell-type-specific predictions. [file 41587_2022_1612_MOESM3_ESM.zip › Cell type-specific predictions/chr2_88500000.pdf]

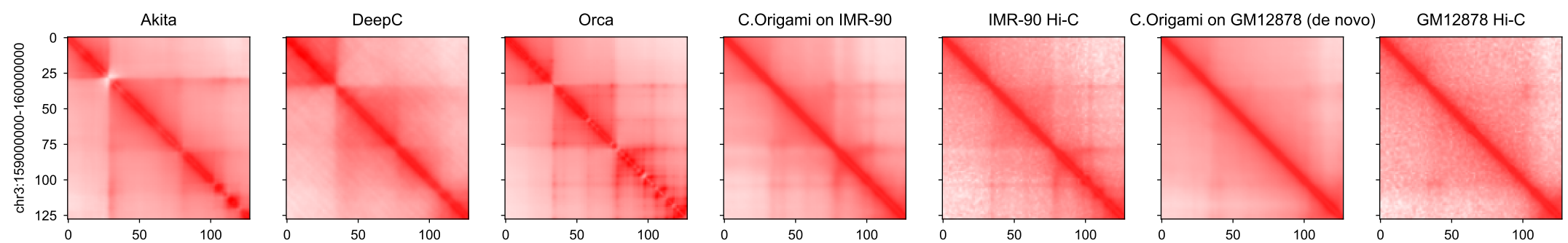

Supplement: Supplementary file 3 — Cell-type-specific predictions. [file 41587_2022_1612_MOESM3_ESM.zip › Cell type-specific predictions/chr3_159500000.pdf]

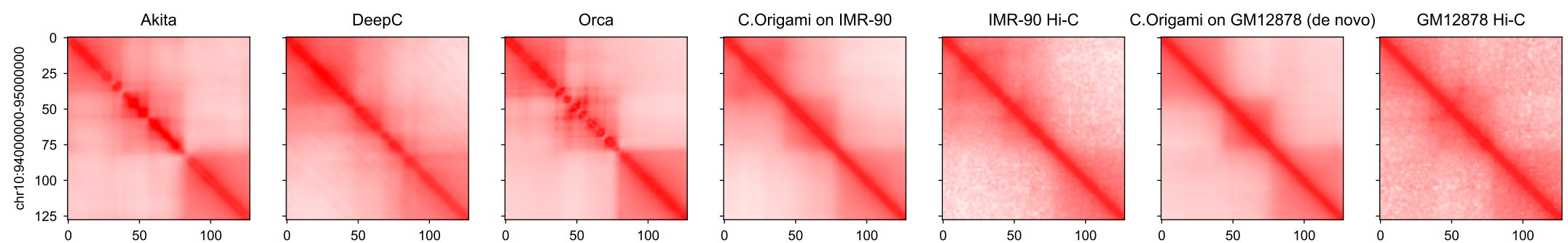

Supplement: Supplementary file 3 — Cell-type-specific predictions. [file 41587_2022_1612_MOESM3_ESM.zip › Cell type-specific predictions/chr10_94500000.pdf]

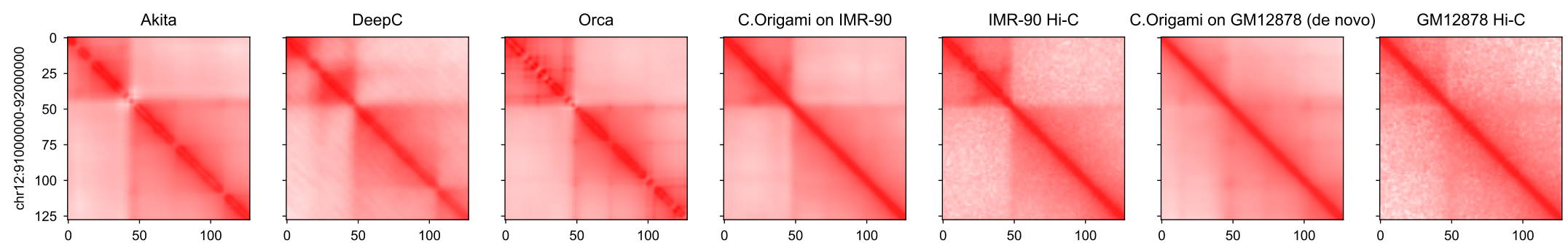

Supplement: Supplementary file 3 — Cell-type-specific predictions. [file 41587_2022_1612_MOESM3_ESM.zip › Cell type-specific predictions/chr12_91500000.pdf]

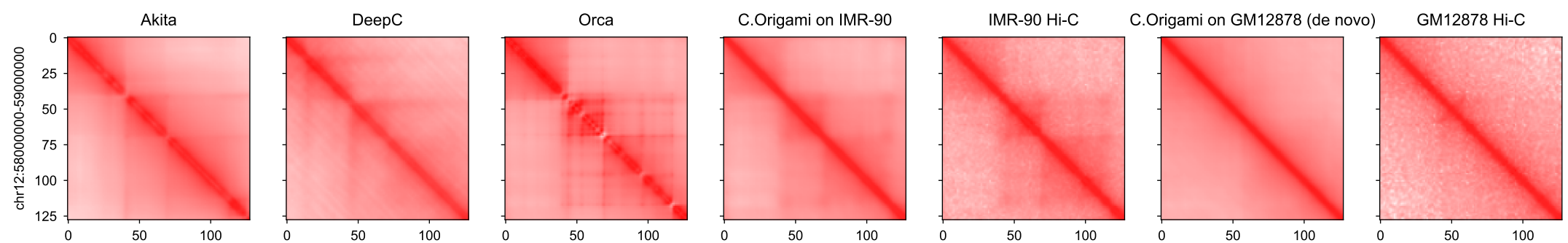

Supplement: Supplementary file 3 — Cell-type-specific predictions. [file 41587_2022_1612_MOESM3_ESM.zip › Cell type-specific predictions/chr12_58500000.pdf]

chr19:14500000-15500000

Akita

DeepC

Orca

C.Origami on IMR-90

IMR-90 Hi-C

C.Origami on GM12878 (de novo)

GM12878 Hi-C

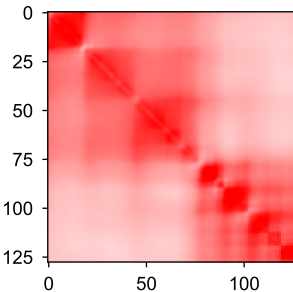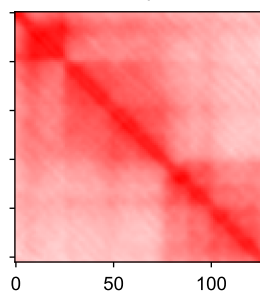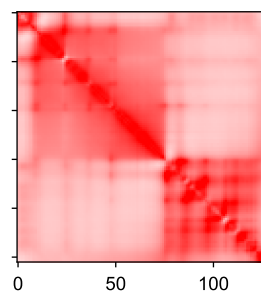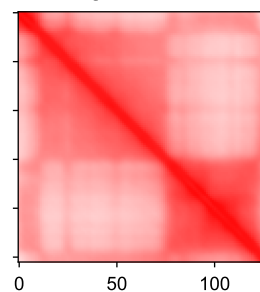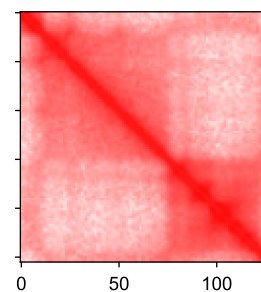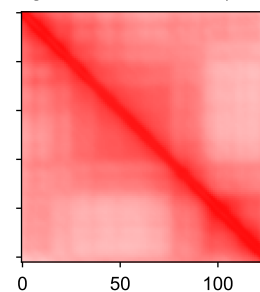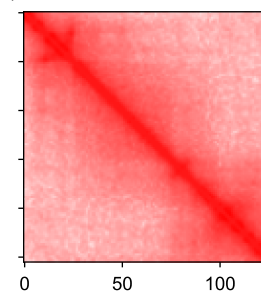

Supplement: Supplementary file 3 — Cell-type-specific predictions. [file 41587_2022_1612_MOESM3_ESM.zip › Cell type-specific predictions/chr19_15000000.pdf]

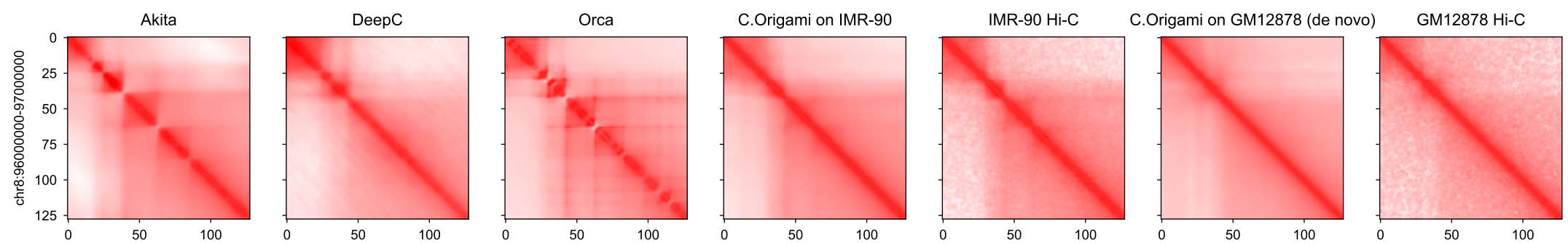

Supplement: Supplementary file 3 — Cell-type-specific predictions. [file 41587_2022_1612_MOESM3_ESM.zip › Cell type-specific predictions/chr8_96500000.pdf]

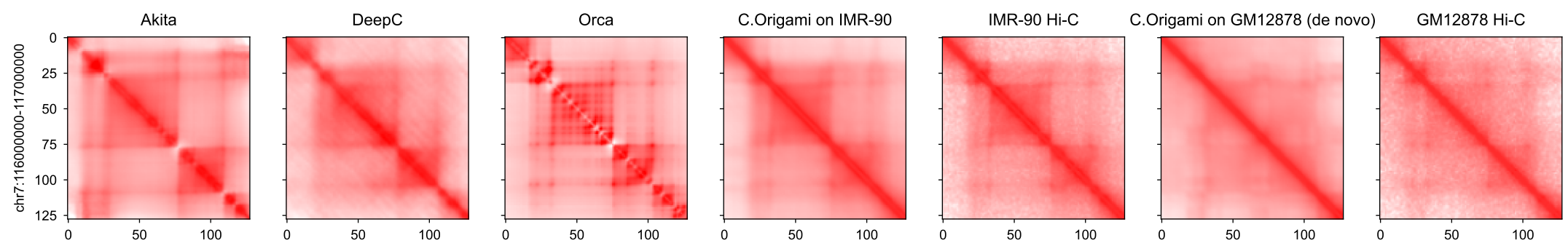

Supplement: Supplementary file 3 — Cell-type-specific predictions. [file 41587_2022_1612_MOESM3_ESM.zip › Cell type-specific predictions/chr7_116500000.pdf]

chr6:16500000-17500000

Akita

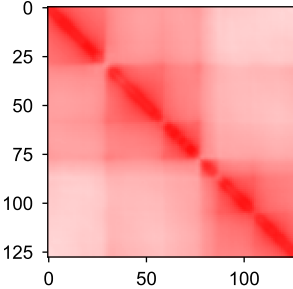

DeepC

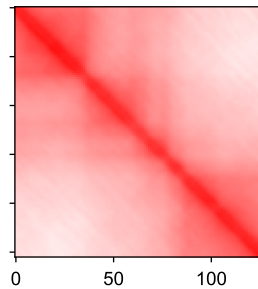

Orca

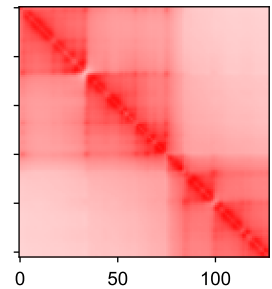

C.Origami on IMR-90

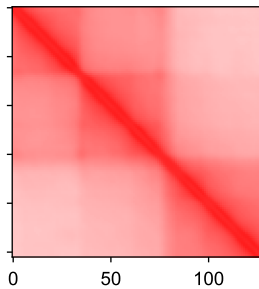

IMR-90 Hi-C

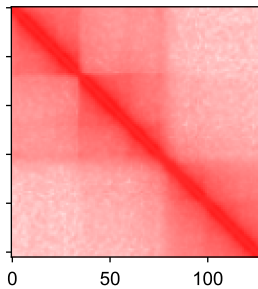

C.Origami on GM12878 (de novo)

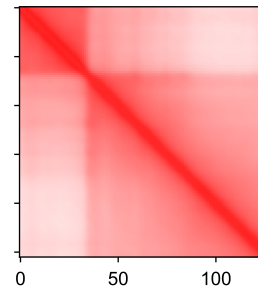

GM12878 Hi-C

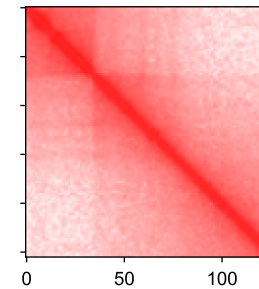

Supplement: Supplementary file 3 — Cell-type-specific predictions. [file 41587_2022_1612_MOESM3_ESM.zip › Cell type-specific predictions/chr6_17000000.pdf]

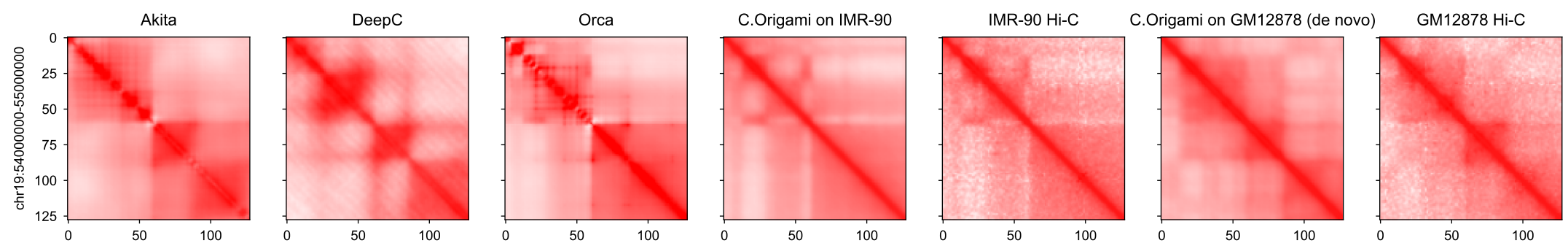

Supplement: Supplementary file 3 — Cell-type-specific predictions. [file 41587_2022_1612_MOESM3_ESM.zip › Cell type-specific predictions/chr19_54500000.pdf]

chr11:59500000-60500000

Akita

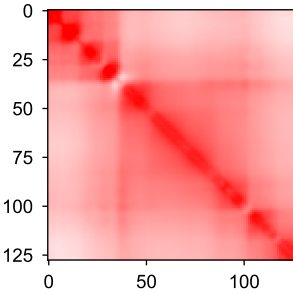

DeepC

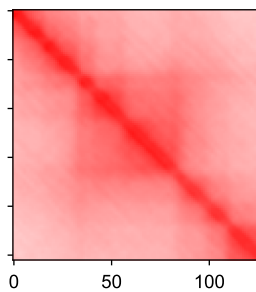

Orca

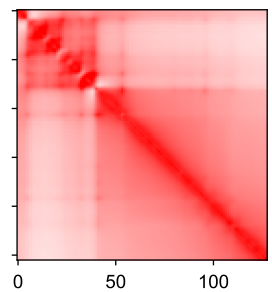

C.Origami on IMR-90

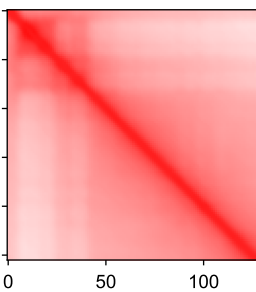

IMR-90 Hi-C

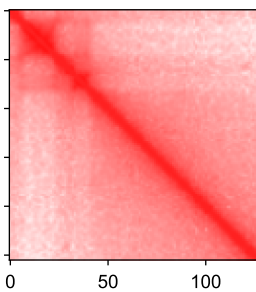

C.Origami on GM12878 (de novo)

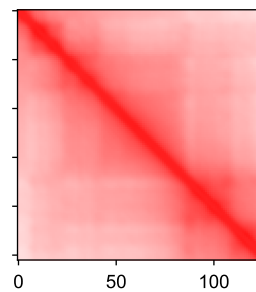

GM12878 Hi-C

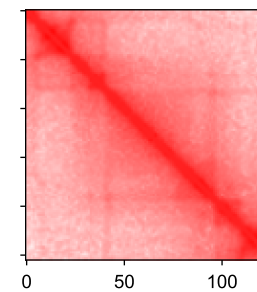

Supplement: Supplementary file 3 — Cell-type-specific predictions. [file 41587_2022_1612_MOESM3_ESM.zip › Cell type-specific predictions/chr11_60000000.pdf]

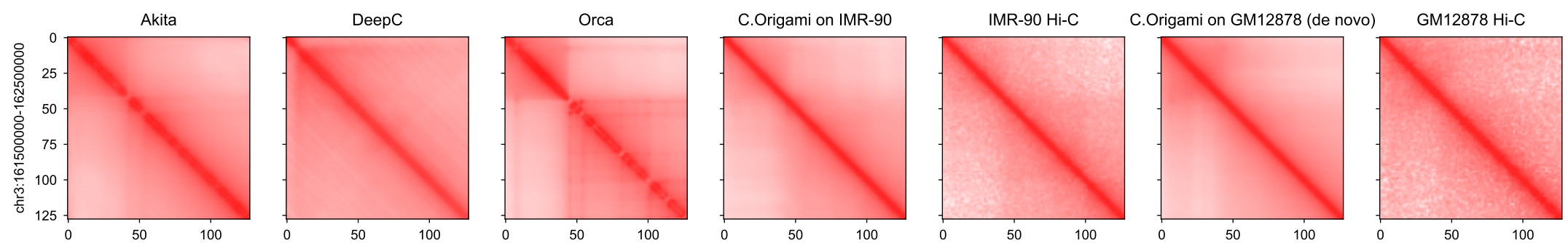

Supplement: Supplementary file 3 — Cell-type-specific predictions. [file 41587_2022_1612_MOESM3_ESM.zip › Cell type-specific predictions/chr3_162000000.pdf]

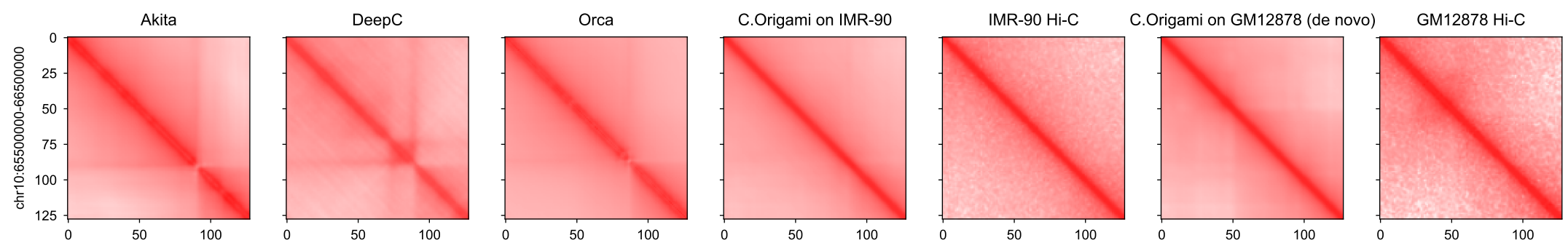

Supplement: Supplementary file 3 — Cell-type-specific predictions. [file 41587_2022_1612_MOESM3_ESM.zip › Cell type-specific predictions/chr10_66000000.pdf]

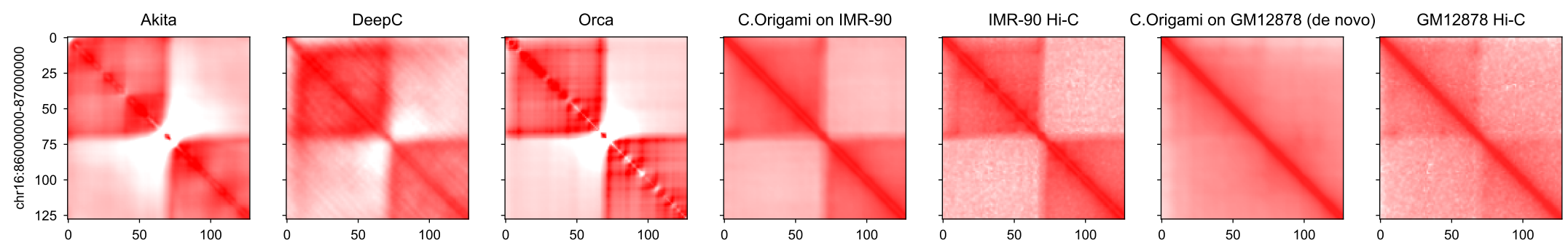

Supplement: Supplementary file 3 — Cell-type-specific predictions. [file 41587_2022_1612_MOESM3_ESM.zip › Cell type-specific predictions/chr16_86500000.pdf]

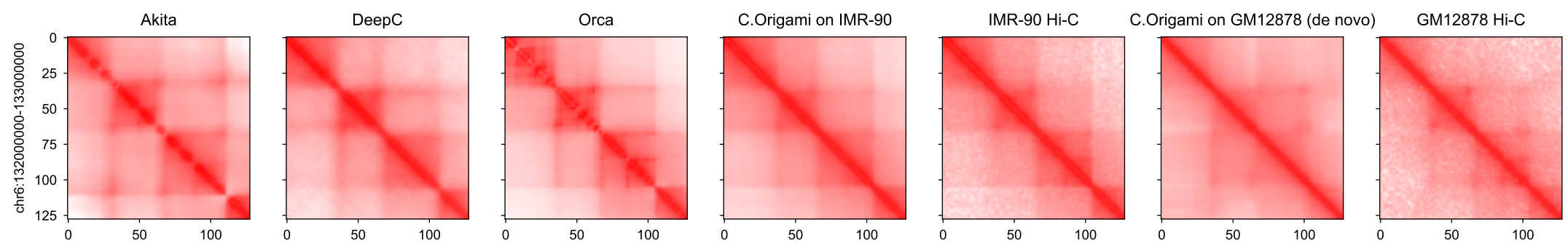

Supplement: Supplementary file 3 — Cell-type-specific predictions. [file 41587_2022_1612_MOESM3_ESM.zip › Cell type-specific predictions/chr6_132500000.pdf]

chr15:360000000-370000000

Akita

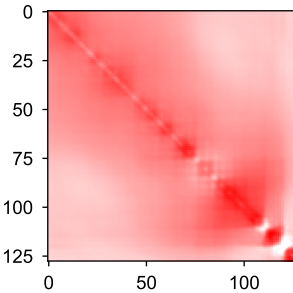

DeepC

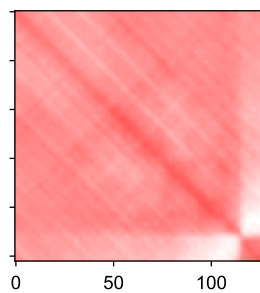

Orca

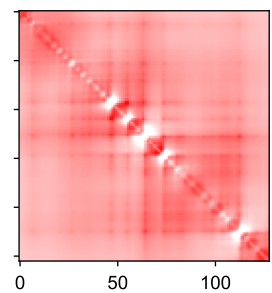

C.Origami on IMR-90

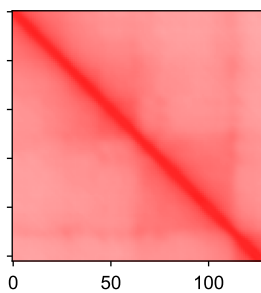

IMR-90 Hi-C

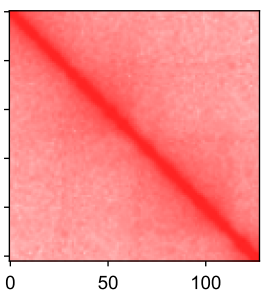

C.Origami on GM12878 (de novo)

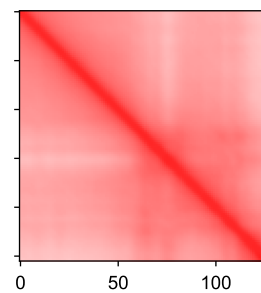

GM12878 Hi-C

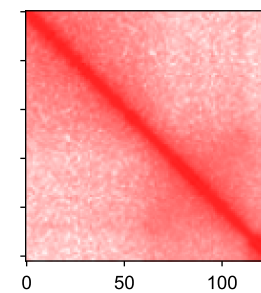

Supplement: Supplementary file 3 — Cell-type-specific predictions. [file 41587_2022_1612_MOESM3_ESM.zip › Cell type-specific predictions/chr15_36500000.pdf]

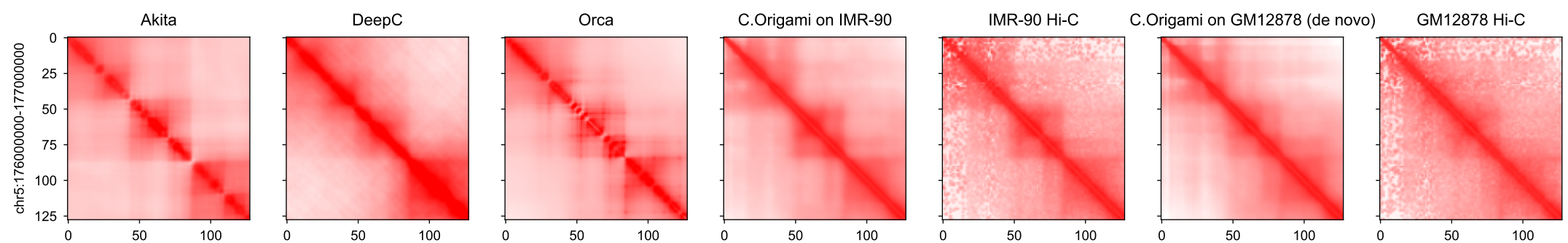

Supplement: Supplementary file 3 — Cell-type-specific predictions. [file 41587_2022_1612_MOESM3_ESM.zip › Cell type-specific predictions/chr5_176500000.pdf]

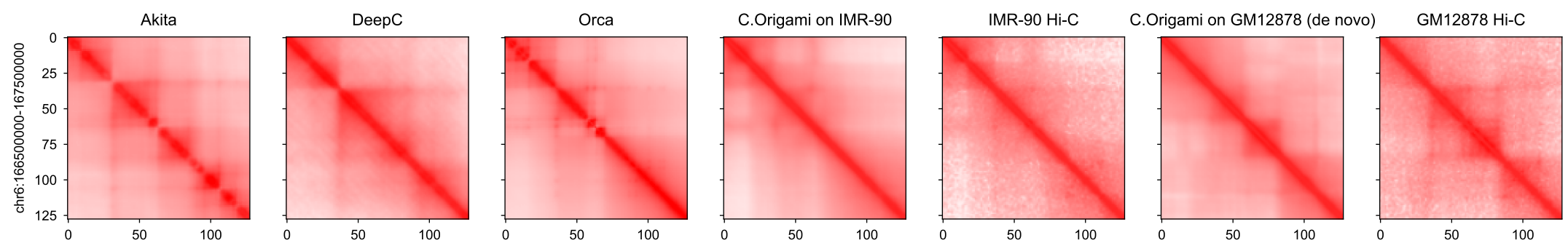

Supplement: Supplementary file 3 — Cell-type-specific predictions. [file 41587_2022_1612_MOESM3_ESM.zip › Cell type-specific predictions/chr6_167000000.pdf]

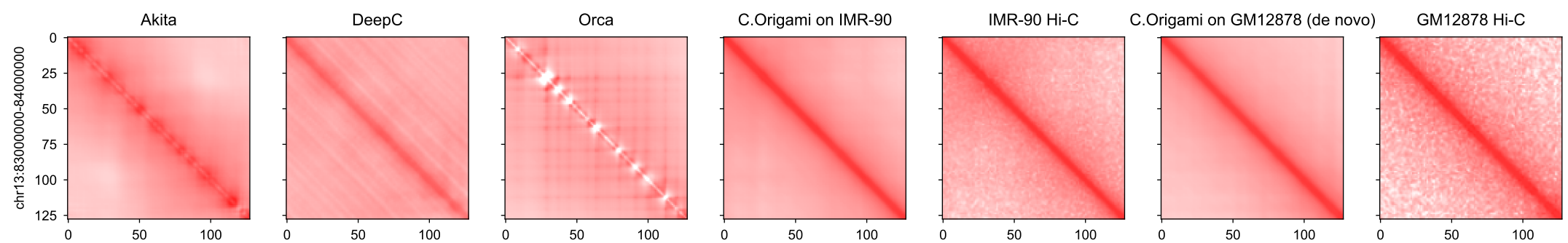

Supplement: Supplementary file 3 — Cell-type-specific predictions. [file 41587_2022_1612_MOESM3_ESM.zip › Cell type-specific predictions/chr13_83500000.pdf]

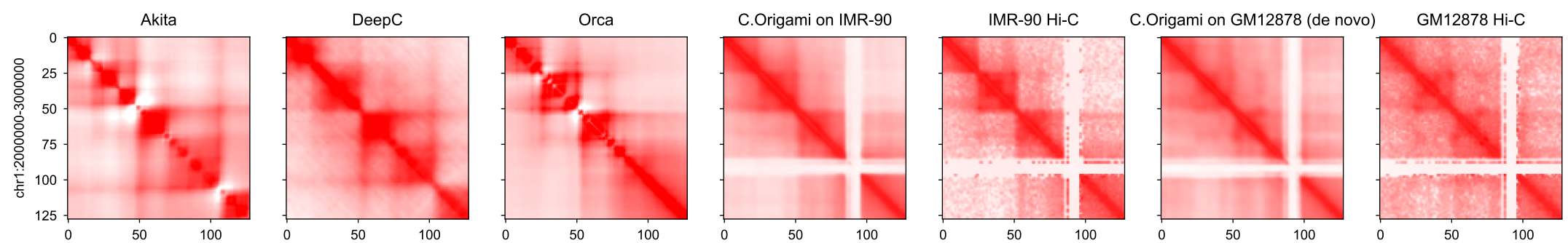

Supplement: Supplementary file 3 — Cell-type-specific predictions. [file 41587_2022_1612_MOESM3_ESM.zip › Cell type-specific predictions/chr1_2500000.pdf]

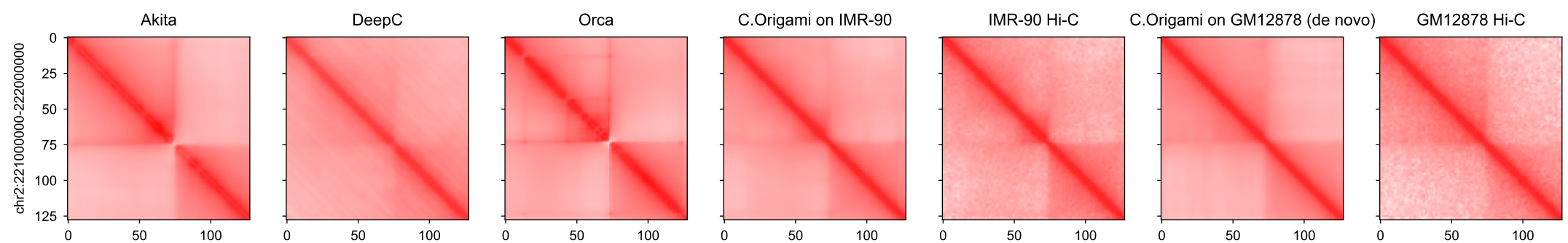

Supplement: Supplementary file 3 — Cell-type-specific predictions. [file 41587_2022_1612_MOESM3_ESM.zip › Cell type-specific predictions/chr2_221500000.pdf]

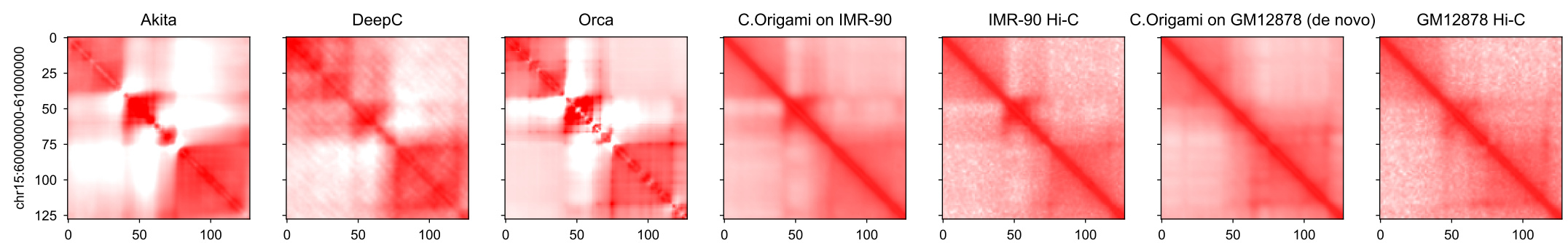

Supplement: Supplementary file 3 — Cell-type-specific predictions. [file 41587_2022_1612_MOESM3_ESM.zip › Cell type-specific predictions/chr15_60500000.pdf]

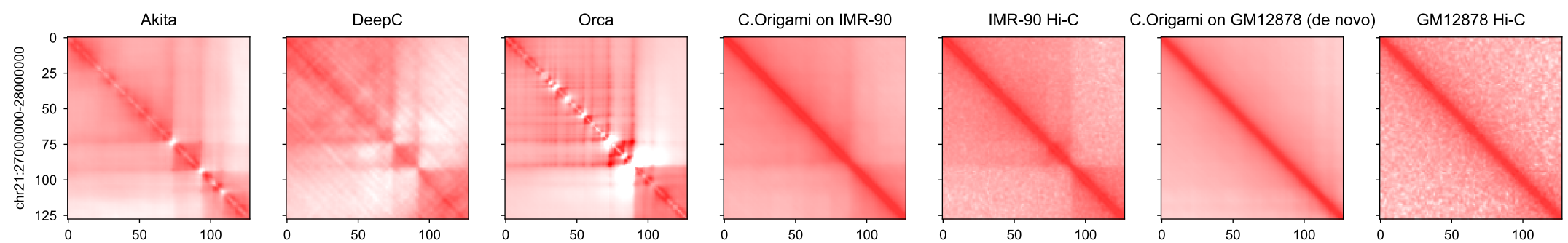

Supplement: Supplementary file 3 — Cell-type-specific predictions. [file 41587_2022_1612_MOESM3_ESM.zip › Cell type-specific predictions/chr21_27500000.pdf]

chr5:17000000-18000000

Akita

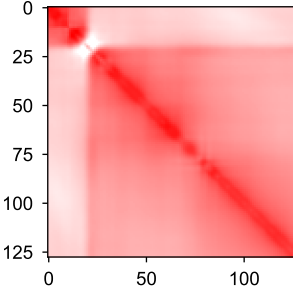

DeepC

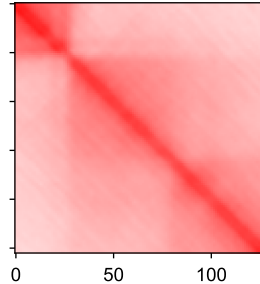

Orca

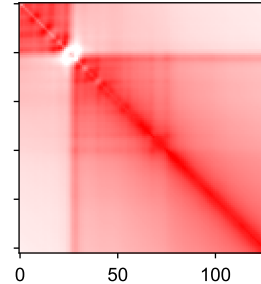

C.Origami on IMR-90

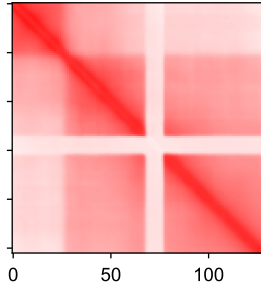

IMR-90 Hi-C

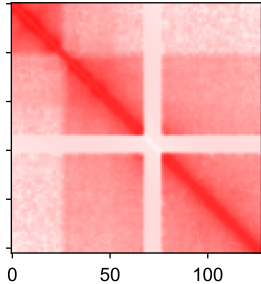

C.Origami on GM12878 (de novo)

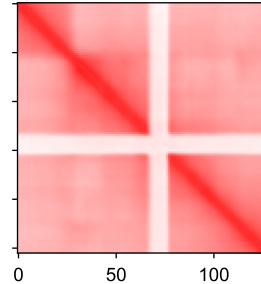

GM12878 Hi-C

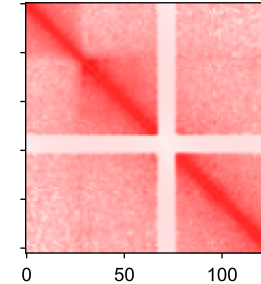

Supplement: Supplementary file 3 — Cell-type-specific predictions. [file 41587_2022_1612_MOESM3_ESM.zip › Cell type-specific predictions/chr5_17500000.pdf]

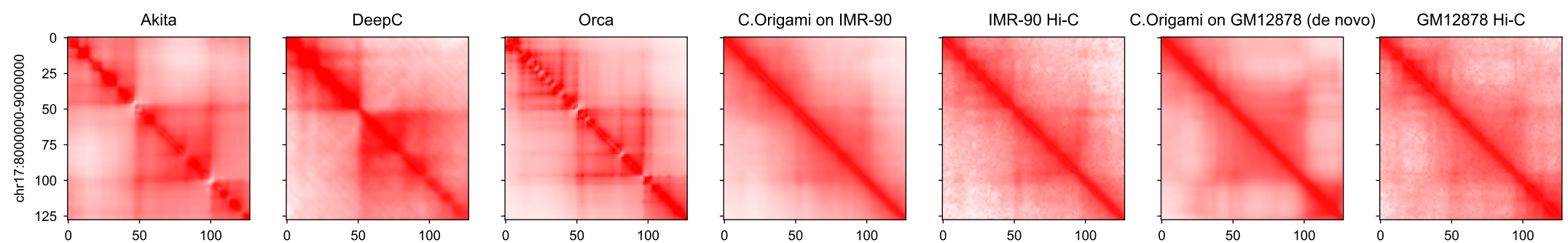

Supplement: Supplementary file 3 — Cell-type-specific predictions. [file 41587_2022_1612_MOESM3_ESM.zip › Cell type-specific predictions/chr17_8500000.pdf]

chr14:26500000-27500000

Akita

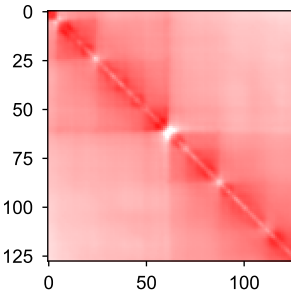

DeepC

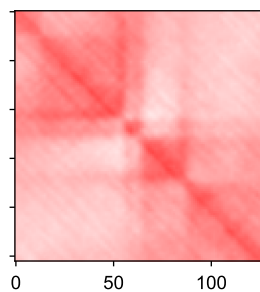

Orca

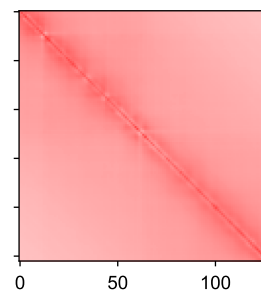

C.Origami on IMR-90

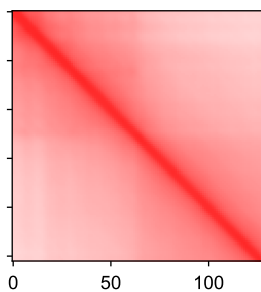

IMR-90 Hi-C

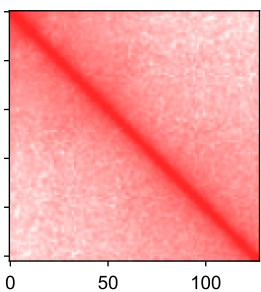

C.Origami on GM12878 (de novo)

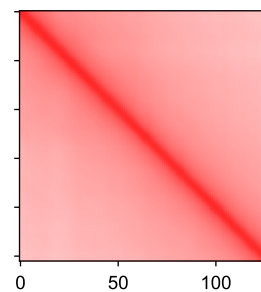

GM12878 Hi-C

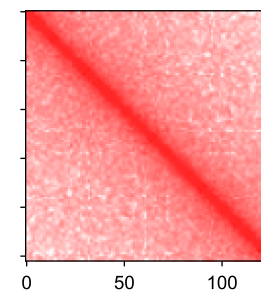

Supplement: Supplementary file 3 — Cell-type-specific predictions. [file 41587_2022_1612_MOESM3_ESM.zip › Cell type-specific predictions/chr14_27000000.pdf]

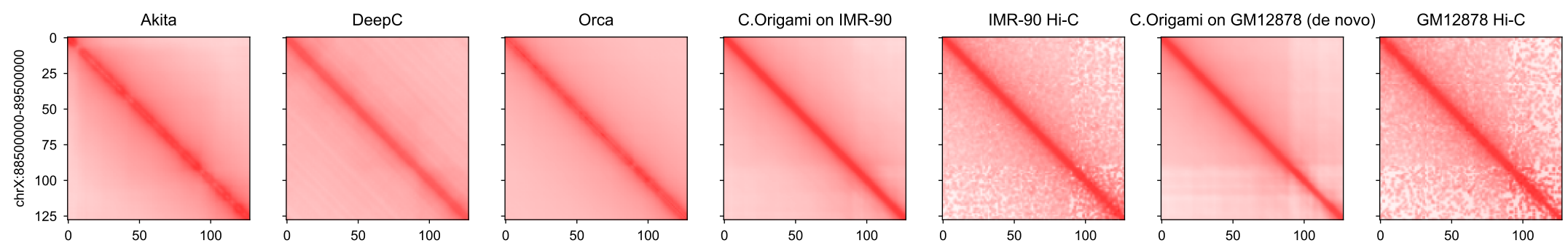

Supplement: Supplementary file 3 — Cell-type-specific predictions. [file 41587_2022_1612_MOESM3_ESM.zip › Cell type-specific predictions/chrX_89000000.pdf]

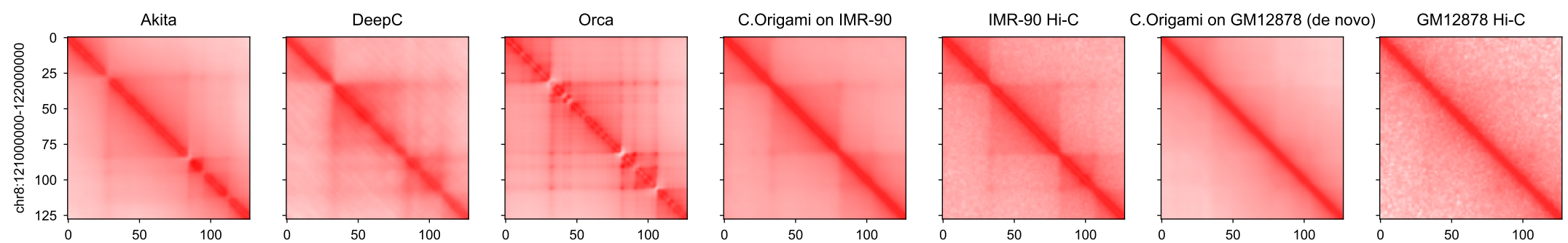

Supplement: Supplementary file 3 — Cell-type-specific predictions. [file 41587_2022_1612_MOESM3_ESM.zip › Cell type-specific predictions/chr8_121500000.pdf]

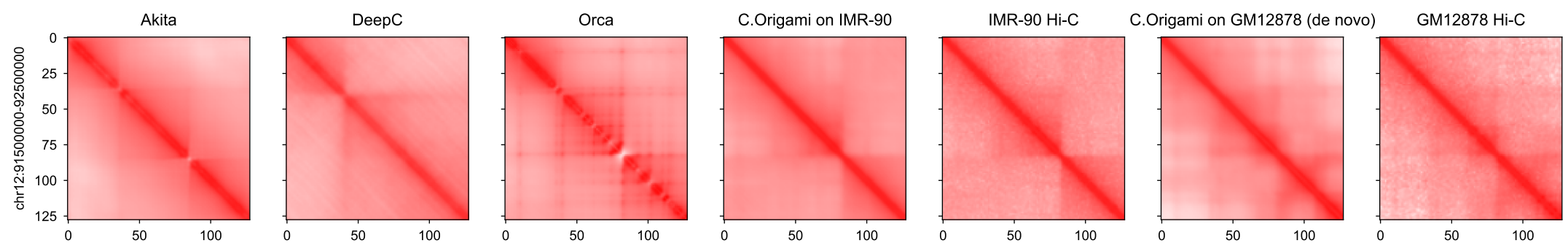

Supplement: Supplementary file 3 — Cell-type-specific predictions. [file 41587_2022_1612_MOESM3_ESM.zip › Cell type-specific predictions/chr12_92000000.pdf]

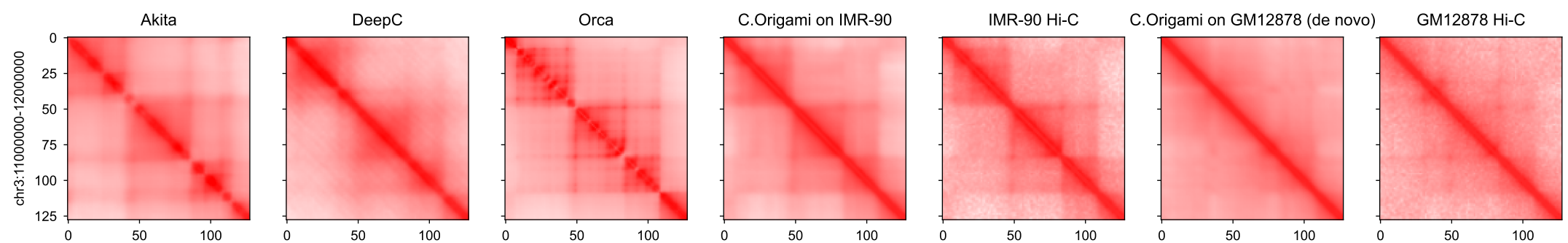

Supplement: Supplementary file 3 — Cell-type-specific predictions. [file 41587_2022_1612_MOESM3_ESM.zip › Cell type-specific predictions/chr3_11500000.pdf]

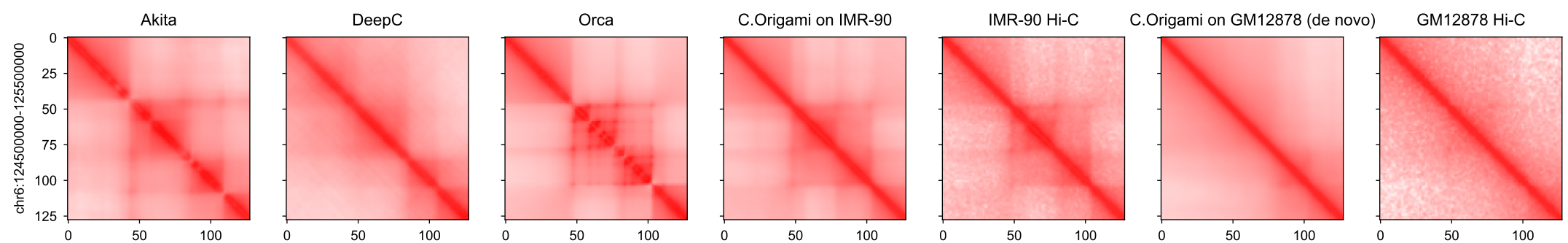

Supplement: Supplementary file 3 — Cell-type-specific predictions. [file 41587_2022_1612_MOESM3_ESM.zip › Cell type-specific predictions/chr6_125000000.pdf]

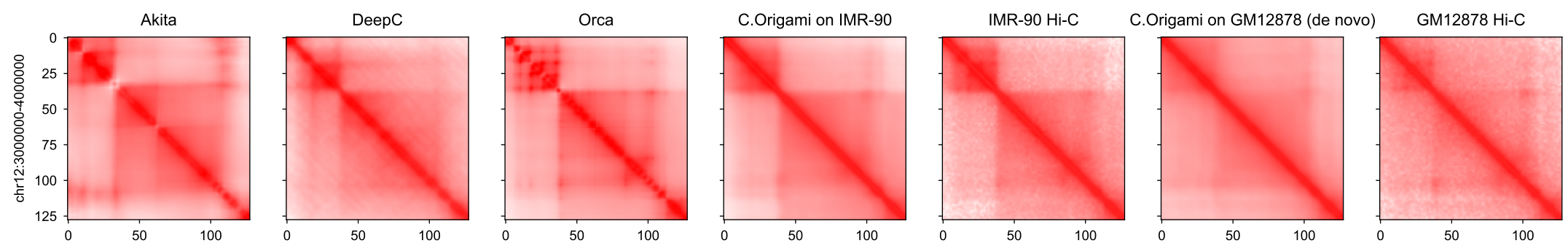

Supplement: Supplementary file 3 — Cell-type-specific predictions. [file 41587_2022_1612_MOESM3_ESM.zip › Cell type-specific predictions/chr12_3500000.pdf]

chr14:290000000-300000000

Akita

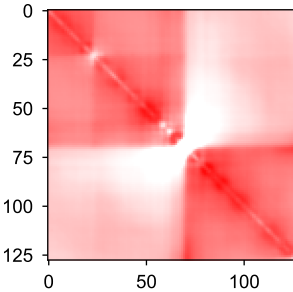

DeepC

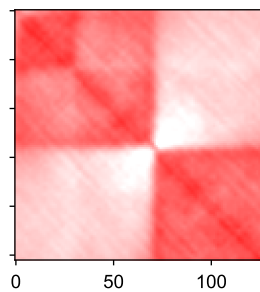

Orca

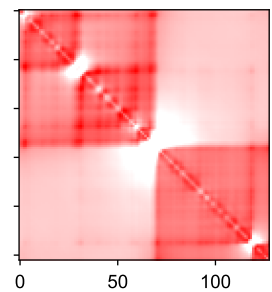

C.Origami on IMR-90

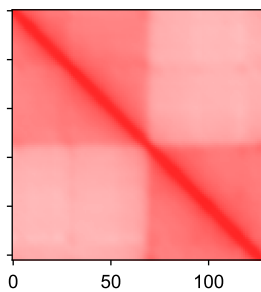

IMR-90 Hi-C

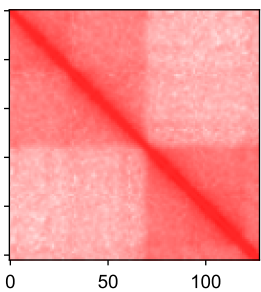

C.Origami on GM12878 (de novo)

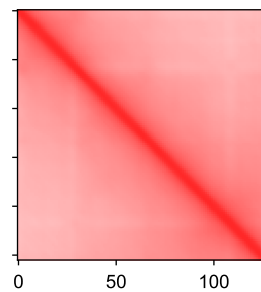

GM12878 Hi-C

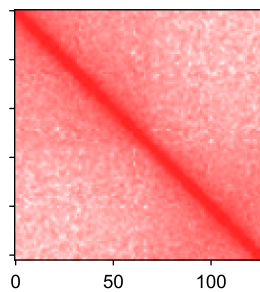

Supplement: Supplementary file 3 — Cell-type-specific predictions. [file 41587_2022_1612_MOESM3_ESM.zip › Cell type-specific predictions/chr14_29500000.pdf]

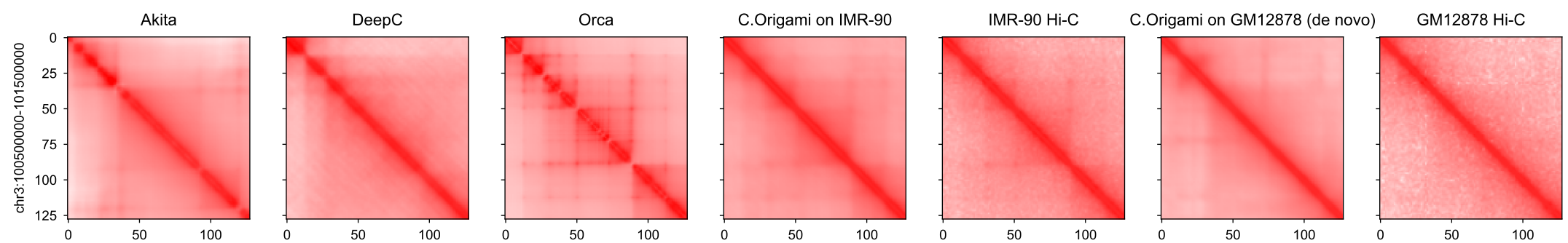

Supplement: Supplementary file 3 — Cell-type-specific predictions. [file 41587_2022_1612_MOESM3_ESM.zip › Cell type-specific predictions/chr3_101000000.pdf]

chr7:65500000-66500000

Akita

DeepC

Orca

C.Origami on IMR-90

IMR-90 Hi-C

C.Origami on GM12878 (de novo)

GM12878 Hi-C

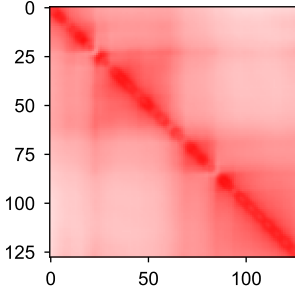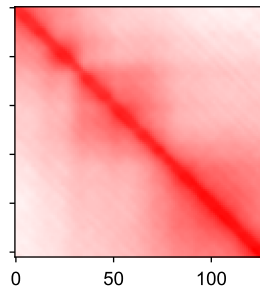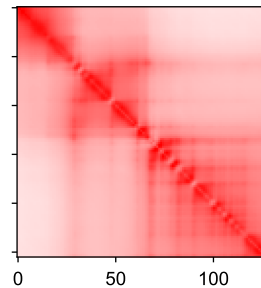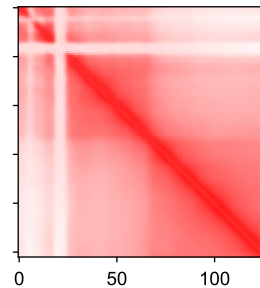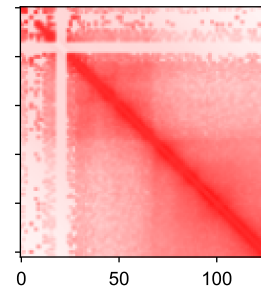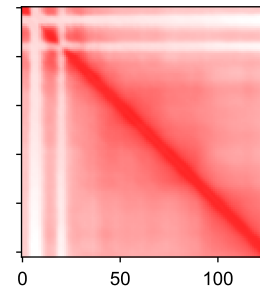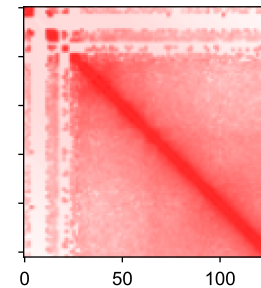

Supplement: Supplementary file 3 — Cell-type-specific predictions. [file 41587_2022_1612_MOESM3_ESM.zip › Cell type-specific predictions/chr7_66000000.pdf]

chr4:30000000-31000000

Akita

DeepC

Orca

C.Origami on IMR-90

IMR-90 Hi-C

C.Origami on GM12878 (de novo)

GM12878 Hi-C

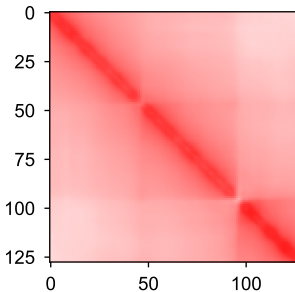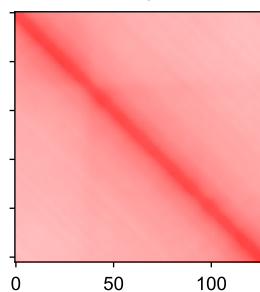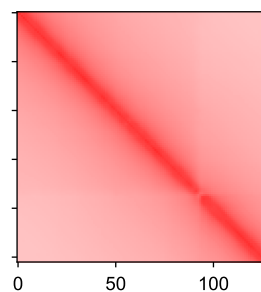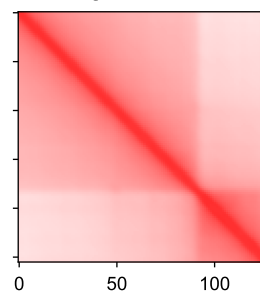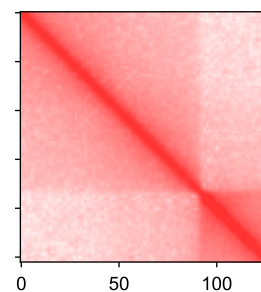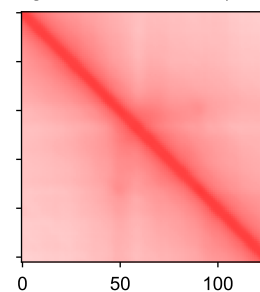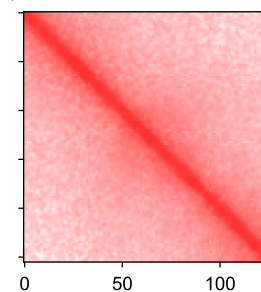

Supplement: Supplementary file 3 — Cell-type-specific predictions. [file 41587_2022_1612_MOESM3_ESM.zip › Cell type-specific predictions/chr4_30500000.pdf]

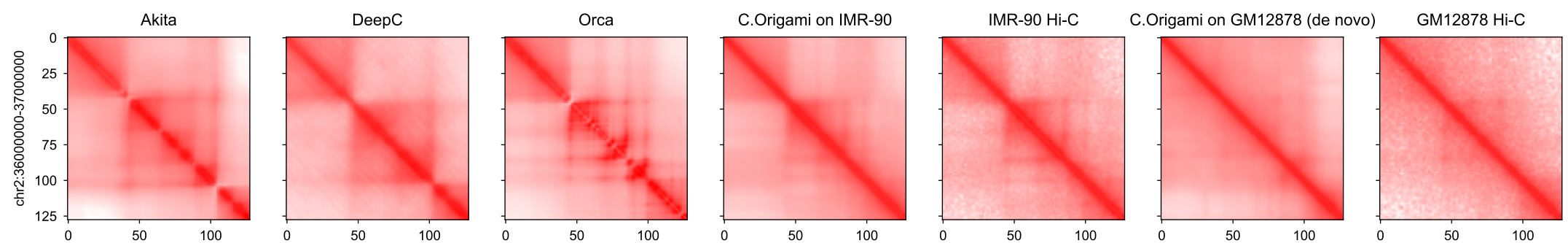

Supplement: Supplementary file 3 — Cell-type-specific predictions. [file 41587_2022_1612_MOESM3_ESM.zip › Cell type-specific predictions/chr2_36500000.pdf]

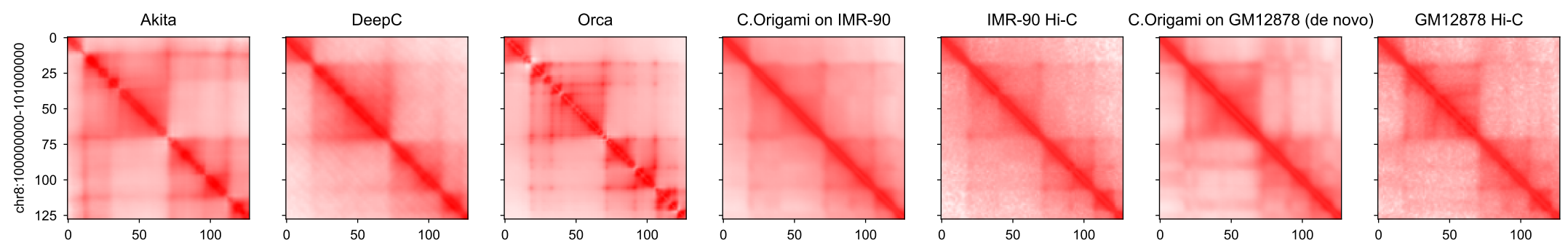

Supplement: Supplementary file 3 — Cell-type-specific predictions. [file 41587_2022_1612_MOESM3_ESM.zip › Cell type-specific predictions/chr8_100500000.pdf]

chr5:18500000-19500000

Akita

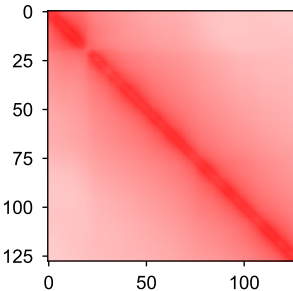

DeepC

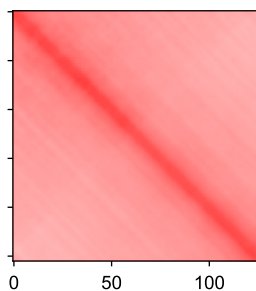

Orca

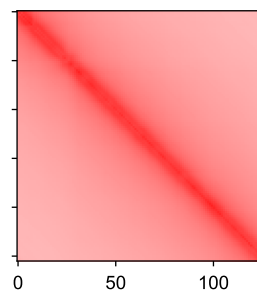

C.Origami on IMR-90

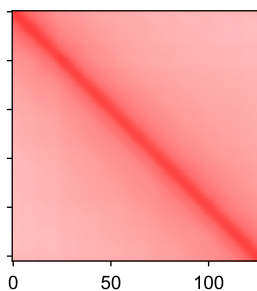

IMR-90 Hi-C

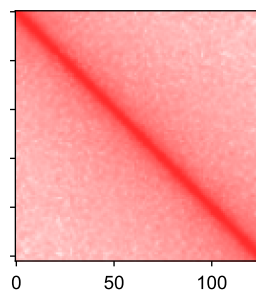

C.Origami on GM12878 (de novo)

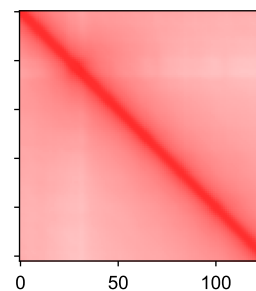

GM12878 Hi-C

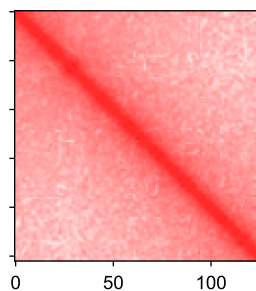

Supplement: Supplementary file 3 — Cell-type-specific predictions. [file 41587_2022_1612_MOESM3_ESM.zip › Cell type-specific predictions/chr5_19000000.pdf]

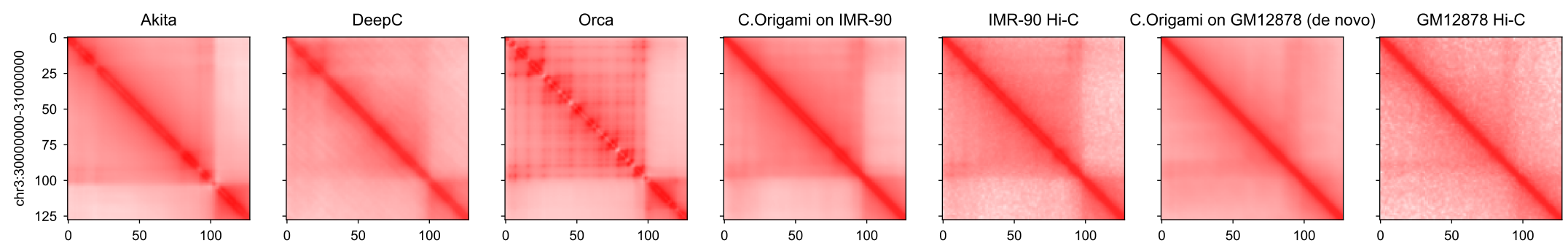

Supplement: Supplementary file 3 — Cell-type-specific predictions. [file 41587_2022_1612_MOESM3_ESM.zip › Cell type-specific predictions/chr3_30500000.pdf]

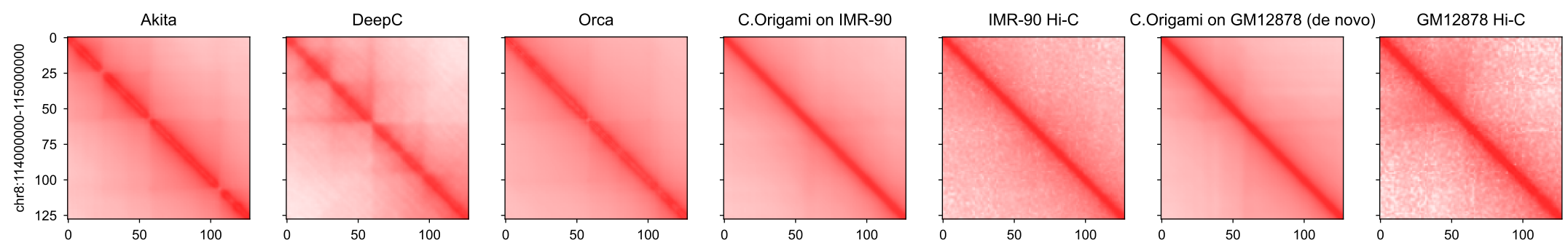

Supplement: Supplementary file 3 — Cell-type-specific predictions. [file 41587_2022_1612_MOESM3_ESM.zip › Cell type-specific predictions/chr8_114500000.pdf]

chr7:94000000-95000000

Akita

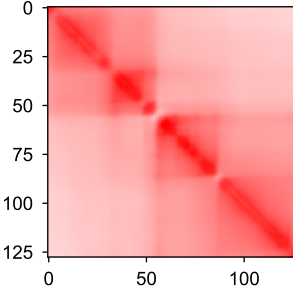

DeepC

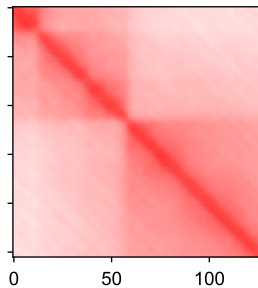

Orca

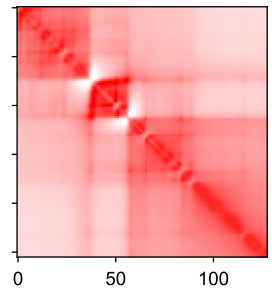

C.Origami on IMR-90

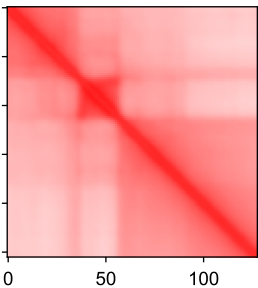

IMR-90 Hi-C

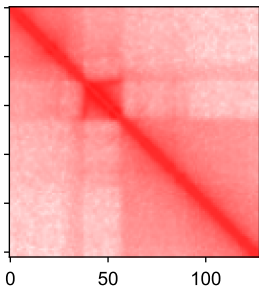

C.Origami on GM12878 (de novo)

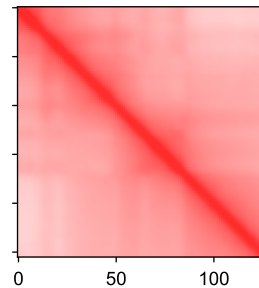

GM12878 Hi-C

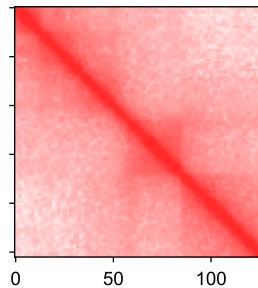

Supplement: Supplementary file 3 — Cell-type-specific predictions. [file 41587_2022_1612_MOESM3_ESM.zip › Cell type-specific predictions/chr7_94500000.pdf]

chr8:12500000-13500000

Akita

DeepC

Orca

C.Origami on IMR-90

IMR-90 Hi-C

C.Origami on GM12878 (de novo)

GM12878 Hi-C

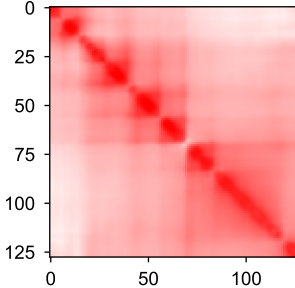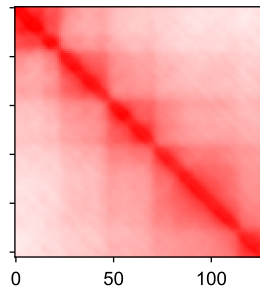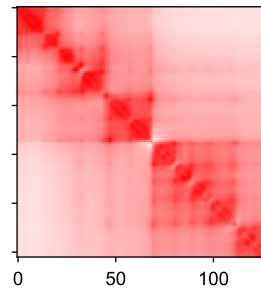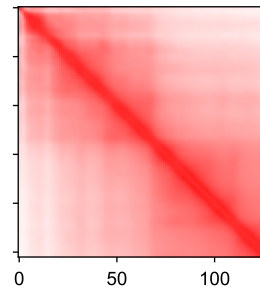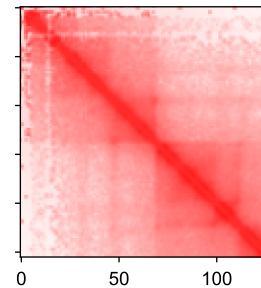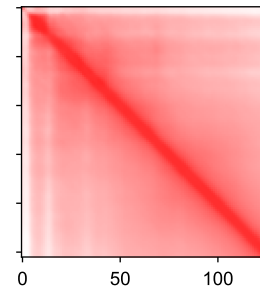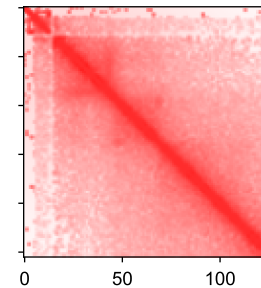

Supplement: Supplementary file 3 — Cell-type-specific predictions. [file 41587_2022_1612_MOESM3_ESM.zip › Cell type-specific predictions/chr8_13000000.pdf]

chr16:16500000-17500000

Akita

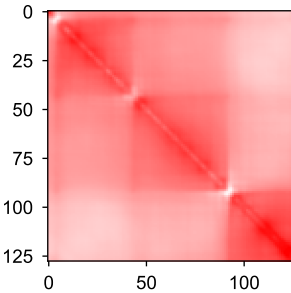

DeepC

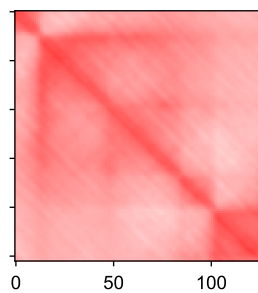

Orca

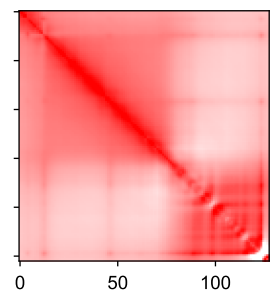

C.Origami on IMR-90

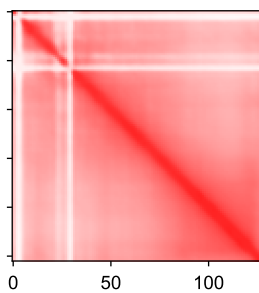

IMR-90 Hi-C

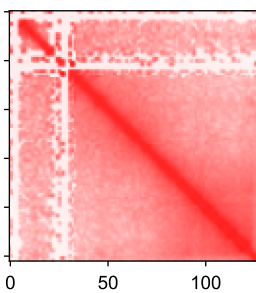

C.Origami on GM12878 (de novo)

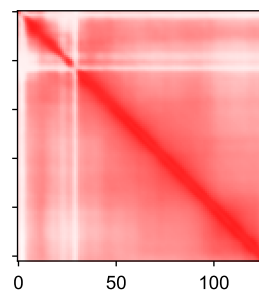

GM12878 Hi-C

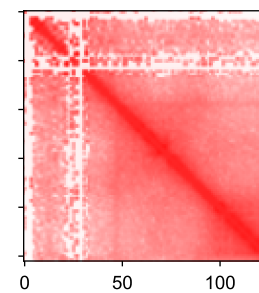

Supplement: Supplementary file 3 — Cell-type-specific predictions. [file 41587_2022_1612_MOESM3_ESM.zip › Cell type-specific predictions/chr16_17000000.pdf]

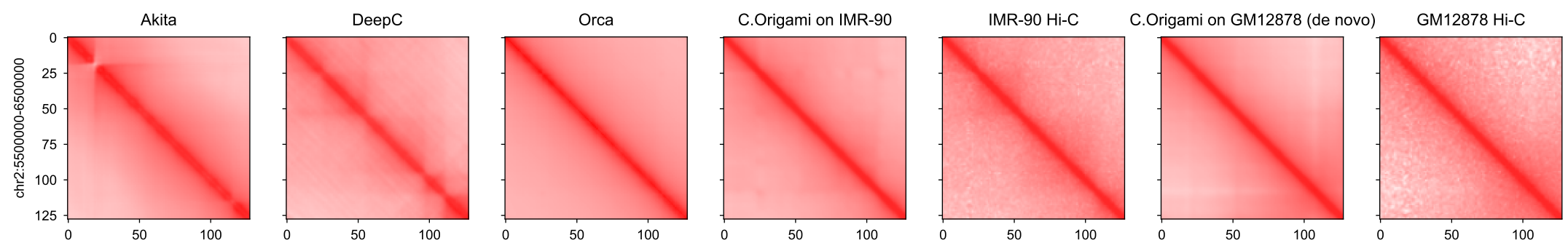

Supplement: Supplementary file 3 — Cell-type-specific predictions. [file 41587_2022_1612_MOESM3_ESM.zip › Cell type-specific predictions/chr2_6000000.pdf]

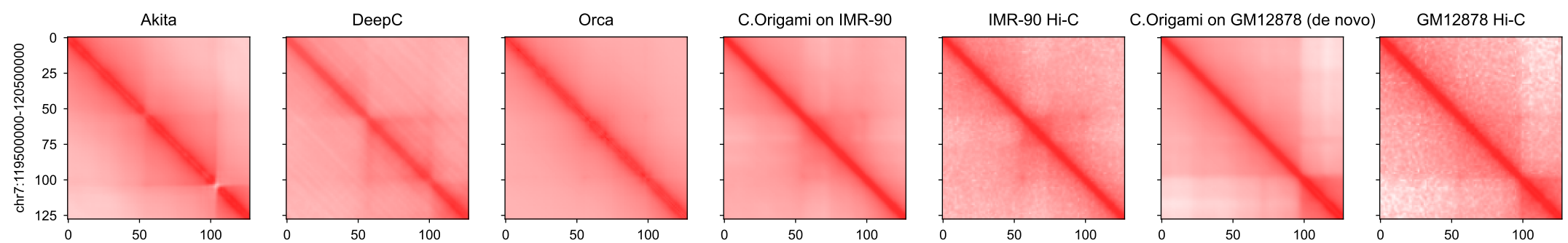

Supplement: Supplementary file 3 — Cell-type-specific predictions. [file 41587_2022_1612_MOESM3_ESM.zip › Cell type-specific predictions/chr7_120000000.pdf]

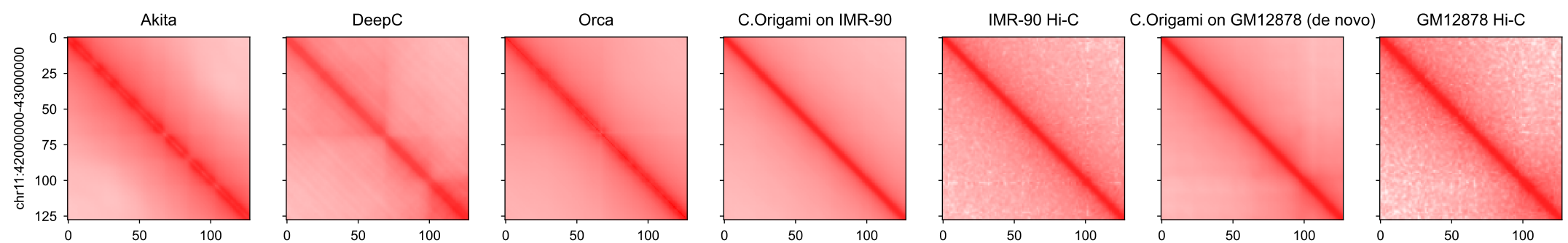

Supplement: Supplementary file 3 — Cell-type-specific predictions. [file 41587_2022_1612_MOESM3_ESM.zip › Cell type-specific predictions/chr11_42500000.pdf]

chr17:73500000-74500000

Akita

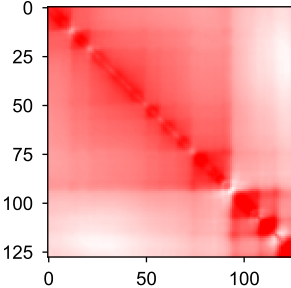

DeepC

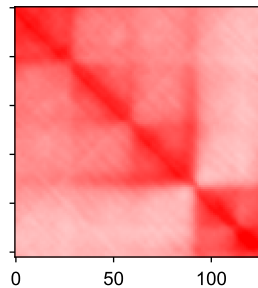

Orca

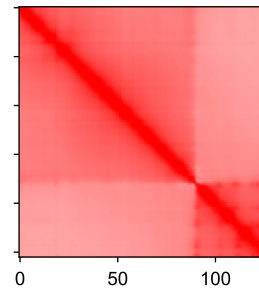

C.Origami on IMR-90

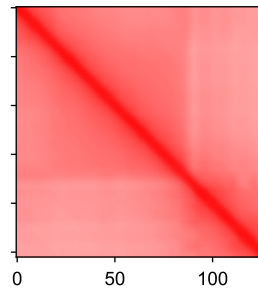

IMR-90 Hi-C

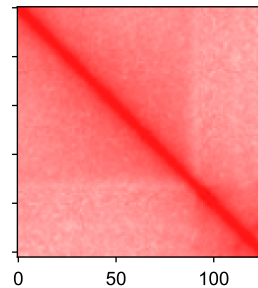

C.Origami on GM12878 (de novo)

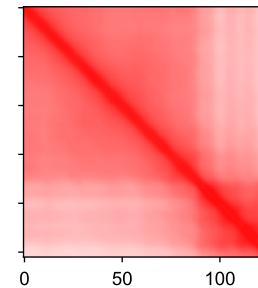

GM12878 Hi-C

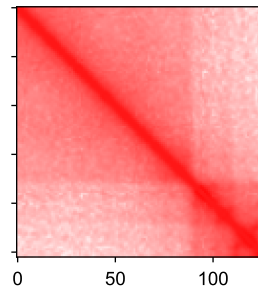

Supplement: Supplementary file 3 — Cell-type-specific predictions. [file 41587_2022_1612_MOESM3_ESM.zip › Cell type-specific predictions/chr17_74000000.pdf]

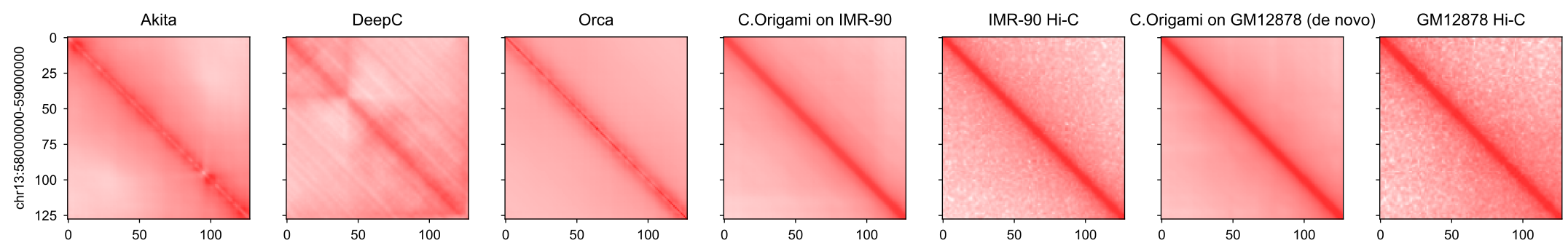

Supplement: Supplementary file 3 — Cell-type-specific predictions. [file 41587_2022_1612_MOESM3_ESM.zip › Cell type-specific predictions/chr13_58500000.pdf]

chr11:94000000-95000000

Akita

DeepC

Orca

C.Origami on IMR-90

IMR-90 Hi-C

C.Origami on GM12878 (de novo)

GM12878 Hi-C

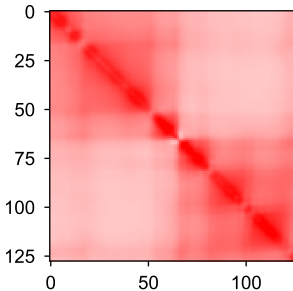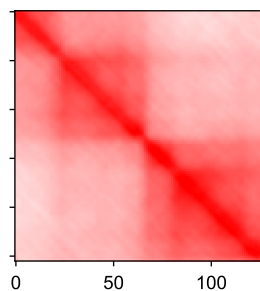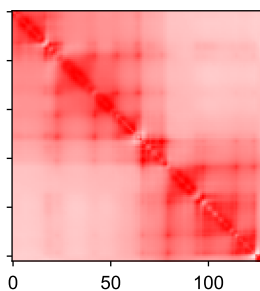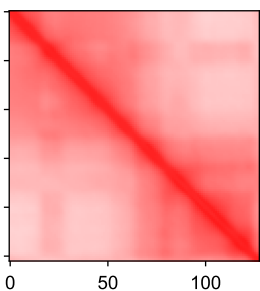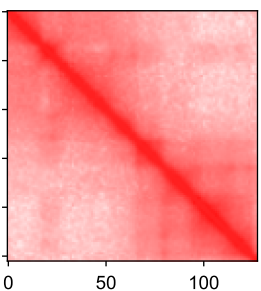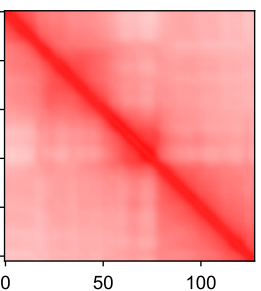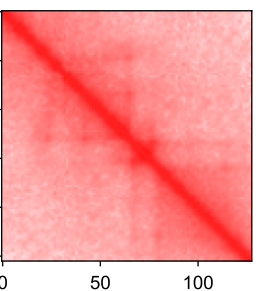

Supplement: Supplementary file 3 — Cell-type-specific predictions. [file 41587_2022_1612_MOESM3_ESM.zip › Cell type-specific predictions/chr11_94500000.pdf]

chr8:75500000-76500000

Akita

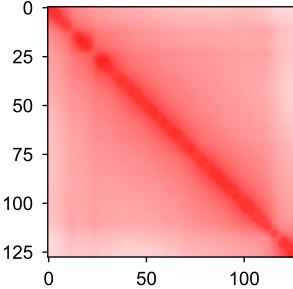

DeepC

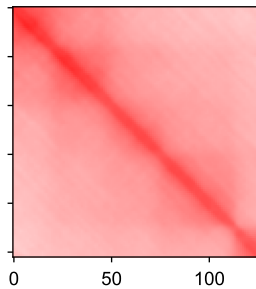

Orca

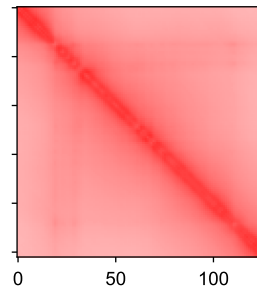

C.Origami on IMR-90

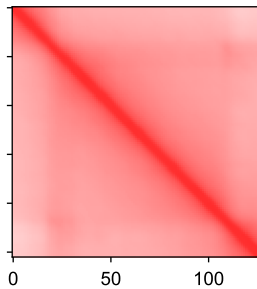

IMR-90 Hi-C

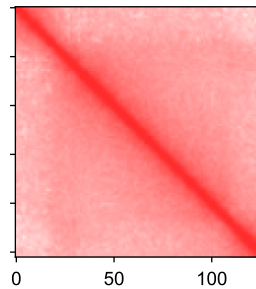

C.Origami on GM12878 (de novo)

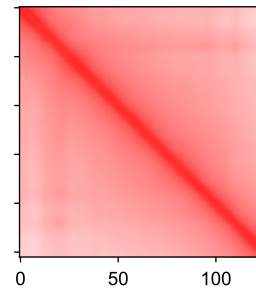

GM12878 Hi-C

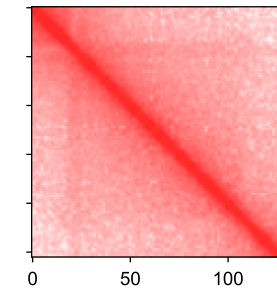

Supplement: Supplementary file 3 — Cell-type-specific predictions. [file 41587_2022_1612_MOESM3_ESM.zip › Cell type-specific predictions/chr8_76000000.pdf]

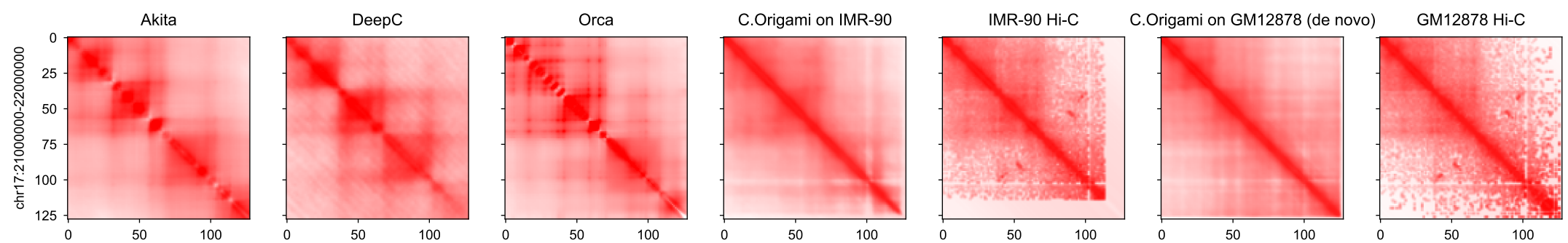

Supplement: Supplementary file 3 — Cell-type-specific predictions. [file 41587_2022_1612_MOESM3_ESM.zip › Cell type-specific predictions/chr17_21500000.pdf]

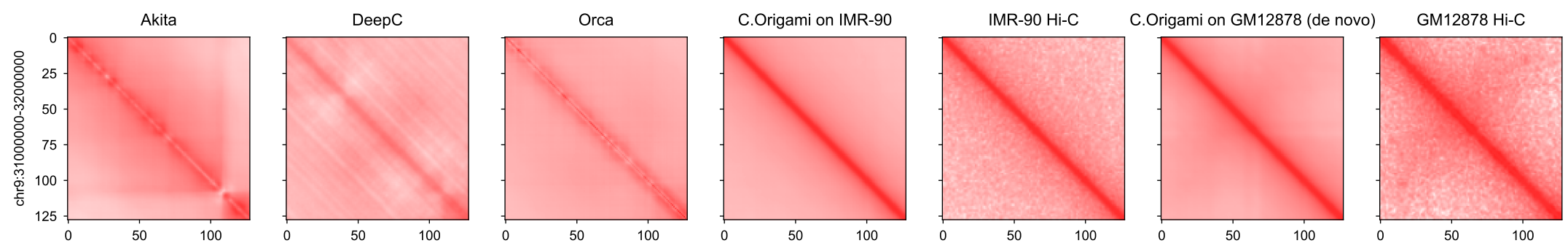

Supplement: Supplementary file 3 — Cell-type-specific predictions. [file 41587_2022_1612_MOESM3_ESM.zip › Cell type-specific predictions/chr9_31500000.pdf]

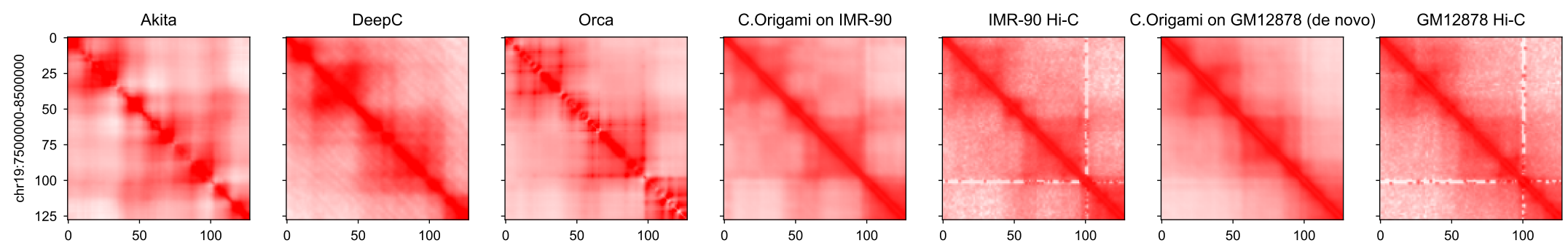

Supplement: Supplementary file 3 — Cell-type-specific predictions. [file 41587_2022_1612_MOESM3_ESM.zip › Cell type-specific predictions/chr19_8000000.pdf]

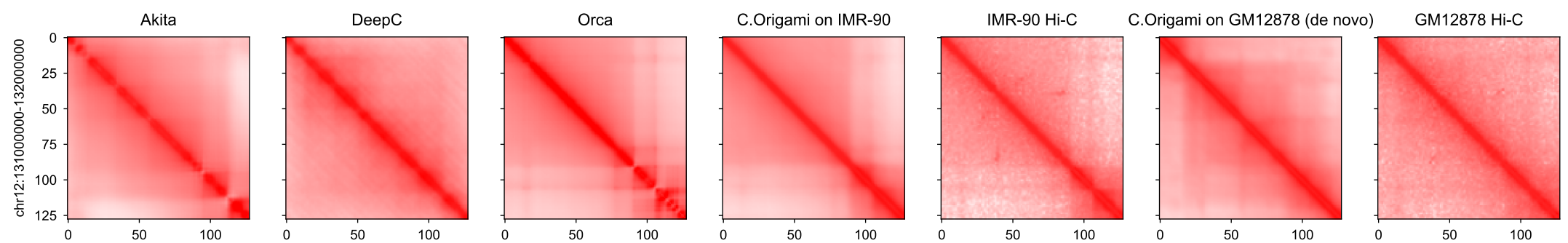

Supplement: Supplementary file 3 — Cell-type-specific predictions. [file 41587_2022_1612_MOESM3_ESM.zip › Cell type-specific predictions/chr12_131500000.pdf]

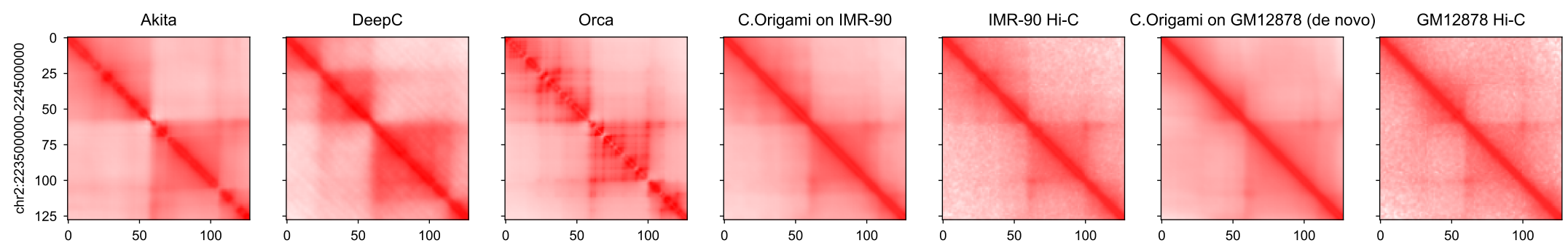

Supplement: Supplementary file 3 — Cell-type-specific predictions. [file 41587_2022_1612_MOESM3_ESM.zip › Cell type-specific predictions/chr2_224000000.pdf]

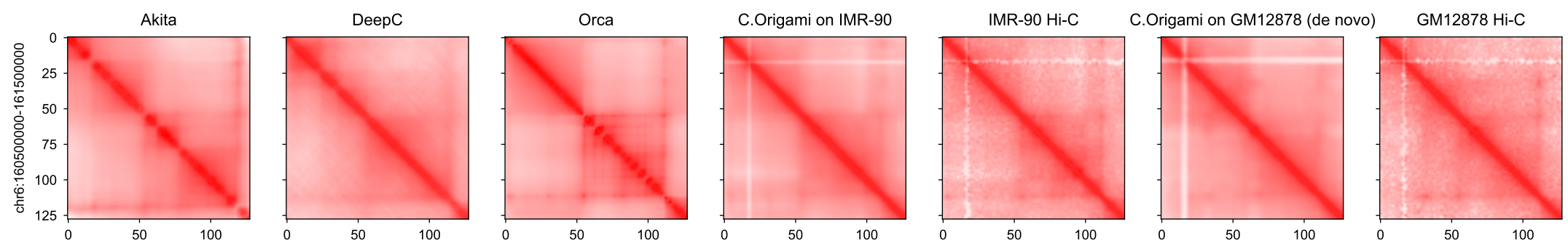

Supplement: Supplementary file 3 — Cell-type-specific predictions. [file 41587_2022_1612_MOESM3_ESM.zip › Cell type-specific predictions/chr6_161000000.pdf]

chr16:33000000-34000000

Akita

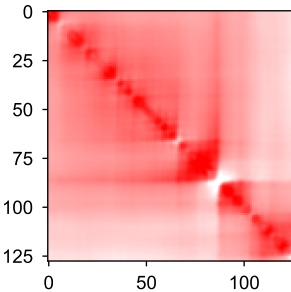

DeepC

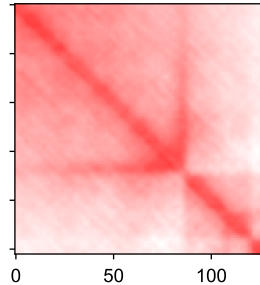

Orca

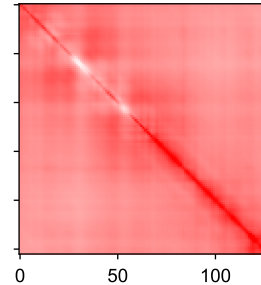

C.Origami on IMR-90

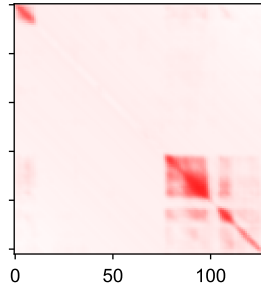

IMR-90 Hi-C

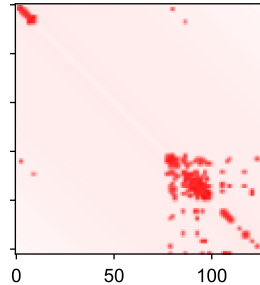

C.Origami on GM12878 (de novo)

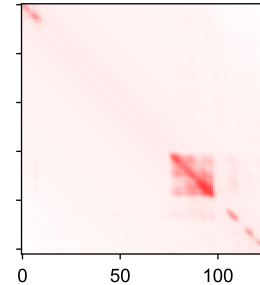

GM12878 Hi-C

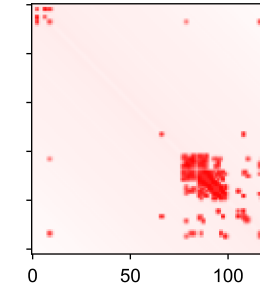

Supplement: Supplementary file 3 — Cell-type-specific predictions. [file 41587_2022_1612_MOESM3_ESM.zip › Cell type-specific predictions/chr16_33500000.pdf]

chr8:17500000-18500000

Akita

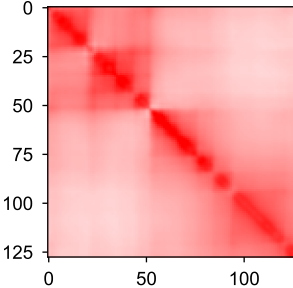

DeepC

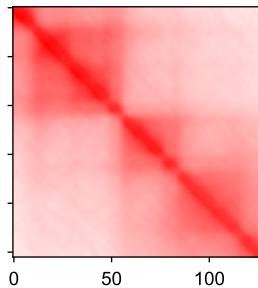

Orca

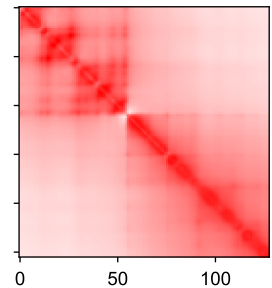

C.Origami on IMR-90

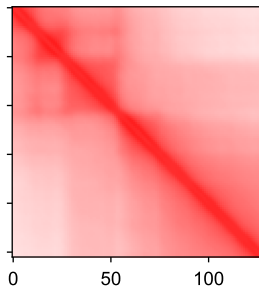

IMR-90 Hi-C

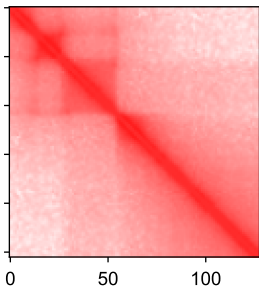

C.Origami on GM12878 (de novo)

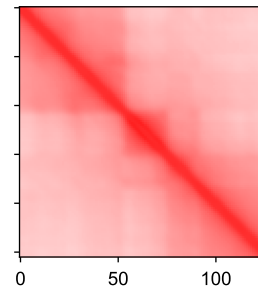

GM12878 Hi-C

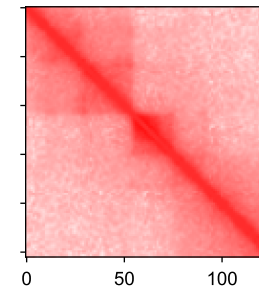

Supplement: Supplementary file 3 — Cell-type-specific predictions. [file 41587_2022_1612_MOESM3_ESM.zip › Cell type-specific predictions/chr8_18000000.pdf]

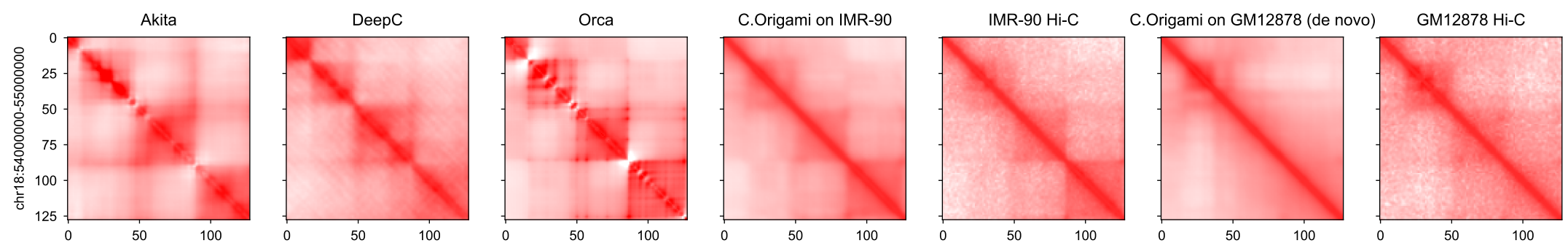

Supplement: Supplementary file 3 — Cell-type-specific predictions. [file 41587_2022_1612_MOESM3_ESM.zip › Cell type-specific predictions/chr18_54500000.pdf]

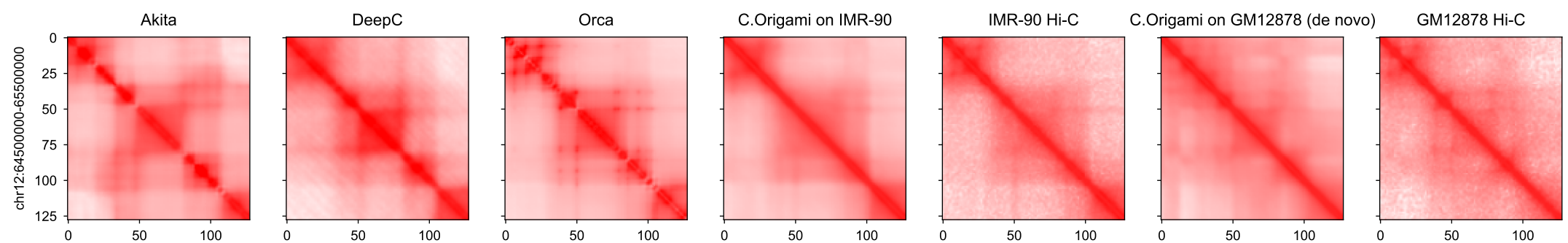

Supplement: Supplementary file 3 — Cell-type-specific predictions. [file 41587_2022_1612_MOESM3_ESM.zip › Cell type-specific predictions/chr12_65000000.pdf]

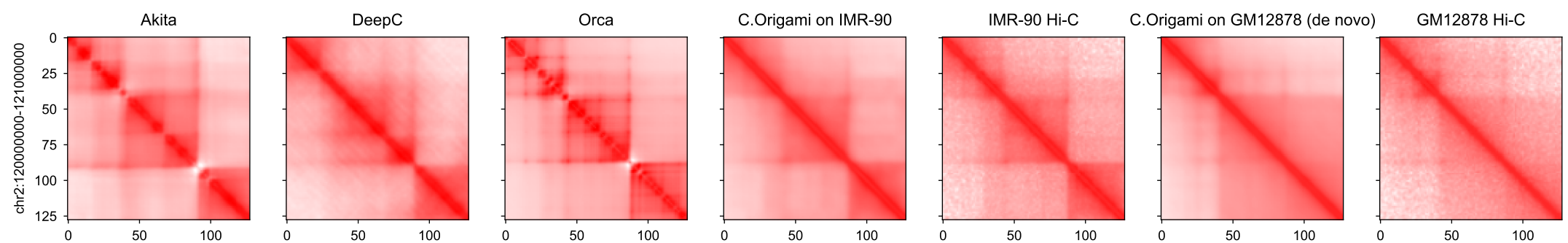

Supplement: Supplementary file 3 — Cell-type-specific predictions. [file 41587_2022_1612_MOESM3_ESM.zip › Cell type-specific predictions/chr2_120500000.pdf]

chr4:186500000-187500000

Akita

DeepC

Orca

C.Origami on IMR-90

IMR-90 Hi-C

C.Origami on GM12878 (de novo)

GM12878 Hi-C

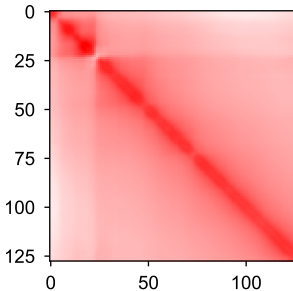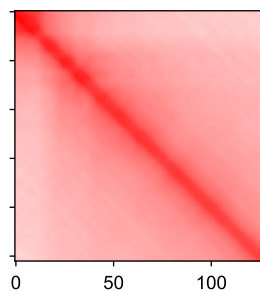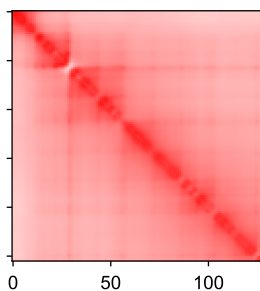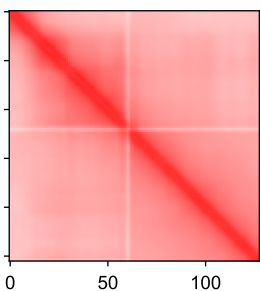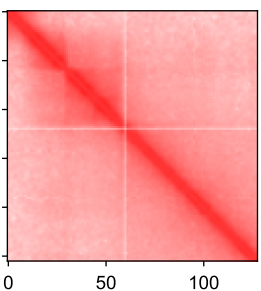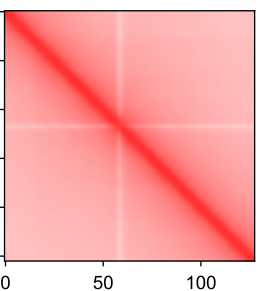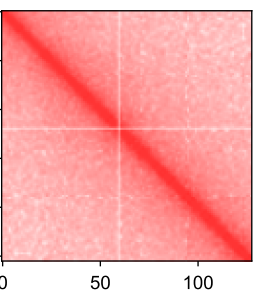

Supplement: Supplementary file 3 — Cell-type-specific predictions. [file 41587_2022_1612_MOESM3_ESM.zip › Cell type-specific predictions/chr4_187000000.pdf]

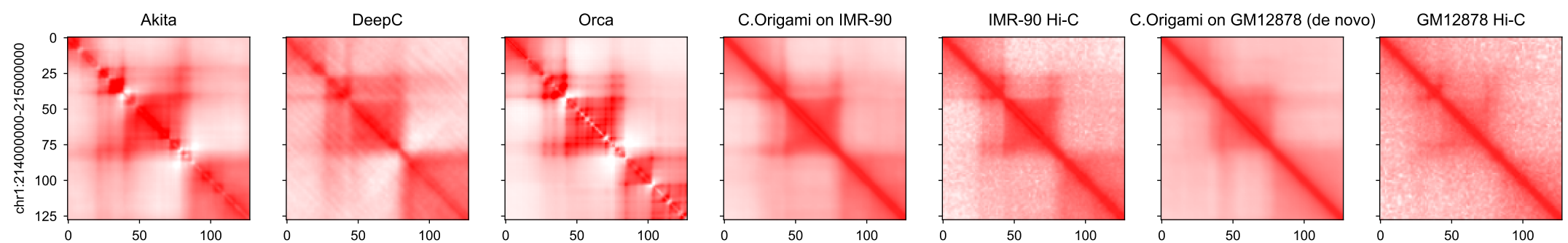

Supplement: Supplementary file 3 — Cell-type-specific predictions. [file 41587_2022_1612_MOESM3_ESM.zip › Cell type-specific predictions/chr1_214500000.pdf]

chr3:74000000-75000000

Akita

DeepC

Orca

C.Origami on IMR-90

IMR-90 Hi-C

C.Origami on GM12878 (de novo)

GM12878 Hi-C

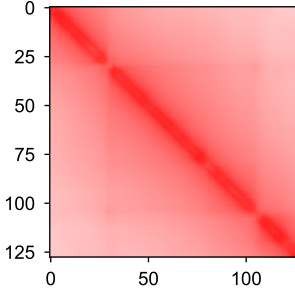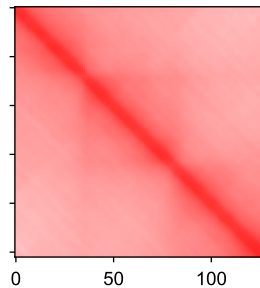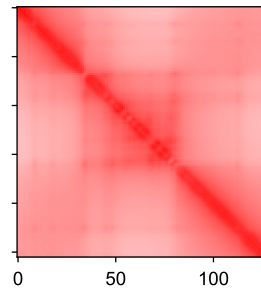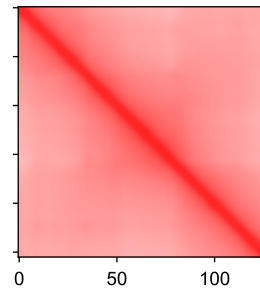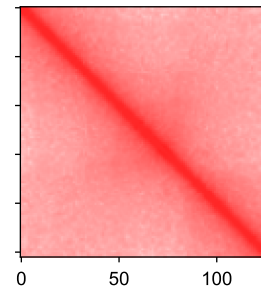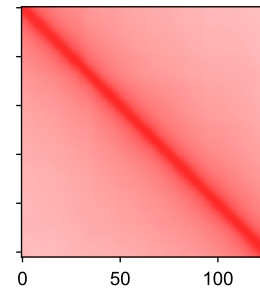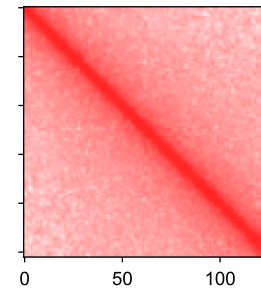

Supplement: Supplementary file 3 — Cell-type-specific predictions. [file 41587_2022_1612_MOESM3_ESM.zip › Cell type-specific predictions/chr3_74500000.pdf]

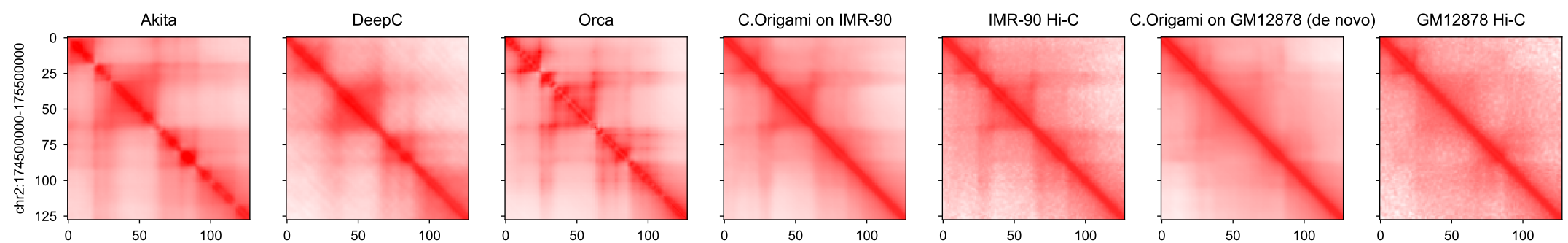

Supplement: Supplementary file 3 — Cell-type-specific predictions. [file 41587_2022_1612_MOESM3_ESM.zip › Cell type-specific predictions/chr2_175000000.pdf]

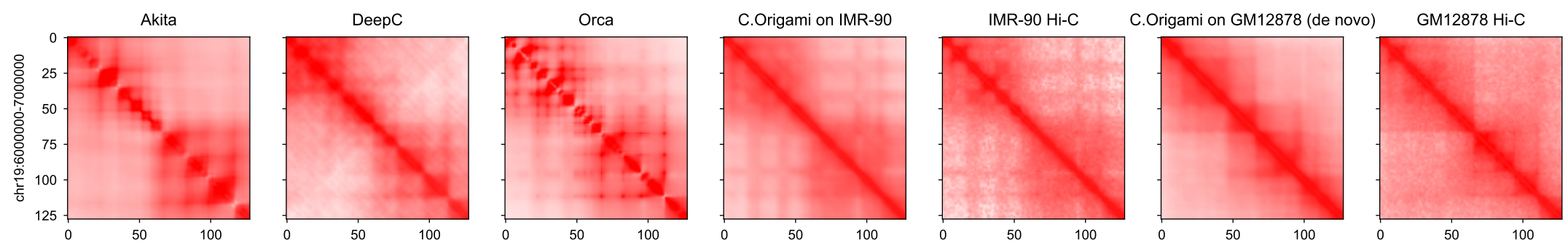

Supplement: Supplementary file 3 — Cell-type-specific predictions. [file 41587_2022_1612_MOESM3_ESM.zip › Cell type-specific predictions/chr19_6500000.pdf]

chr7:77000000-78000000

Akita

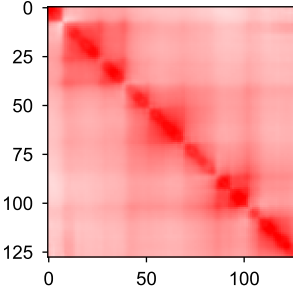

DeepC

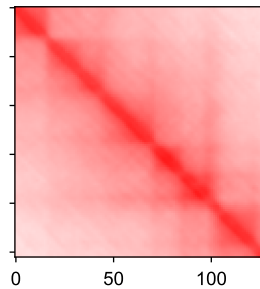

Orca

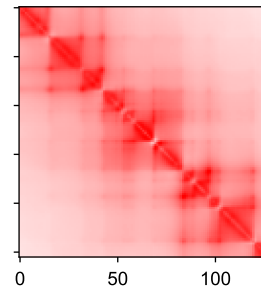

C.Origami on IMR-90

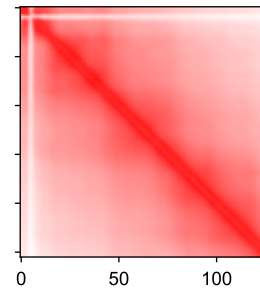

IMR-90 Hi-C

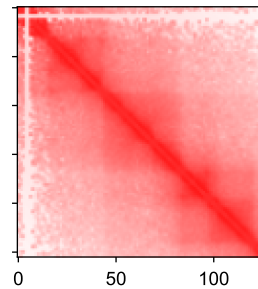

C.Origami on GM12878 (de novo)

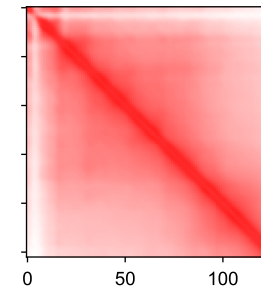

GM12878 Hi-C

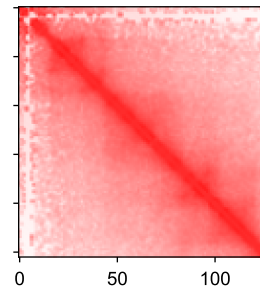

Supplement: Supplementary file 3 — Cell-type-specific predictions. [file 41587_2022_1612_MOESM3_ESM.zip › Cell type-specific predictions/chr7_77500000.pdf]

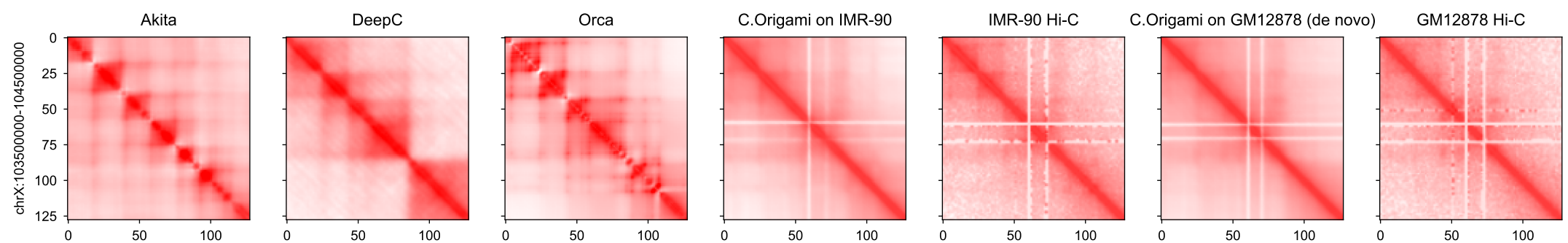

Supplement: Supplementary file 3 — Cell-type-specific predictions. [file 41587_2022_1612_MOESM3_ESM.zip › Cell type-specific predictions/chrX_104000000.pdf]

chr18:75000000-76000000

Akita

DeepC

Orca

C.Origami on IMR-90

IMR-90 Hi-C

C.Origami on GM12878 (de novo)

GM12878 Hi-C

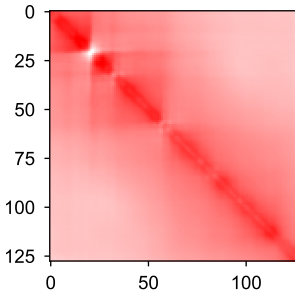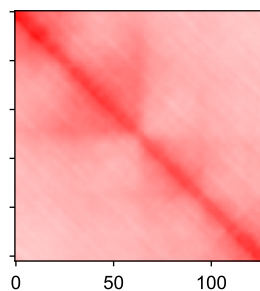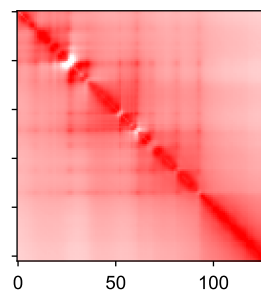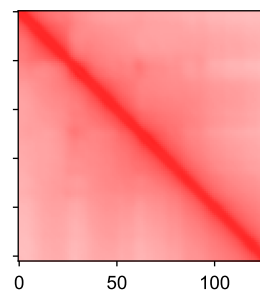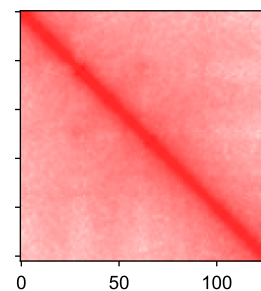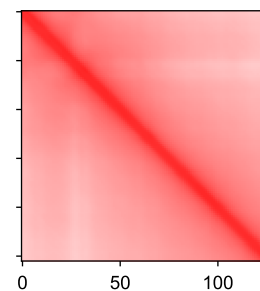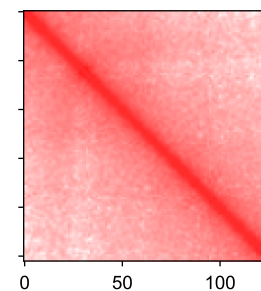

Supplement: Supplementary file 3 — Cell-type-specific predictions. [file 41587_2022_1612_MOESM3_ESM.zip › Cell type-specific predictions/chr18_75500000.pdf]

chr6:71000000-72000000

Akita

DeepC

Orca

C.Origami on IMR-90

IMR-90 Hi-C

C.Origami on GM12878 (de novo)

GM12878 Hi-C

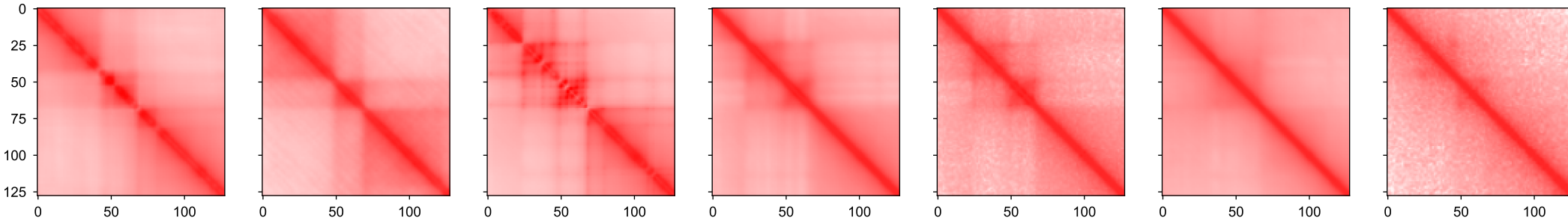

Supplement: Supplementary file 3 — Cell-type-specific predictions. [file 41587_2022_1612_MOESM3_ESM.zip › Cell type-specific predictions/chr6_71500000.pdf]

chr5:26500000-27500000

Akita

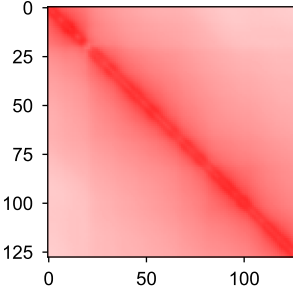

DeepC

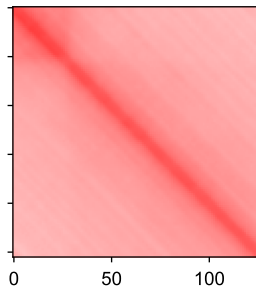

Orca

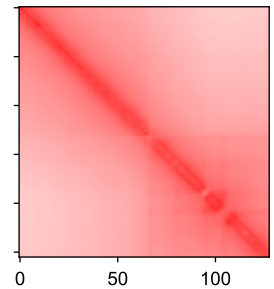

C.Origami on IMR-90

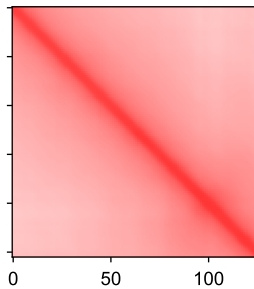

IMR-90 Hi-C

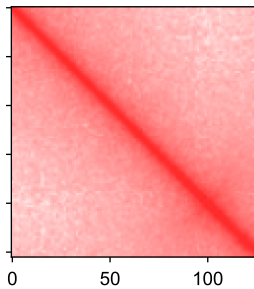

C.Origami on GM12878 (de novo)

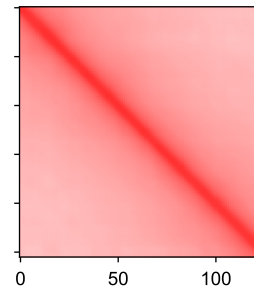

GM12878 Hi-C

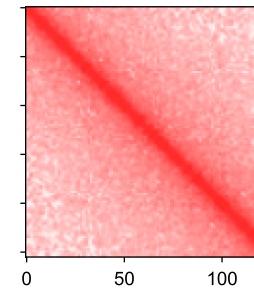

Supplement: Supplementary file 3 — Cell-type-specific predictions. [file 41587_2022_1612_MOESM3_ESM.zip › Cell type-specific predictions/chr5_27000000.pdf]

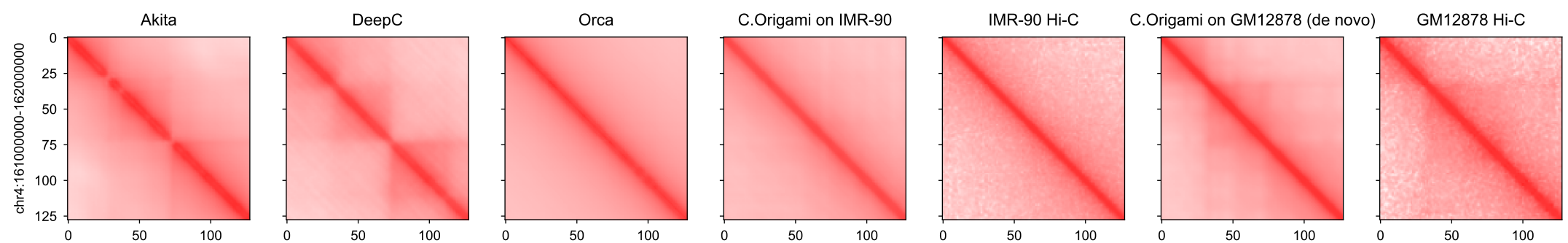

Supplement: Supplementary file 3 — Cell-type-specific predictions. [file 41587_2022_1612_MOESM3_ESM.zip › Cell type-specific predictions/chr4_161500000.pdf]

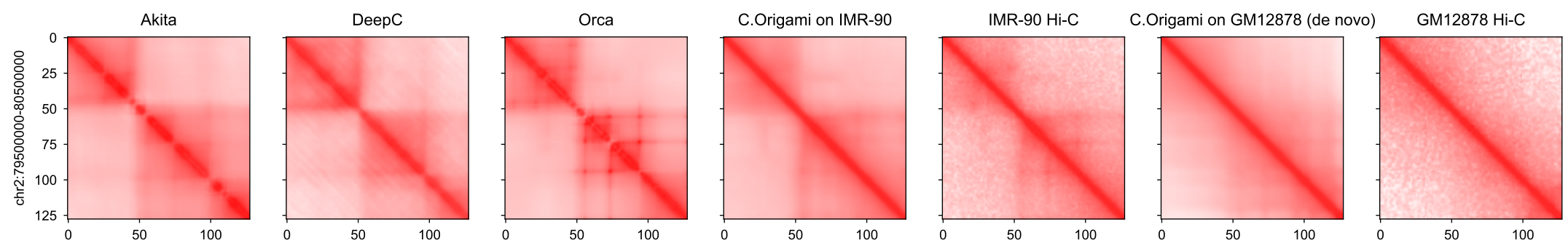

Supplement: Supplementary file 3 — Cell-type-specific predictions. [file 41587_2022_1612_MOESM3_ESM.zip › Cell type-specific predictions/chr2_80000000.pdf]

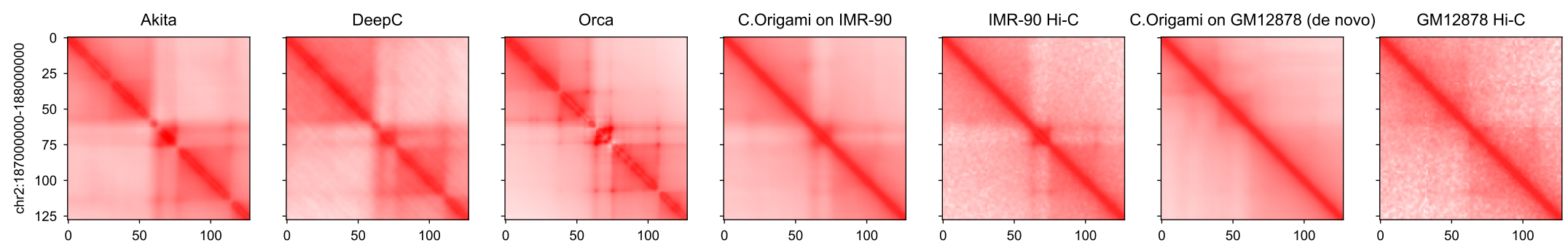

Supplement: Supplementary file 3 — Cell-type-specific predictions. [file 41587_2022_1612_MOESM3_ESM.zip › Cell type-specific predictions/chr2_187500000.pdf]

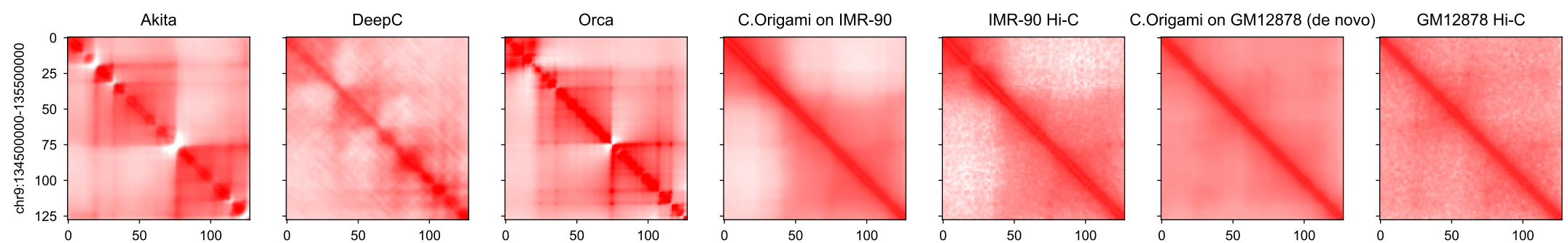

Supplement: Supplementary file 3 — Cell-type-specific predictions. [file 41587_2022_1612_MOESM3_ESM.zip › Cell type-specific predictions/chr9_135000000.pdf]

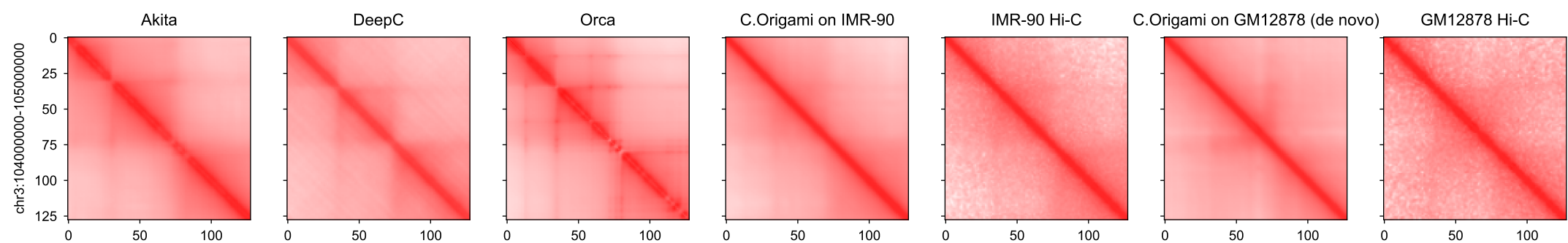

Supplement: Supplementary file 3 — Cell-type-specific predictions. [file 41587_2022_1612_MOESM3_ESM.zip › Cell type-specific predictions/chr3_104500000.pdf]

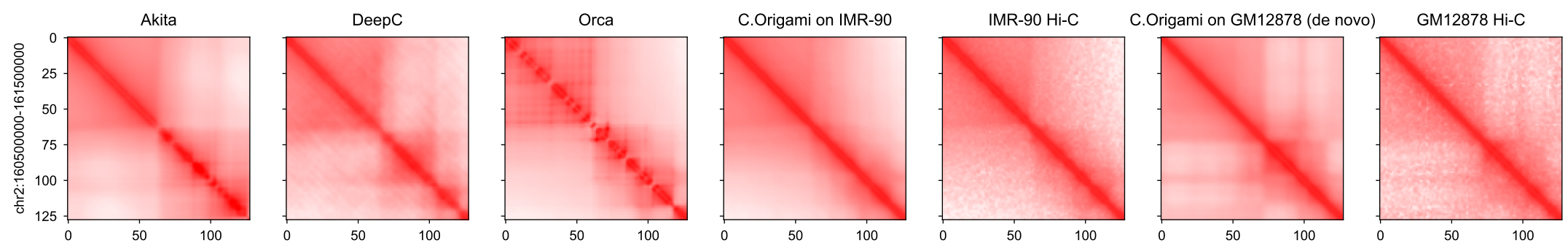

Supplement: Supplementary file 3 — Cell-type-specific predictions. [file 41587_2022_1612_MOESM3_ESM.zip › Cell type-specific predictions/chr2_161000000.pdf]

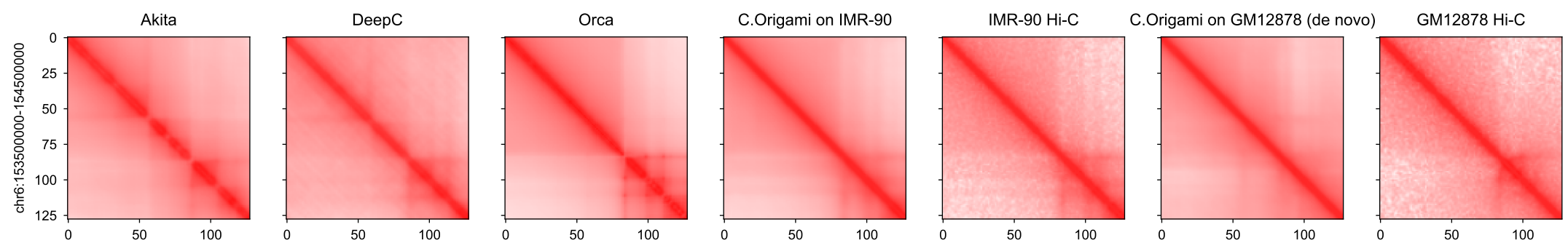

Supplement: Supplementary file 3 — Cell-type-specific predictions. [file 41587_2022_1612_MOESM3_ESM.zip › Cell type-specific predictions/chr6_154000000.pdf]

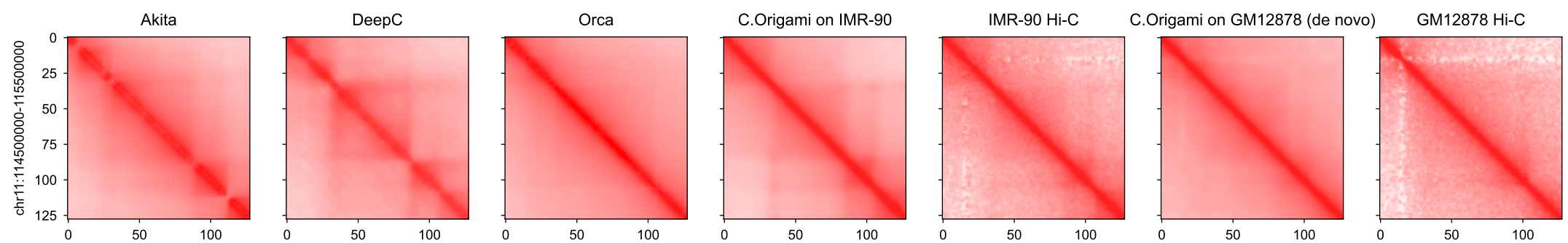

Supplement: Supplementary file 3 — Cell-type-specific predictions. [file 41587_2022_1612_MOESM3_ESM.zip › Cell type-specific predictions/chr11_115000000.pdf]

chrX:30500000-31500000

Akita

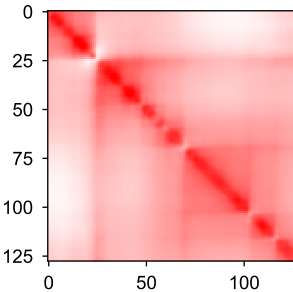

DeepC

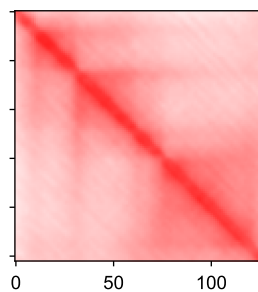

Orca

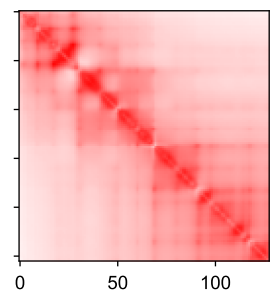

C.Origami on IMR-90

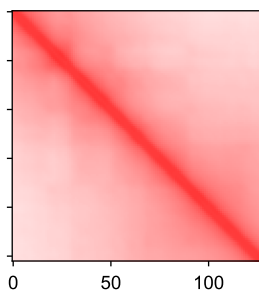

IMR-90 Hi-C

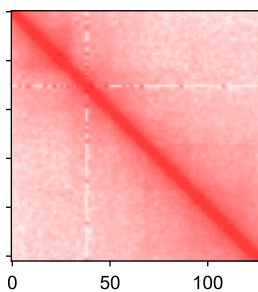

C.Origami on GM12878 (de novo)

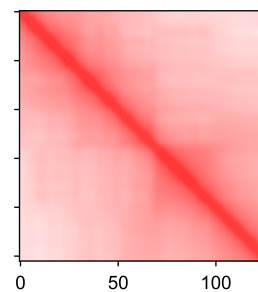

GM12878 Hi-C

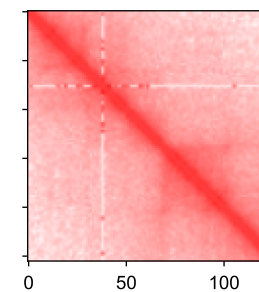

Supplement: Supplementary file 3 — Cell-type-specific predictions. [file 41587_2022_1612_MOESM3_ESM.zip › Cell type-specific predictions/chrX_31000000.pdf]

chr2:66000000-67000000

Akita

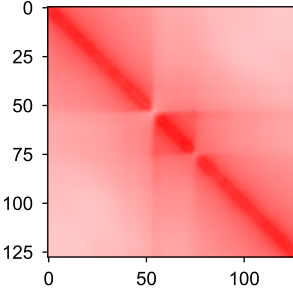

DeepC

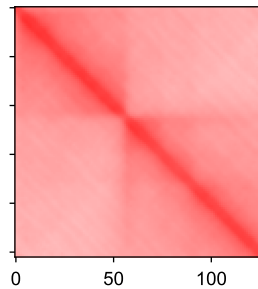

Orca

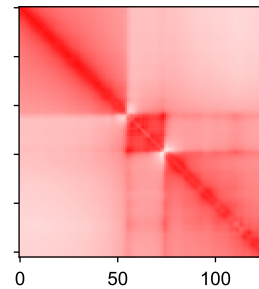

C.Origami on IMR-90

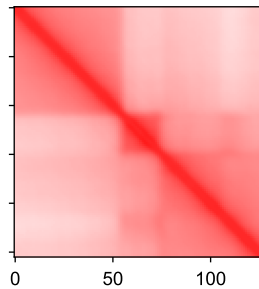

IMR-90 Hi-C

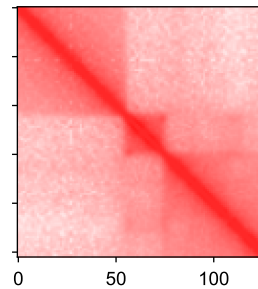

C.Origami on GM12878 (de novo)

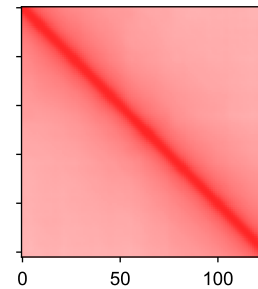

GM12878 Hi-C

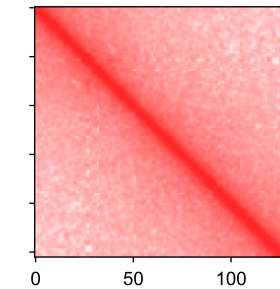

Supplement: Supplementary file 3 — Cell-type-specific predictions. [file 41587_2022_1612_MOESM3_ESM.zip › Cell type-specific predictions/chr2_66500000.pdf]

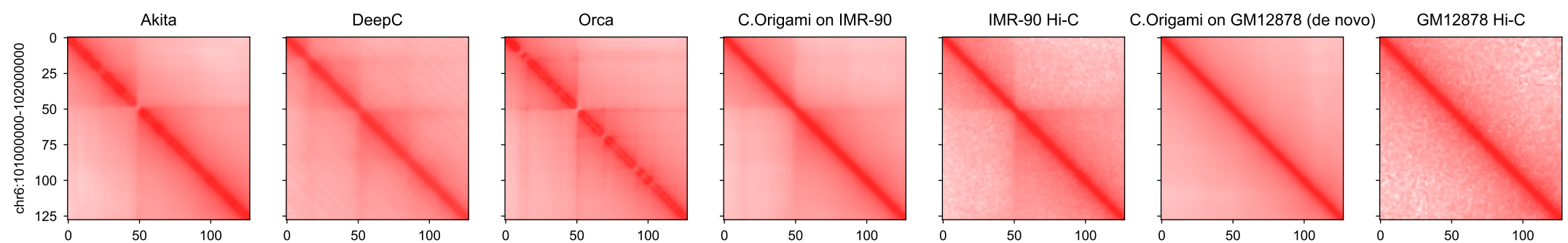

Supplement: Supplementary file 3 — Cell-type-specific predictions. [file 41587_2022_1612_MOESM3_ESM.zip › Cell type-specific predictions/chr6_101500000.pdf]

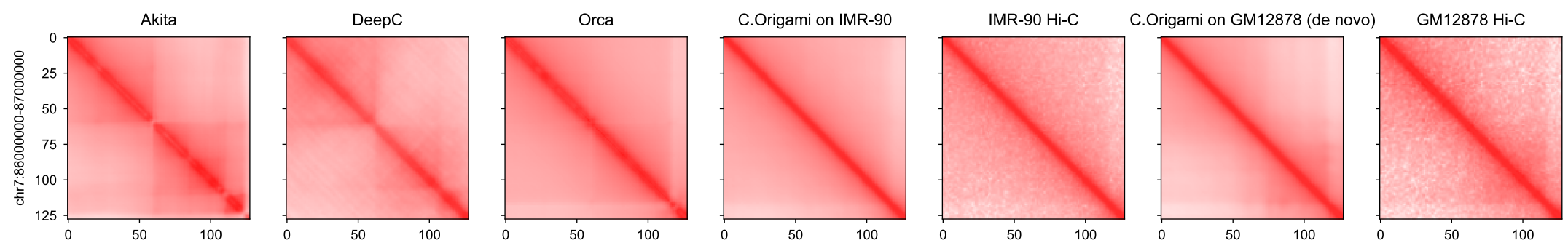

Supplement: Supplementary file 3 — Cell-type-specific predictions. [file 41587_2022_1612_MOESM3_ESM.zip › Cell type-specific predictions/chr7_86500000.pdf]

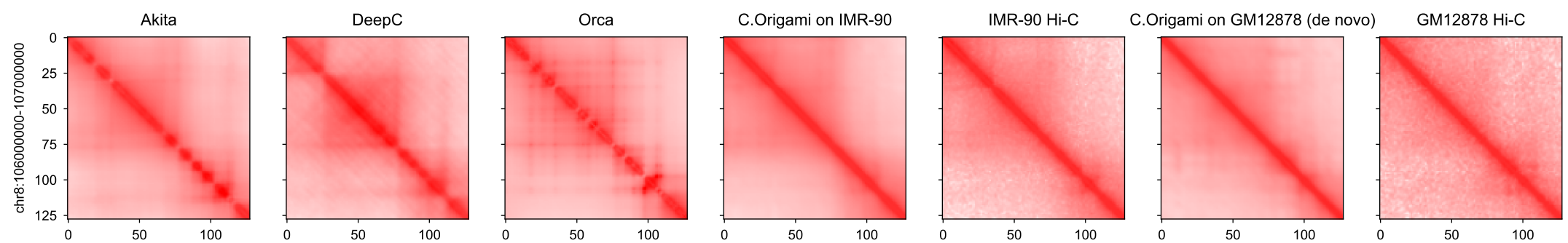

Supplement: Supplementary file 3 — Cell-type-specific predictions. [file 41587_2022_1612_MOESM3_ESM.zip › Cell type-specific predictions/chr8_106500000.pdf]

chr3:18500000-19500000

Akita

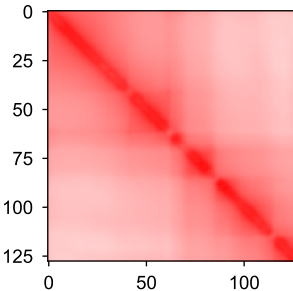

DeepC

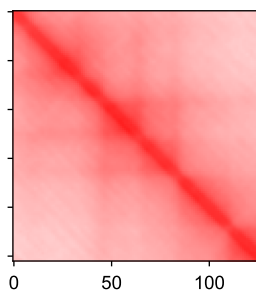

Orca

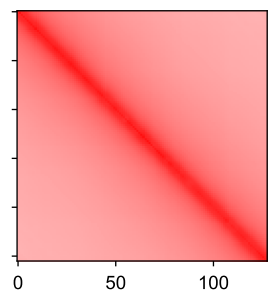

C.Origami on IMR-90

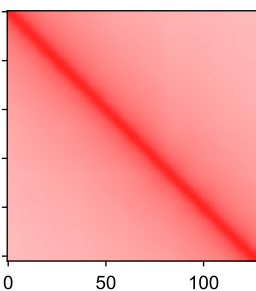

IMR-90 Hi-C

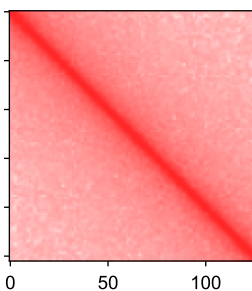

C.Origami on GM12878 (de novo)

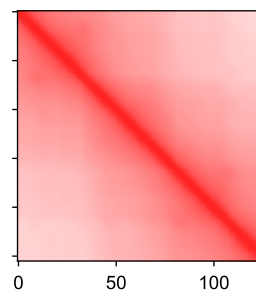

GM12878 Hi-C

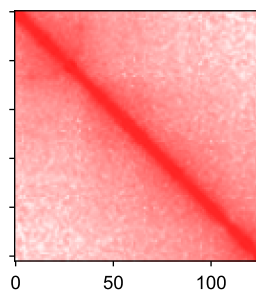

Supplement: Supplementary file 3 — Cell-type-specific predictions. [file 41587_2022_1612_MOESM3_ESM.zip › Cell type-specific predictions/chr3_19000000.pdf]
