# Supplementary material for: Cell-type-specific prediction of 3D chromatin organization enables high-throughput in silico genetic screening
Source: Nat Biotechnol. Author manuscript; Available in PMC 2023 Aug 24. (PMC10329734; doi:10.1038/s41587-022-01612-8)
Supplement: Supplementary file 1 [file NIHMS1879256-supplement-Supplementary_Figures.pdf]

Supplementary information

Cell Type	Enzyme	Accession Number	Reference
IMR-90	MboI	GSE63525	Rao *et al*[32]
GM12878	MboI	GSE63525	Rao *et al*[32]
H1-hESC	MboI	4DNESFSCP5L8	Calandrelli *et al*[64]
K562	MboI	GSE63525	Rao *et al*[32]
CUTLL1	Arima	GSE115896	Kloetgen *et al*[30]
T cell	Arima	GSE115896	Kloetgen *et al*[30]
Patski (Mouse)	Arima	GSE71831	Darrow *et al*[45]
ESC (Mouse)	HindIII	GSE98671	Nora *et al*[38]

Supplementary Table 1: Hi-C data used for training and validation.

Cell Type	CTCF ChIP-seq	ATAC-seq
IMR-90	ENCSR000EFI	ENCSR200OML
GM12878	ENCSR000AKB	ENCSR095QNB
H1-hESC	ENCSR000AMF	GSE85330
K562	ENCSR000AKO	ENCSR483RKN
CUTLL1	GSE115893	GSE216430
Jurkat	GSE115893	GSE90718
T cell	GSE115893	GSE101498
Patski (Mouse)	ENCSR419OOD	ENCSR351QUO
ESC (Mouse)	GSE98671	N.A.

Supplementary Table 2: CTCF ChIP-seq and ATAC-seq used for training and validation.

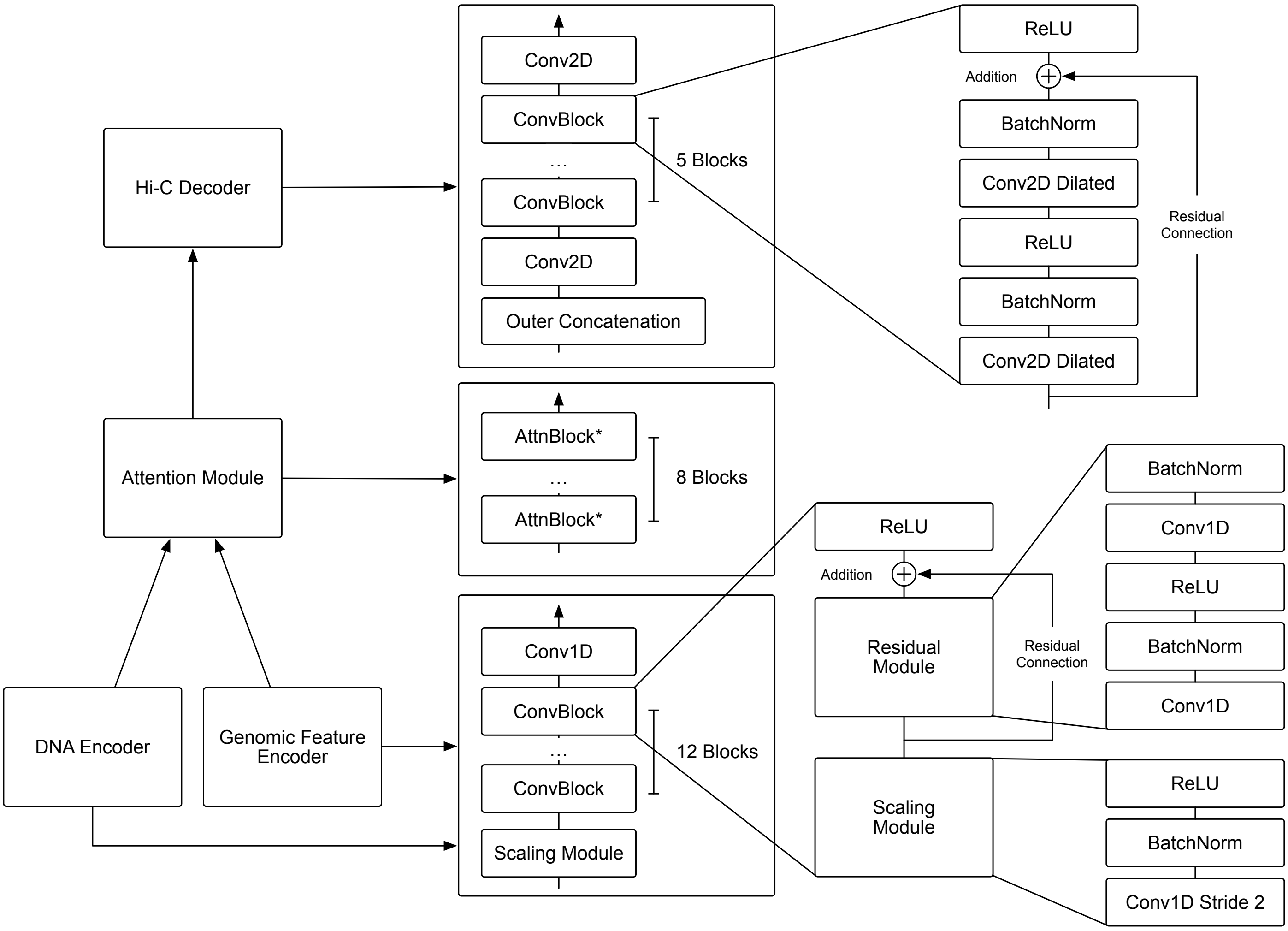

Supplementary Figure 1: C.Origami model structure and module components. A schematic of C.Origami model architecture. The DNA encoder and Genomic Feature encoder have similar architectures and only different in input channels where DNA encoder has 5 channels and feature encoder has 2 channels. We built the encoder with 12 convolution blocks. Each block consists of a scaling module and a residual module. The scaling module downscales input features by a factor of two with a stride-2 1D convolution layer. The residual module promotes information propagation in very deep networks[56]. The number of modules was carefully chosen so that the 2,097,152 input are scaled down to 256 bins at the end of the encoder. To enhance interactions within the 2Mb window, we used an attention module consisting of eight attention blocks. Each position of the output is concatenated with every other position to form a 2D matrix, resembling a vector outer product process. To refine the final prediction, we used a 5-layer dilated 2D convolutional network as decoder. We deliberately chose the dilation parameters to ensure that every position at the last layer has a receptive field covering the input range.

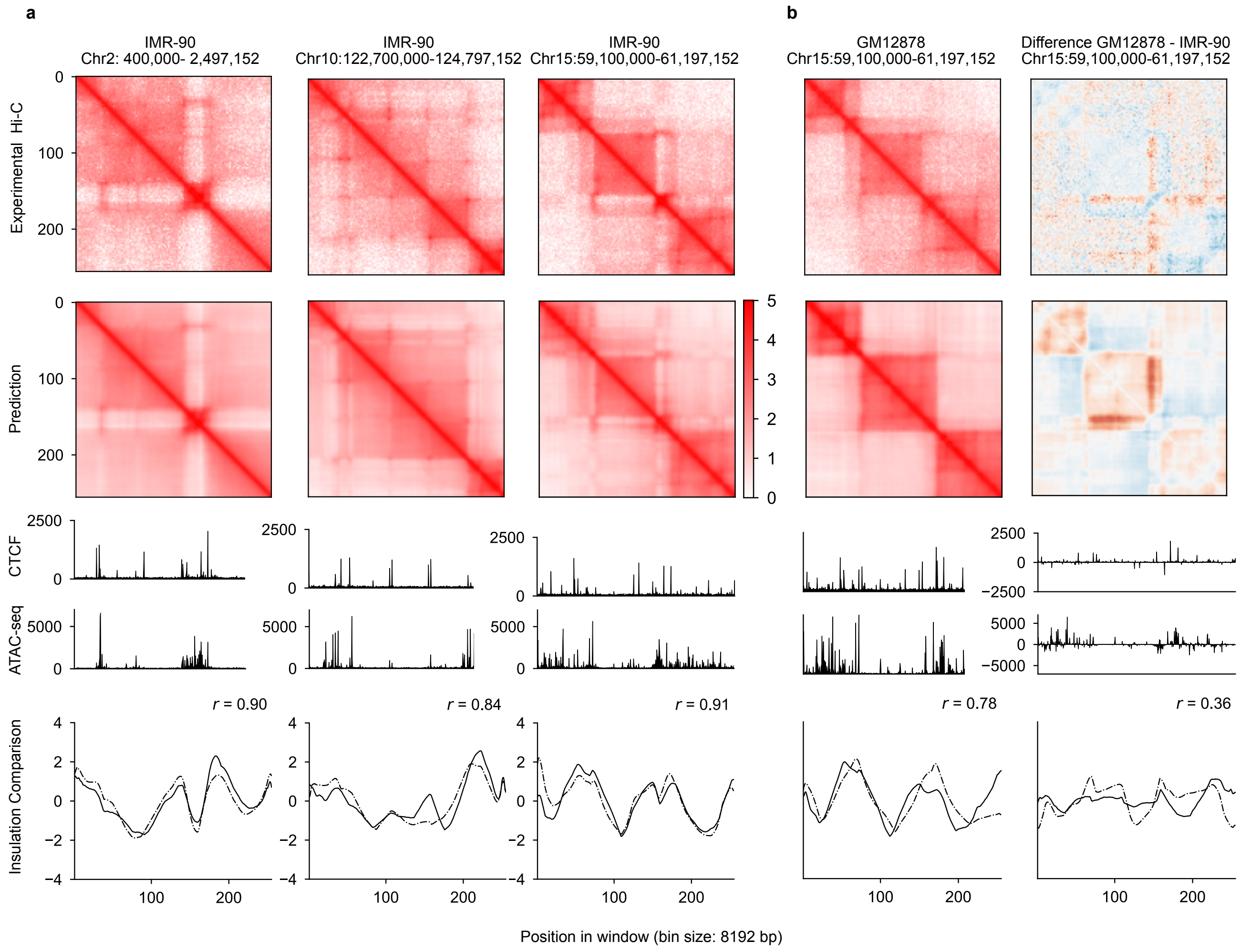

Supplementary Figure 2: Performance of C.Origami trained with DNA sequence and CTCF ChIP-seq. a, Prediction from a model trained with DNA sequence and CTCF ChIP-seq. The plots were organized the same way as Fig. 2. **b**, De novo predicting chromatin organization of the chromosome 15 locus in GM12878 using the model trained with DNA sequence and CTCF binding profiles. The difference between IMR-90 and GM12878 is presented on the right. While C.Origami trained with DNA sequence and CTCF profile achieved good performance in validation and test set in IMR-90 (**a**), it missed predicting some fine-scale chromatin structures in GM12878.

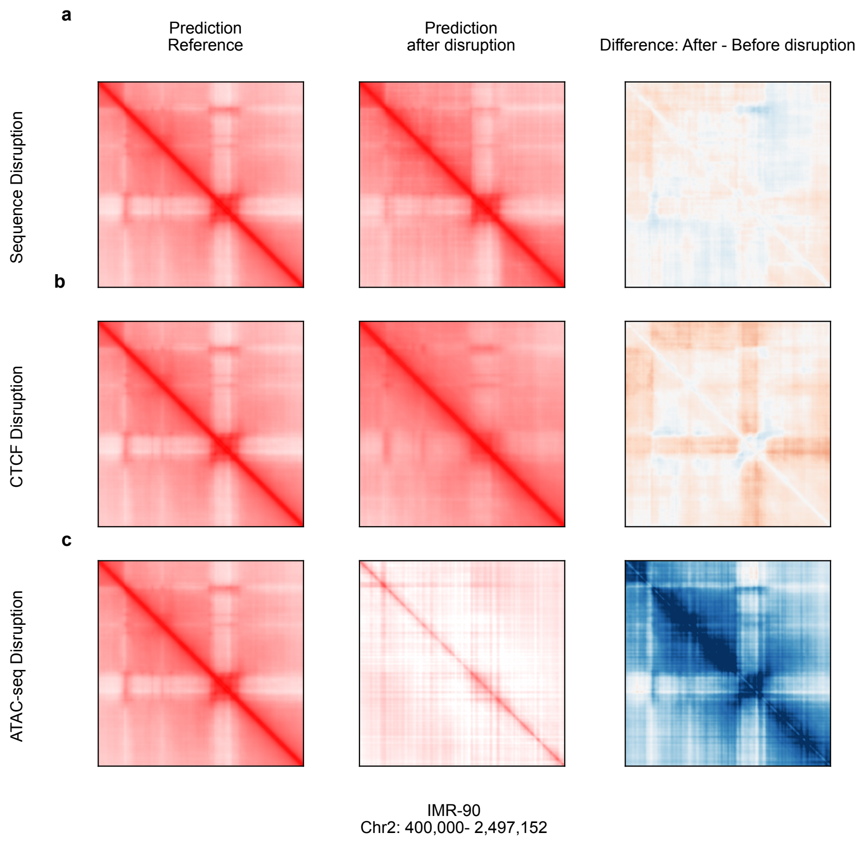

Supplementary Figure 3: Ablation study on different input features. Using C.Origami trained with DNA sequence, CTCF binding, and chromatin accessibility profiles, the experiments were performed by random shuffling DNA sequences at base pair level (**a**), random shuffling CTCF signal (**b**), and random shuffling ATAC-seq signal (**c**). From left to right, reference prediction with all inputs (left), prediction with sequence shuffled (middle), difference between perturbed prediction and reference prediction (right).

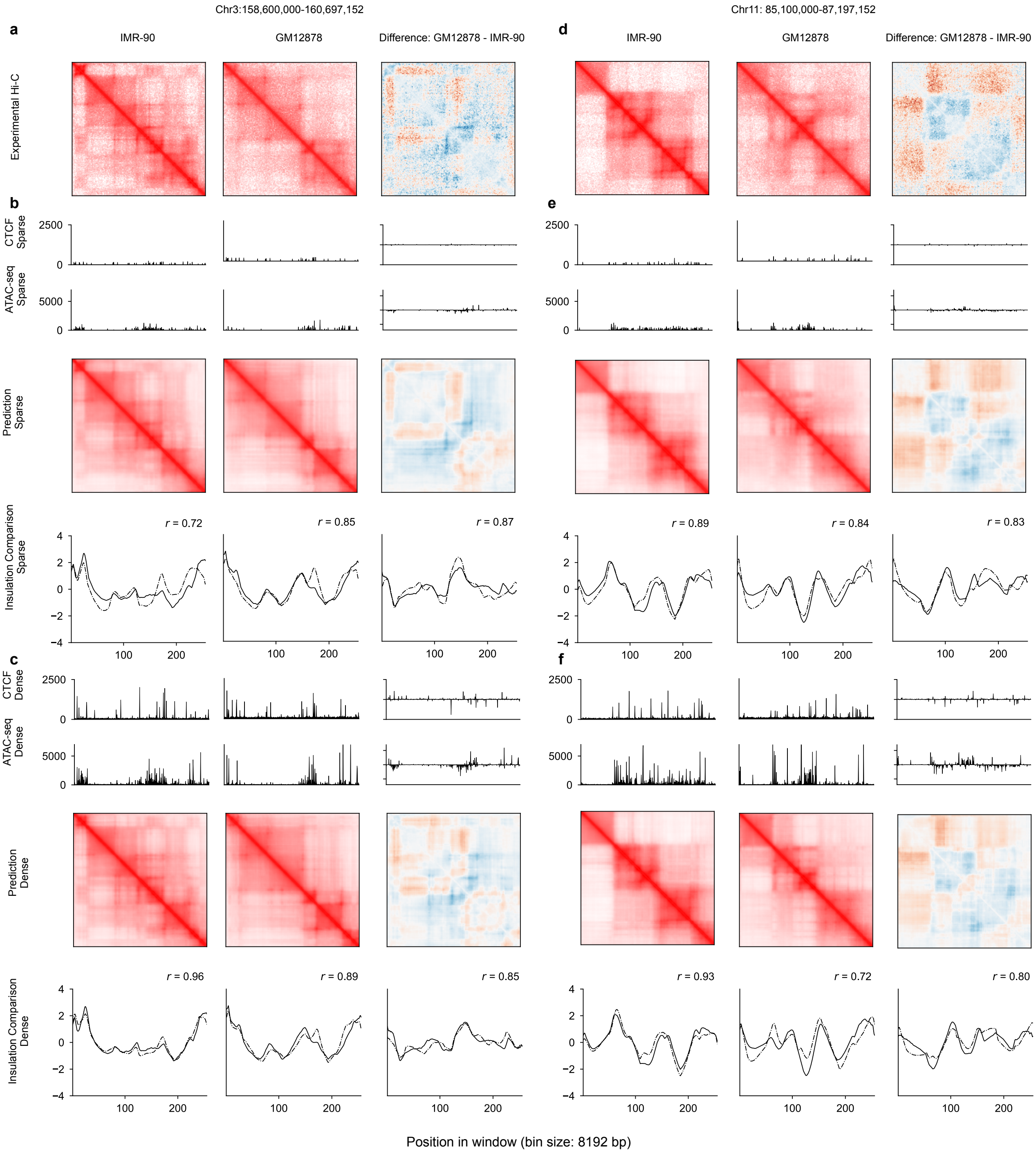

Supplementary Figure 4: Performance comparison of C.Origami models trained with sparse information and dense information. **a**, Experimental Hi-C matrices of IMR-90 and GM12878 cells at chr3: 158,600,000-160,697,152. The difference between the two cell lines were presented on the right. **b-c**, Cell type-specific prediction of the chromatin organization at the same locus using C.Origami models trains with sparse genomic information (**b**) or dense genomic information (**c**). For each set of plots in **b** and **c**, the input CTCF ChIP-seq and ATAC-seq profiles were aligned with the predicted Hi-C matrices and the insulation score results. **d-f**, Same as **a-c** at a difference locus, chr10: 85,100,000-87,197,152.

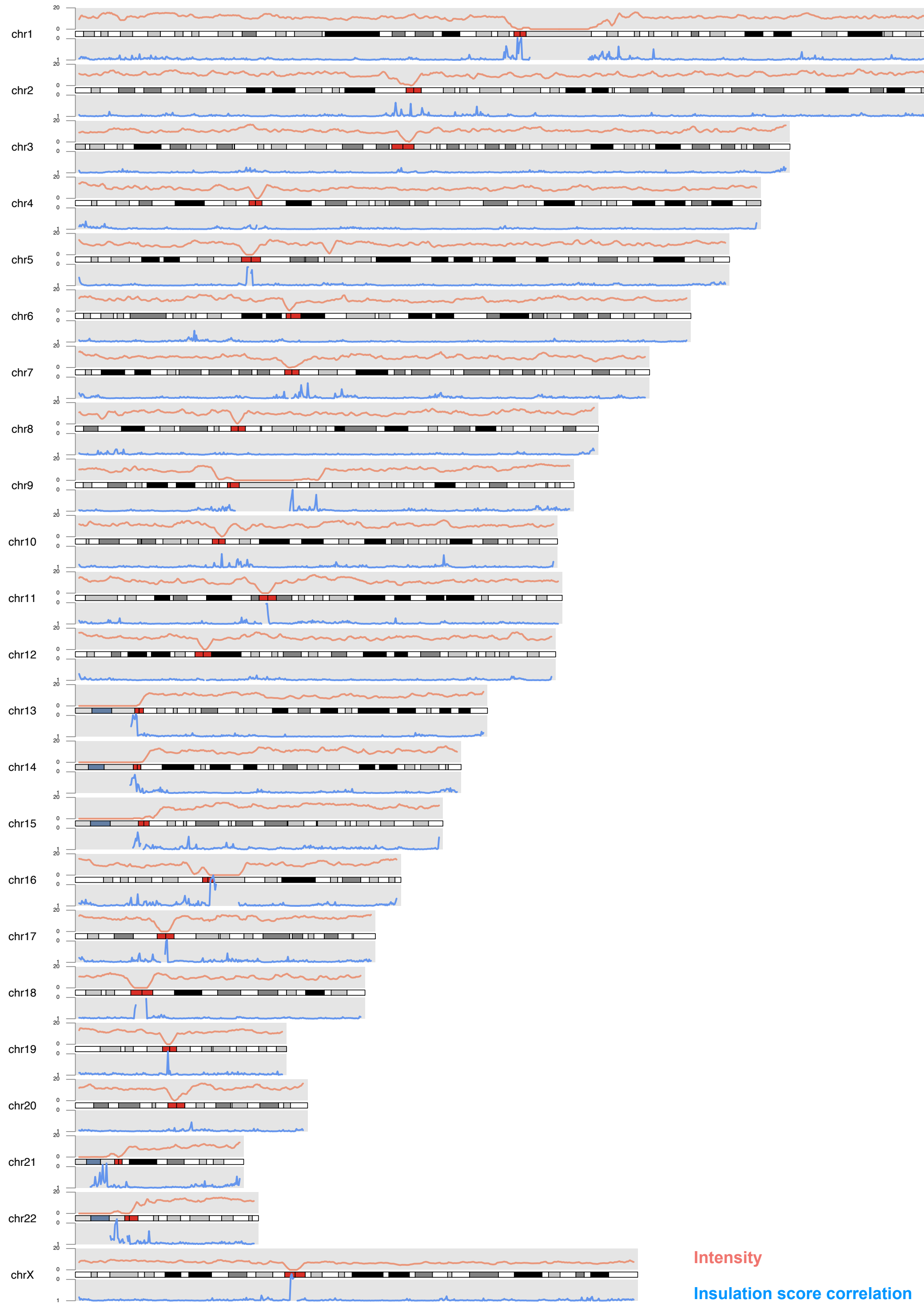

Supplementary Figure 5: Chromosome karyotype visualization along with chromosome-wide Hi-C intensity and correlation of insulation scores. The results were visualized using karyoploteR[57]. Chromosome 1 to chromosome X were plotted to visualize the Pearson correlation coefficients of insulation scores calculated from prediction and that from experimental Hi-C. Average intensity of 2Mb windows were plotted in red. Centromere regions were denoted with red segments on the genome. The few data points with low intensity are regions corresponding to unmappable or repeat sequences such as centromeres and telomeres.

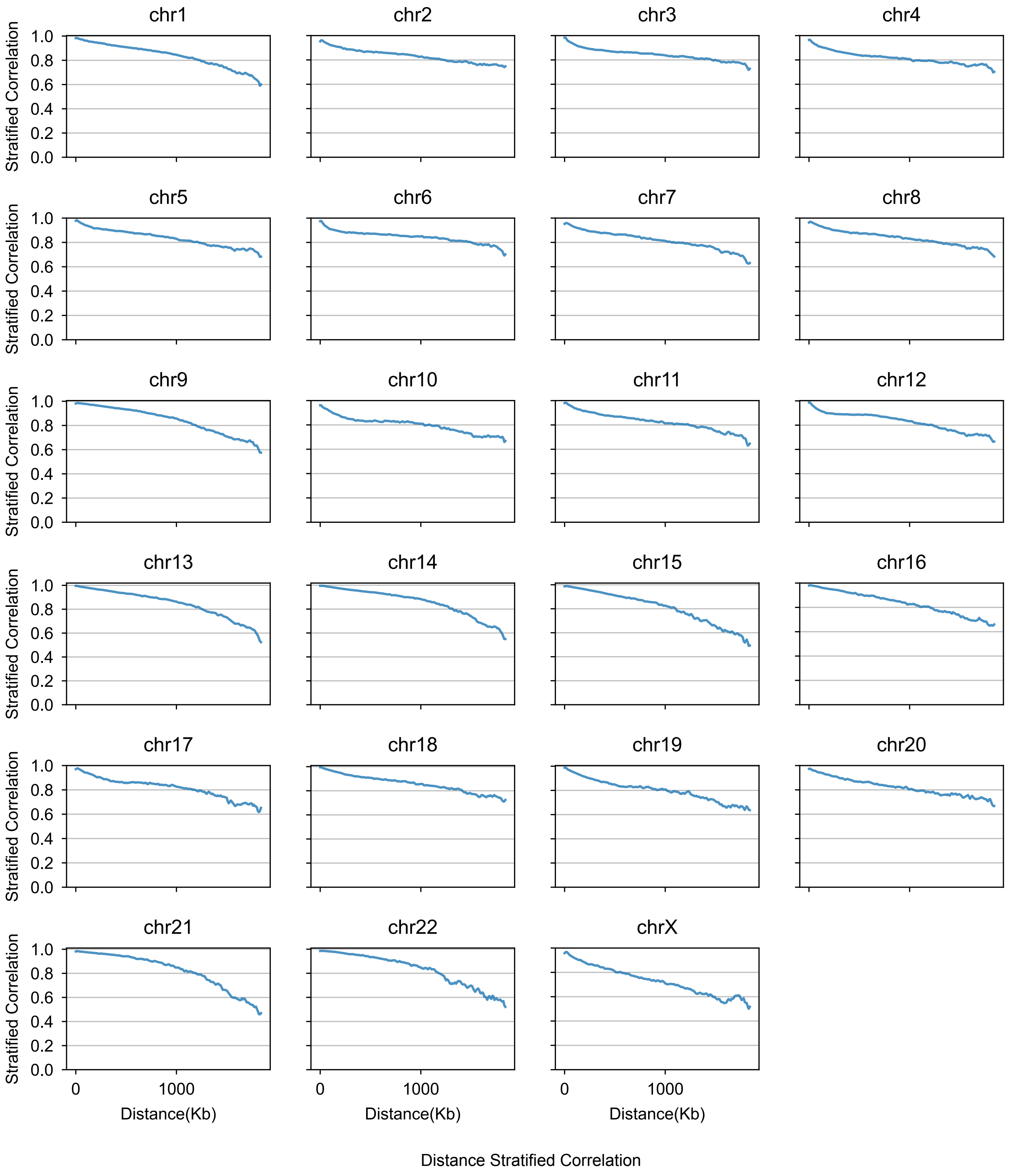

Supplementary Figure 6: Chromosome-level distance-stratified intensity correlation. Interaction intensity distribution of prediction and experimental Hi-C on validation (chromosome 10) and test chromosome (chromosome 15). Chromosome-level distance-stratified correlation between prediction and experimental Hi-C were calculated on each chromosome of IMR-90 cells.

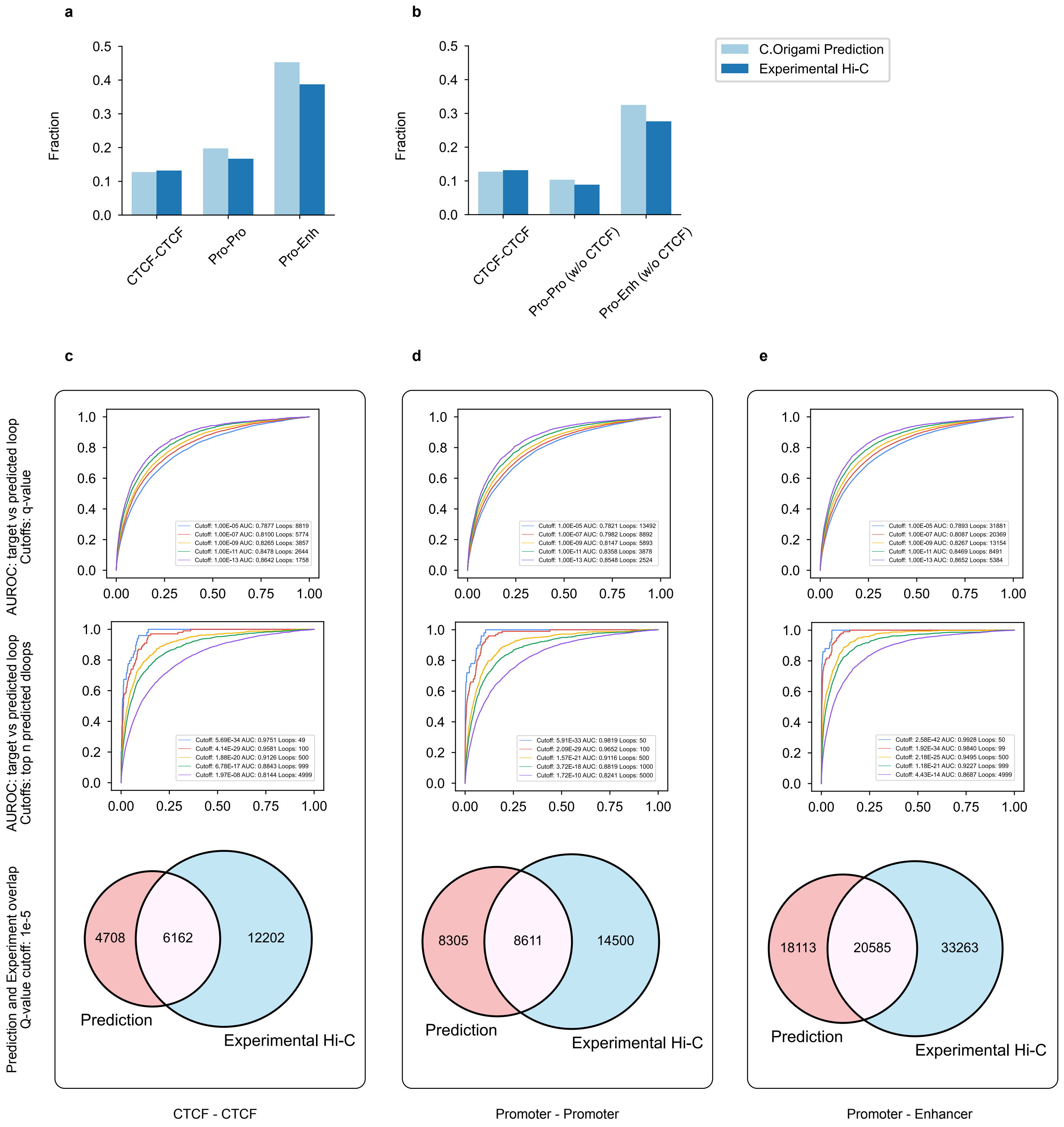

Supplementary Figure 7: Performance of detecting loop interactions under different chromatin backgrounds. **a-b**, Percentages of loop counts in three different categories, including CTCF-CTCF loop, promoter-promoter loop, and promoter-enhancer loop. Significant chromatin loop referring to global background were called at different q-value in IMR-90 cells and then categorized according to their anchor content. Within each panel, AUROC between loops from experiment and prediction was calculated with q-value cutoffs ranging from 1e-5 to 1e-13, similar to the previous loop analysis. Category counts were divided by the total number of loops called. **c-e**, ROC curves and the Venn diagrams of the significant chromatin loops called in experimental Hi-C and prediction categorized by anchor content: CTCF-CTCF loop (**c**), promoter-promoter loop (**d**), and promoter-enhancer loop (**e**). AUROC from top 50 to top 5000 loops were also plotted.

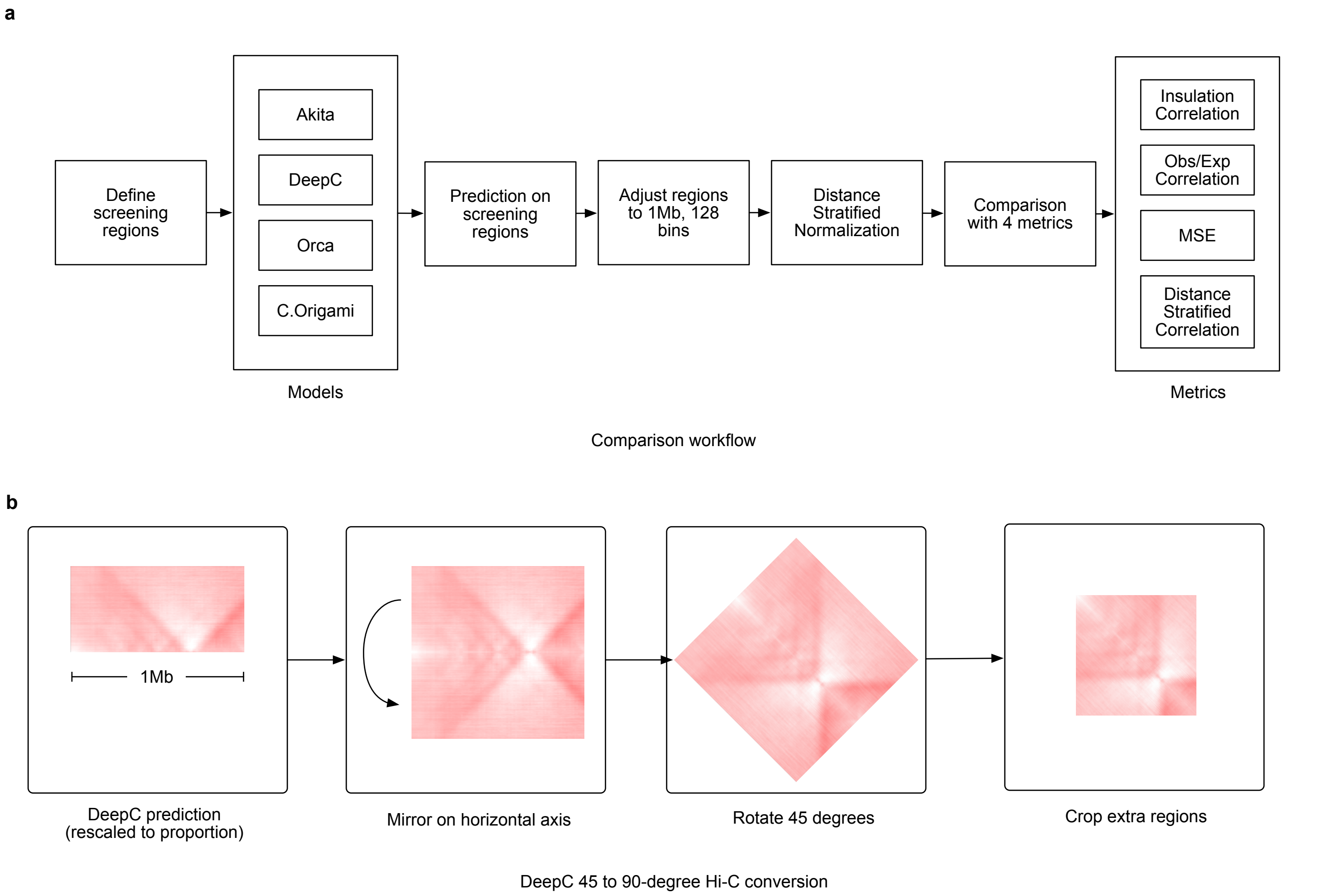

Supplementary Figure 8: Workflow of comparing performance of models predicting 3D chromatin organization. **a**, Workflow of the comparison procedures to standardize and evaluate the predictions from Akita, DeepC, Orca, and C.Origami. **b**, Post-processing of DeepC prediction results. DeepC method by default produces a 45 degree Hi-C map, thus requiring mirroring, rotation and cropping steps to make the results comparable to Hi-C targets.

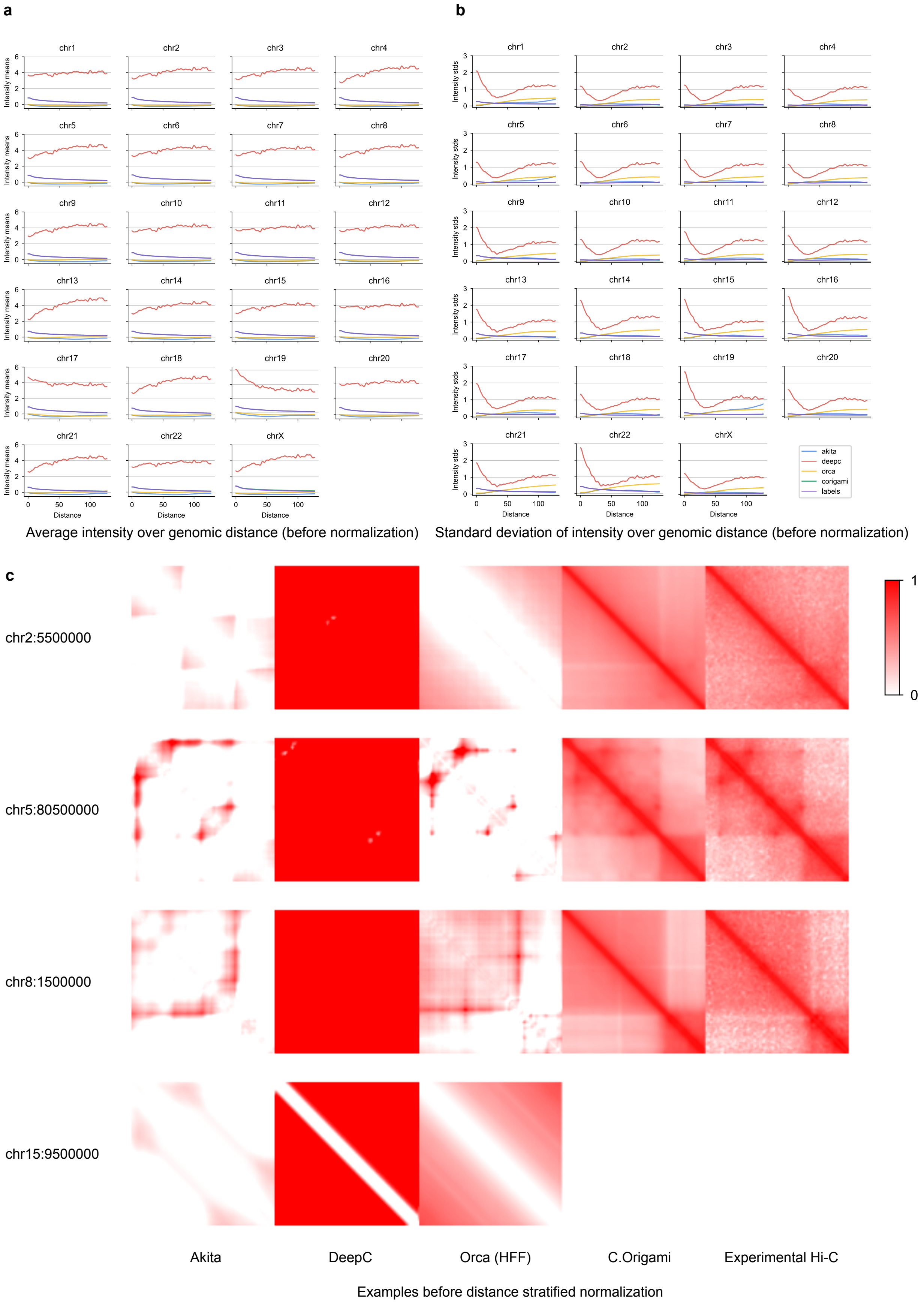

Supplementary Figure 9: Distance-stratified statistics of raw predictions results from the four models in comparison. a-b, Distance-stratified mean intensity (**a**) and standard deviation (**b**) of predicted Hi-C results from the four models. The horizontal axis denotes the rescaled 128 bins representing a 1Mb region. DeepC has a different distribution of intensities compared to the rest of the models. The abnormality could be a result of its custom percentile normalization on the training target. **c**. Raw prediction results from four models together with experimental Hi-C. Intensity values was set to be from 0 to 1 according to experimental Hi-C data.

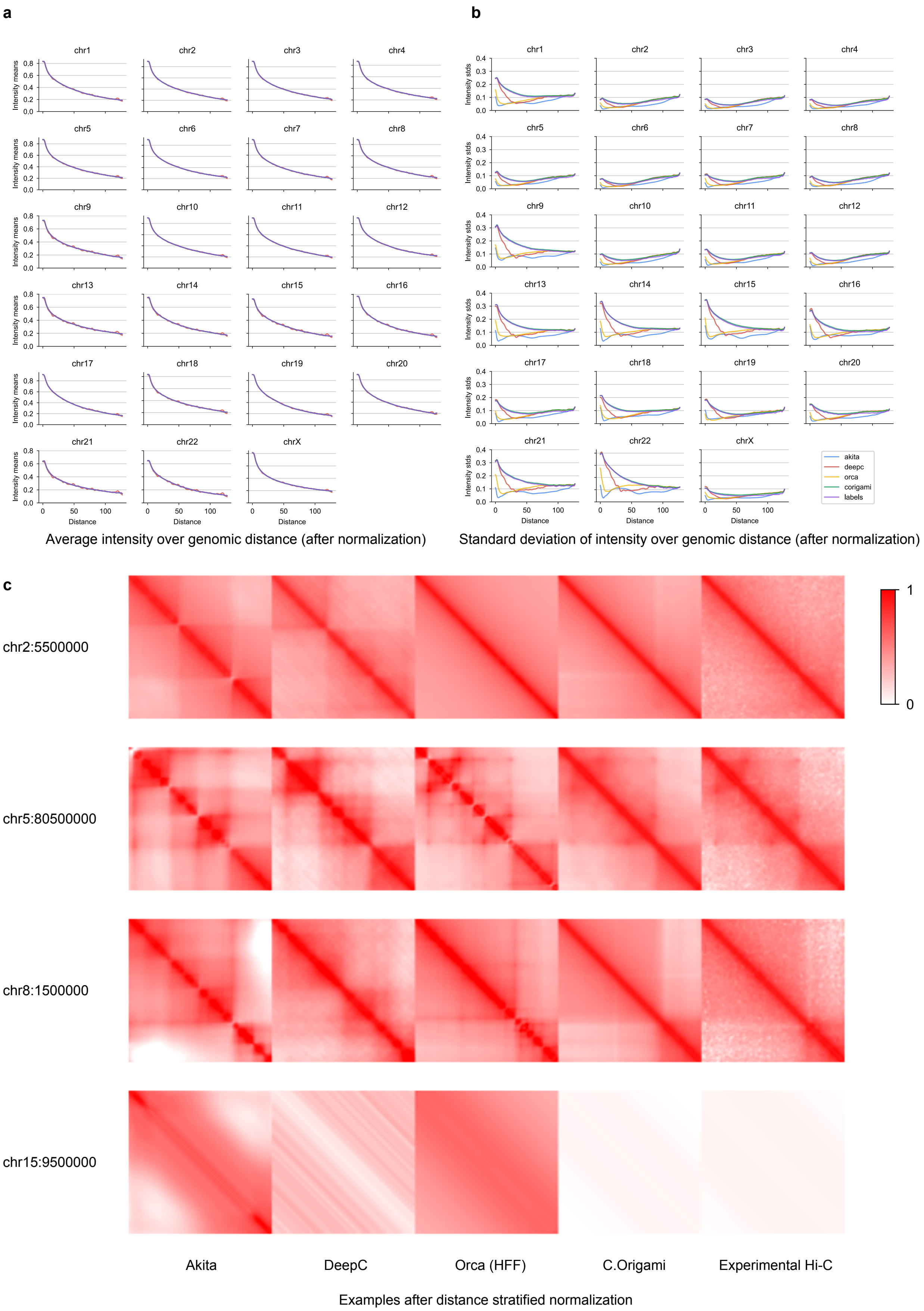

Supplementary Figure 10: Distance-stratified statistics of prediction results after standardization. a-b, Distance-stratified mean intensity (**a**) and standard deviation (**b**) of predicted Hi-C results from the four models after distance-stratified normalization. After normalization, the differences between all model predictions are comparable to experimental Hi-C. **c**. Normalized prediction results from four models together with experimental Hi-C. Intensity values was set to be from 0 to 1 according to experimental Hi-C data. Presented loci are from the same regions as in Supplementary Figure 12. In comparison, normalized predictions are more comparable in between and closer to the experimental Hi-C.

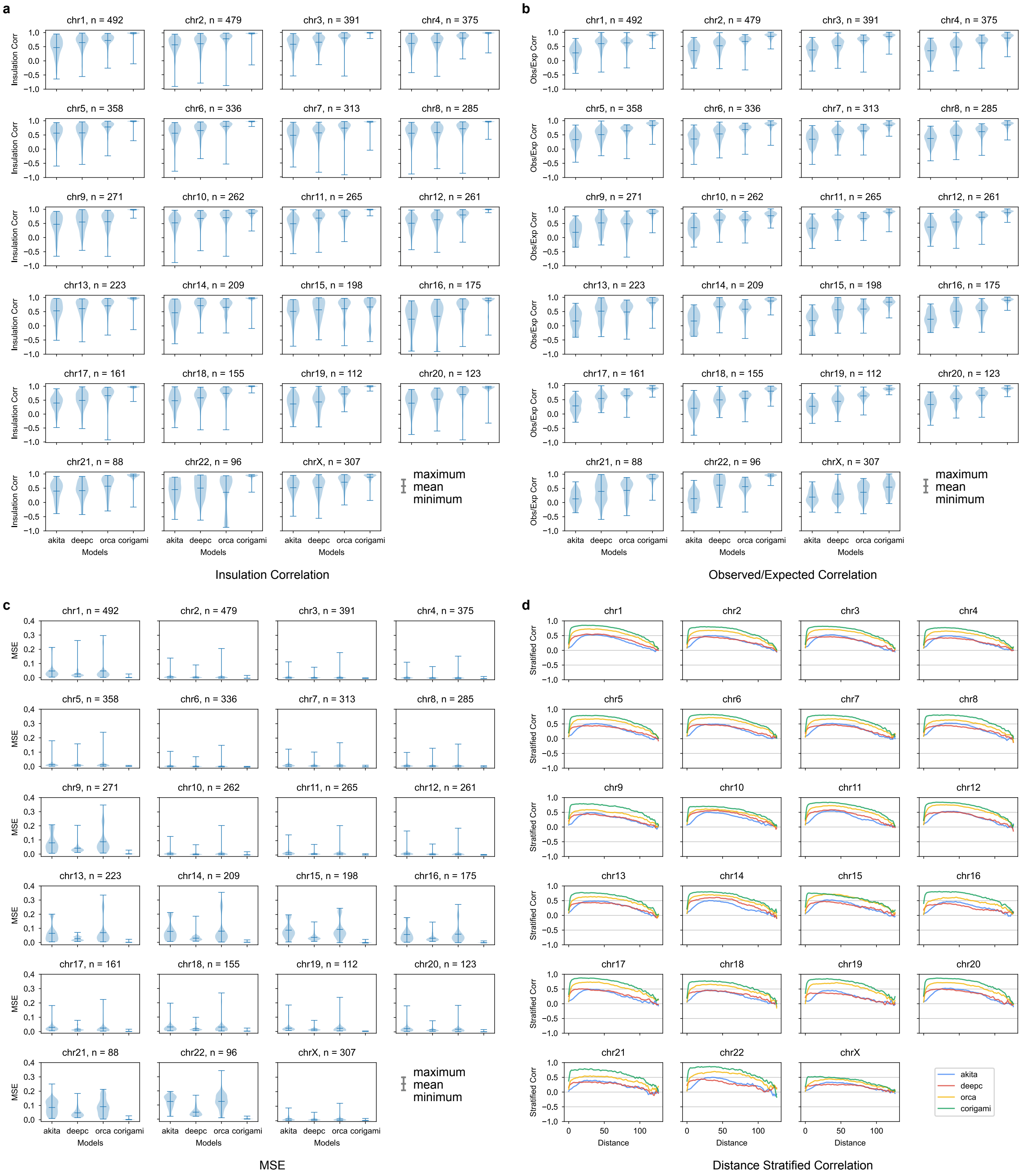

Supplementary Figure 11: Genome-wide comparison of model performance in IMR-90 cells. For predictions from each model (Akita, DeepC, Orca and C.Origami), we measured insulation score correlation (**a**), observed vs expected Hi-C matrices correlation (**b**), mean squared error (MSE, **c**), and distance-stratified correlation (**d**). Error bars in the violin plots indicate minimum, mean and maximum values within each group.

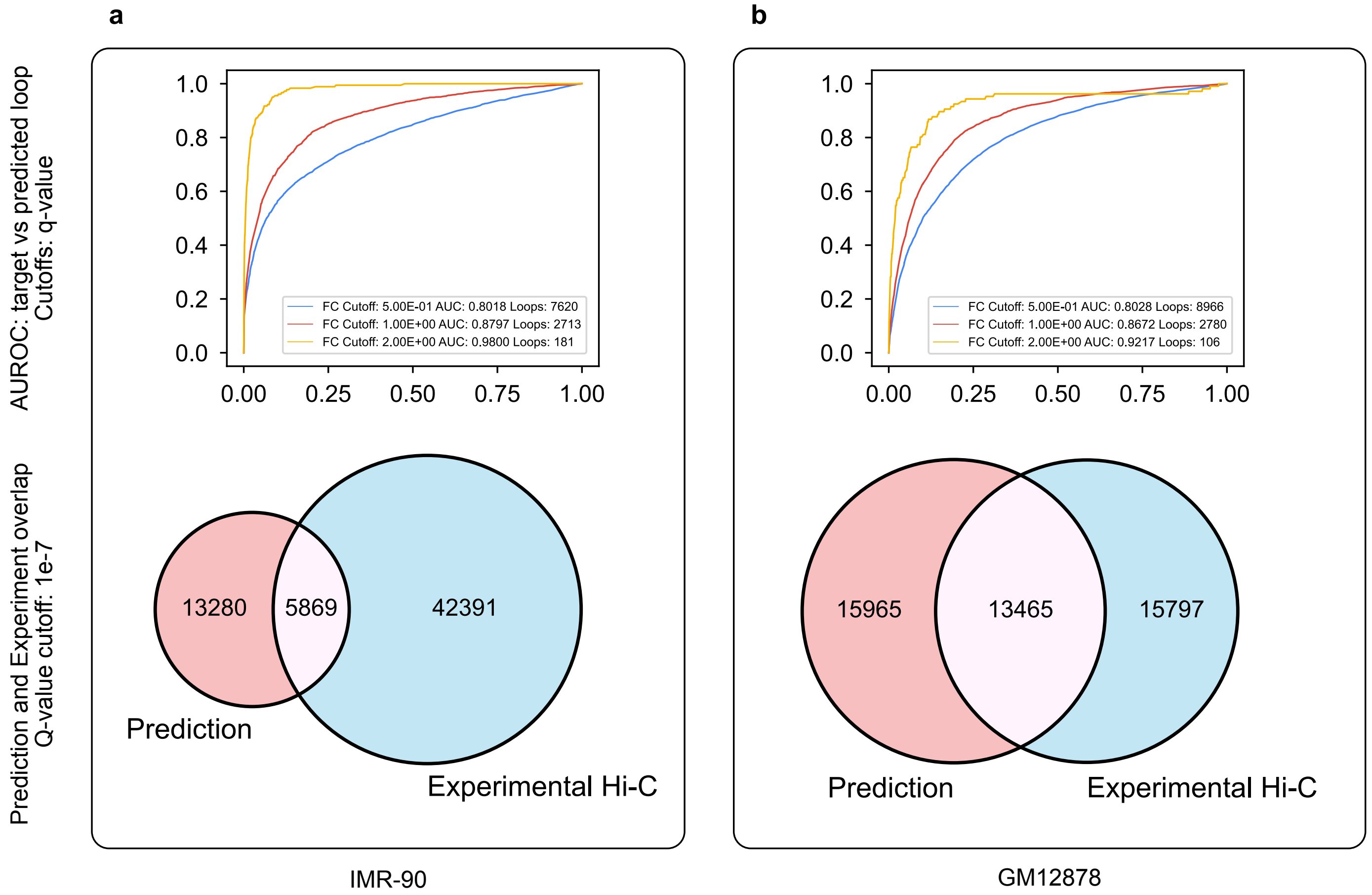

Supplementary Figure 12: Performance of detecting cell type-specific loop interactions between IMR-90 and GM12878. a-b, Comparing cell type-specific loops between prediction and experiment in IMP-90 (**a**) and GM12878 cells (**b**). Loops detected from prediction were first filtered with a more stringent q-value cutoff of 1e-7 in both cell types. We then calculated cell type-specific loops according to signal value fold change. Within each panel, AUROC between loops from experiment and prediction was calculated with log2 fold change cutoffs ranging from 0.5 to 2. Overlap between loops called from prediction and experimental data is presented in a Venn diagram with a q-value cutoff of 1e-7.

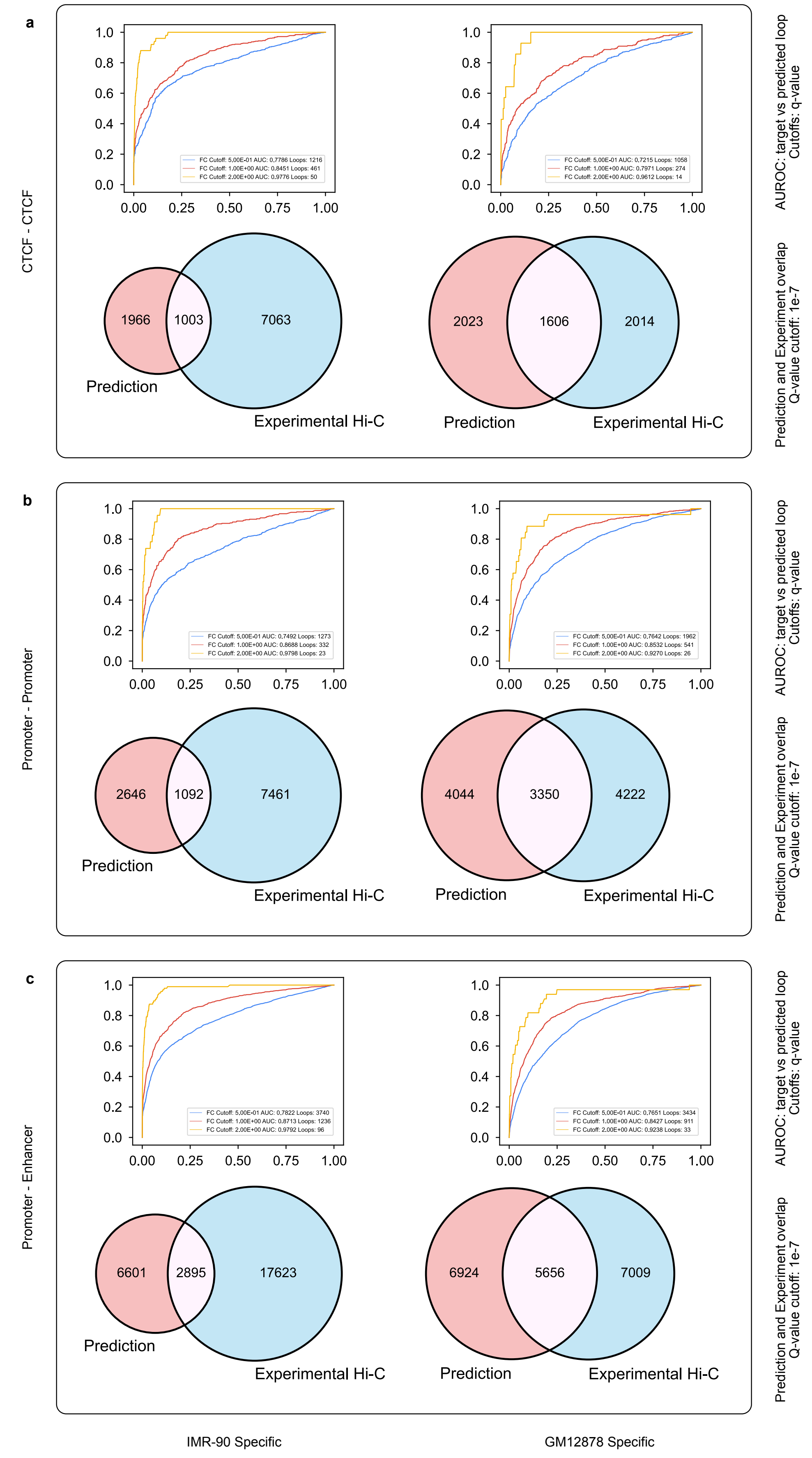

Supplementary Figure 13: Performance of detecting cell type-specific loop interactions between IMR-90 and GM12878 under different chromatin backgrounds. a-c, Evaluating cell type-specific loop detection performance in three types of loops: CTCF-CTCF loop (**a**), promoter-promoter loop (**b**), and promoter-enhancer loop (**c**). Loops were first filtered with a stringent q-value cutoff of 1e-7. We then calculated cell type-specific loops according to signal value fold change. Within each panel, AUROC between loops from experiment and prediction was calculated with log2 fold change cutoffs ranging from 0.5 to 2. Overlap between loops called from prediction and experimental data is presented in a Venn diagram with a q-value cutoff of 1e-7.

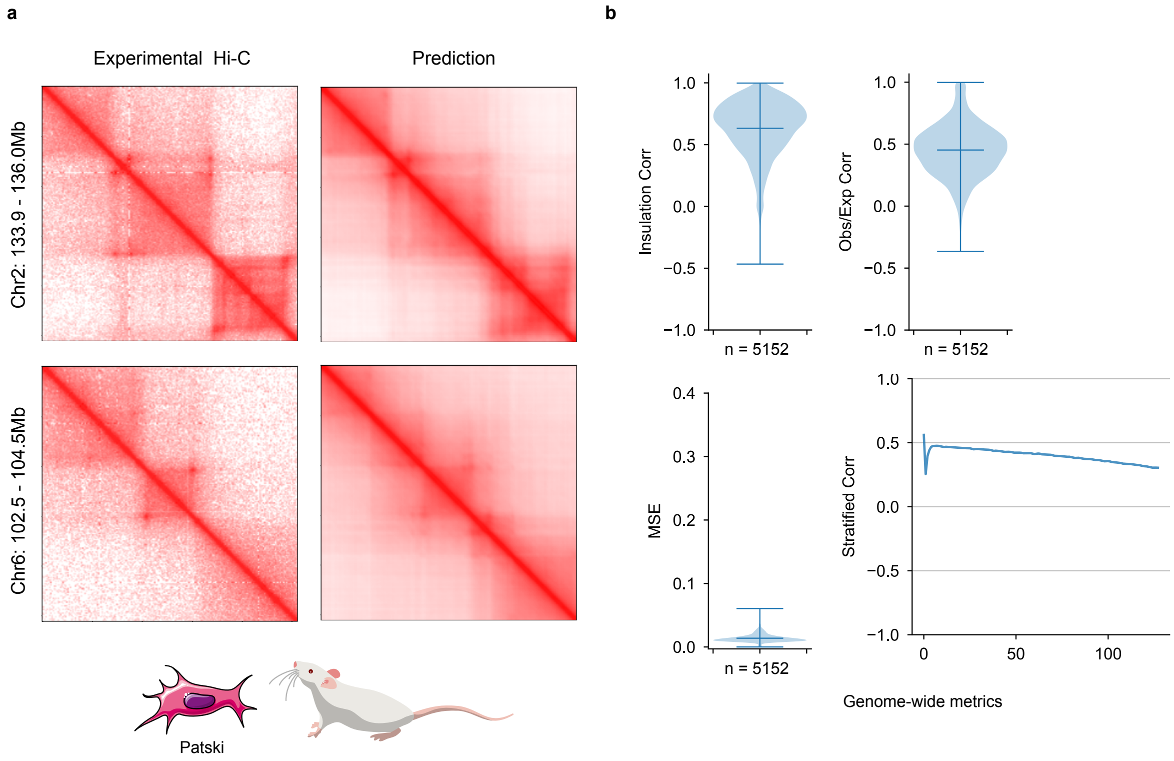

Supplementary Figure 14: Transferring model trained on human cell type to mouse. **a**, Experimental Hi-C and C.Origami prediction results of two representative loci in hybrid mouse Patski cells. **b**, Genome-wide performance metrics of predicting mouse chromatin organization using C.Origami trained with human data. Presented matrices include insulation score correlation, observed vs expected matrix correlation, mean squared error, and distance-stratified correlation. Error bars in the violin plots indicate minimum, mean and maximum values.

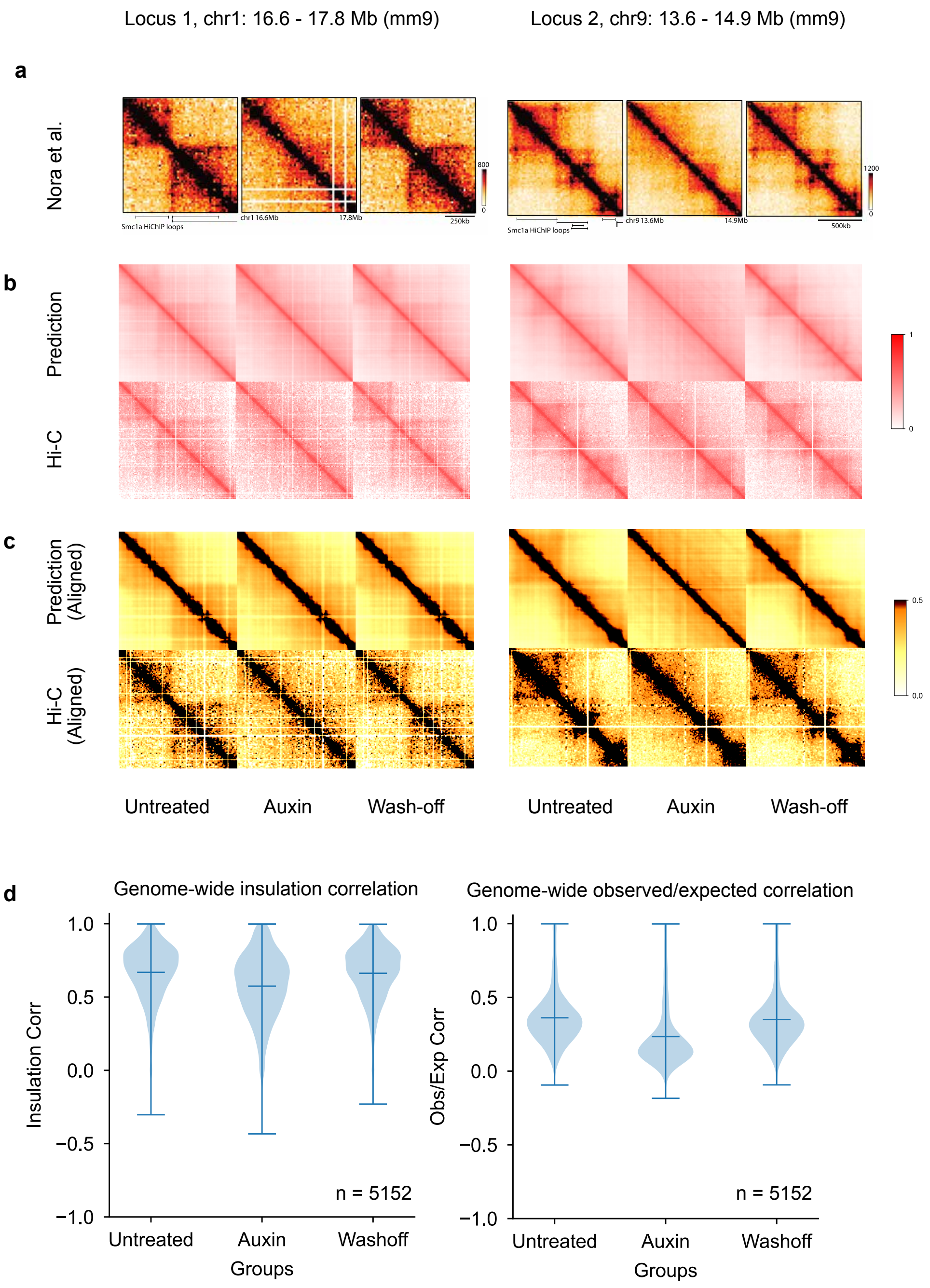

Supplementary Figure 15: Predicting chromatin organization dynamics upon auxin-induced CTCF depletion and restoration in mESCs. **a**, Experimental results adopted from Nora *et al*[32]. at two loci indicated on top. All plots were visualized in triplicates, indicating conditions of before CTCF depletion (Untreated), CTCF depleted (Auxin), and CTCF restored (Wash-off). **b**, C.Origami prediction at the corresponding 2Mb-wide windows using DNA sequence and CTCF ChIP-seq profiles from *Nora et al*[32]. Corresponding experimental Hi-C matrices from Nora et al. were processed by HiC-bench and visualized in parallel. **c**, Adjusted prediction and Hi-C matrices from b. Matrix size and location were adjusted to match the exact position from the experimental results as shown in **a**. Colormap was adjusted to match the original figure in Nora *et al*[32]. **d**, Genome-wide performance metrics for evaluating C.Origami prediction upon CTCF depletion and restoration. Presented correlations include insulation score (left panel) and observed vs expected matrix values (right panel). Error bars in the violin plots indicate minimum, mean and maximum values.

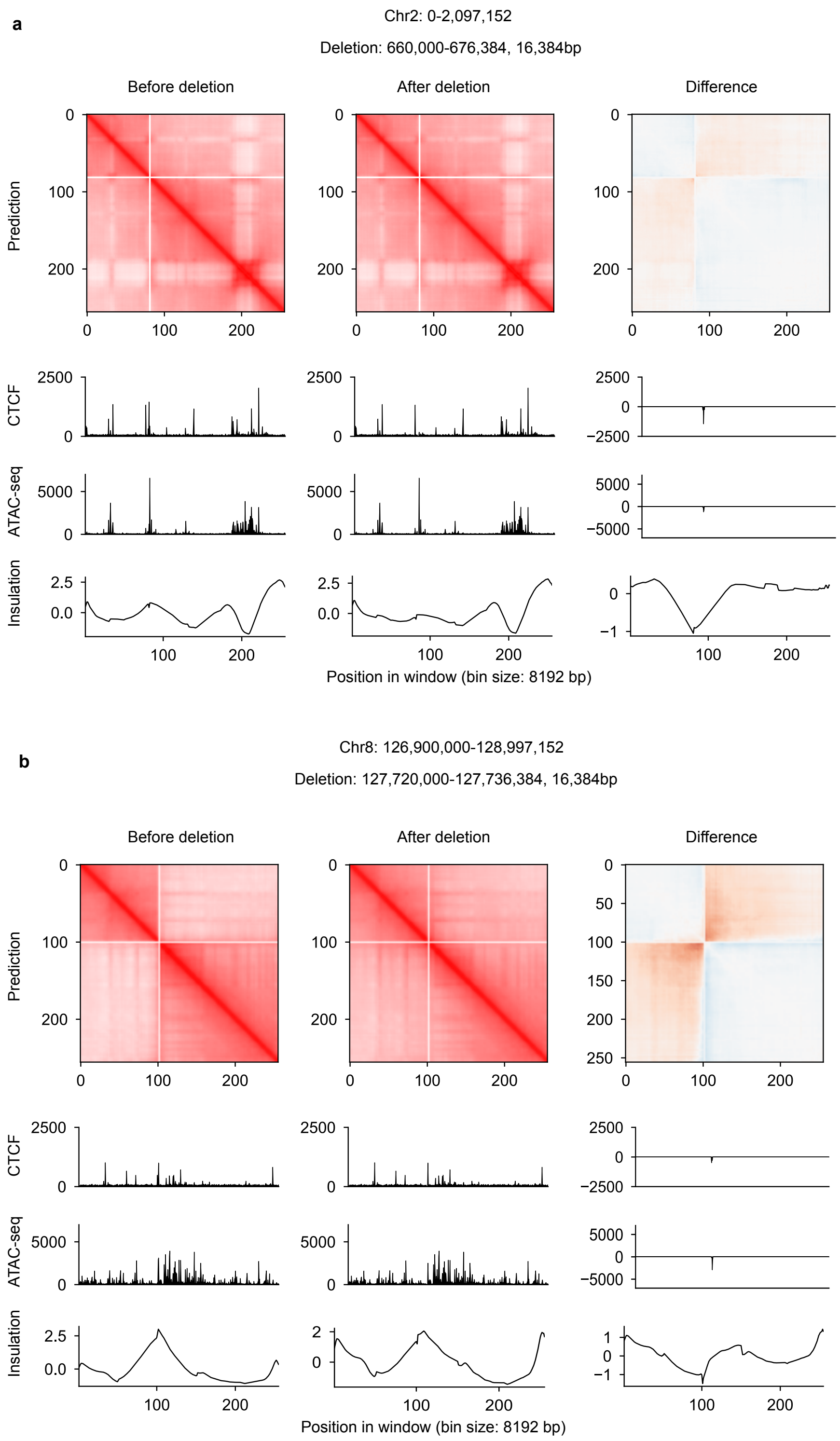

Supplementary Figure 16: *In silico* genetic experiments performed on IMR-90 cells. Two *in silico* deletion experiments were separately represented in **a** and **b**. Each experiment included the prediction before (left) and after deletion (middle). The difference in chromatin folding after deletion were presented on the right.

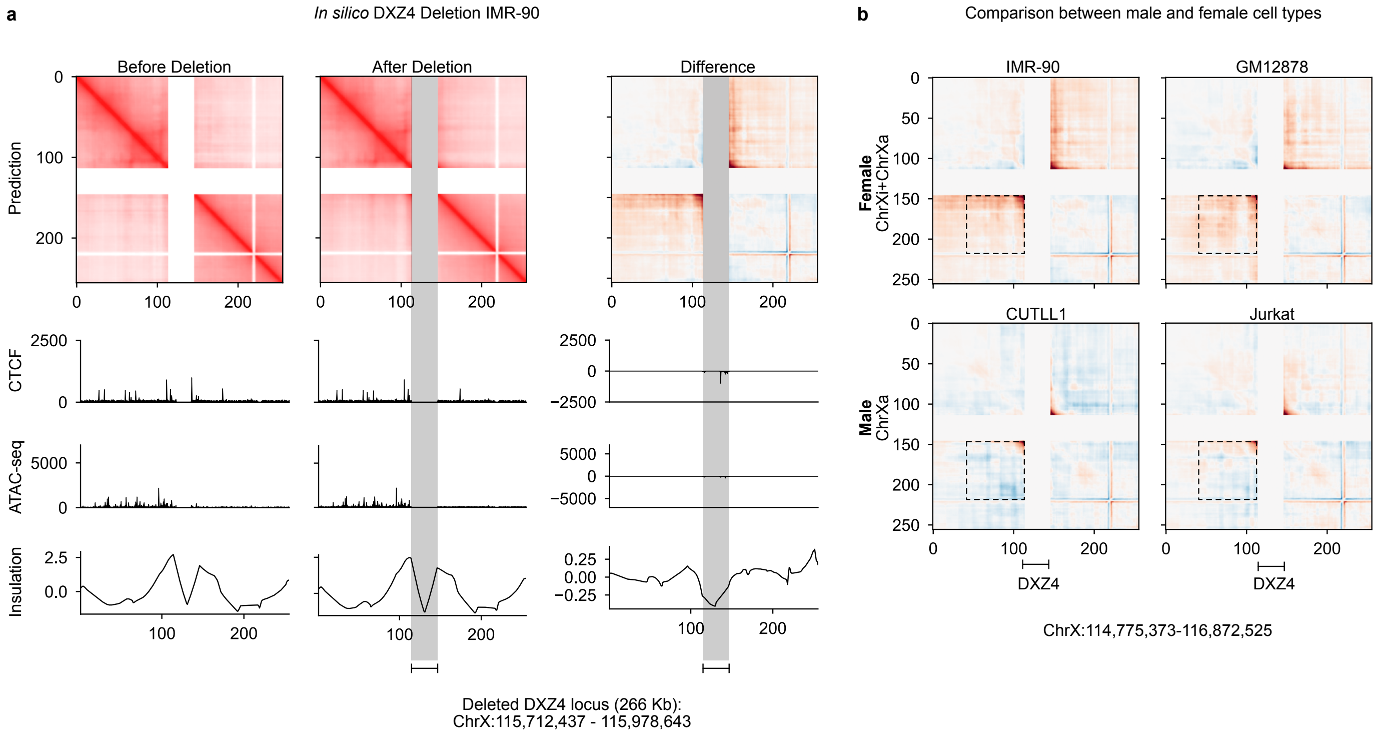

Supplementary Figure 17: Predicting X chromosome organization changes upon DXZ4 deletion prediction in male and female cell types. a, Chromatin organization changes upon *in silico* deletion of a 266Kb repeats at the DXZ4 locus in IMR-90, a female cell line. The perturbed region mimics the experimental knock-out in Darrow *et al*[42]. The deleted region is indicated by a gray bar. **b**, Chromatin organization changes upon in silico deletion of the DXZ4 locus in two female cell lines (IMR-90, GM12878), and two the male cell lines (bottom: CUTLL1, Jurkat). Deleting DXZ4 locus led to substantial loss of insulation at the two flanking regions of DXZ4 locus in the female cell lines, while the effect was very minimal in the male cell lines, supporting the role of DXZ4 in regulation X chromosome inactivation. Interaction regions are denoted by dotted boxes.

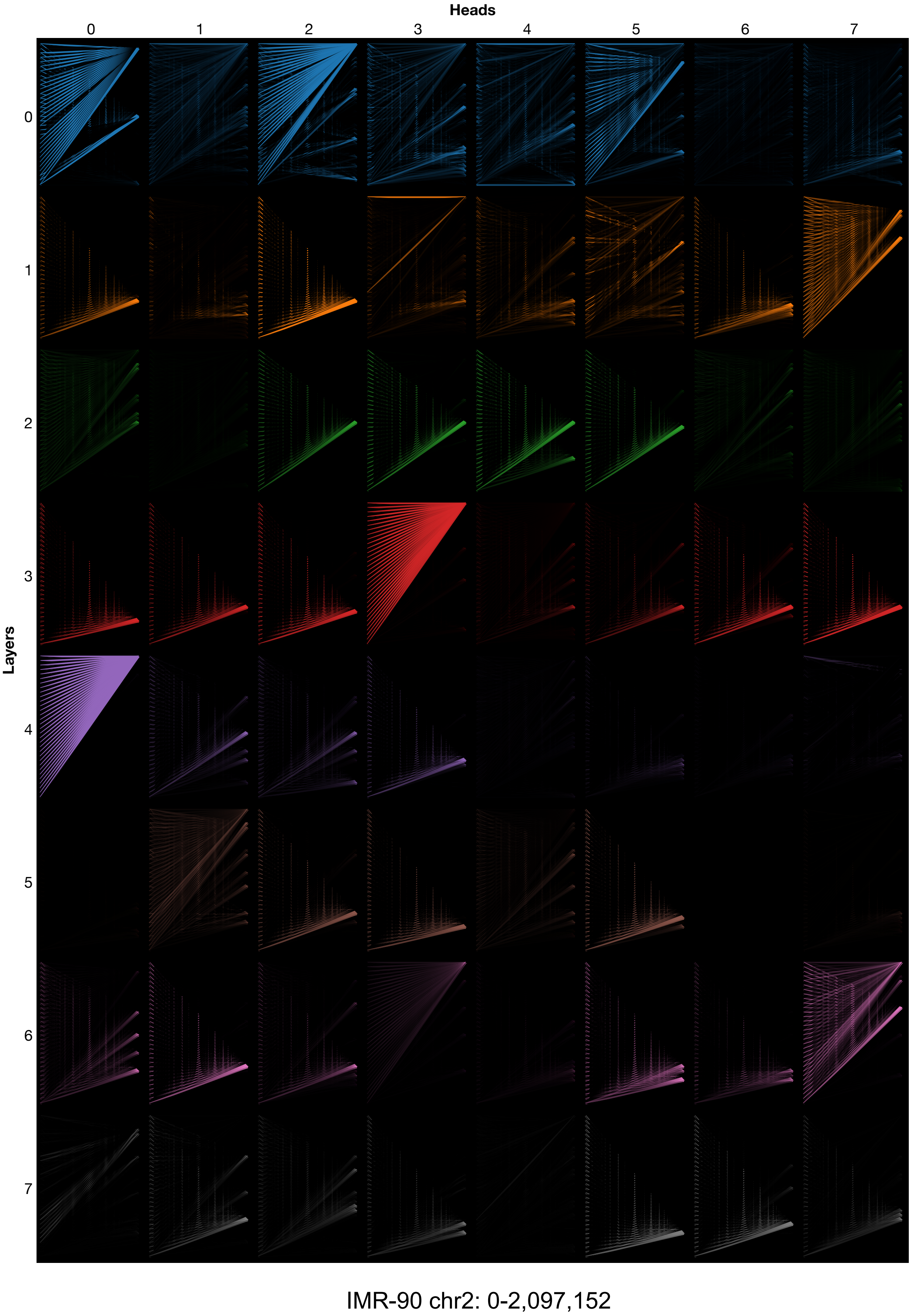

Supplementary Figure 18: Attention weights generated from the transformer module of C.Origami. A detailed view of the attention weights in eight heads (columns) across eight layers (rows), generated by the BertViz package[59]. The y axis of each row represents a 2Mb genomic distance. Brightness of the line segment between two different locations denotes interaction intensity.

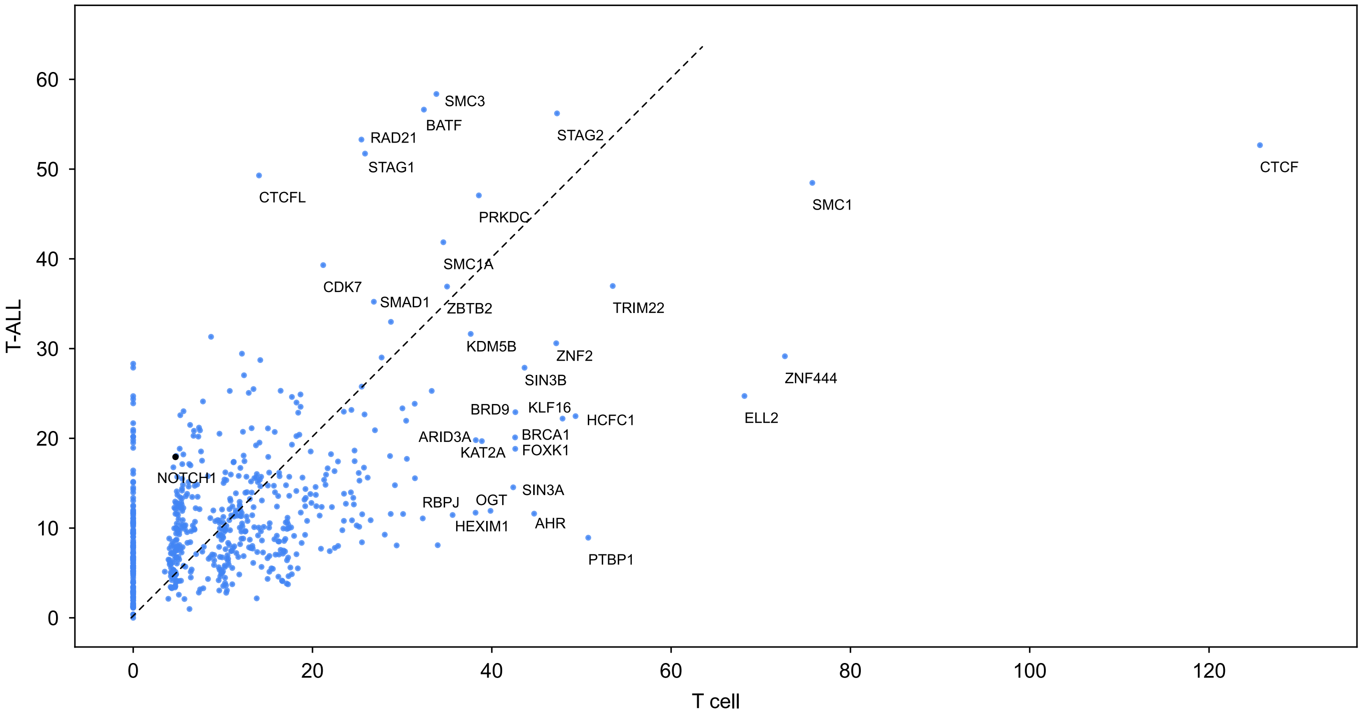

Supplementary Figure 19: Scatter plot of *trans*-acting factor binding enrichment in ISGS-identified impactful elements in T-ALL and normal T cells. Odds ratio of enrichment between T-ALL and normal T cells were plotted on the y axis and x axis, respectively. T-ALL odds ratio was aggregated from enrichment in CUTLL1 and Jurkat. Only factors with odds ratio larger than 35 were labeled, except NOTCH1 which was highlighted for comparison with CDK7 (referring to Figure 6).

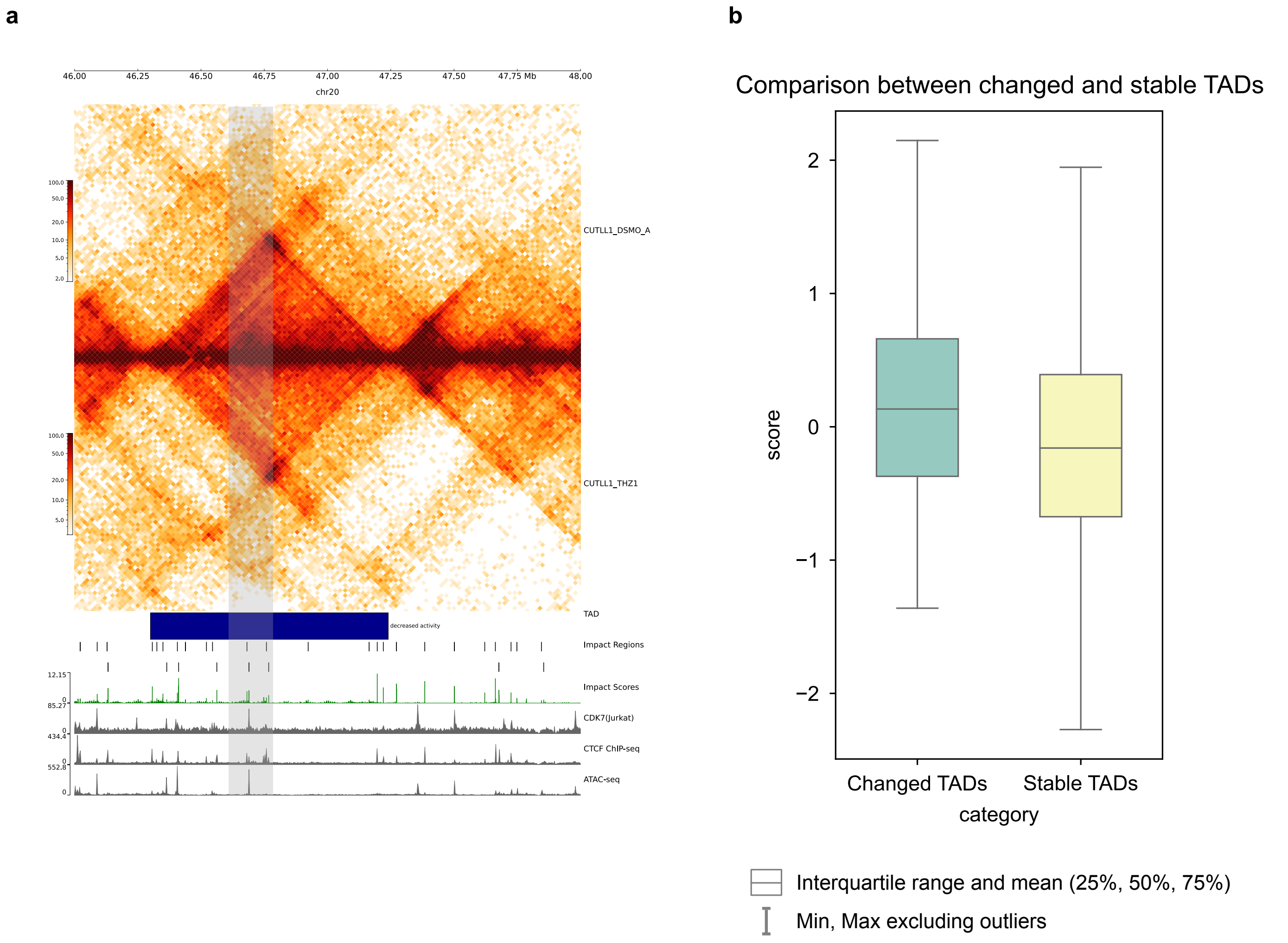

Supplementary Figure 20: Overlap between impactful elements and CDK7-inhibition induced TAD changes. a, An example of TAD with decreased activity. Grey bar indicates a prominent decrease in interaction in the CDK7-inhibition (+THZ1) group. The TAD intensity plots were aligned with impactful regions, impactful scores, CDK7 ChIP-seq, CTCF ChIP-seq, and ATAC-seq signals from top to bottom. **b**, Impact score of DNA elements in changed TADs and stable TADs determined from pharmaceutical inhibition of CDK7. The overall impact scores in the changed TADs are significantly higher (independent two-sided t-test, p-value = 1.72e-05).
